# Chemical speciation of MeHg^+^ and Hg^2+^ in aqueous solution and HEK cells nuclei by means of DNA interacting fluorogenic probes†
†Electronic supplementary information (ESI) available: Experimental details, characterization data, and additional experiments. See DOI: 10.1039/c5sc00718f


**DOI:** 10.1039/c5sc00718f

**Published:** 2015-04-30

**Authors:** Borja Díaz de Greñu, José García-Calvo, José Cuevas, Gabriel García-Herbosa, Begoña García, Natalia Busto, Saturnino Ibeas, Tomás Torroba, Blanca Torroba, Antonio Herrera, Sebastian Pons

**Affiliations:** a Department of Chemistry , Faculty of Science , University of Burgos , 09001 Burgos , Spain . Email: ttorroba@ubu.es; b Molecular Biology Institute of Barcelona , IBMB-CSIC , Barcelona Science Park , 08028 Barcelona , Spain

## Abstract

Selected new fluorogenic probes that interact in different ways with Hg^2+^ and MeHg^+^ have been prepared and used for the chemical speciation of both cations in aqueous solution as well as in HEK293 cells. The best selective speciation of Hg^2+^ and MeHg^+^ has been achieved by *in vitro* approaches based on fluorogenic probes supported in cultured cells, due to the particular sensitivity of the HEK293 cells to permeation by Hg^2+^, MeHg^+^ and the fluorogenic probes. In particular, MeHg^+^ was selectively detected in cell nuclei by probe JG45.

## Abstract 2

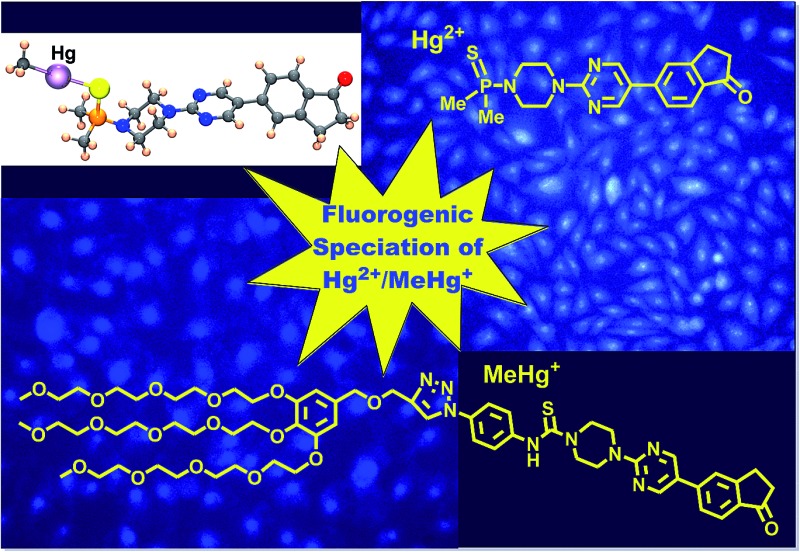
Speciation of Hg^2+^ and MeHg^+^ has been achieved by *in vitro* approaches with fluorogenic probes supported in cultured cells.

## Introduction

Environmental contamination by mercury is a serious issue because of the large amounts of mercury released into the environment by human activities such as coal-fired power stations, primary metals production and the chlor-alkali industry. Another major concern is the persistence of mercury in the environment, not only as the volatile mercury metal but also as the water-soluble mercury(ii) (Hg^2+^) and methylmercury(ii) (MeHg^+^) cations.^1^ Both species of Hg(ii) are strongly interconnected in the environment because of the natural cycle of mercury that maintains Hg^2+^ and MeHg^+^ concentrations in natural water resources, that are subsequently accumulated in food products produced in these regions.^2^ Undoubtedly, one of the best known neurotoxins is MeHg^+^, a ubiquitous environmental toxicant that leads to long-lasting neurological and developmental deficits in animals and humans,^3^ especially during prenatal exposure.^4^ In the aquatic environment MeHg^+^ is accumulated in fish, which represent a major source of human exposure.^5^ MeHg^+^ acts by blocking neurotransmitter release, interfering with transport of amino acids and ions, binding to sulfhydryl groups and inhibiting protein synthesis. MeHg^+^ has a high mobility in the body due to formation of a complex with the amino acid cysteine. Since this complex of MeHg^+^ and cysteine resembles the structure of the large and neutral amino acid methionine, it can enter the cell and exit as a complex with reduced glutathione, thus forming water-soluble complexes in tissues.^6^ Due to their inherent toxicity, Hg(ii) species have to be continuously monitored. There are many chemical probes that are useful for the fast detection of Hg^2+^ contamination. Usually, they work by complexation of Hg^2+^ by colorimetric or fluorogenic reagents.^7^ Complementing these methodologies, chemical dosimeters act by specific reactions with Hg^2+^, which subsequently undergo a colour or fluorescence change.^8^ Very few colorimetric or fluorogenic probes are able to detect MeHg^+^,^9^ and neither chemical probes nor dosimeters can discriminate speciation of Hg^2+^ and MeHg^+^,^10^ despite the enormous interest that MeHg^+^-induced neurotoxicity promotes^11^ and its imaging in living systems.^12^ Fluorogenic probes having sulfur atoms are able to interact with Hg(ii) species,^13^ therefore they could be employed to detect MeHg^+^ by mimicking its behaviour in cells, opening the way to biomimetic selective molecular probes for Hg(ii) species. Following this idea we decided to design and test some new sulfur-containing fluorogenic probes for their ability to selectively interact with Hg(ii) species. We have previously prepared Hg^2+^/MeHg^+^ colorimetric dosimeters^14^ and fluorescent reporters for significant analytes.^15^ In the latter case, the best examples included some recently reported14*a* charge-transfer fluorogenic probes bearing conjugated donor and acceptor groups in their structure, as well as a secondary amine group that was not involved in the charge transfer process. We thought that a sulfur-containing functional group in such position should exert a quenching effect on the initial fluorescence of the core structure by a photoinduced electron transfer (PET) from the sulfur atom. Subsequent interaction of the sulfur-containing group with thiophilic cations should therefore increase fluorescence of the probe, thus making this kind of compound suitable for the detection of Hg(ii) cations. Following this idea we now report the use of new fluorogenic probes for the selective detection and speciation of Hg^2+^ and MeHg^+^ in aqueous–organic mixtures and in HEK293 cells.

## Results and discussion

**Scheme 1:**
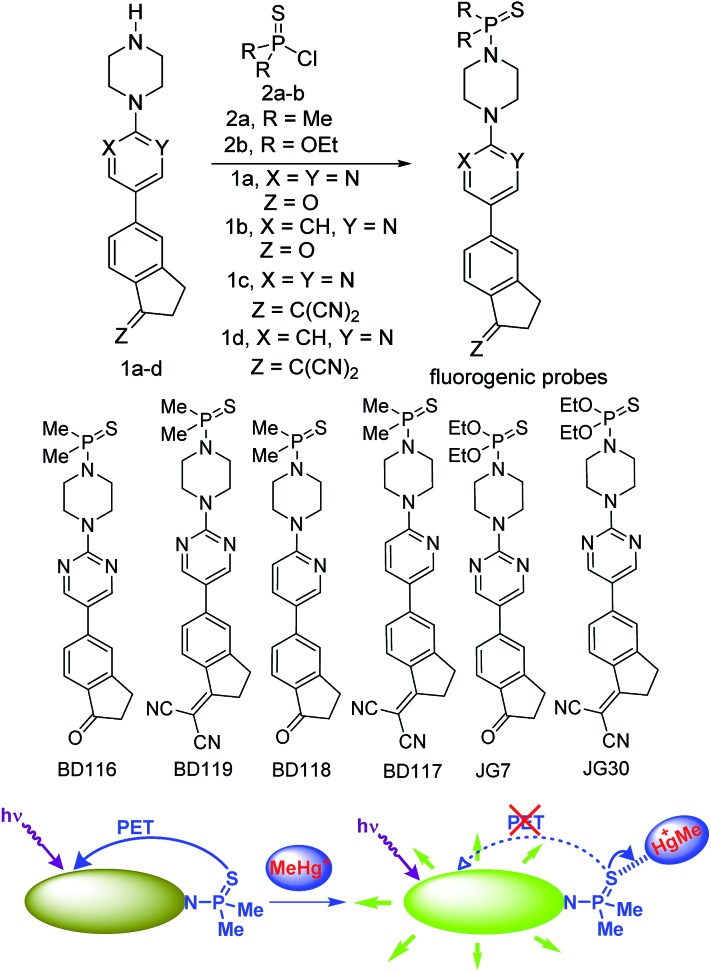
Synthesis of fluorogenic probes and their action.

For our current purpose we have designed new fluorogenic probes for Hg^2+^/MeHg^+^. The probes were prepared in fair yields by reaction of previously synthesized compounds **1a–d** and dimethylphosphinothioic chloride **2a** or *O*,*O*-diethyl phosphorochloridothioate **2b** in the presence of Hünig's base (Scheme 1). In this way we obtained derivatives BD116, BD119, BD118, BD117, JG7 and JG30, in which the fluorescence of the 5-(2-aminopyrimidin)-5-ylindane core is quenched by PET effect in some polar solvents (ESI†). Therefore interaction of thiophilic cations with the sulfur atom in those solvents should increase fluorescence of these compounds by an OFF–ON process (Scheme 1).

**Fig. 1:**
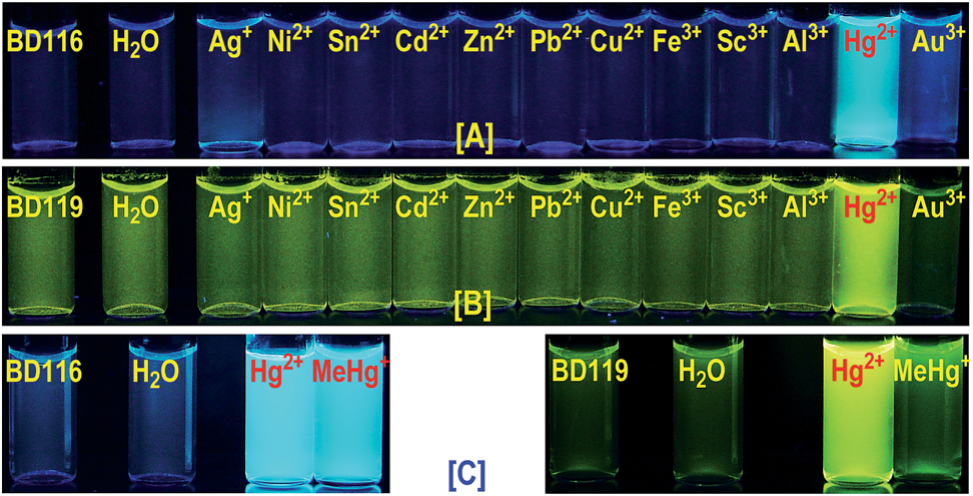
Observable colours under 366 nm UV light. The samples contain BD116 (A) or BD119 (B) 10^–4^ M solutions in MeOH–H_2_O 20 : 80 mixed with 2 equivalents of common cations as 5 × 10^–3^ M solutions in water. From left to right: probe, H_2_O, Ag^+^, Ni^2+^, Sn^2+^, Cd^2+^, Zn^2+^, Pb^2+^, Cu^2+^, Fe^3+^, Sc^3+^, Al^3+^, Hg^2+^, Au^3+^. (C) BD116, 10^–4^ M, MeCN–H_2_O 20 : 80 and BD119, 10^–4^ M, MeCN–H_2_O 40 : 60, from left to right: probe, H_2_O, Hg^2+^, MeHg^+^ (2 equivalents).

The ketone derivatives BD116, BD118 and JG7 are creamy solids giving colourless solutions whereas the dicyanomethylene derivatives BD119, BD117 and JG30 are yellow solids giving pale yellow solvatochromic solutions in the most common organic solvents. The solvent effects were experimentally studied in terms of the Lippert–Mataga equations in selected examples, showing a strong influence of general solvent effects and the absence of hydrogen-bonding interactions contributing to the Stokes shifts (ESI, pp. S34–S36†). We next tested 10^–4^ M solutions of the six fluorogenic probes in mixtures of different solvents (DMSO, DMF, acetone, MeCN, MeOH) and water (100%, 80 : 20, 60 : 40 and 20 : 80) by adding one to four equivalents of 5 × 10^–3^ M solutions of selected cations, as perchlorate or triflate salts, or anions, as tetrabutylammonium salts, in water and recorded all changes that the fluorogenic probes underwent with each analyte under a common TLC-UV light, *λ* = 366 nm, by qualitative measurements (photographs). As examples, photographs of BD116 and BD119 are shown in Fig. 1. These experiments showed that BD116 and BD119 were very efficient OFF–ON fluorogenic probes for the selective detection of Hg^2+^ in mixtures of methanol–water 20 : 80 v/v or acetonitrile–water 20 : 80 v/v with comparable efficiency. In contrast, the remainder of the probes were less efficient fluorogenic probes in those mixtures of solvents or showed partial interaction with competing thiophilic cations such as Ag^+^ and Au^3+^, when used in mixtures of solvents with decreasing proportion of water. None of the probes was sensitive to the presence of common anions neither in organic solvents nor in their mixtures with water.

We next tested 10^–4^ M solutions of probes BD116 and BD119 by adding one to four equivalents of 5 × 10^–3^ M solution of MeHg^+^ and compared the results to Hg^2+^. In this way, BD116 showed an enhanced fluorescence in the presence of MeHg^+^, as well as Hg^2+^, in all tested solvent mixtures, whereas BD119 produced a fluorescent response only in the presence of Hg^2+^. An immediate consequence is that the use of both probes should permit the speciation of MeHg^+^ and Hg^2+^, because of the unique sensitivity of BD116 to MeHg^+^ and Hg^2+^ in contrast to the lack of sensitivity of BD119 to MeHg^+^, which was only sensitive to Hg^2+^.

### Fluorescence quantitative experiments

**Fig. 2:**
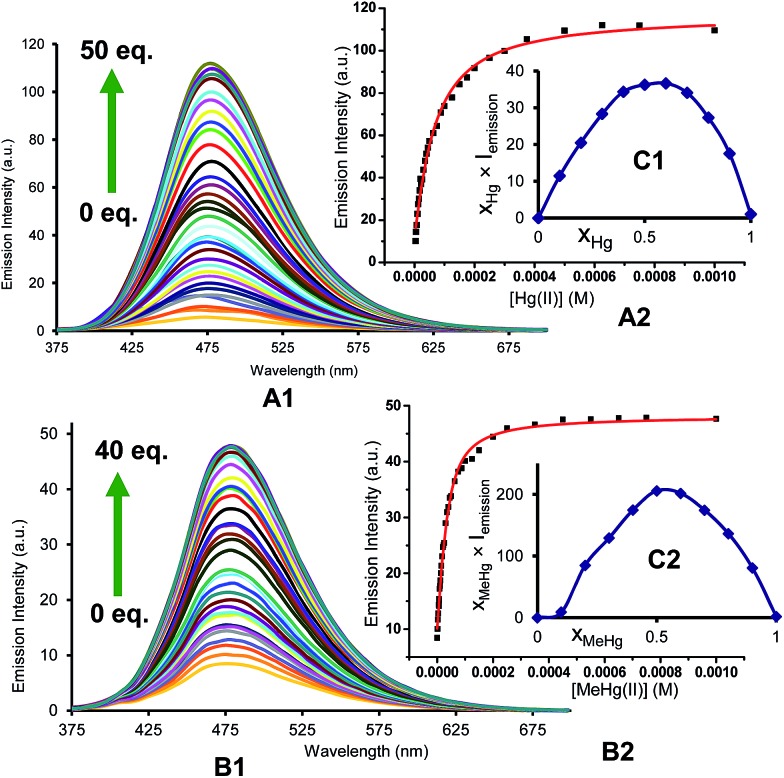
(A) Emission intensity spectra (*λ*_exc_ = 350 nm) and (B) titration profile (*λ*_em_ = 480 nm) of BD116, 2.5 × 10^–5^ M in MeOH–H_2_O 20 : 80, with several additions of (1) Hg^2+^ or (2) MeHg^+^. The captions show the equivalents of cation for each curve; (C) Job's plots of the complexes (1) BD116/Hg^2+^ and (2) BD116/MeHg^+^ (*λ*_exc_ = 350 nm) in MeOH–H_2_O 20 : 80.

For quantitative experiments we selected a concentration value of 2.5 × 10^–5^ M in which the absorption and the emission behaved in a linear fashion. UV/Vis and fluorescent titrations were carried out by successive additions of each analyte to the cuvette containing the probe until saturation of the signal, usually for concentrations ranging between 2.5 × 10^–6^ and 1.25 × 10^–3^ M. With these titrations we obtained stability constants, limits of detection and quantum yields in the presence of Hg^2+^ and MeHg^+^. Titrations of BD116, 2.5 × 10^–5^ M in MeOH–H_2_O 20 : 80 v/v, with Hg^2+^ showed a 20-fold increase of the initially weak fluorescent emission (*λ*_exc_ = 350 nm, *λ*_em_ = 480 nm) and a 2-fold increase of the quantum yield. Fitting of the emission intensity titration curves to a 1 : 1 complex equation gave a stability constant *K* = (1.9 ± 0.1) × 10^4^ M^–1^ for the reversible complexation between BD116 and Hg^+^. Replication of the titration five times afforded a detection limit of (5.0 ± 1.0) × 10^–6^ M for the determination of Hg^2+^. Titrations of BD116 with MeHg^+^ in the same conditions showed a 6-fold increase of emission band centred at 480 nm. Fitting of the emission intensity titration curves to a 1 : 1 complex equation gave a stability constant of *K* = (5.7 ± 0.4) × 10^4^ M^–1^, higher than the value obtained in the previous case, and replicates of the titration gave a detection limit of (9.9 ± 1.1) × 10^–6^ M for the determination of MeHg^+^, lower than the previous case. Fig. 2 shows the emission spectra and titration profiles for complexation of BD116 and Hg^2+^ or MeHg^+^. In turn, titrations of BD119 with Hg^2+^ in the same conditions showed a 6-fold increase of the emission band (*λ*_exc_ = 398 nm [isosbestic point], *λ*_em_ = 554 nm). Fitting of the emission intensity titration curves to a 1 : 1 complex equation gave a stability constant of *K* = (5.5 ± 0.5) × 10^3^ M^–1^, much lower than the value obtained for the complex BD116/Hg^2+^ (Fig. S27, ESI, p. S19†) and replicates of the titration gave a detection limit of (6.79 ± 1.9) × 10^–6^ M for the determination of Hg^2+^, lower than in previous case. The stoichiometry of binding processes were confirmed by Job's plot experiments of the fluorescence *vs*. the mole fraction of Hg^2+^ or MeHg^+^ of the three complexes (Fig. 2, Job's plot for BD116/Hg^2+^ and BD116/MeHg^+^, Fig. S99, ESI, p. S78† for BD119/Hg^2+^). In all cases the curves reached a maximum for *X*_Hg^2+^_ = *X*_MeHg^+^_ = 0.5, confirming the formation of 1 : 1 complexes.

### *T*-jump kinetic studies

**Fig. 3:**
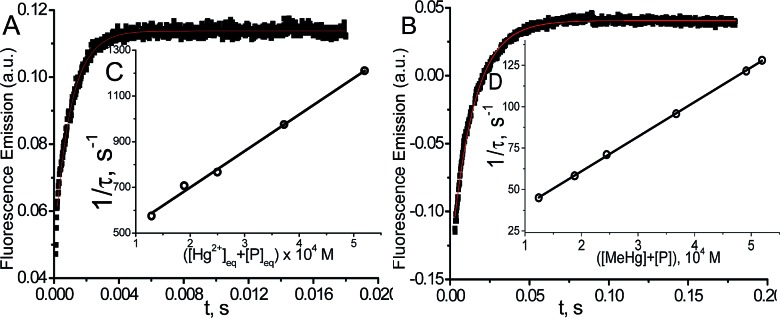
Kinetic curve (A) for BD116/Hg^2+^*C*_P_ = 2.5 × 10^–5^ M, *C*_Hg_/*C*_P_ = 15, *λ*_exc_ = 350 nm, *λ*_em_ = 460 nm and (B) for BD116/MeHg^+^*C*_P_ = 2.5 × 10^–5^, *C*_MeHg_/*C*_P_ = 20, *λ*_exc_ = 350 nm, *λ*_em_ = 460 nm. (C) Effect of the [Hg^2+^] + [P] and (D) [MeHg^+^] + [P] equilibrium concentrations on 1/*τ*. Continuous line is the linear fitting. Solvent MeOH–water 20 : 80, *I* = 0.1 M (NaClO_4_), pH = 7.0, *T* = 25 °C.

The kinetic study of the binding processes for BD116/Hg^2+^ and for BD116/MeHg^+^ were monitored by means of the ultra-fast *T*-jump technique in conducting solutions having a constant ionic strength (*I*) of 0.1 M NaClO_4_, a concentration of the probe 2.5 × 10^–5^ M in MeOH–H_2_O 20 : 80 v/v and under metal cation excess conditions ranging from 1.25 × 10^–4^ to 3.75 × 10^–4^ M to ensure pseudo-first order conditions. The processes were recorded by fluorescent emission in 0.7 cm optical length cuvettes each containing a total volume of 1 ml. The system, initially in equilibrium, was perturbed up to a final temperature of 25 °C, and the relaxation process was observed by fluorescent measurements. At least five replicates were performed for each composition probe-cation and the outliers were discarded. One monoexponential kinetic effect was observed in both cases. Examples of the recorded kinetic traces are provided in Fig. 3A and B. Fitting of the kinetic curves by exponential functions enabled us to obtain the reciprocal relaxation times, 1/*τ*. The kinetic analysis yielded the apparent binding reaction between the probe (P) and the metal, M (Hg^2+^ or MeHg^+^).1
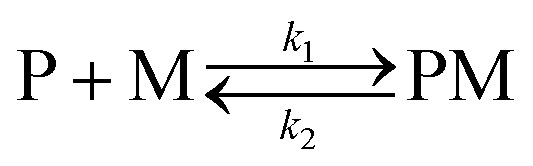



The linear dependence of the time constants (1/*τ*) on the reactants concentrations according to eqn (2) is shown in Fig. 3C and D. From the slope/intercept values we calculated the kinetic equilibrium constant *K* = *k*_1_/*k*_2_.2
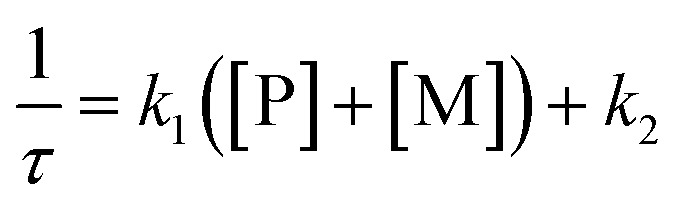



To a first approximation, the values of [P] and [M] are deduced from the thermodynamic values of the overall binding constant *K* obtained from static measurements, then they were re-evaluated from *K* = *k*_1_/*k*_2_ until convergence is reached. The linear fitting to eqn (2) showed that the mechanism of PM formation was in accordance to eqn (1) in both systems. The values of *k*_1_, *k*_2_ and *K* of the BD116/Hg^2+^ and BD116/MeHg^+^ systems are shown in Table 1.

**Table 1:** Apparent binding constant, *K*, and formation and dissociation kinetic constants, *k*_1_ and *k*_2_, obtained for the BD116/Hg^2+^ and BD116/MeHg^+^ systems at pH = 7.0, *I* = 0.1 M (NaClO_4_) and *T* = 25 °C

System	10^–5^*k*_1_/M^–1^ s^–1^	*k*_2_/s^–1^	10^–4^*K* (= *k*_1_/*k*_2_)/M^–1^
BD116/Hg^2+^	16 ± 1	380 ± 30	0.42 ± 0.06^*a*^
			0.41 ± 0.05^*b*^
BD116/MeHg^+^	2.1 ± 0.5	19.2 ± 0.5	1.10 ± 0.03^*a*^
			1.0 ± 0.1^*b*^

^*a*^Kinetics, *T*-jump fluorescence detection.

^*b*^Fluorescence titrations.

**Fig. 4:**
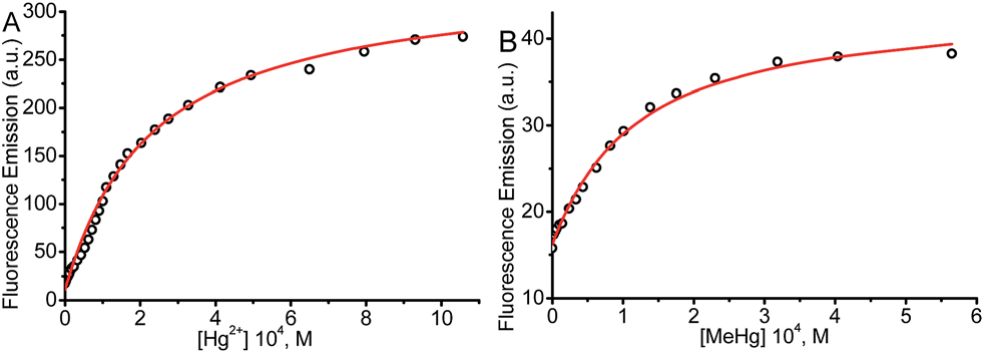
Fluorescence binding isotherm for (A) BD116/Hg^2+^ and (B) BD116/MeHg^+^ systems. [NaClO_4_] = 0.1 M, *λ*_exc_ = 350 nm, *λ*_em_ = 460 nm. Continuous lines are fitting according to eqn (3). The thermodynamic values obtained for *K* were nearly identical to those from *T*-jump measurements (Table 1).

The values for the equilibrium constant, *K*, denote greater affinity of the BD116 probe with MeHg^+^ than with Hg^2+^. Table 1 shows that the values of the rate constants for formation, *k*_1_, and dissociation, *k*_2_, of the BD116/Hg^2+^ complex were 8 and 20 times larger, respectively, than those of the BD116/MeHg^+^ complex. That is, the S···MeHg^+^ binding dissociates 20 times slower than S···Hg^2+^, revealing that in the equilibrium the hydrophobic nature of the interaction prevails. The *K* values were calculated also by fluorescence titration, yielding very similar results (Table 1). Micromolar amounts of M were added to the probe directly into the cuvette, measuring the fluorescence. Fig. 4 shows the binding isotherms according to the larger fluorescence of PM compared to that of P. The monophasic form of the binding isotherm revealed that only one type of complex metal/probe was operative, according to the kinetic mechanism (eqn (1)).3
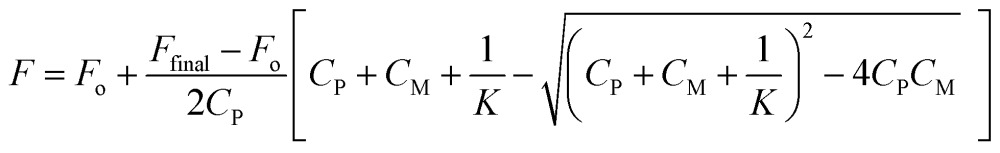



### Quantum mechanics calculations

**Fig. 5:**
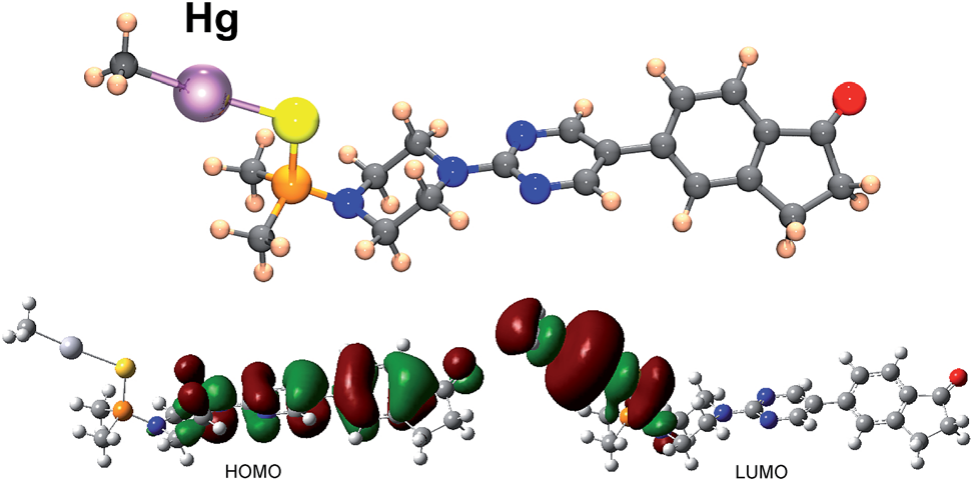
Structure, HOMO and LUMO of complex BD116/MeHg^+^ calculated by using DFT methodologies. HOMO displays a π bonding structure centered in the diaryl part and π antibonding contributions of the adjacent nitrogen p orbital and the carbonyl oxygen p orbital; LUMO is an σ antibonding interaction centred in the C–Hg–S fragment.

To throw light to the fact that only probe BD116 was able to form stable complexes with MeHg^+^ we performed DFT calculations by using the Gaussian 03 packing software.^16^ Full geometry optimization was performed at the B3LYP/6-31G**(Lanl2DZ for Hg) level of theory, performing the optimization in the gas phase. The preferred coordination process for all host and guest pairs took place through the sulfur atom of ligand molecules (a model of the complex BD116/MeHg^+^ along with the frontier orbitals is shown in Fig. 5). This fact was confirmed by ^1^H-NMR titration spectra of BD116 (5.0 × 10^–3^ M) in CD_3_CN with Hg^2+^, in which a downfield chemical shift for both methyl groups of the dimethylthiophosphinoic group was recorded as Hg^2+^ was added, indicating that the coordination process took place by the close sulfur atom (ESI, Fig. S100, pp. S78–S79†). The two close methylene groups of the piperazine moiety were also affected to a lesser extent by a weak downfield effect. DFT calculations also showed that complexation processes were spontaneous for BD116/Hg^2+^, BD116/MeHg^+^ and BD119/Hg^2+^ with calculated free energies of –91.8, –3.6 and –90.0 kcal mol^–1^ respectively, but in the case of BD119/MeHg^+^ the obtained free energy was positive (0.1 kcal mol^–1^), so the process was not spontaneous. This result was in agreement with the experimental behaviour of the probes.

### Speciation of MeHg^+^ and Hg^2+^ in aqueous solution and in live cells

**Fig. 6:**
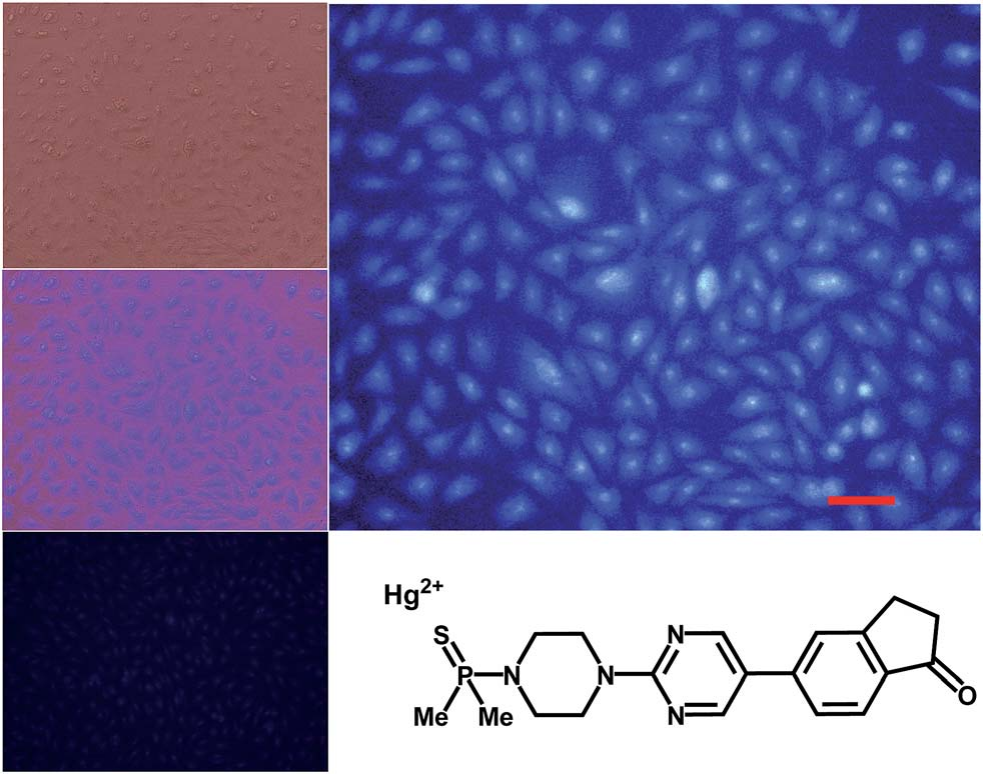
Fluorescence imaging of HEK293 cells in the presence of BD116 and 300 μM Hg^2+^. Top left: cells under visible light. Centre left: overlay maximum UV-Vis light. Bottom left: BD116 only. Top right: BD116 in the presence of 300 μM Hg^2+^. Scale bar: 100 μm.

The different selectivity of probes to Hg^2+^ and MeHg^+^ was used for the chemical speciation of each analyte in aqueous test samples containing mixtures of both cations, performed for the first time only with the help of fluorogenic probes by the standard addition methodology (ESI, pp. S73–S74†). Thus, BD119 permitted the selective analysis of Hg^2+^ and BD116 permitted the determination of the sum of Hg^2+^ + MeHg^+^. By comparison of the results obtained with each probe and a careful calibration of the fluorescent titrations, to compensate for the different sensitivity of each probe to each analyte, the concentration of each analyte was obtained. In this way, a representative sample containing 4.7 × 10^–6^ M [Hg^2+^] and 7.1 × 10^–6^ M [MeHg^+^] gave (4.8 ± 2.2) × 10^–6^ M [Hg^2+^] by titration with BD119 and (8.1 ± 2.5) × 10^–6^ M [MeHg^+^] from the difference between titrations with BD116 and BD119. BD116 was also used for the titration of stored solutions of MeHg^+^, with the surprising conclusion that aqueous solutions are fairly stable but methanol–water solutions slowly degrade on time by precipitation, a result confirmed by ICP-MS analysis of supernatant (ESI, pp. S75–76†). We next tested the usefulness of probes for the speciation of Hg(ii) species in live cells by incubating human embryonic kidney cell line 293 (HEK293) cells with BD116 or BD119 solutions (100 μM in PBS–MeOH 80 : 20) for 1 h at 37 °C. Then the plates were washed three times with PBS and incubated with Hg^2+^ (100 or 300 μM) in PBS for 1 h and the fluorescent emission was measured by using *λ*_exc_ = 388 or 358 nm. Cells remained viable after incubation in the presence of the probes, which were permeable to the cellular membrane. Controls of cells without probe were measured as blanks and the relative intensity of intracellular Hg^2+^ fluorescence was compared. Probe BD116 showed low levels of background intracellular fluorescence in the absence of Hg^2+^ but the intracellular fluorescence dramatically increased with addition of Hg^2+^ up to 300 μM. The emission was higher in the nucleus than in the cytoplasm of cells, due to a higher accumulation of the probe in the nucleus (Fig. 6).

Similar experiments with cell cultures incubated with BD116 and successive addition of MeHg^+^ gave very weak differences in fluorescence in the absence or in the presence of MeHg^+^. Probe BD119 showed only very little increase of fluorescence with addition of Hg^2+^ in the same conditions. There was no effect of the presence of MeHg^+^ in experiments using BD119.

### Interactions with DNA

The unusual accumulation of the probe BD116 in the nucleus suggested that the probe could interact with DNA. A deeper study on the interaction of BD116 and ctDNA alone, or incubated with MeHg^+^, was conducted at pH = 7.0 and ionic strength 0.1 M with absorbance, fluorescence, circular dichroism (CD), viscosity and differential scanning calorimetry techniques. For the BD116/ctDNA system (in the absence of MeHg^+^), Fig. S88A–D (ESI, p. S57†), show the formation of two types of BD116/DNA complexes, observed by both absorbance and fluorescence. The analysis of the BD116/ctDNA system was performed in DMSO–water mixtures containing upto 3% DMSO. The binding process was described by the apparent reaction (4):

4 P + DNA ⇌ PDNA,

where the probe (P) interacted with ctDNA to give the bound species (PDNA). The equilibrium constant was defined as *K* = [PD]/([P][D]). The titration curves were analysed at 344 nm according to eqn (5).5
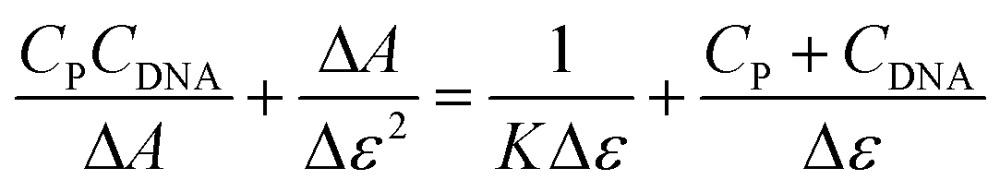



In eqn (5)*C*_DNA_ and *C*_P_ are the total dye and polymer concentrations, respectively; Δ*A* = *A* – *ε*_P_*C*_P_ is the change of absorbance (*A*) during titration, and *ε*_P_ = Δ*A*°/*C*_P_, where *A*° denotes the initial absorbance of the dye solution; Δ*ε* = *ε*_PDNA_ – *ε*_P_ is the amplitude of the binding isotherm. A similar equation was used in fluorescence titration experiments, by changing Δ*A* by Δ*Φ* and Δ*ε* by Δ*φ* in eqn (5). Fitting according to eqn (5) required an iterative procedure as Δ*ε* was not known. This was put equal to zero in the left term of the equation in a first approximation, and then calculated from the reciprocal slope of the plot; calculation was repeated until convergence was reached (usually three iterations only). A similar behavior was found when ctDNA was replaced by ctDNA incubated overnight with MeHg^+^ (Fig. S89A–D, ESI, p. S57†). Table 2 shows the values of the binding constants obtained for both systems by absorbance and fluorescence.

**Table 2:** Thermodynamic parameters for the BD116/DNA and BD116/(ctDNA + MeHg^+^) systems obtained from absorbance and fluorescence titrations

System	10^–5^*K*/M^–1^
BD116/ctDNA	4.36 ± 1.93^*a*^
	5.26 ± 1.94^*b*^
BD116/(ctDNA + MeHg^+^)	1.72 ± 0.33^*a*^
	2.36 ± 0.31^*b*^

^*a*^Absorbance.

^*b*^Fluorescence.

The binding constants were the same order of magnitude, even though the presence of MeHg^+^ reduces its value by a half due to the DNA/MeHg^+^ interaction. To verify the type of binding, viscometric, CD and DSC measurements were performed at constant ctDNA and varying the probe concentration. Fig. S90A and B (ESI, p. S58†) showed that, in the absence of MeHg^+^, the relative length of DNA remained the same over the whole concentration range, whereas only a small decrease in viscosity was observed in the presence of MeHg^+^. Fig. S90C and D (ESI, p. S58†) showed that the structural differences observed with CD were only modest, even though the molar dichroism changed when ctDNA was incubated with MeHg^+^ (larger separation of the bands at 278 nm). Fig. S90E and F (ESI, p. S58†) showed that the variation in the melting temperature obtained by DSC for the two systems remained unchanged upon increase in the probe concentration. The set of results leads to the conclusion that the BD116 probe interacts along the groove of DNA, both in the absence and in the presence of MeHg^+^. The small structural alterations caused by MeHg^+^ entailed diminution of the affinity of BD116 with ctDNA.

### Preparation of MeHg^+^ fluorogenic probes for live cell imaging

**Scheme 2:**
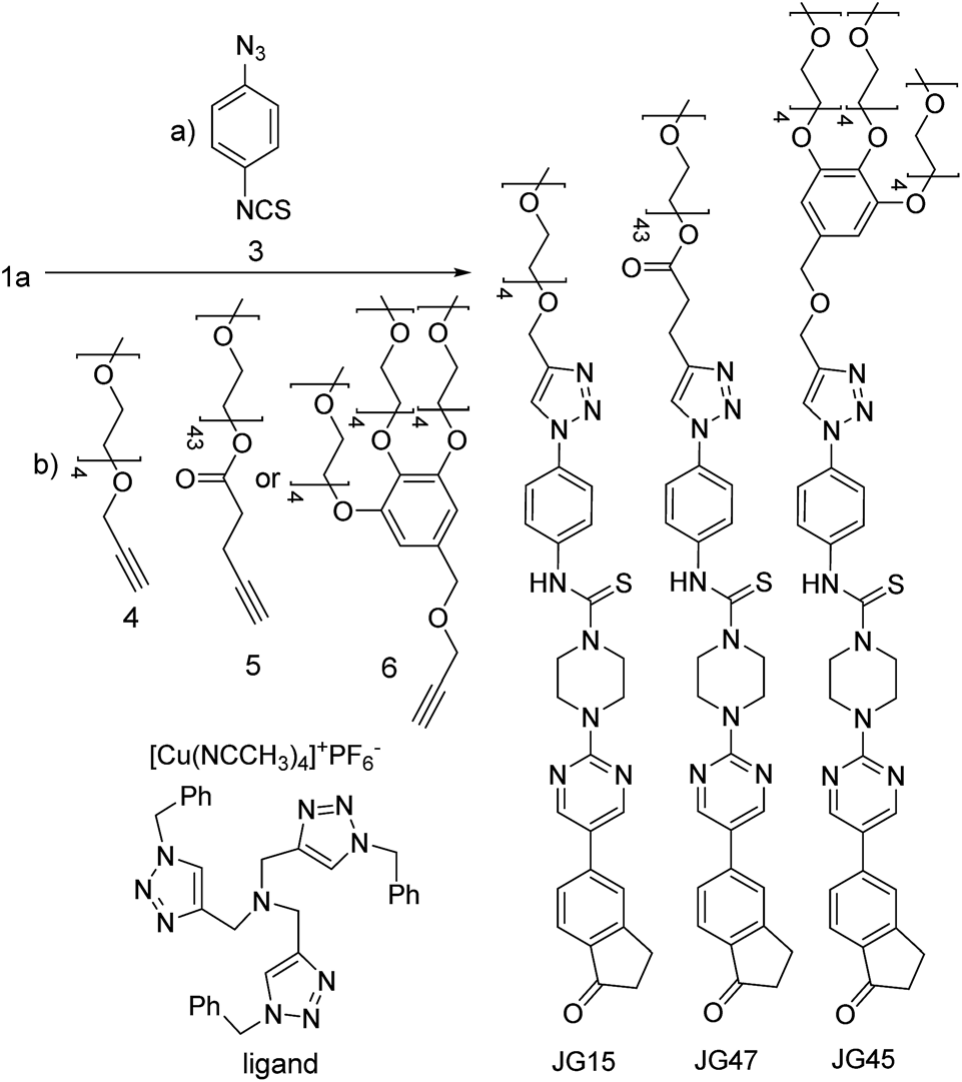
Synthesis of water-soluble fluorogenic probes.

All those experiments established that the structure of BD116 was a good starting point for the preparation of selective MeHg^+^ fluorogenic probes for live cell imaging. With this in mind, we synthesized new water soluble derivatives of **1a** that retained sensitivity to Hg(ii) derivatives (Scheme 2).

**Fig. 7:**
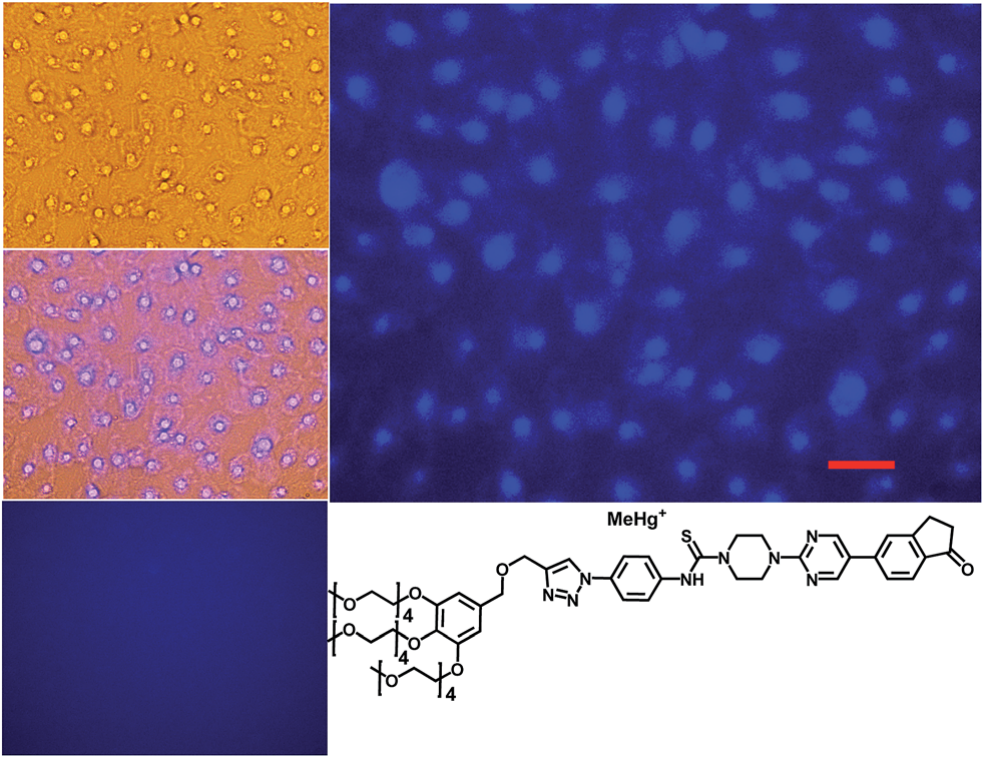
Fluorescence imaging of HEK293 cells incubated in the presence of JG45 and 400 μM MeHg^+^. Top left: cells under visible light. Middle left: overlay maximum UV-Vis light. Bottom left: cells with JG45 only. Right: cells with JG45 and 400 μM MeHg^+^. Scale bar: 100 μm.

The syntheses of probes were performed in good yields by reaction of **1a** with commercial 1-azido-4-isothiocyanatobenzene **3** followed by click reaction with 2,5,8,11,14-pentaoxaheptadec-16-yne **4**, usually employed to solubilize drugs for cell penetration,^17^ or the methoxy and 4-pentynoate-terminated poly(ethylene glycol) (*n*_(average)_ = 43) **5**, also used for bioconjugation,^18^ or the tri-PEG (2-propyn-1-yloxy)methylbenzene derivative **6**, closely related to a commonly used starting material for water solubilizing dendrimers,^19^ in all cases under catalysis by [Cu(NCCH_3_)_4_]^+^PF_6_^–^ and tris((1-benzyl-1*H*-1,2,3-triazol-4-yl)methyl)amine (see ESI†). The simplest derivative JG15 kept the OFF–ON fluorogenic selective sensing of Hg^2+^ in methanol–water 90 : 10 mixture, proving the versatility of these probes, but was not soluble enough to work in pure water. The probes JG47 and JG45 showed a similar sensitivity for Hg^2+^ in water as solvent, albeit JG45 was much more sensitive to MeHg^+^ in organic solvents (ESI, Fig. S81, p. S53†); therefore these two probes were tested for imaging and speciation of Hg(ii) species in image microscopy. HEK293 cells were incubated with JG47 or JG45 solutions (100 μM in PBS with Ca^2+^ and Mg^2+^) for 1 h at 37 °C. Then the plates were washed three times with PBS and incubated with Hg^2+^ (100–500 μM HgClO_4_) or MeHg^+^ (100–400 μM MeHgCl) in PBS + Ca^2+^ + Mg^2+^ for 1 h and the fluorescent emission was measured by exciting at *λ*_exc_ = 388 nm. Cells remained viable after incubation in the presence of the probes, which were permeable to the cellular membrane. Controls of cells without probe were measured as blanks and the relative intensity of intracellular Hg^2+^ and MeHg^+^ fluorescence were compared. Probes JG47 or JG45 showed low levels of background intracellular fluorescence in the absence of Hg^2+^ or MeHg^+^. There was very little change in the intracellular fluorescence when JG47 was tested in the presence of Hg^2+^ or MeHg^+^. The change in fluorescence was also very small for JG45 and Hg^2+^. However, the intracellular fluorescence dramatically increased by addition of MeHg^+^ up to 400 μM to the HEK293 cells incubated with JG45. The emission was observed almost exclusively in the nucleus of cells (Fig. 7) with very dim fluorescence in the cytoplasm.

Actually, in this case, the selective enhancement of fluorescence of probe JG45 in the presence of MeHg^+^ inside the nuclei of HEK293 cells was due to lipophilicity of both MeHg^+^ and JG45, which tended to concentrate in the nucleus of cells, acting as an optimum lipophilic environment for interaction of probe and cation. This fact was a reflection of the previously observed behaviour of JG45 in organic solvents and constitutes a new paradigm for the design of selective fluorescent probes for imaging MeHg^+^ on the basis of a sensitivity linked to lipophilicity.

## Conclusions

In summary, we have prepared new fluorogenic probes that interact in different ways with two closely related cations of high environmental concern, Hg^2+^ and MeHg^+^. The chemical probes were used for the chemical speciation of both cations in organic–aqueous solvents as well as in HEK293 cells. By far, the best selective speciation of Hg^2+^ and MeHg^+^ has been achieved by *in vitro* approaches based on the fluorogenic probes supported in cultured cells, due to the particular sensitivity of the HEK293 cells to permeation by Hg^2+^, MeHg^+^ and the fluorogenic probes. These achievements provide the biochemical bases to the understanding of MeHg^+^ selective detection and imaging, contributing to the discovery of endogenous and exogenous molecular probes that provide efficient means for speciation between Hg(ii) species.

## Supplementary Material

Supplementary information
