# Supplementary material for: Chemical speciation of MeHg+ and Hg2+ in aqueous solution and HEK cells nuclei by means of DNA interacting fluorogenic probes
Source: Chem Sci. 2015 Apr 30;6(7):3757–64. doi: 10.1039/c5sc00718f (PMC5707467; doi:10.1039/c5sc00718f)
Supplement: Supplementary file 1 [file SC-006-C5SC00718F-s001.pdf]

Electronic Supplementary Material (ESI) for Chemical Science.
This journal is © The Royal Society of Chemistry 2015

Supporting Information

Chemical Speciation of $MeHg^{+}$ and Hg^{2+} in Water Solutions and HEK Cells Nuclei by means of DNA Interacting Fluorogenic Probes.

Borja Díaz de Greñu, José García-Calvo, José V. Cuevas, Gabriel García-Herbosa, Begoña García, Natalia Busto, Saturnino Ibeas, Tomás Torroba,* Blanca Torroba, Antonio Herrera, Sebastian Pons*

Department of Chemistry, Faculty of Sciences, University of Burgos, 09001 Burgos, Spain. ttorroba@ubu.es, *Molecular Biology Institute of Barcelona, IBMB-CSIC, Barcelona Science Park, 08028 Barcelona, Spain.* spfbmc@ibmb.csic.es

Experimental Part……………………………………………………S2-S56

BD116/DNA interaction ……………………………………………S57-S58

Quantum Chemical Calculations …………………………………S59-S72

Speciation ………………………………………………………S73-S74

Stability of MeHg(II) solutions …………………………………S75-S77

Job's-Plot ………………………………………………………S78

^{1}H-NMR titration ……………………………………………S78-S79

Mass Spectrometry Titration ……………………………………S80

Experimental Part:

Materials and methods: The reactions performed with air sensitive reagents were conducted under dry nitrogen. The solvents were previously distilled under nitrogen over calcium hydride or sodium filaments. Melting points were determined in a Gallenkamp apparatus and are not corrected. Infrared spectra were registered in a Nicolet Impact 410 spectrometer in potassium bromide tablets. NMR spectra were recorded in Varian Mercury-300 and Varian Unity Inova-400 machines, in DMSO-d_6, $CDCl_3$, CD_3CN, CD_3OD. Chemical shifts are reported in ppm with respect to residual solvent protons, coupling constants ($J_{X\text{-}X'}$) are reported in Hz. Elemental analyses of C, H and N were taken in a Leco CHNS-932. Mass spectra were taken in a Micromass AutoSpec machine, by electronic impact at 70 eV. Quantitative UV-visible measures were performed with a Varian, Cary 300 Bio UV spectrophotometer, in 1 cm UV cells at 25ºC. Fluorescence spectra were recorded in a Varian Cary Eclipse or a Hitachi F-7000 FL spectrofluorometers, in 1 cm quarz cells at 25ºC. The pH values were measured at room temperature using a Metrohm 16 DMS Titrino pH meter fitted out with a combined glass electrode and a 3 M KCl solution as a liquid junction, which was calibrated with Radiometer Analytical SAS buffer solutions. Spectrophotometric measurements and thermal denaturalization experiments were performed on a Hewlett-Packard 8453A spectrophotometer (Agilent Technologies, Palo Alto, California) fitted out with diode array detection and computer-assisted temperature control systems. Fluorescence titrations with DNA were performed on a Shimadzu Corporation RF-5301PC spectrofluorometer (Duisburg, Germany) at λ_{exc} = 490 nm and λ_{em} = 555 nm. The titrations were performed by adding increasing amounts of ctDNA directly into the cell with the dye solution. The fast kinetic measurements were performed with a Dialog T-jump instrument built up according to the Riegler et al. prototype,[1] a temperature-jump apparatus for fluorescence measurements, in a 0.7 cm path-length cell, working in the fluorescence mode. The kinetic curves, collected with an Agilent (Santa Clara, CA) 54622A oscilloscope, were transferred to a PC and evaluated with the Table Curve program of the Jandel Scientific package (AISN software, Richmond, CA). The time constants were averaged out of ten-fold repeated kinetic experiments, the observed spread of time constants being 10%. Viscosity measurements were performed with an Ubbelodhe viscometer (Schott, Mainz, Germany) immersed in a thermostat at 25.0 ± 0.1°C. The sample viscosity was averaged out of triplicated readings of the flow time measured with a digital stopwatch The viscosity data were analyzed using $\eta/\eta_0 = (t - t_0)/(t_{DNA} - t_0)$, where t_0 and t_{DNA} are the solvent and ct-DNA solution flow times, respectively, whereas t is the flow time of the [2c']+ and DNA mixture. Mean values of replicated measurements were used to evaluate the sample viscosity, η, and that of ct-DNA alone, η_0, the η/η_0 ratio being related to the relative DNA length according to $L/L_0 = (\eta/\eta_0)^{1/3}$.[2] Circular dichroism: (CD) spectra were recorded on a MOS-450 Bio-Logic spectrometer (Claix, France). The measurements were performed at 25 °C in 1.0 cm path-length cells. The aqueous solutions were prepared with doubly deionized water from a Millipore Q apparatus (APS; Los Angeles, California). Calf thymus DNA (ctDNA) (Sigma Aldrich) was dissolved in water and sonicated with a MSE-Sonyprep sonicator by applying 20 cycles of 10 s sonication and 20 s pause, at a 98 µm amplitude to suitable DNA samples (10 ml CT-DNA 2×10^{-3} M). The sonicator tip was introduced directly into a solution kept in an ice bath to minimize thermal effects. The agarose gel electrophoresis tests performed indicate that the polymer length was reduced to approximately 1000 base-pair fragments. Stock solutions were standardized spectrophotometrically using $\varepsilon = 13200\ M^{-1}cm^{-1}$ (expressed in molarity of base-pairs) at λ = 260 nm, I = 0.1 M (NaCl) and pH = 7.0 sodium cacodylate. The DNA concentration, expressed in molarity of base-pairs, is denoted as Cp. The ionic strength was adjusted using sodium chloride; sodium cacodylate, $(CH_3)_2AsO_2Na$, was used to keep the acidity constant at pH 7.0. Quantum Chemical Calculations: DFT calculations were performed with the hybrid method known as B3LYP, in which the Becke three-parameter exchange functional[3] and the Lee–Yang–Parr correlation functional were used,[4] implemented in the Gaussian 03 (Revision C.02) program suite.[5] The effective core potentials (ECPs) of Hay and Wadt with a double-z valence

[1] R. Rigler, C. R. Rabl and T. M. Jovin, *Rev. Sci. Instrum.,* 1974, **45**, 580.]

[2] G. Cohen and H. Eisenberg, *Biopolymers,* 1969, **8**, 45.

[3] A. D. Becke, *J. Chem. Phys.,* 1993, **98**, 5648.

[4] Lee, C. T.; Yang, W. T.; Parr, R. G. *Phys. Rev. B* **1988**, *37*, 785-789.

[5] M. J. Frisch, G. W. Trucks, H. B. Schlegel, G. E. Scuseria, M. A. Robb, J. R. Cheeseman, J. Montgomery, J. A., T. Vreven, K. N. Kudin, J. C. Burant, J. M. Millam, S. S. Yengar, J. Tomasi, V. Barone, B. Mennucci, M. Cossi, G. Scalmani, N. Rega, G. A. Petersson, H. Nakatsuji, M. Hada, M. Ehara, K. Toyota, R. Fukuda, J. Hasegawa, M. Ishida, T. Nakajima, Y. Honda, O. Kitao, H. Nakai, M. Klene, X. Li, J. E. Knox, H. P. Hratchian, J. B. Cross, C. Adamo, J. Jaramillo, R. Gomperts, R. E. Stratmann, O. Yazyev, A. J. Austin, R. Cammi, C. Pomelli, J. W. Ochterski, P. Y. Ayala, K. Morokuma, G. A. Voth, P. Salvador, J. J. Dannenberg, V. G. Zakrzewski, S. Dapprich, A. D. Daniels, M. C. Strain, O. Farkas, D. K. Malick, A. D. Rabuck, K. Raghavachari, J. B. Foresman, J. V. Ortiz, Q. Cui, A. G. Baboul, S. Clifford, J. Cioslowski, B. B. Stefanov, G. Liu, A. Liashenko, P. Piskorz, I. Komaromi, R. L. Martin, D. J. Fox, T. Keith, M. A. Al-Laham, C. Y. Peng, A. Nanayakkara, M.

basis set (Lanl2DZ)[6] were used in describing Hg. The basis set for the main group elements was split-valence in all atoms [C, N, O, S, P and H, abbreviated as 6-31G(d,p)].[7] The structures were optimized and the energies of the structures were calculated at the level described above and transition states were confirmed by a vibrational analysis (one imaginary frequency) and IRC calculations.[8] Microscopy experiments: HEK 293T is a standard human cell line originated from human embryonic kidney cells. Cells were grown on uncoated T175 plastic flasks in Dulbecco's modified Eagles medium (DMEM, Gibco) with 10 % fetal bovine serum (FBS, Gibco) and 1 % Gentamicin (Gibco). Cells were cultured in 12-well plates 24 hours before each experiment. Before treatment, cells were washed twice with phosphate buffered saline (PBS) and then exposed to BD116 and BD119 probes (100 µM, in 80% PBS: 20% MeOH) or to JG45 and JG47 (100uM, in PBS $+Ca^{2+}+Mg^{2+}$) at 37 ºC for 1 hour. Cells were washed again with PBS to remove the probes from the extracellular media and incubated with $HgCl_2$ (100-500µM) or MeHgCl (100-400µM) for at least 1 hour. Control cultures without probe were exposed to similar conditions. After that, cultures were analyzed by using fluorescence microscopy (Inverted Microscope Leica DMI3000B, λ_{exc} = 358 nm) and a laser-scanning confocal microscope (SP5, Leica, λ_{exc} = 358 nm, λ_{em} = 570 nm).

Challacombe, P. M. W. Gill, B. Johnson, W. Chen, M. W. Wong, C. Gonzalez and J. A. Pople, *Gaussian 03, Revision C.02 ed.,* Gaussian, Inc., Wallingford, CT, 2004.

[6] P. J. Hay and W. R. Wadt, *J. Chem. Phys.,* 1985, **82**, 299.

[7] (*a*) P. C., Hariharan and J. A. Pople, *Theor. Chim. Acta,* 1973, **28**, 213; (*b*) M. M. Francl, W. J. Pietro, W. J. Hehre, J. S. Binkley, M. S. Gordon, D. J. DeFrees and J. A. Pople, *J. Chem. Phys.* 1982, **77**, 3654.

[8] C. Gonzalez and H. B. Schlegel, *J. Chem. Phys.* 1989, **90**, 2154.

SYNTHESIS OF BD116:

Synthesis of tert-butyl 4-(5-(1-oxoindan-5-yl)pyrimidin-2-yl)piperazine-1-carboxylate:

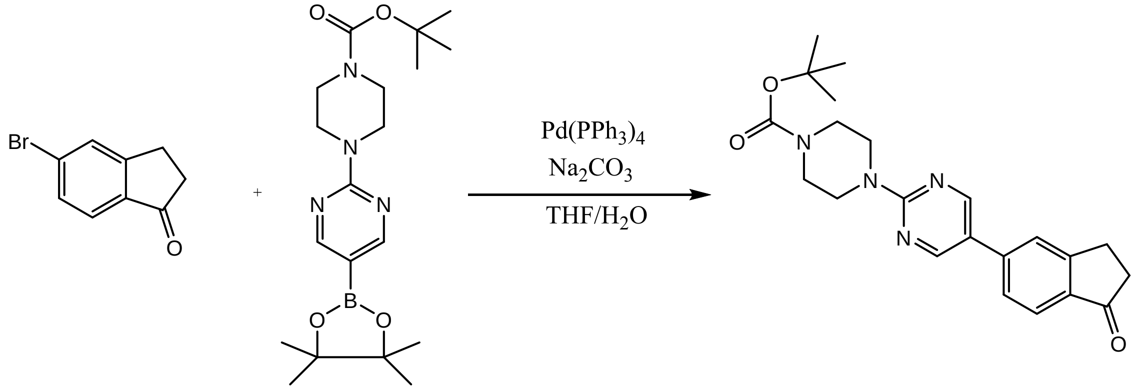

5-Bromoindanone (500 mg, 2.38 mmol), 2-[4-(*N*-Boc-piperazin-1-yl]pyrimidine-5-boronic acid pinacol ester (1003 mg, 2.57 mmol) and Na_2CO_3 (1500 mg, 14.2 mmol) were dissolved in a mixture of THF:H_2O (10:1, 33 mL) under stirring in a nitrogen atmosphere, then $Pd(PPh_3)_4$ (5 mg) was added and the resulting mixture was heated under reflux for 24 hours. Then the mixture was added to water (100 mL) and extracted with CH_2Cl_2 (3 x 50 mL). The combined organic extracts were dried (Na_2SO_4) and the solvent was evaporated under reduced pressure. The residue was purified by flash chromatography (silica, 3 x 30 cm), from CH_2Cl_2 to CH_2Cl_2:AcOEt (6:1), to get tert-butyl 4-(5-(1-oxoindan-5-yl)pyrimidin-2-yl)piperazine-1-carboxylate (930 mg, 95%) as a white solid, mp: 198-199 ºC (decomp.). IR (KBr, cm^{-1}): 2975, 2927, 2860, 1717 (C=O), 1599, 1525, 1421, 1362, 1310, 1277, 1251, 1181, 1025, 996, 944, 840. ^1H NMR ($CDCl_3$, 300 MHz) δ: 8.52 (s, 2H, H-C=N), 7.72 (d, *J* = 8.0 Hz, 1H, ArH), 7.49 (s, 1H, ArH), 7.42 (d, *J* = 8.0 Hz, 1H, ArH), 3.81 (t, *J* = 6.0 Hz, 4H, 2×CH_2) 3.46 (t, *J* = 6.0 Hz, 4H, 2×CH_2), 3.11 (t, *J* = 5.8 Hz, 2H, CH_2), 2.65 (t, *J* = 5.80 Hz, 2H, CH_2), 1.43 (s, 9H, 3×CH_3). ^{13}C NMR & DEPT ($CDCl_3$, 75 MHz) δ: 206.4 (C=O), 161.3, 156.4 (CH_{Ar}), 156.3, 154.9, 142.1, 136.0, 125.2 (CH_{Ar}), 124.7 (CH_{Ar}), 123.5 (CH_{Ar}), 122.2, 80.2, 43.9 (CH_2), 36.6 (CH_2), 28.6 (CH_3), 26.0 (CH_2). MS (EI) m/z (%): 394 (M^+, 39), 338 (24), 264 (22), 252 (34), 238 (100), 226 (32). HRMS (EI): calcd. for $C_{22}H_{26}N_4O_3$: 394.2005 (M^+); found: 394.1989.

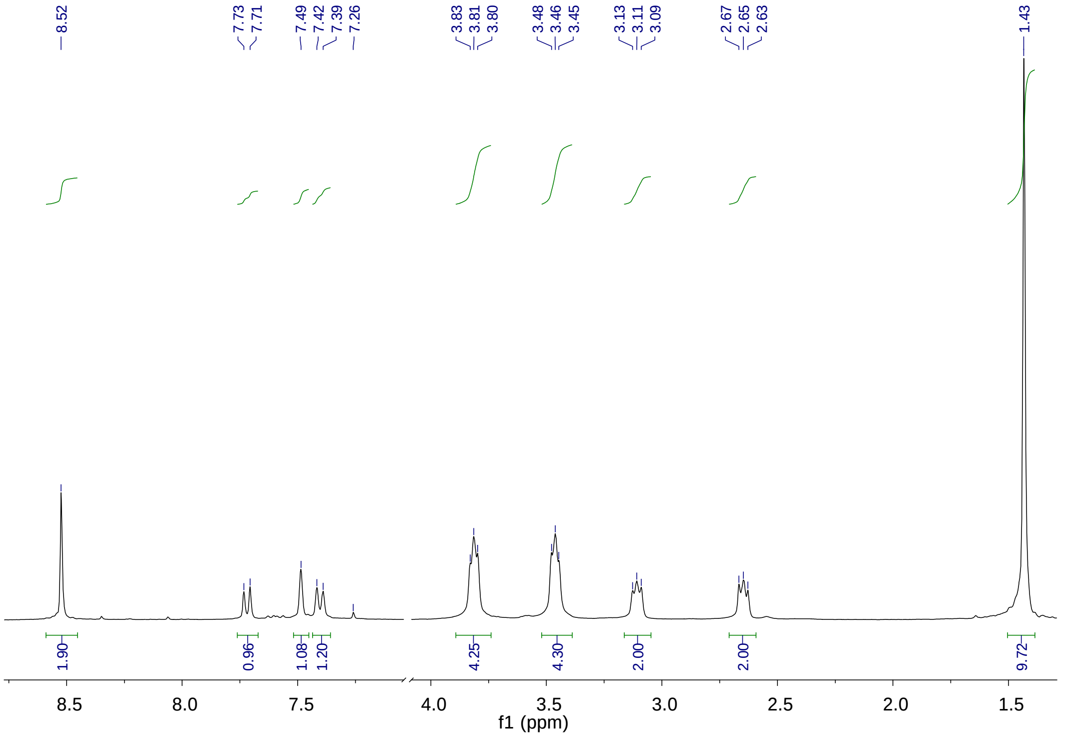

Figure S1: ^1H NMR ($CDCl_3$, 300 MHz) of tert-butyl 4-(5-(1-oxoindan-5-yl)pyrimidin-2-yl)piperazine-1-carboxylate

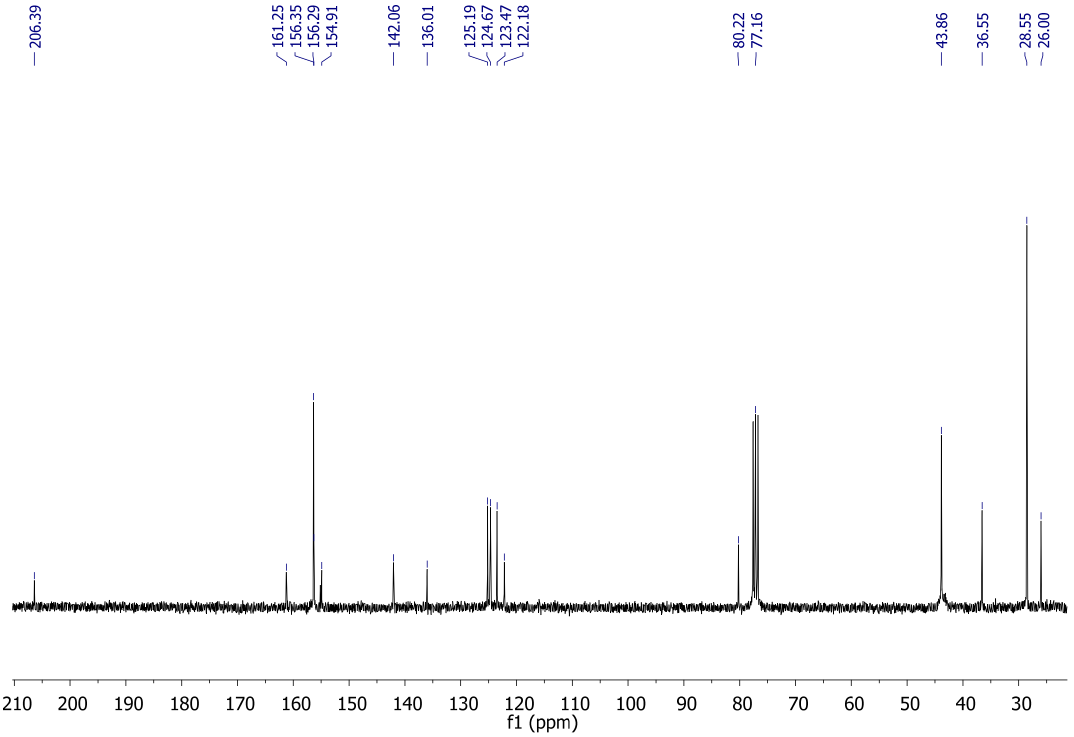

Figure S2: ^{13}C NMR ($CDCl_3$, 75 MHz) of tert-butyl 4-(5-(1-oxoindan-5-yl)pyrimidin-2-yl)piperazine-1-carboxylate

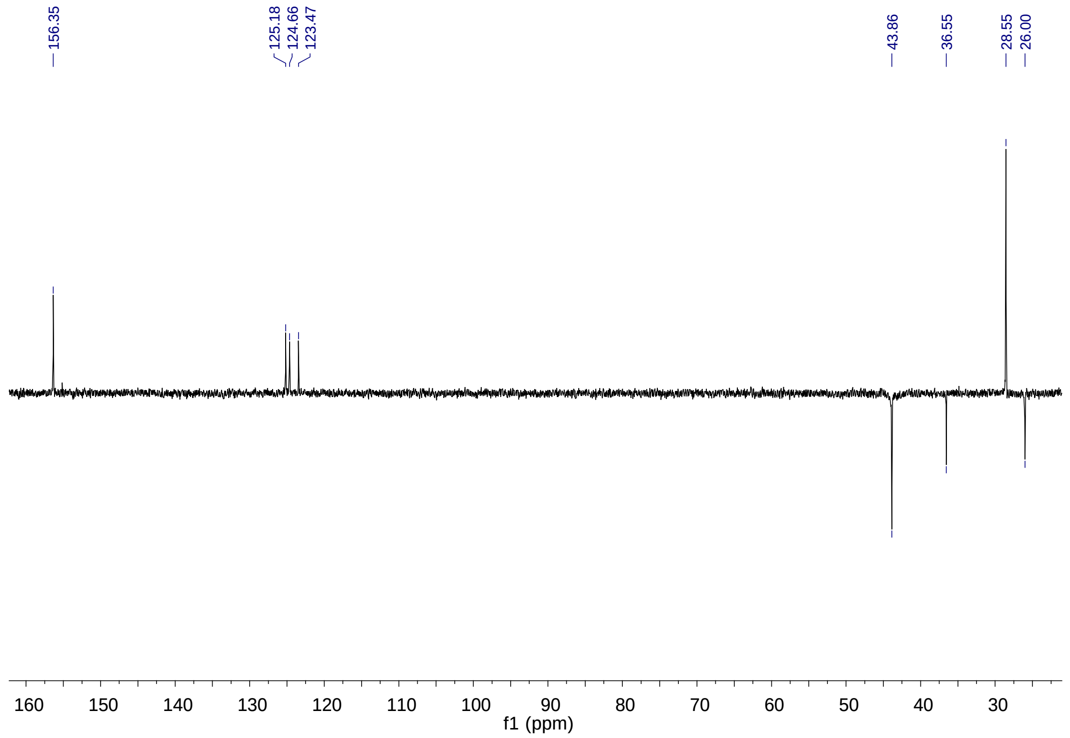

Figure S3: DEPT ($CDCl_3$, 75 MHz) of tert-butyl 4-(5-(1-oxoindan-5-yl)pyrimidin-2-yl)piperazine-1-carboxylate

Synthesis of 5-(2-(piperazin-1-yl)pyrimidin-5-yl)indan-1-one 1a:

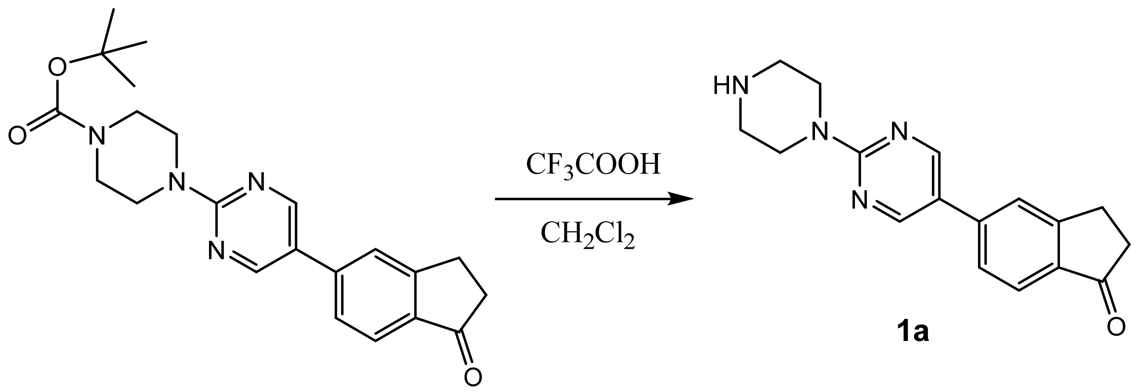

Trifluoroacetic acid (10 mL, ρ= 1.489 g/mL, 130.59 mmol) was added dropwise with stirring to a solution of tert-butyl 4-(5-(1-oxoindan-5-yl)pyrimidin-2-yl)piperazine-1-carboxylate (874 mg, 2.22 mmol) in CH_2Cl_2 (20 mL) and the resulting mixture was stirred at room temperature for 15 minutes. Then the mixture was added to water (50 mL), basified to pH= 10 with 5% NaOH and extracted with CH_2Cl_2 (4 x 50 mL). The combined organic extracts were dried (Na_2SO_4), the solvent was evaporated under reduced pressure and the residue was recrystallized from CH_2Cl_2-MeOH (5:1) to get 5-(2-(piperazin-1-yl)pyrimidin-5-yl)indan -1-one (617 mg, 95%) as a white solid, mp: 189-190 ºC (decomp.). IR (KBr, cm^{-1}): 3221 (N-H), 2940, 2843, 1706 (C=O), 1603, 1502, 1455, 1363, 1307, 1260, 1117, 940. ^1H NMR ($CDCl_3$, 300 MHz) δ: 8.55 (s, 2H, H-C=N), 7.76 (d, J = 8.0 Hz, 1H, ArH), 7.52 (s, 1H, ArH), 7.44 (d, J = 8.0 Hz, 1H, ArH), 3.84 (d, J = 6.0 Hz, 4H, 2×CH_2), 3.15 (d, J = 6.0 Hz, 2H, CH_2), 2.92 (d, J = 6.0 Hz, 4H, 2×CH_2), 2.69 (d, J = 6.0 Hz, 2H, CH_2), 1.84 (s, 1H, NH). ^{13}C NMR & DEPT ($CDCl_3$, 75 MHz) δ: 206.3 (C=O), 161.3, 156.2 (CH_{Ar}), 156.2, 142.2, 135.8, 125.0 (CH_{Ar}), 124.5 (CH_{Ar}), 123.2 (CH_{Ar}), 121.5, 46.1 (CH_2), 45.1 (CH_2), 36.5 (CH_2), 25.9 (CH_2). MS (EI) m/z (%): 294 (M^+, 22), 252 (100), 238 (29), 226 (59). HRMS (EI): calcd. for $C_{17}H_{18}N_4O$: 294.1481 (M^+); found: 294.1482.

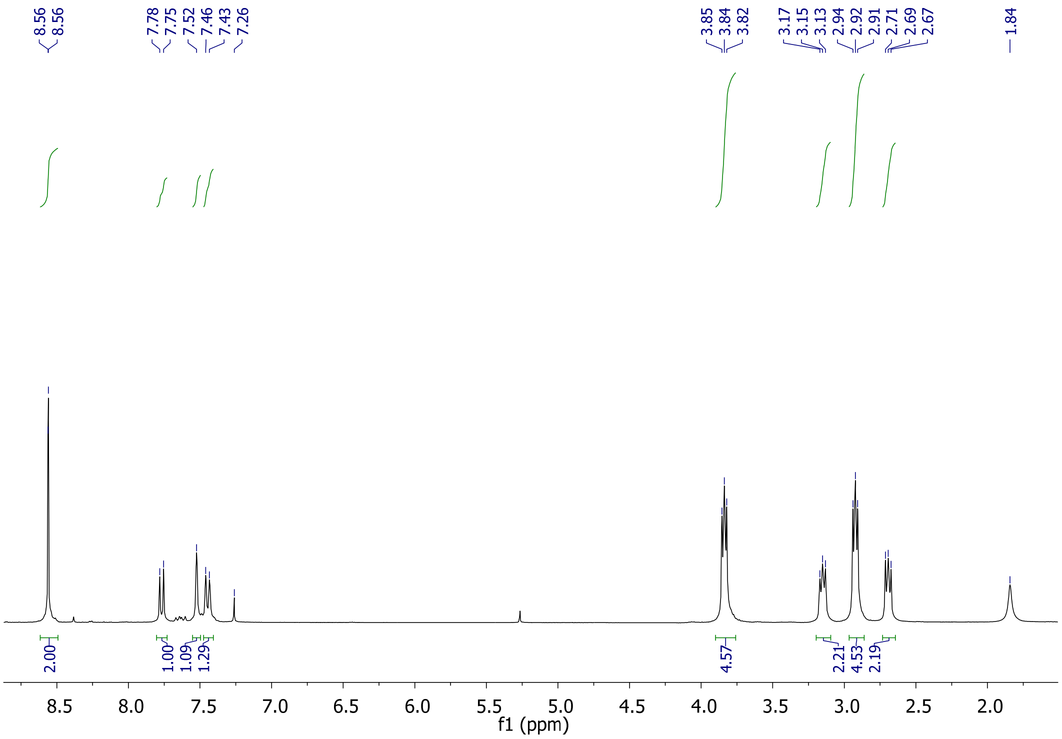

Figure S4: ^1H NMR ($CDCl_3$, 300 MHz) of 5-(2-(piperazin-1-yl)pyrimidin-5-yl)indan-1-one 1a

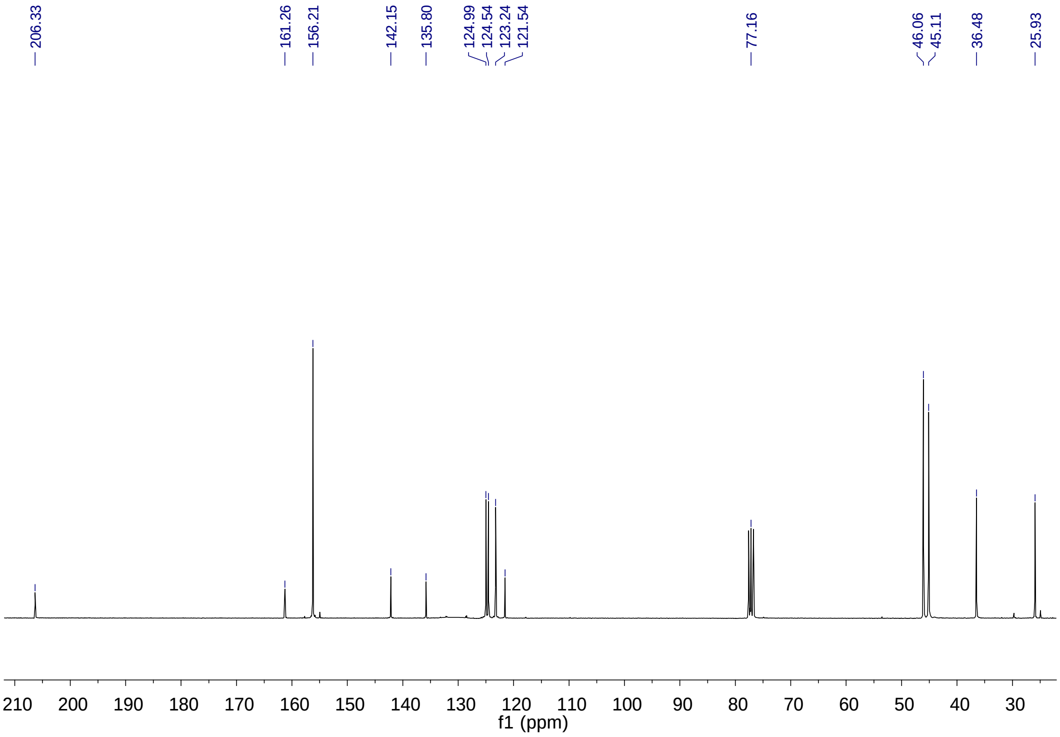

Figure S5: ^{13}C NMR ($CDCl_3$, 75 MHz) of 5-(2-(piperazin-1-yl)pyrimidin-5-yl)indan-1-one 1a

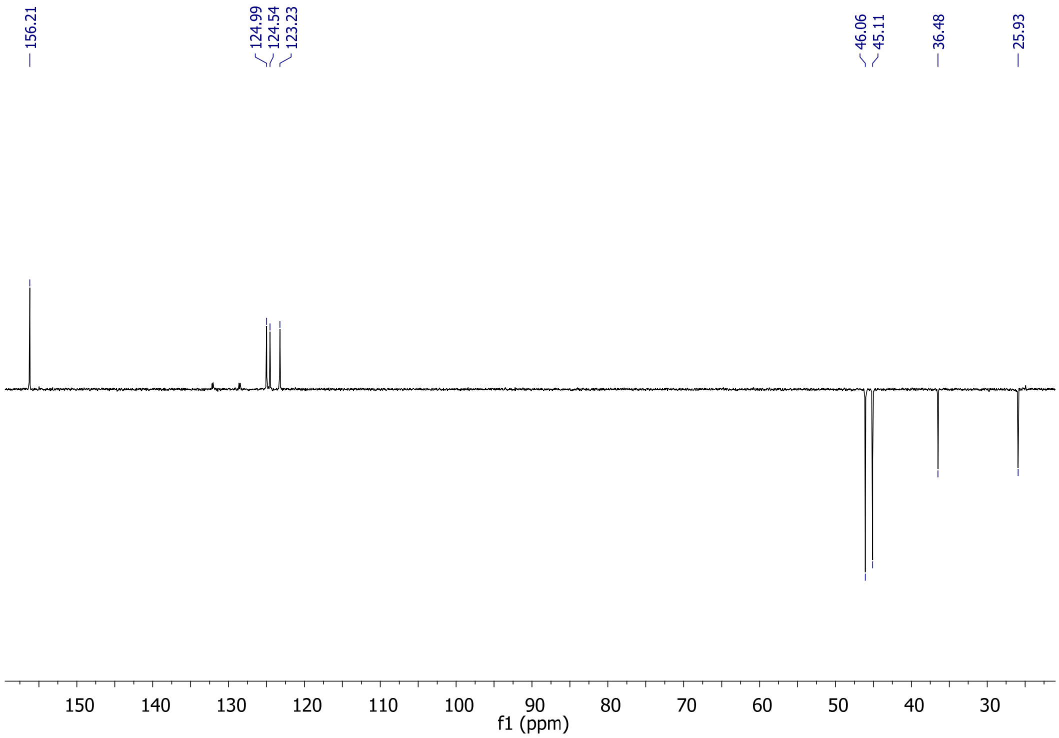

Figure S6: DEPT ($CDCl_3$, 75 MHz) of 5-(2-(piperazin-1-yl)pyrimidin-5-yl)-indan-1-one 1a

Synthesis of 5-(2-(4-(dimethylphosphorothioyl)piperazin-1-yl)pyrimidin-5-yl)-indan-1-one BD116:

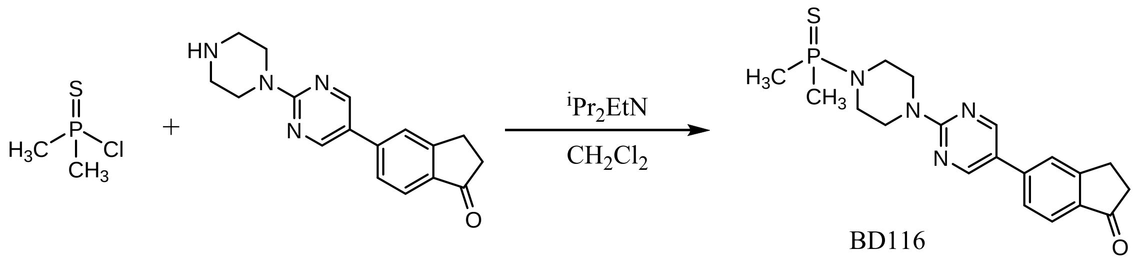

Dimethylthiophosphinic chloride (16.1 µL, ρ = 1.230 g/mL, 0.15 mmol) was added to a solution of 5-(2-(piperazin-1-yl)pyrimidin-5-yl)-indan-1-one **1a** (50 mg, 0.17 mmol) in CH_2Cl_2 (20 mL) in an ice bath. Then *N*,*N*-diisopropylethylamine (29.6 µL, ρ= 0.742 g/mL, 0.17 mmol) was added to the mixture and the resulting mixture was stirred for 15 minutes at 0 ºC, then for 20 minutes at room temperature and then for 40 minutes at room temperature. Then the solvent was evaporated under reduced pressure, the residue was washed up with hexane and the crude product was purified by flash chromatography (silica, 1 × 20 cm) from CH_2Cl_2 to CH_2Cl_2:MeOH (100:1.5), to get 5-(2-(4-(dimethylphosphorothioyl)piperazin-1-yl)pyrimidin-5-yl)-indan-1-one BD116 (56 mg, 85%) as a white solid. mp**:** 185-186 ºC (decomp.). IR (KBr, cm^{-1}): 3005, 2979, 2905, 2846, 1702 (C=O), 1599, 1510, 1440, 1362, 1314, 1258, 1114, 966, 740. ^{1}H NMR ($CDCl_3$, 300 MHz) δ: 8.58 (s, 2H, H-C=N), 7.78 (d, *J* = 8.0 Hz, 1H, ArH), 7.54 (s, 1H, ArH), 7.46 (d, *J* = 8.0 Hz, 1H, ArH), 3.91 (t, *J* = 5.0 Hz, 4H, 2×CH_2), 3.19-3.08 (m, 6H, 3×CH_2), 2.71 (t, *J* = 6.0 Hz, 2H, CH_2), 1.86 (s, 3H, P-CH_3), 1.82 (s, 3H, P-CH_3). ^{13}C NMR ($CDCl_3$, 75 MHz) δ: 206.3 (C=O), 160.9, 156.1 (2 × CH_{Ar}), 141.8, 135.8, 125.0 (CH_{Ar}), 124.4 (CH_{Ar}), 123.3 (CH_{Ar}), 122.1, 44.2 (CH_2), 44.0 (CH_2), 43.9 (CH_2), 36.4 (CH_2), 25.8 (CH_2), 21.7 (CH_3), 20.8 (CH_3). MS (EI) m/z (%): 386 (M^+, 37), 353 (19), 293 (24), 264 (100), 252 (34), 238 (28), 122 (57), 93 (51). HRMS (EI): calcd. for $C_{19}H_{23}N_4OPS$: 386.1330 (M^+); found: 386.1331.

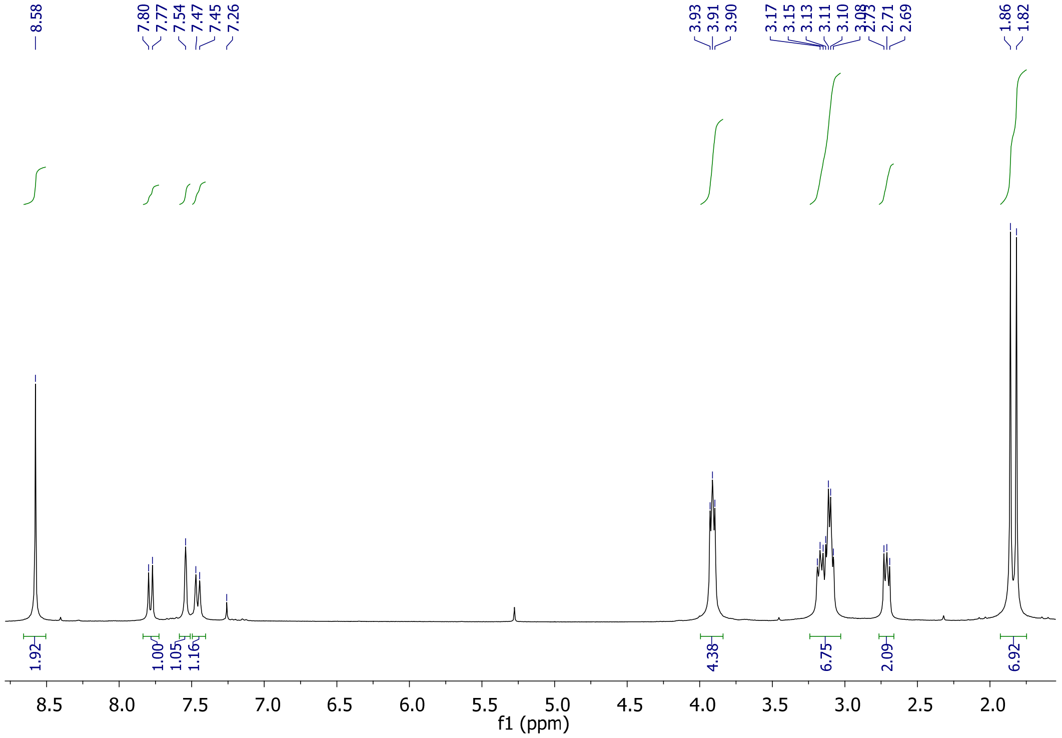

Figure S7: ^{1}H NMR ($CDCl_3$, 300 MHz) of 5-(2-(4-(dimethylphosphorothioyl)piperazin-1-yl)pyrimidin-5-yl)-indan-1-one BD116

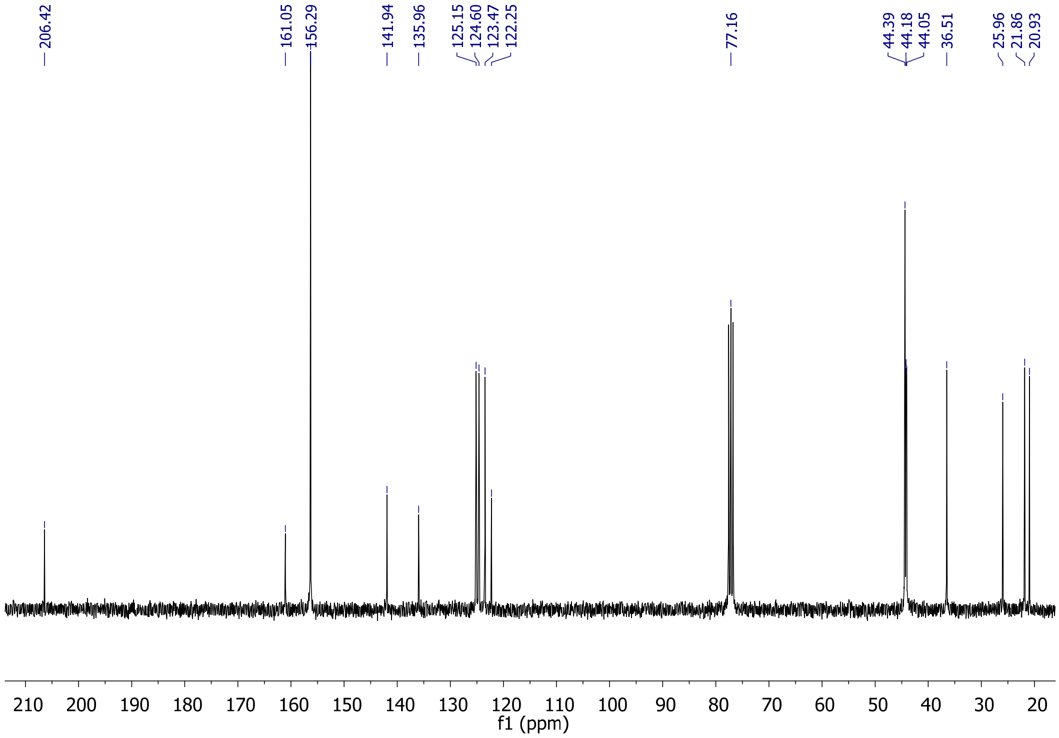

Figure S8: ^{13}C NMR ($CDCl_3$, 75 MHz) of 5-(2-(4-(dimethylphosphorothioyl)piperazin-1-yl)pyrimidin-5-yl)-indan-1-one BD116

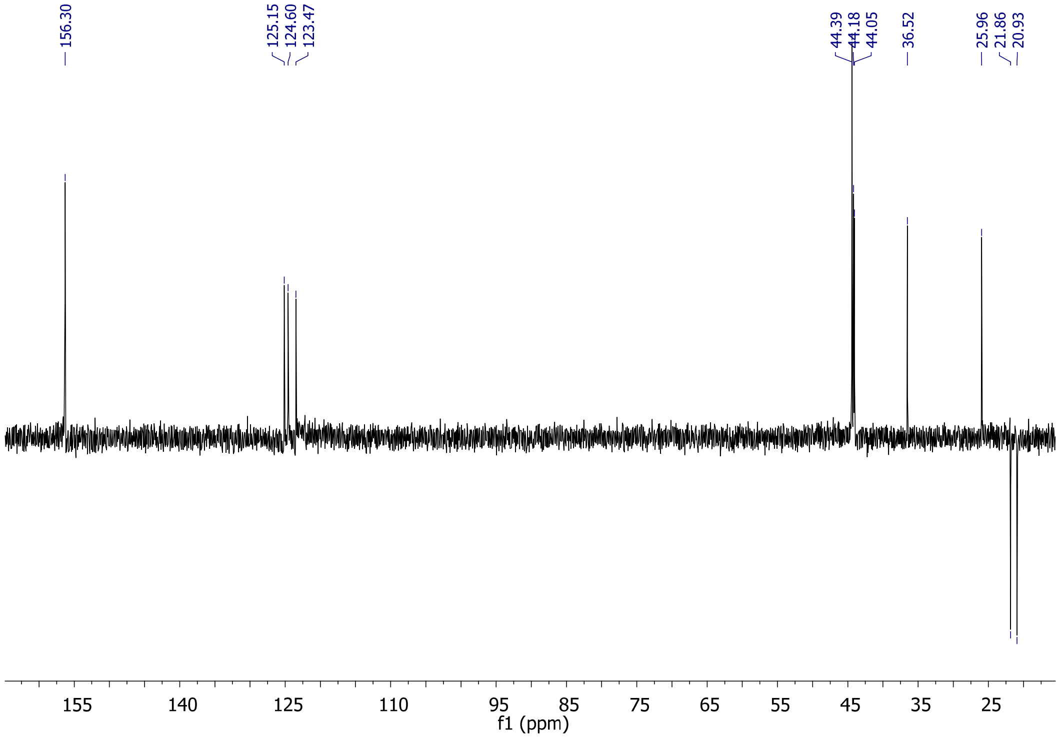

Figure S9: DEPT ($CDCl_3$, 75 MHz) of 5-(2-(4-(dimethylphosphorothioyl)piperazin-1-yl)pyrimidin-5-yl)-indan-1-one BD116

Solvatochromism

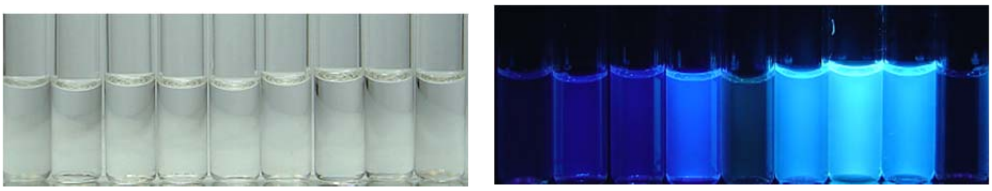

(1) (2) (3) (4) (5) (6) (7) (8) (9) (1) (2) (3) (4) (5) (6) (7) (8) (9)

(a) (b)

Figure S10: BD116 10^{-4} M in (1) Hexane, (2) Et_2O, (3) THF, (4) CH_2Cl_2, (5) MeOH, (6) MeCN, (7) DMSO, (8) DMF or (9) Acetone under (a) direct sunlight and (b) 366 nm light.

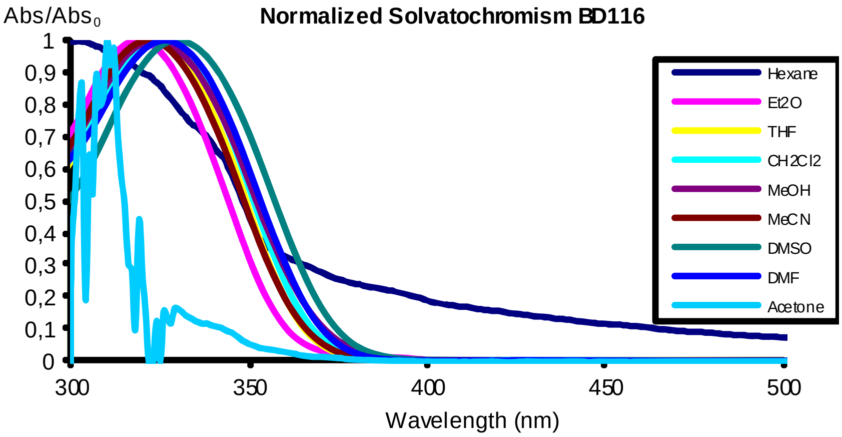

Figure S11: Normalized absorption spectra of **BD116** 10^{-4} M in different solvents.

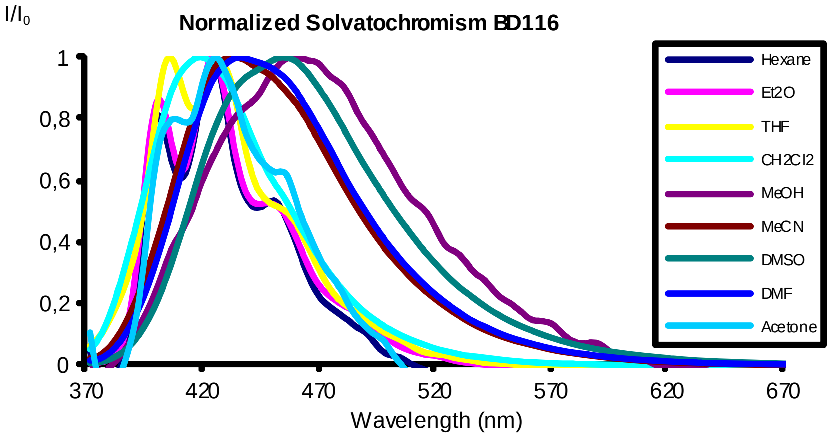

Figure S12: Normalized emission spectra of **BD116** 10^{-4} M in different solvents. $\lambda_{exc} = 366$ nm.

Titrations: Solvent (methanol:water (20:80)), concentration (2.5×10^{-5} M). UV/Vis and fluorescent titrations were carried out in order to obtain stability constants, limits of detection and quantum yields in presence of Hg(II) and MeHg(II). These titrations were carried out by successive additions of each analyte to the cuvette containing the probe until saturation of the signal, usually for concentrations ranging between 2.5×10^{-6} M and 1.25×10^{-3} M.

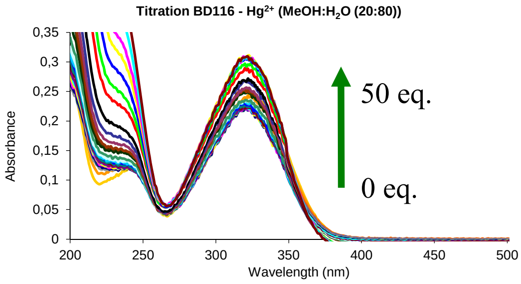

F. S13a

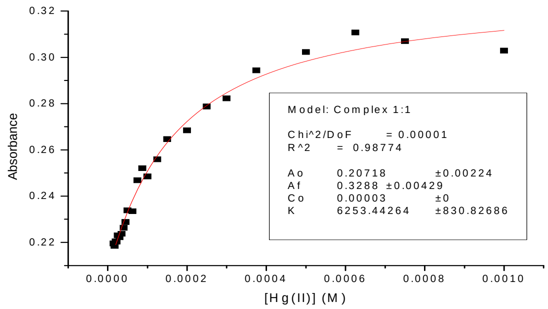

F. S13b

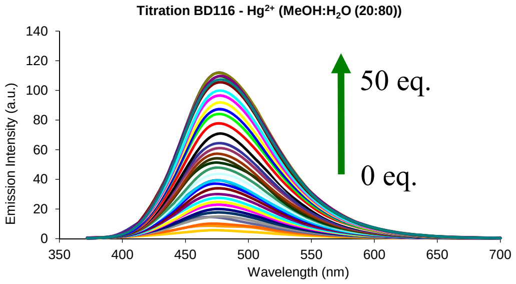

F. S13c

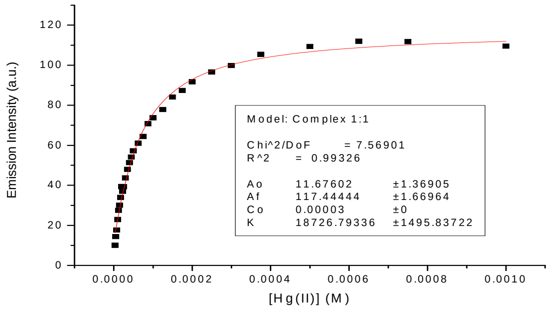

F. S13d

Figures S13a-d: (a) Absorbance spectra, (b) absorbance titration profile (λ_{abs}= 323 nm), (c) emission intensity spectra (λ_{exc}= 350 nm) and (d) emission intensity titration profile ($\lambda_{emission}$= 480 nm) of BD116 2.5 × 10^{-5} M in methanol:water (20:80) with several Hg(II) additions. The caption shows the equivalents of cation for each curve.

Titration experiments were carried out with MeHg(II) using the same mixture of solvents (methanol:water (20:80)) and concentration of the probe (2.5 × 10^{-5} M).

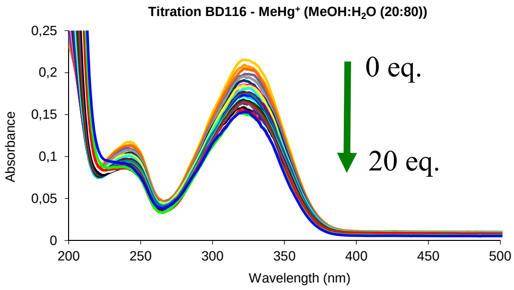

F. S14a

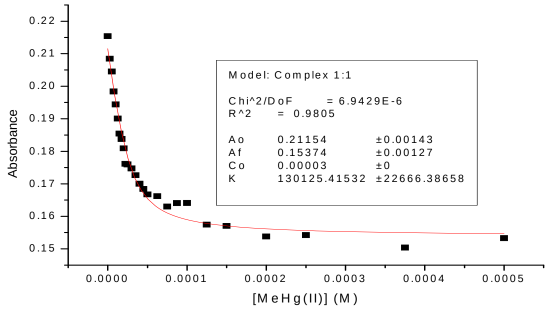

F. S14b

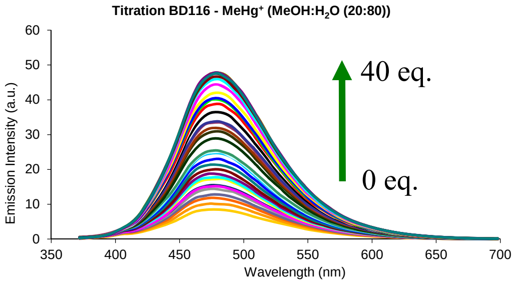

F. S14c

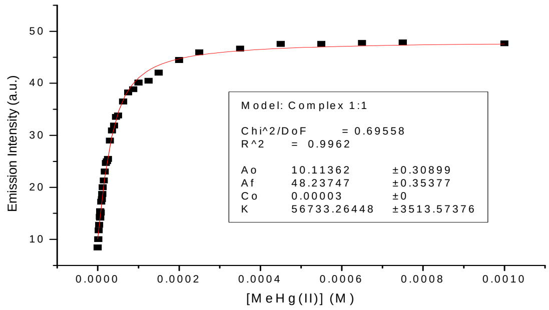

F. S14d

Figures S14a-d: (a) Absorbance spectra, (b) absorbance titration profile (λ_{abs}= 323 nm), (c) emission intensity spectra (λ_{exc}= 350 nm) and (d) emission intensity titration profile ($\lambda_{emission}$= 480 nm) of BD116 2.5 × 10^{-5} M in methanol:water (20:80) with several MeHg(II) additions. The captions show the equivalents of cation for each curve.

SYNTHESIS OF BD119:

Synthesis of tert-butyl 4-(5-(1-(dicyanomethylene)indan-5-yl)pyrimidin-2-yl)piperazine-1-carboxylate:

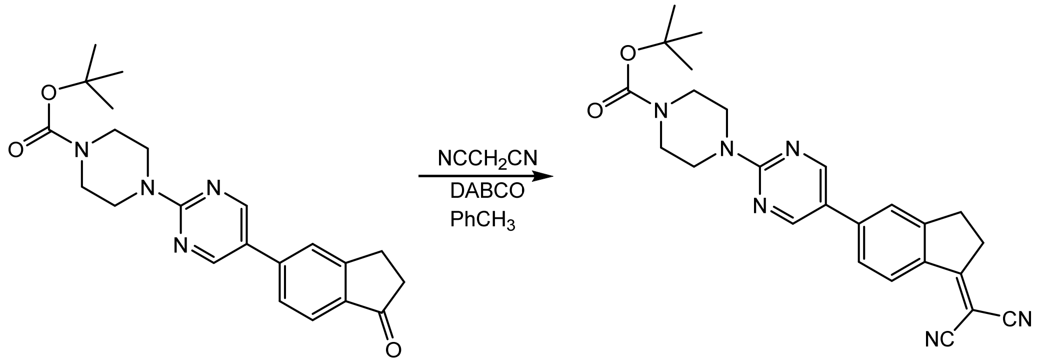

Malononitrile (375 mg, 5.69 mmol) and DABCO (224 mg, 2.01 mmol) were added to a solution of tert-butyl 4-(5-(1-oxoindan-5-yl)pyrimidin-2-yl)piperazine-1-carboxylate (660 mg, 1.67 mmol) in toluene (50 mL) and the mixture was heated under reflux for 4 h. Then the mixture was added to water (100 mL acidified with 5 mL of HCl 1 M) and extracted with CH_2Cl_2 (4 x 40 mL). The combined organic extracts were dried (Na_2SO_4) and the solvent evaporated under reduced pressure. The crude product was purified by flash chromatography (silica, 3 x 30 cm) from CH_2Cl_2 to CH_2Cl_2:MeOH (100:1.5), to get tert-butyl 4-(5-(1-(dicyanomethylene)indan-5-yl)pyrimidin-2-yl)piperazine-1-carboxylate (410 mg, 55%) as a yellow solid, mp: 177-178 ºC (decomp.). IR (KBr, cm^{-1}): 3005, 2971, 2927, 2864, 2217 (C≡N), 1676, 1595, 1562, 1499, 1410, 1362, 1314, 1244, 1158, 1129, 1088, 951, 832, 803, 774. ^{1}H NMR ($CDCl_3$, 300 MHz) δ: 8.62 (s, 2H, H-C=N), 8.43 (d, J = 7.8 Hz, 1H, ArH), 7.57 (s, 1H, ArH), 7.56 (d, J = 7.8 Hz, 1H, ArH), 3.88 (d, J = 6.0 Hz, 4H, 2×CH_2), 3.51 (d, J = 6.0 Hz, 4H, 2×CH_2), 3.31-3.28 (m, 2H, CH_2), 3.24-3.20 (m, 2H, CH_2), 1.48 (s, 9H, 3×CH_3). ^{13}C NMR & DEPT ($CDCl_3$, 75 MHz) δ: 178.3, 161.3, 156.4 (CH_{Ar}), 155.3, 154.9, 142.5, 134.5, 126.9 (CH_{Ar}), 125.4 (CH_{Ar}), 122.3 (CH_{Ar}), 121.1, 113.8 (CN), 113.2 (CN), 80.3, 73.7, 43.8 (CH_2), 34.8 (CH_2), 29.7 (CH_2), 28.5 (CH_3). MS (EI) m/z (%): 442 (M^+, 48), 386 (35), 312 (24), 300 (42), 286 (100), 274 (32). HRMS (EI): calcd. for $C_{25}H_{26}N_6O_2$: 442.2117 (M^+); found: 442.2122.

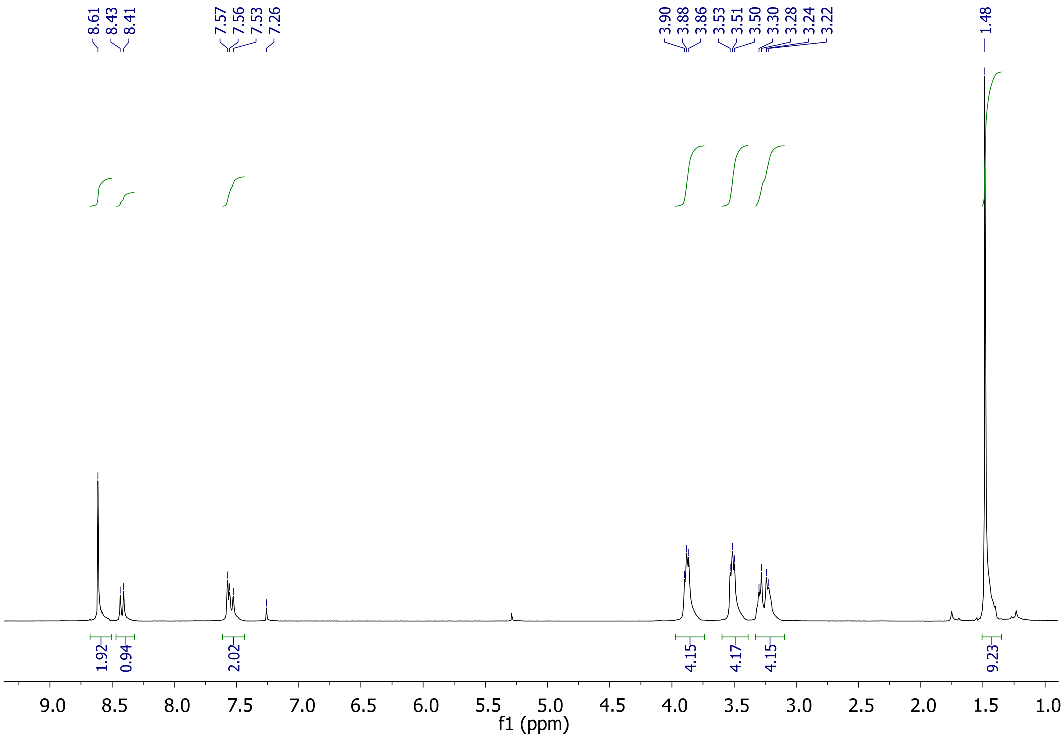

Figure S15: ^{1}H NMR (CDCl$_3$, 300 MHz) of tert-butyl 4-(5-(1-(dicyanomethylene)indan-5-yl)pyrimidin-2-yl)piperazine-1-carboxylate

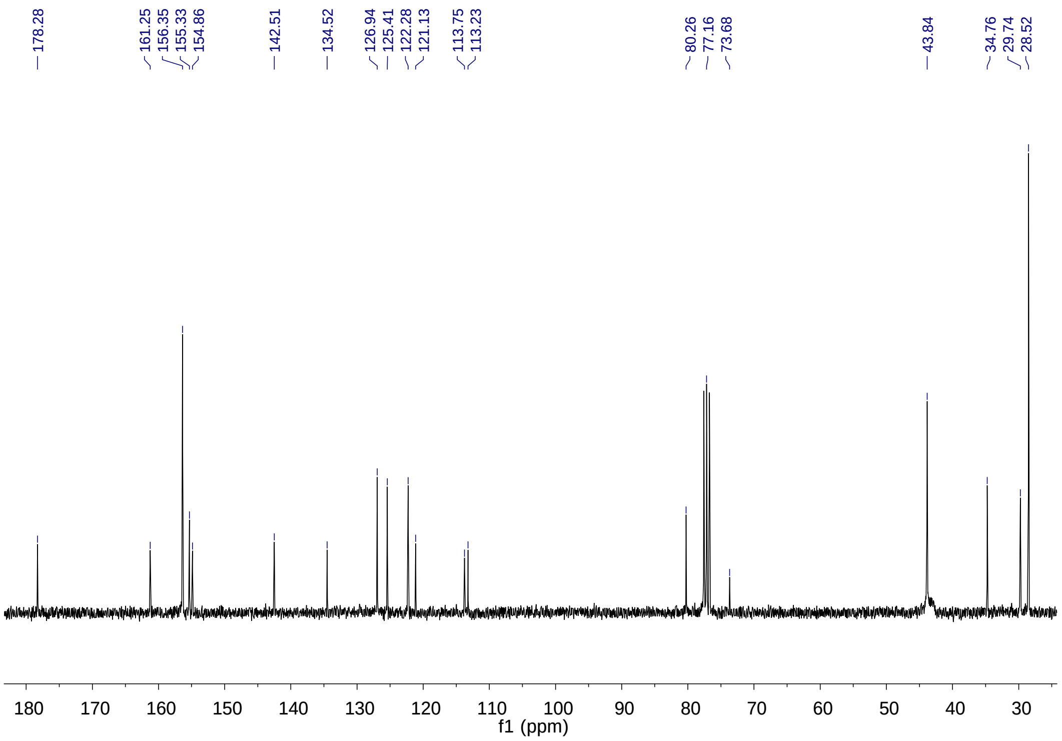

Figure S16: ^{13}C NMR (CDCl$_3$, 75 MHz) of tert-butyl 4-(5-(1-(dicyanomethylene)indan-5-yl)pyrimidin-2-yl)piperazine-1-carboxylate

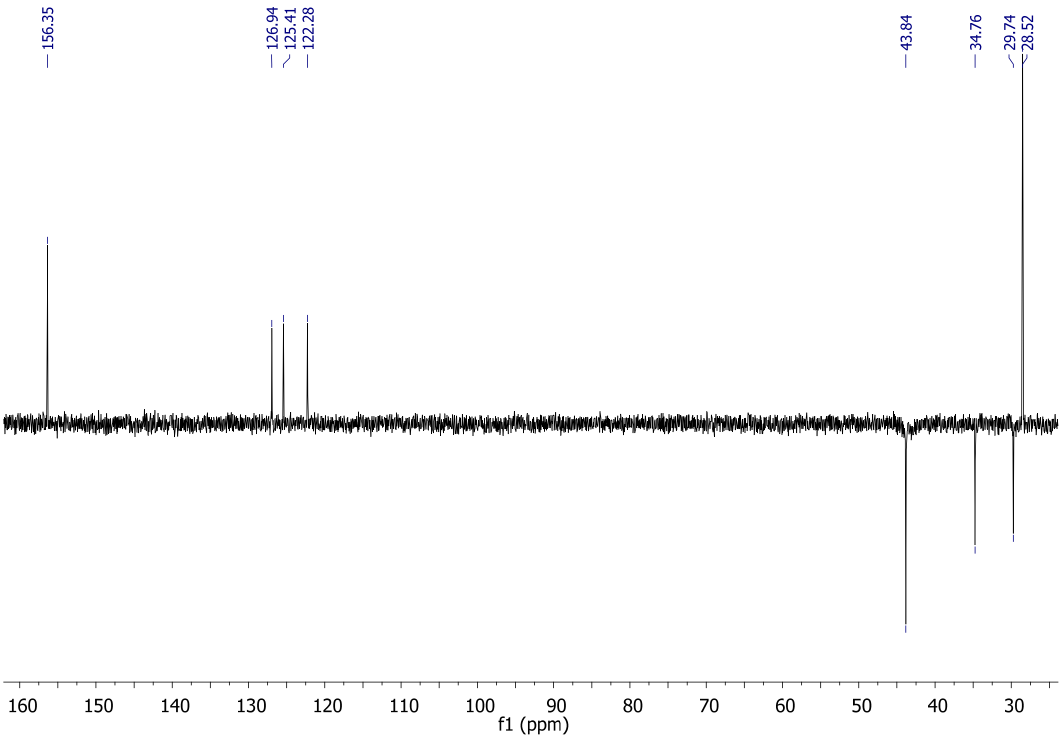

Figure S17: DEPT (CDCl$_3$, 75 MHz) of tert-butyl 4-(5-(1-(dicyanomethylene)indan-5-yl)pyrimidin-2-yl)piperazine-1-carboxylate

Synthesis of 1-dicyanomethylene-5-[(2-piperazin-1-yl)pyrimidin-5-yl]indane 1c:

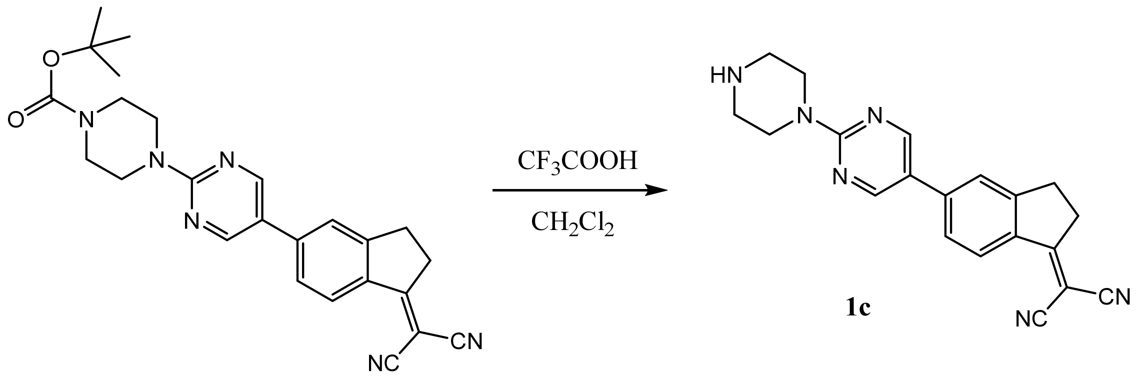

Trifluoroacetic acid (5 mL, ρ = 1.489 g/mL, 65.30 mmol) was added dropwise with stirring to a solution of tert-butyl 4-(5-(1-(dicyanomethylene)-indan-5-yl)pyrimidin-2-yl)piperazine-1-carboxylate (415 mg, 0.94 mmol) in CH_2Cl_2 (10 mL) and the resulting mixture was stirred at room temperature for 15 minutes. Then the mixture was added to water (50 mL), basified to pH = 10 with 5% aqueous NaOH and extracted with CH_2Cl_2 (5 x 40 mL). The combined organic extracts were dried (Na_2SO_4), the solvent evaporated under reduced pressure and the residue was recrystallized from CH_2Cl_2-MeOH (5:1) to get 2-(5-(2-(piperazin-1-yl)pyrimidin-5-yl)-indan-1-ylidene)malononitrile **1c** (315 mg, 98%) as a yellow solid, mp: 214-215 ºC (decomp.). IR (KBr, cm^{-1}): 3434 (N-H), 3027, 2966, 2853, 2227 (C≡N), 1670, 1594, 1568, 1521, 1455, 1362, 1322, 1276, 1204, 1168, 1132, 822, 799, 723. ^{1}H NMR (DMSO-d_6, 300 MHz) δ: 8.95 (s, 2H, H-C=N), 8.25 (d, J = 8.5 Hz, 1H, ArH), 7.99 (s, 1H, ArH), 7.92 (d, J = 8.5 Hz, 1H, ArH), 4.04 (s, 4H, 2×CH_2), 3.41 (s, 1H, NH), 3.32-3.30 (m, 2H, CH_2), 3.21 (s, 6H, 3×CH_2). ^{13}C NMR & DEPT (DMSO-d_6, 75 MHz) δ: 180.0, 160.4, 156.7 (CH_{Ar}), 156.3, 141.4, 134.1, 125.5 (CH_{Ar}), 125.2 (CH_{Ar}), 122.5 (CH_{Ar}), 121.1, 114.0 (CN), 113.6 (CN), 71.7, 42.5 (CH_2), 40.5 (CH_2), 34.7 (CH_2), 29.6 (CH_2). MS (EI) m/z (%): 342 (M^+, 20), 300 (100), 287 (26), 274 (42). HRMS (EI): calcd. for $C_{20}H_{18}N_6$: 342.1593 (M^+); found: 342.1609.

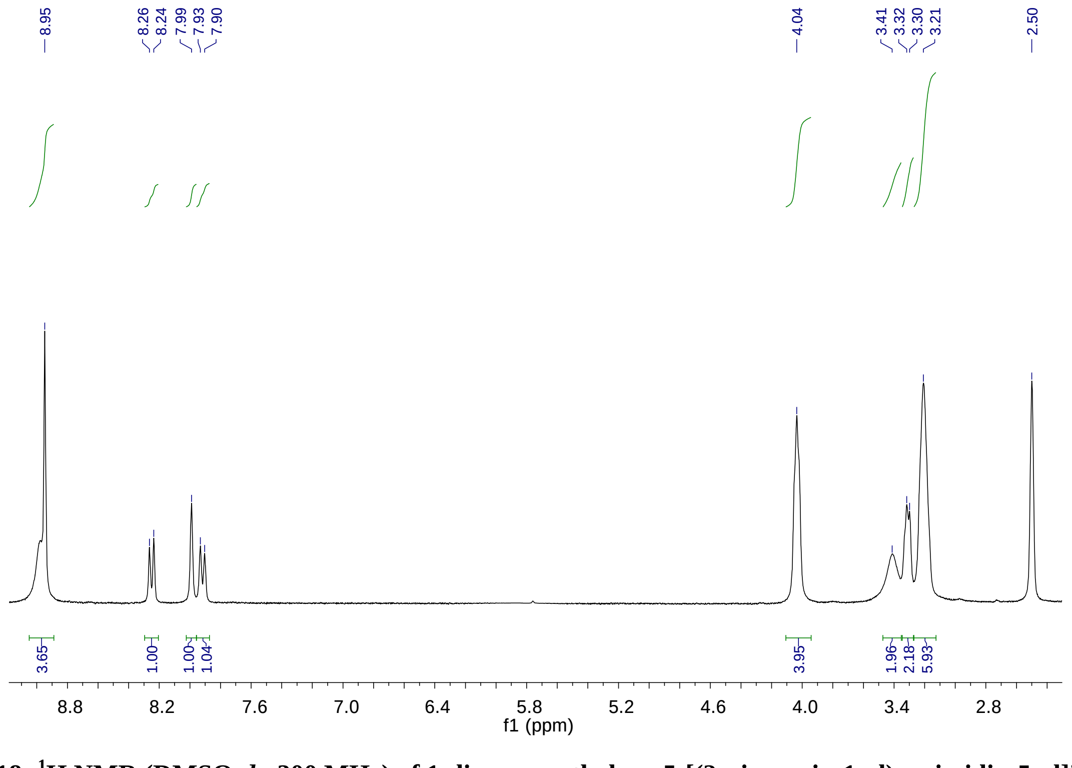

Figure S18: ^{1}H NMR (DMSO-d_6, 300 MHz) of 1-dicyanomethylene-5-[(2-piperazin-1-yl)pyrimidin-5-yl]indane 1c

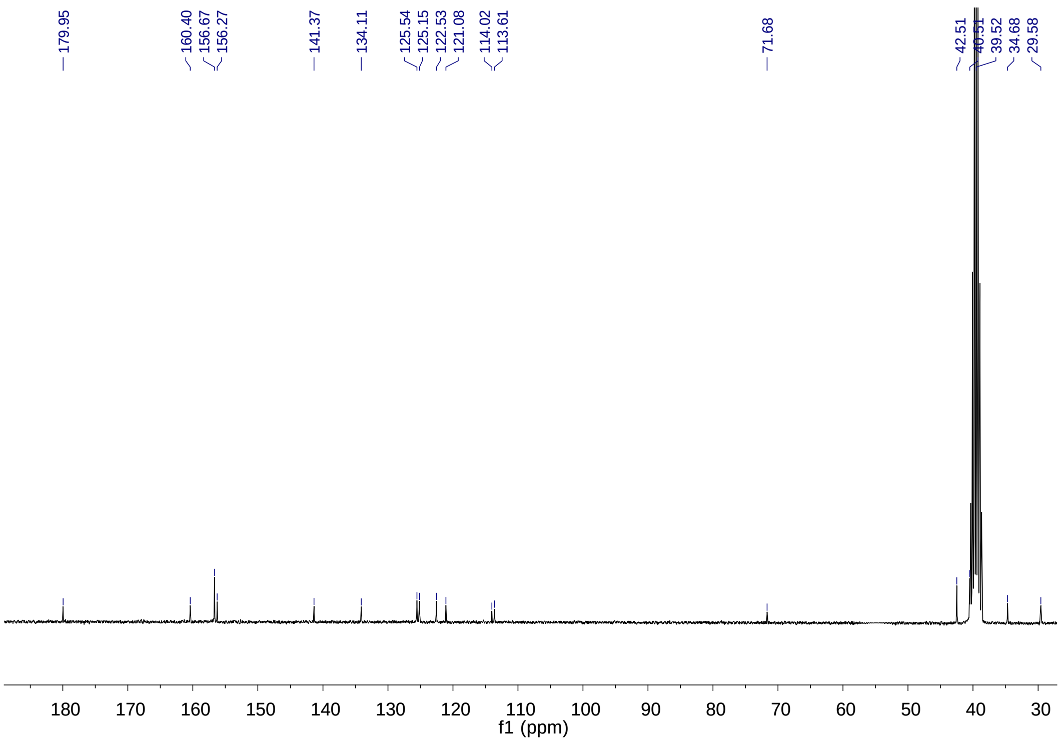

Figure S19: ^{13}C NMR (DMSO-d_6, 75 MHz) of 1-dicyanomethylene-5-[(2-piperazin-1-yl)pyrimidin-5-yl]indane 1c

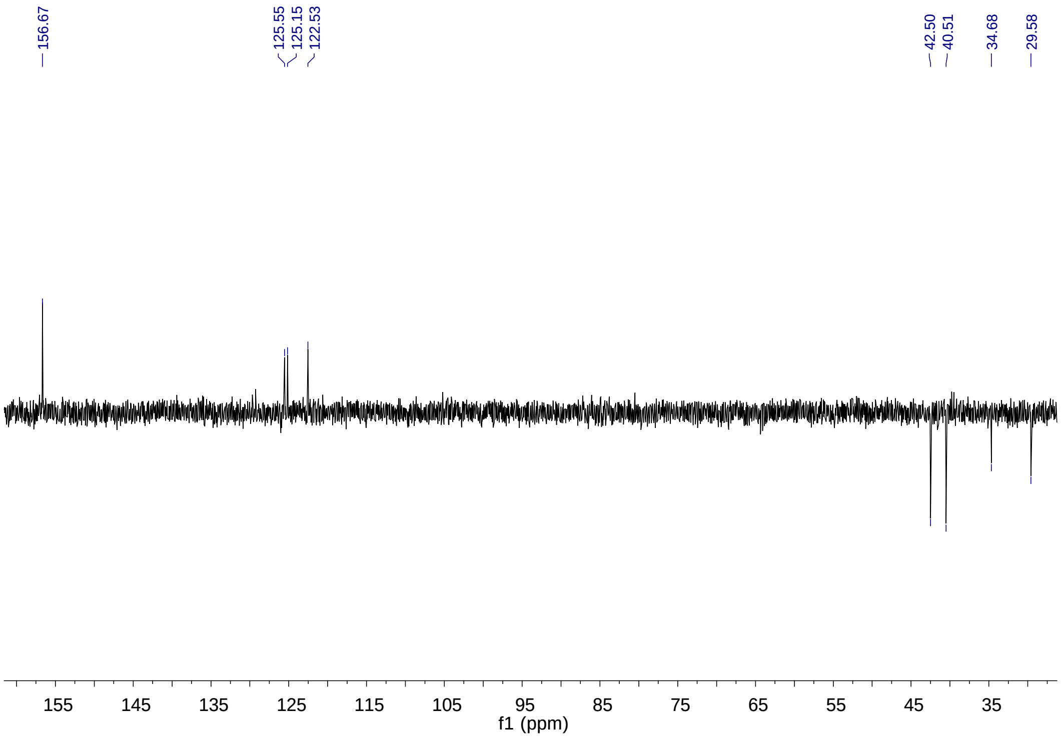

Figure S20: DEPT (DMSO-d_6, 75 MHz) of 1-dicyanomethylene-5-[(2-piperazin-1-yl)pyrimidin-5-yl]indane 1c

Synthesis of 2-(5-(2-(4-(dimethylphosphorothioyl)piperazin-1-yl)pyrimidin-5-yl)-indan-1-ylidene)malononitrile BD119:

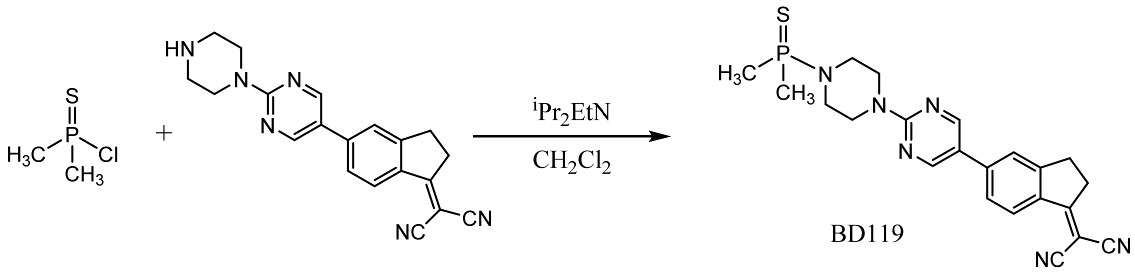

Dimethylthiophosphinic chloride (13.9 µL, ρ = 1.230 g/mL, 0.13 mmol) was added to a solution of 2-(5-(2-(piperazin-1-yl)pyrimidin-5-yl)-indan-1-ylidene)malononitrile **1c** (50 mg, 0.15 mmol) in CH_2Cl_2 (20 mL) in an ice bath. Then, *N*,*N*-diisopropylethylamine (25.4 µL, ρ = 0.742 g/mL, 0.15 mmol) was added to the mixture and the resulting mixture was stirred for 15 minutes at 0 ºC, then for 20 minutes at room temperature and then for 5 hours at 35 ºC. Then the solvent was evaporated under reduced pressure, the residue washed up with hexane and the crude product was purified by flash chromatography (silica, 1 × 20 cm) from CH_2Cl_2 to CH_2Cl_2:AcOEt (10:1), to get 2-(5-(2-(4-(dimethylphosphorothioyl)piperazin-1-yl)pyrimidin-5-yl)-indan-1-ylidene)malononitrile BD119 (50 mg, 79 %) as a yellow solid. mp: >230 ºC. IR (KBr, cm^{-1}): 2911, 2852, 2215 (C≡N), 1590, 1556, 1514, 1447, 1362, 1312, 1261, 1130, 966, 915. ^{1}H NMR ($CDCl_3$, 300 MHz) δ: 8.61 (s, 2H, H-C=N), 8.42 (d, *J* = 8.4 Hz, 1H, ArH), 7.55 (m, 2H), 3.94 (t, *J* = 5.0 Hz, 4H, 2×CH_2), 3.31-3.20 (m, 4H, 2×CH_2), 3.12 (m, 4H, 2×CH_2), 1.87 (s, 3H, P-CH_3), 1.83 (s, 3H, P-CH_3). ^{13}C NMR (DMSO-d_6, 75 MHz) δ: 178.3, 161.2, 156.4 (2×CH_{Ar}), 155.3, 142.5, 134.6, 127.0 (CH_{Ar}), 125.5 (CH_{Ar}), 122.4 (CH_{Ar}), 121.3, 113.8 (C≡N), 113.2 (C≡N), 73.7, 44.4 (CH_2), 44.2 (CH_2), 44.1 (CH_2), 34.8 (CH_2), 29.8 (CH_2), 21.9 (CH_3), 21.0 (CH_3). MS (EI) m/z (%): 434 (M^+, 36), 401 (17), 341 (21), 312 (71), 300 (28), 286 (21), 122 (100), 93 (78). HRMS (EI): calcd. for $C_{22}H_{23}N_6PS$: 434.1443 (M^+); found: 434.1444.

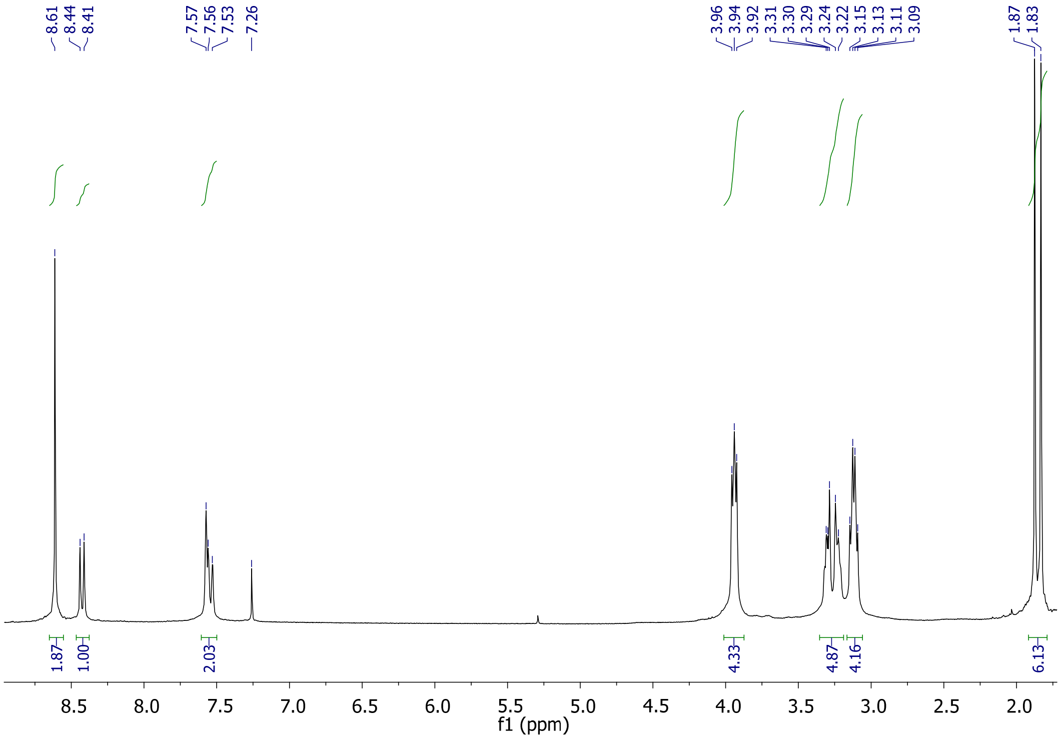

Figure S21: ^{1}H NMR ($CDCl_3$, 300 MHz) of 2-(5-(2-(4-(dimethylphosphorothioyl)piperazin-1-yl)pyrimidin-5-yl)-indan-1-ylidene)malononitrile BD119

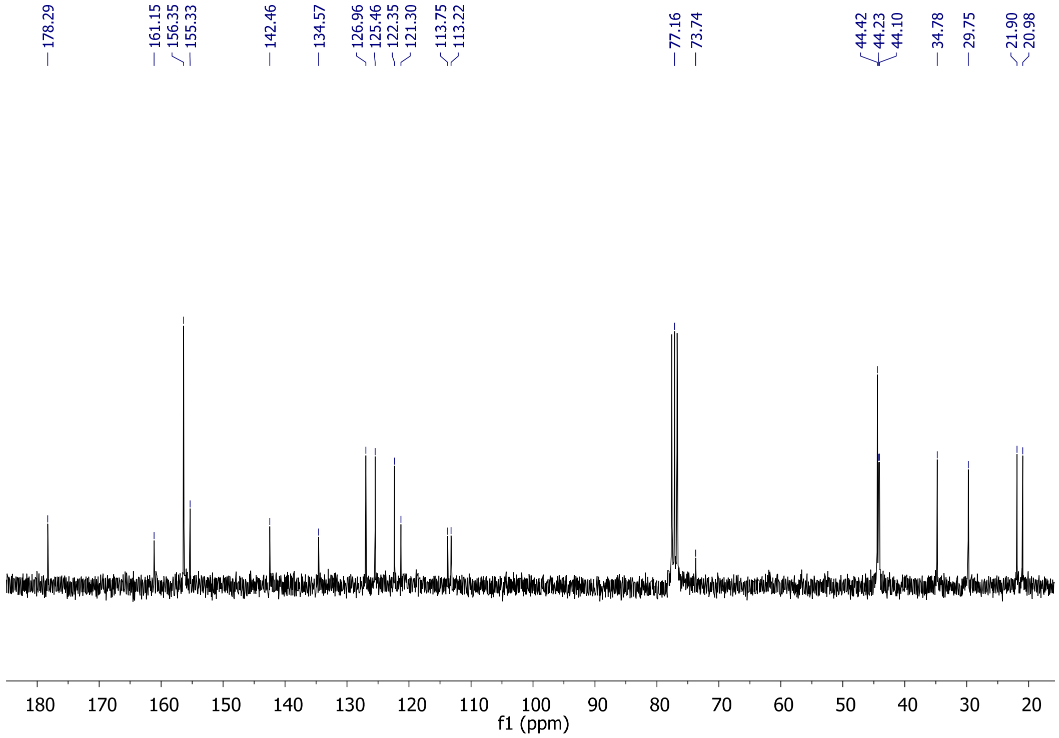

Figure S22: ^{13}C NMR ($CDCl_3$, 75 MHz) of 2-(5-(2-(4-(dimethylphosphorothioyl)piperazin-1-yl)pyrimidin-5-yl)-indan-1-ylidene)malononitrile BD119

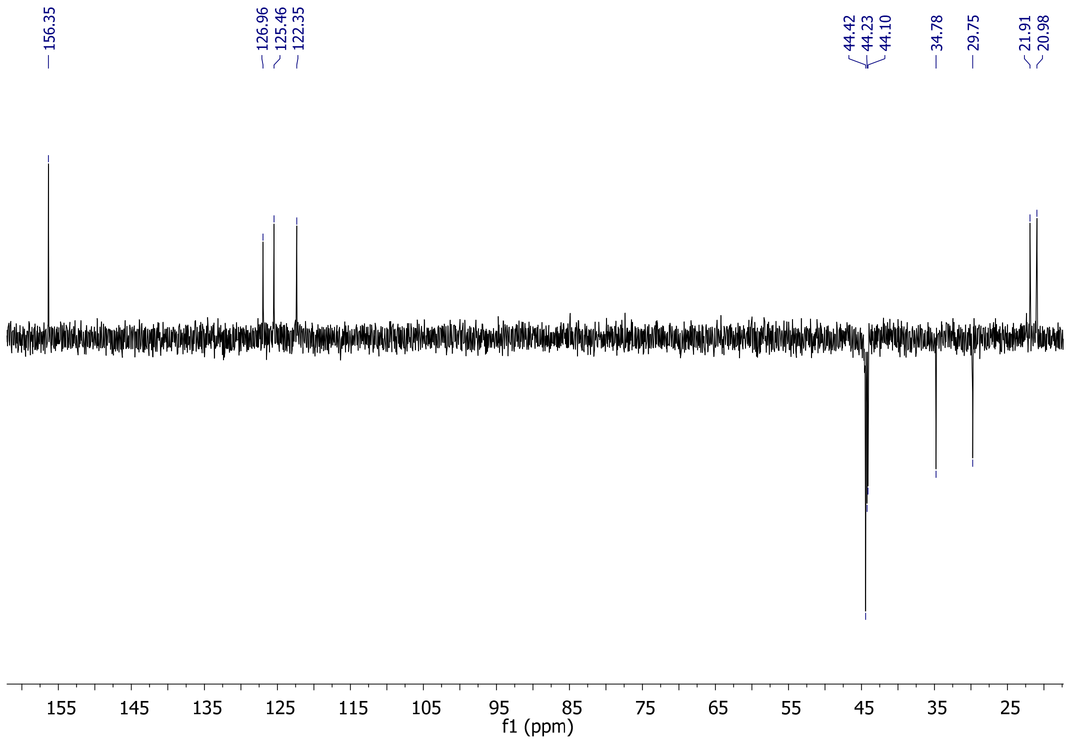

Figure S23: DEPT ($CDCl_3$, 75 MHz) of 2-(5-(2-(4-(dimethylphosphorothioyl)piperazin-1-yl)pyrimidin-5-yl)-indan-1-ylidene)malononitrile BD119

Solvatochromism

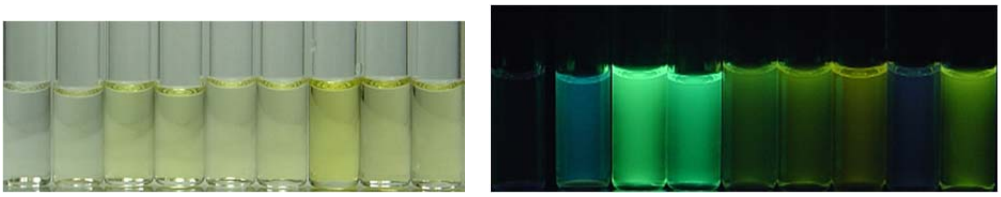

(1) (2) (3) (4) (5) (6) (7) (8) (9) (1) (2) (3) (4) (5) (6) (7) (8) (9)
(a) (b)

Figure S24: BD119 10^{-4} M in (1) Hexane, (2) Et_2O, (3) THF, (4) CH_2Cl_2, (5) MeOH, (6) MeCN, (7) DMSO, (8) DMF or (9) Acetone under (a) direct sunlight and (b) 366 nm light.

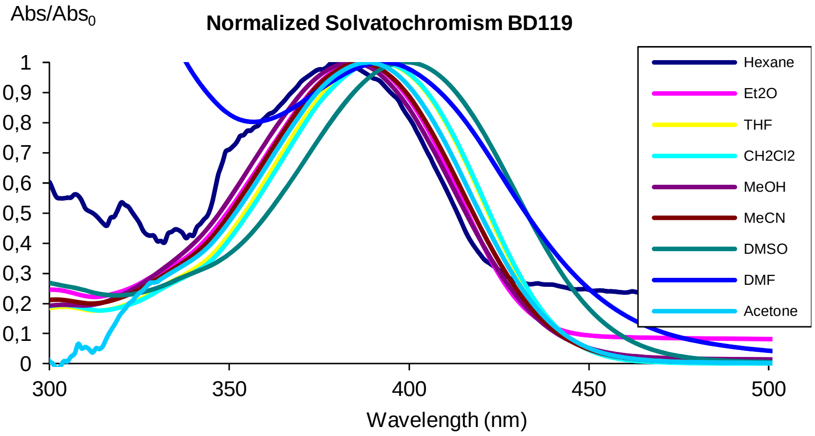

Figure S25: Normalized absorption spectra of **BD119** 10^{-4} M in different solvents.

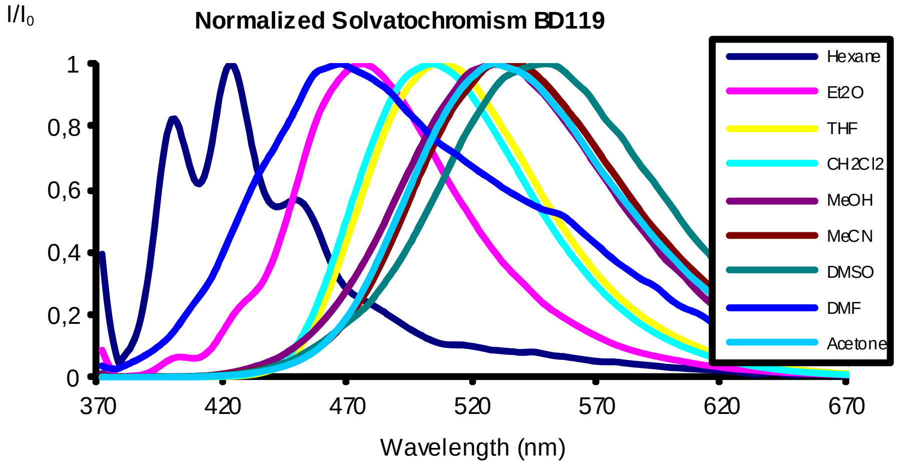

Figure S26: Normalized emission spectra of **BD119** 10^{-4} M in different solvents. $\lambda_{exc} = 366$ nm.

Titrations: Solvent (methanol:water (20:80)) and concentration (2.5×10^{-5} M). UV/Vis and fluorescent titrations were carried out in order to obtain stability constants, limits of detection and quantum yields in presence of Hg(II) and MeHg(II). These titrations were carried out by successive additions of each analyte to the cuvette containing the probe until saturation of the signal, usually for concentrations ranging between 2.5×10^{-6} M and 1.25×10^{-3} M.

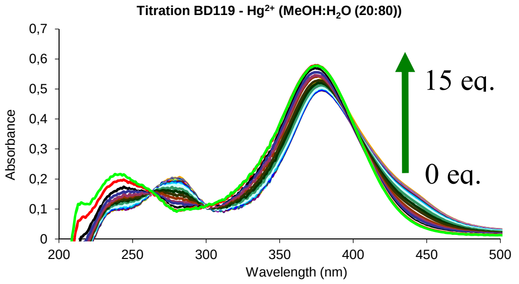

F. S27a

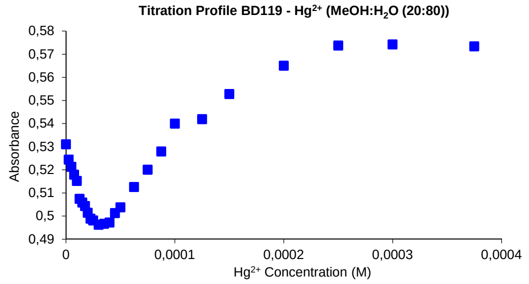

F. S27b

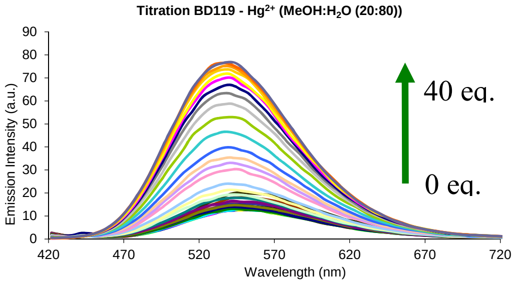

F. S27c

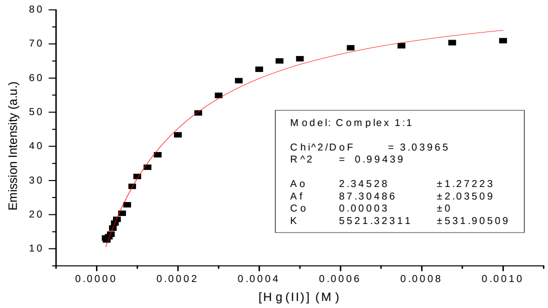

F. S27d

Figures S27a-d: (a) Absorbance spectra, (b) absorbance titration profile (λ_{abs}= 378 nm), (c) emission intensity spectra (λ_{exc}= 398 nm) and (d) emission intensity titration profile ($\lambda_{emission}$= 554 nm) of BD119 2.5×10^{-5} M in methanol:water (20:80) with several Hg(II) additions. The caption shows the equivalents of cation for each curve.

SYNTHESIS OF BD118:

Synthesis of tert-butyl 4-(5-(1-oxoindan-5-yl)pyridin-2-yl)piperazine-1-carboxylate:

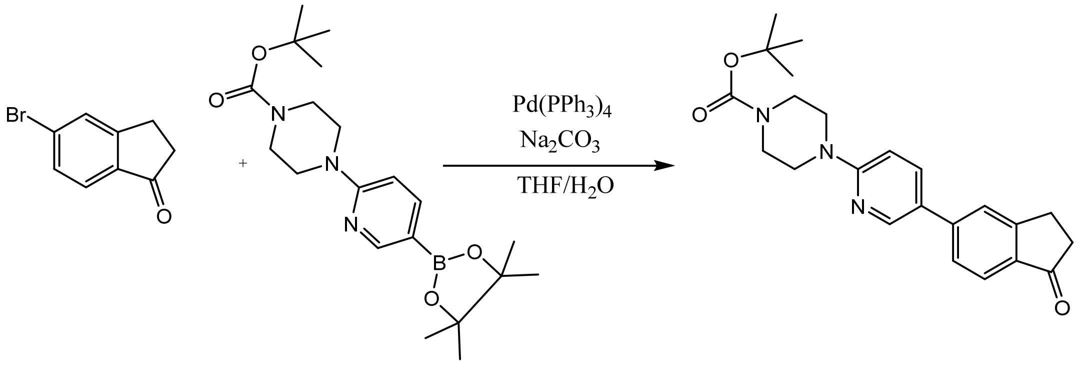

5-Bromoindanone (800 mg, 3.81 mmol), 6-(4-Boc-piperazin-1-yl)pyridine-3-boronic acid pinacol ester (1600 g, 4.11 mmol) and Na_2CO_3 (3423 g, 32.4 mmol) were dissolved in a mixture of THF:H_2O (10:1, 66 mL) under stirring in a nitrogen atmosphere, then $Pd(PPh_3)_4$ (5 mg) was added and the resulting mixture was heated overnight under reflux. Then the mixture was added to water (100 mL) and extracted with CH_2Cl_2 (4 x 40 mL). The combined organic extracts were dried (Na_2SO_4) and the solvent was evaporated under reduced pressure. The residue was purified by flash chromatography (silica, 3 x 30 cm), from CH_2Cl_2 to CH_2Cl_2:AcOEt (5:1), to get tert-butyl 4-(5-(1-oxoindan-5-yl)pyridin-2-yl)piperazine-1-carboxylate (1270 mg, 85%) as a white solid, mp: 190-191 ºC (decomp.). IR (KBr, cm^{-1}): 2976, 2930, 2853, 1696 (C=O), 1675, 1595, 1511, 1429, 1310, 1286, 1239, 1168, 1055, 999, 934, 810. ^{1}H NMR ($CDCl_3$, 300 MHz) δ: 8.45 (d, J = 2.5 Hz, 1H, H-C=N), 7.74 (d, J = 8.0 Hz, 1H, ArH), 7.73 (dd, J_1 = 8.9 Hz, J_2 = 2.5 Hz, 1H, ArH), 7.55 (s, 1H, ArH), 7.48 (d, J = 8.0 Hz, 1H, ArH), 6.69 (d, J = 8.9 Hz, 1H, ArH), 3.59-3.53 (m, 8H, 4×CH_2), 3.13 (t, J = 6.0 Hz, 2H, CH_2), 2.68 (t, J = 6.0 Hz, 2H, CH_2), 1.46 (s, 9H, 3×CH_3). ^{13}C NMR & DEPT ($CDCl_3$, 75 MHz) δ: 206.3 (C=O), 158.7, 156.1, 154.7, 146.6 (CH_{Ar}), 144.6, 136.3 (CH_{Ar}), 135.4, 125.5 (CH_{Ar}), 125.4, 124.2 (CH_{Ar}), 123.6 (CH_{Ar}), 106.8 (CH_{Ar}), 80.0, 44.9 (CH_2), 43.1 (CH_2), 36.4 (CH_2), 28.4 (CH_3), 25.8 (CH_2). MS (EI) m/z (%): 393 (M^+, 29), 263 (14), 251 (17), 237 (100), 224 (18). HRMS (EI): calcd. for $C_{23}H_{27}N_3O_3$: 393.2052 (M^+); found: 393.2040.

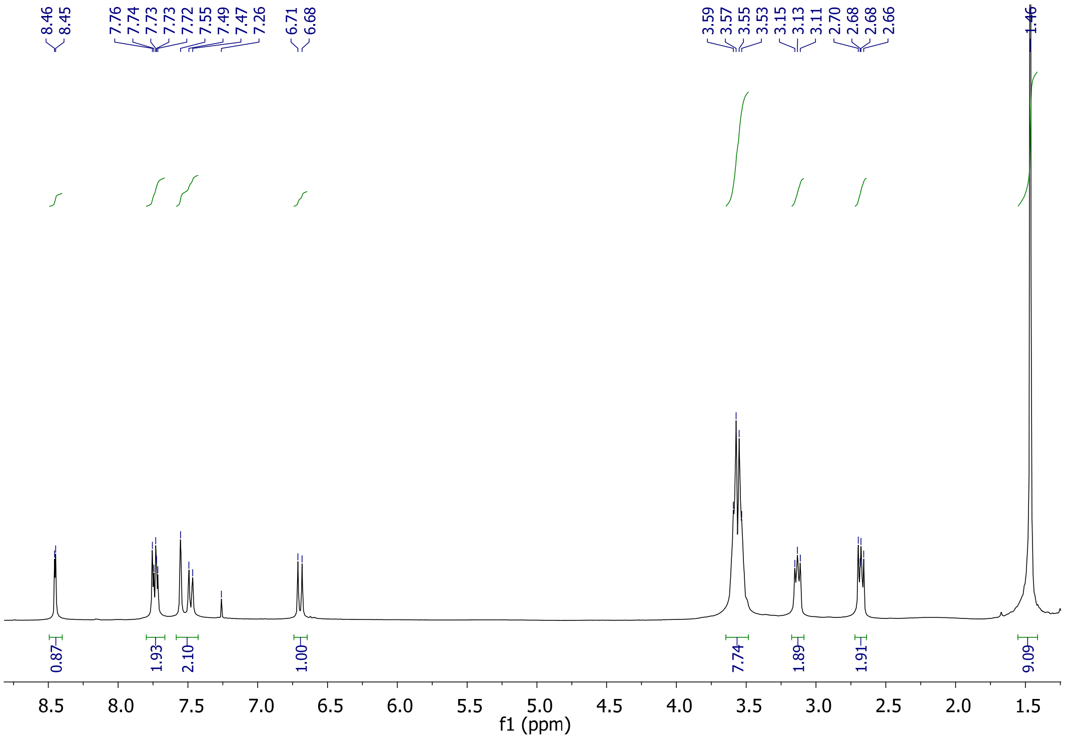

Figure S28: ^{1}H NMR ($CDCl_3$, 300 MHz) of tert-butyl 4-(5-(1-oxoindan-5-yl)pyridin-2-yl)piperazine-1-carboxylate

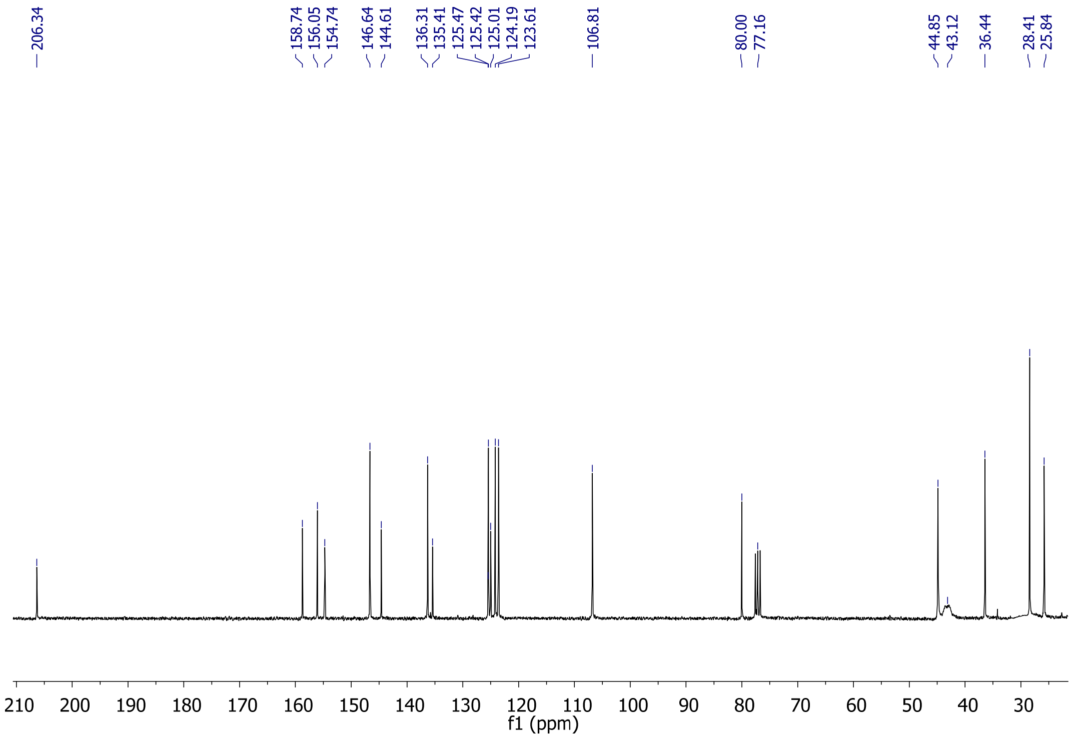

Figure S29: ^{13}C NMR ($CDCl_3$, 75 MHz) of tert-butyl 4-(5-(1-oxoindan-5-yl)pyridin-2-yl)piperazine-1-carboxylate

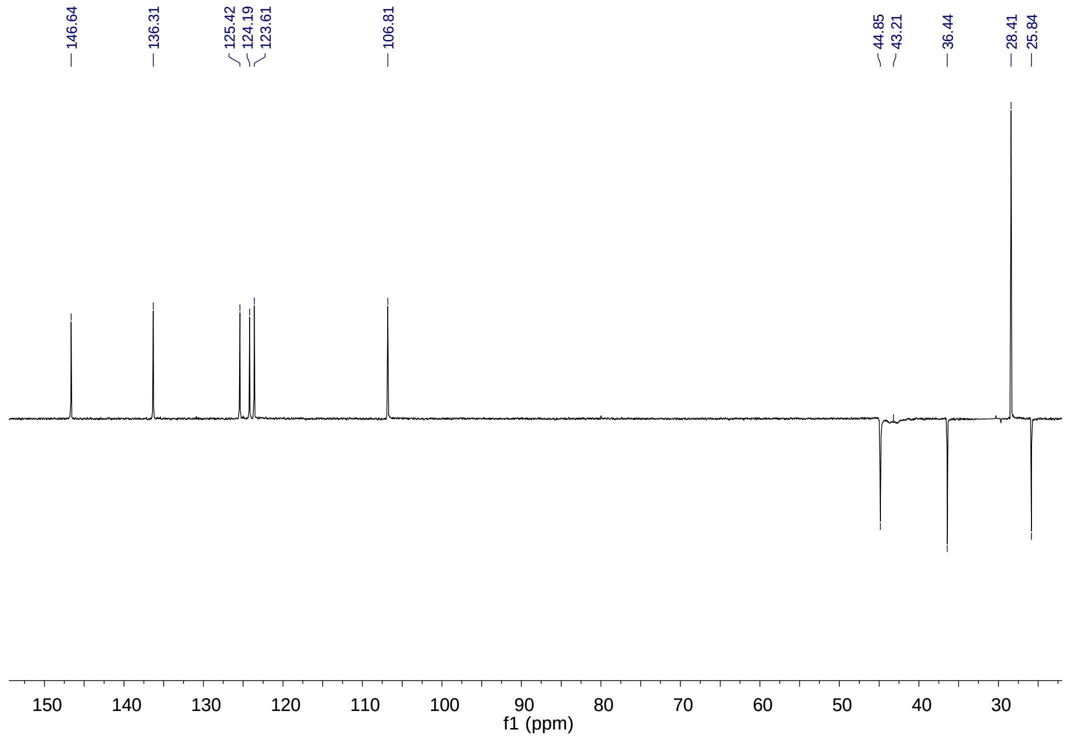

FigureS30: DEPT ($CDCl_3$, 75 MHz) of tert-butyl 4-(5-(1-oxoindan-5-yl)pyridin-2-yl)piperazine-1-carboxylate

Synthesis of 5-(6-(piperazin-1-yl)pyridin-3-yl)indan-1-one 1b:

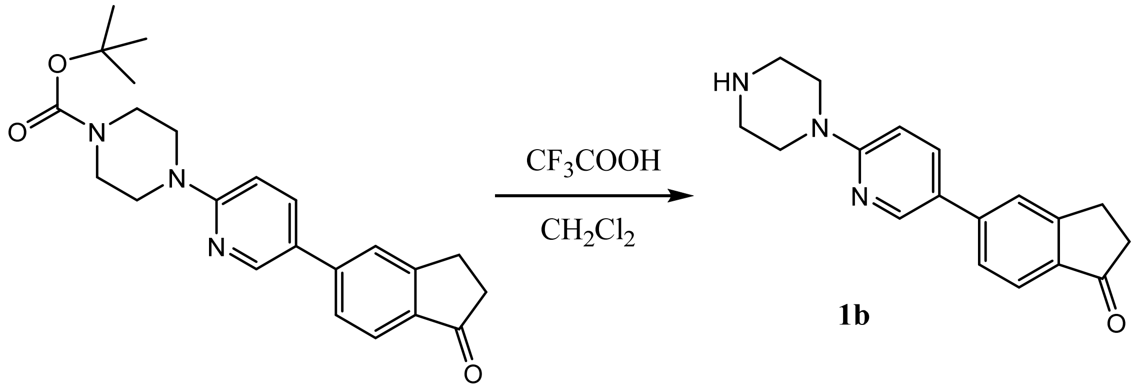

Trifluoroacetic acid (10 mL, ρ= 1.489 g/mL, 130.59 mmol) was added dropwise under stirring to a solution of tert-butyl 4-(5-(1-oxoindan-5-yl)pyridin-2-yl)piperazine-1-carboxylate (800 mg, 2.03 mmol) in CH_2Cl_2 (20 mL) and the resulting mixture was stirred at room temperature for 15 minutes. Then the mixture was added to water (50 mL), basified to pH= 10 with 5% NaOH and extracted with CH_2Cl_2 (4 x 50 mL). The combined organic extracts were dried (Na_2SO_4), the solvent was evaporated under reduced pressure and the residue was recrystallized from CH_2Cl_2-MeOH (5:1) to get 5-(6-(piperazin-1-yl)pyridin-3-yl)-indan-1-one **1b** (590 mg, 99%) as a yellow solid, mp: 163-164 ºC (decomp.). IR (KBr, cm^{-1}): 3316 (N-H), 2949, 2827, 1695 (C=O), 1602, 1506, 1306, 1277, 1251, 1240, 1103, 948, 859, 807. ^{1}H NMR ($CDCl_3$, 300 MHz) δ: 8.46 (d, J = 2.5 Hz, 1H, H-C=N), 7.75 (d, J = 8.0 Hz, 1H, ArH), 7.73 (dd, J_1 = 8.9 Hz, J_2 = 2.5 Hz, 1H, ArH), 7.56 (s, 1H, ArH), 7.49 (d, J = 8.0 Hz, 1H, ArH), 6.69 (d, J = 8.9 Hz, 1H, ArH), 3.57 (t, J = 5.0 Hz, 4H, 2×CH_2), 3.16 (t, J = 6.0 Hz, 2H, CH_2), 2.98 (t, J = 5.0 Hz, 4H, 2×CH_2), 2.69 (t, J = 6.0 Hz, 2H, CH_2), 1.93 (s, 1H, NH). ^{13}C NMR & DEPT ($CDCl_3$, 75 MHz) δ: 206.5 (C=O), 159.3, 156.2, 146.7 (CH_{Ar}), 144.9, 136.3 (CH_{Ar}), 135.4, 125.5 (CH_{Ar}), 124.7, 124.3 (CH_{Ar}), 123.6 (CH_{Ar}), 106.7 (CH_{Ar}), 46.2 (CH_2), 46.0 (CH_2), 36.6 (CH_2), 25.9 (CH_2). MS (EI) m/z (%): 293 (M^+, 42), 263 (24), 251 (100), 237 (86), 225 (81). HRMS (EI): calcd. for $C_{18}H_{19}N_3O$: 293.1528 (M^+); found: 293.1523.

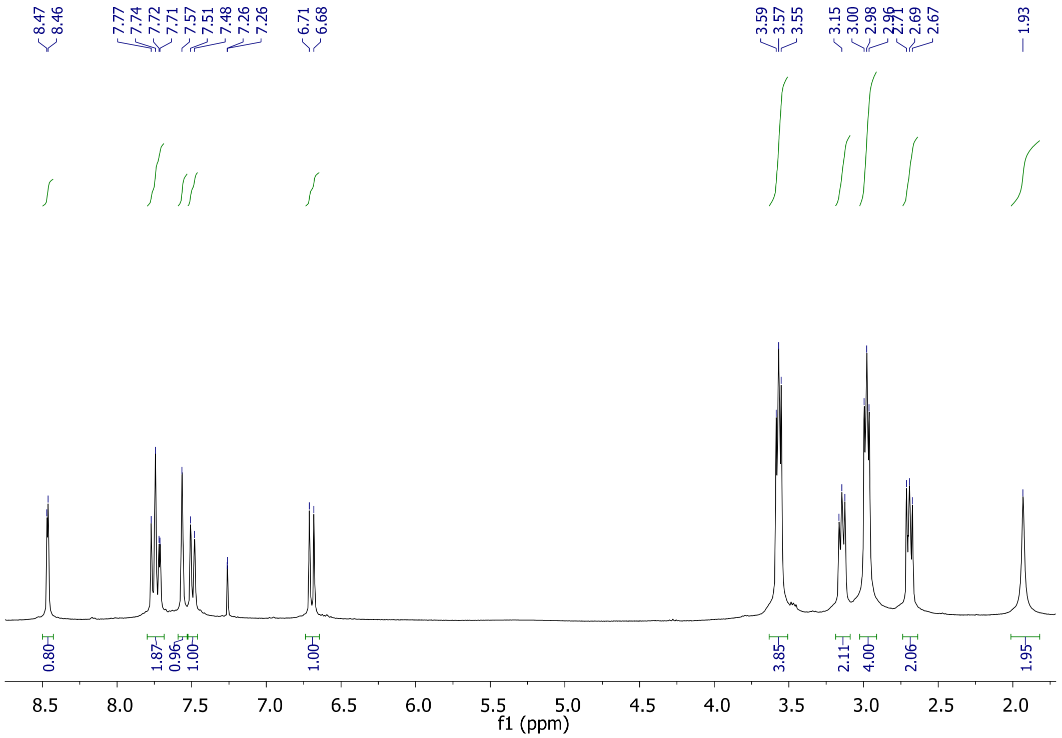

Figure S31: ^{1}H NMR ($CDCl_3$, 300 MHz) of 5-(6-(piperazin-1-yl)pyridin-3-yl)indan-1-one 1b

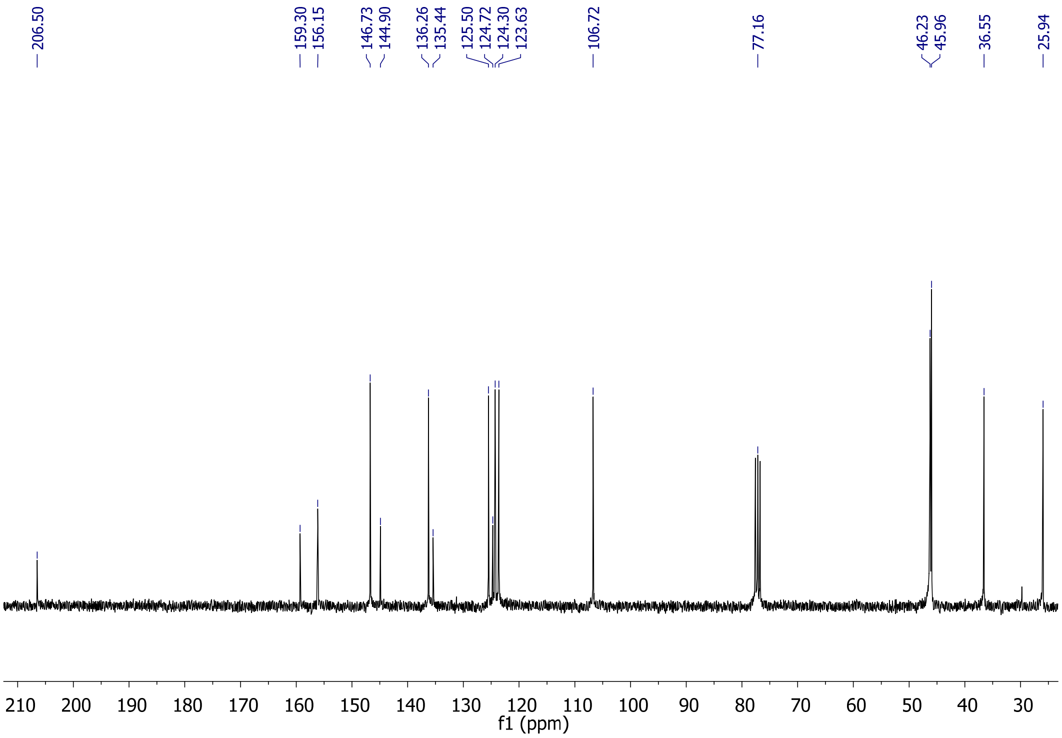

Figure S32: ^{13}C NMR ($CDCl_3$, 75 MHz) of 5-(6-(piperazin-1-yl)pyridin-3-yl)indan-1-one 1b

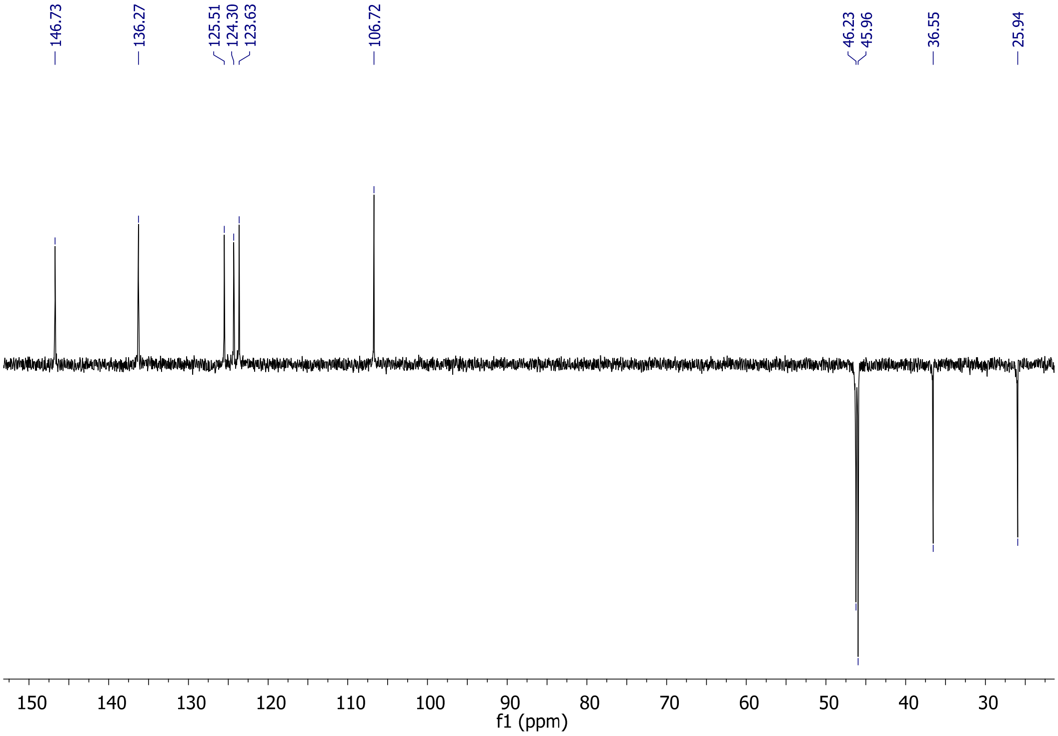

Figure S33: DEPT ($CDCl_3$, 75 MHz) of 5-(6-(piperazin-1-yl)pyridin-3-yl)indan-1-one 1b

Synthesis of 5-(6-(4-(dimethylphosphorothioyl)piperazin-1-yl)pyridin-3-yl)-indan-1-one BD118:

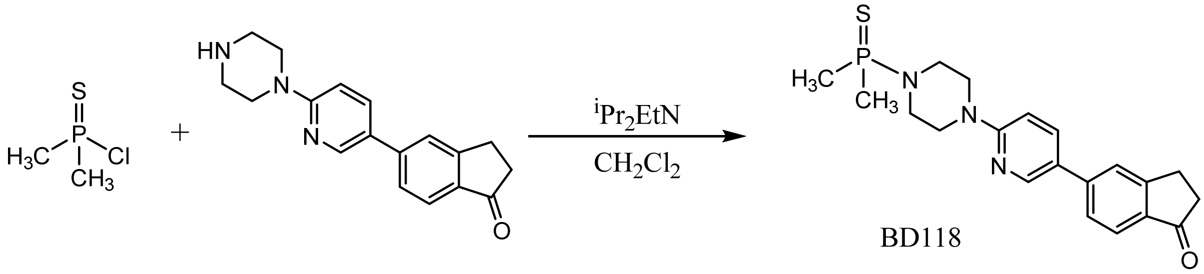

Dimethylthiophosphinic chloride (16.2 µL, ρ = 1.230 g/mL, 0.15 mmol) was added to a solution of 5-(6-(piperazin-1-yl)pyridin-3-yl)-indan-1-one **1b** (50 mg, 0.17 mmol) in CH_2Cl_2 (20 mL) in an ice bath. Then, *N*,*N*-diisopropylethylamine (29.6 µL, ρ = 0.742 g/mL, 0.17 mmol) was added to the mixture and the resulting mixture was stirred for 15 minutes at 0 ºC, then for 20 minutes at room temperature and then for 4 hours at 35 ºC. Then the solvent was evaporated under reduced pressure, the residue was washed up with hexane and the crude product was purified by flash chromatography (silica, 1 × 20 cm) from CH_2Cl_2:AcOEt (40:1) to CH_2Cl_2:MeOH (100:1.5), to get 5-(6-(4-(dimethylphosphorothioyl)piperazin-1-yl)pyridin-3-yl)-indan-1-one BD118 (48 mg, 73%) as a pale yellow solid. mp: 191-192 ºC (decomp.). IR (KBr, cm^{-1}): 3012, 2979, 2907, 2831 (C-H), 1691 (C=O), 1586, 1502, 1442, 1371, 1307, 1231, 1130, 1109, 953, 911, 809, 712. ^{1}H NMR ($CDCl_3$, 300 MHz) δ: 8.46 (d, *J* = 2.0 Hz, 1H, H-C=N), 7.76 (d, *J* = 8.1 Hz, 1H, ArH), 7.74 (m, 1H, ArH), 7.57 (s, 1H, ArH), 7.49 (d, *J* = 8.1 Hz, 1H, ArH), 6.72 (d, *J* = 8.9 Hz, 1H, ArH), 3.63 (t, *J* = 5.0 Hz, 4H, 2×CH_2), 3.18-3.10 (m, 6H, 3×CH_2), 2.70 (t, *J* = 6.0 Hz, 2H, CH_2), 1.85 (s, 3H, P-CH_3), 1.81 (s, 3H, PCH_3). ^{13}C NMR ($CDCl_3$, 75 MHz) δ: 206.4 (C=O), 158.6, 156.0, 146.5 (CH_{Ar}), 144.5, 136.3 (CH_{Ar}), 135.4, 125.4 (CH_{Ar}), 125.2, 124.2 (CH_{Ar}), 123.6 (CH_{Ar}), 106.8 (CH_{Ar}), 45.3 (CH_2), 45.2 (CH_2), 44.1 (CH_2), 36.4 (CH_2), 25.8 (CH_2), 21.7 (CH_3), 20.7 (PCH_3). MS (EI) m/z (%): 385 (M^+, 35), 292 (22), 263 (100), 249 (36), 237 (46), 122 (43), 93 (47). HRMS (EI): calcd. for $C_{20}H_{24}N_3OPS$: 385.1378 (M^+); found: 385.1387.

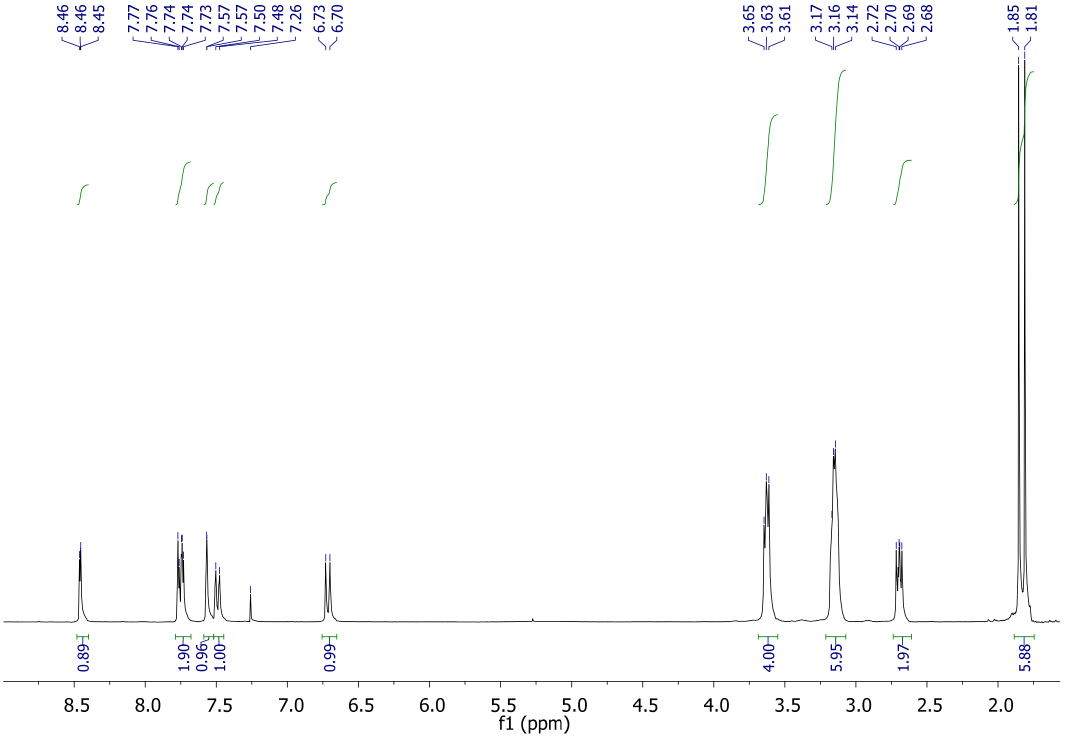

Figure S34: ^{1}H NMR ($CDCl_3$, 300 MHz) of 5-(6-(4-(dimethylphosphorothioyl)piperazin-1-yl)pyridin-3-yl)-indan-1-one BD118

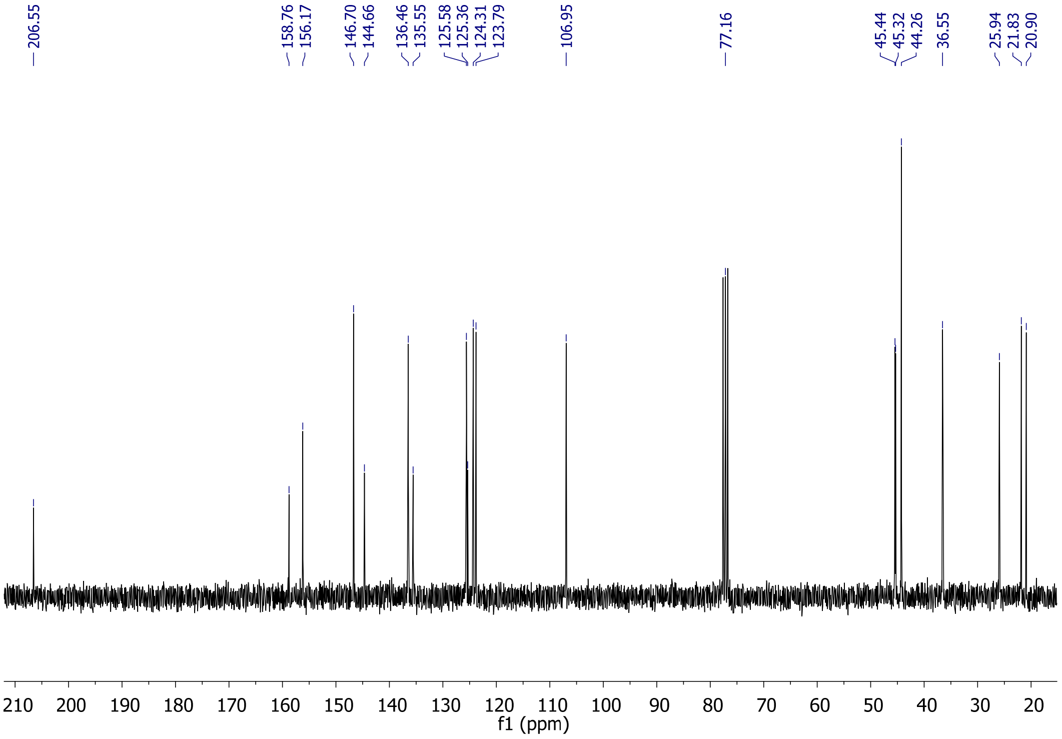

Figure S35: ^{13}C NMR ($CDCl_3$, 75 MHz) of 5-(6-(4-(dimethylphosphorothioyl)piperazin-1-yl)pyridin-3-yl)-indan-1-one BD118

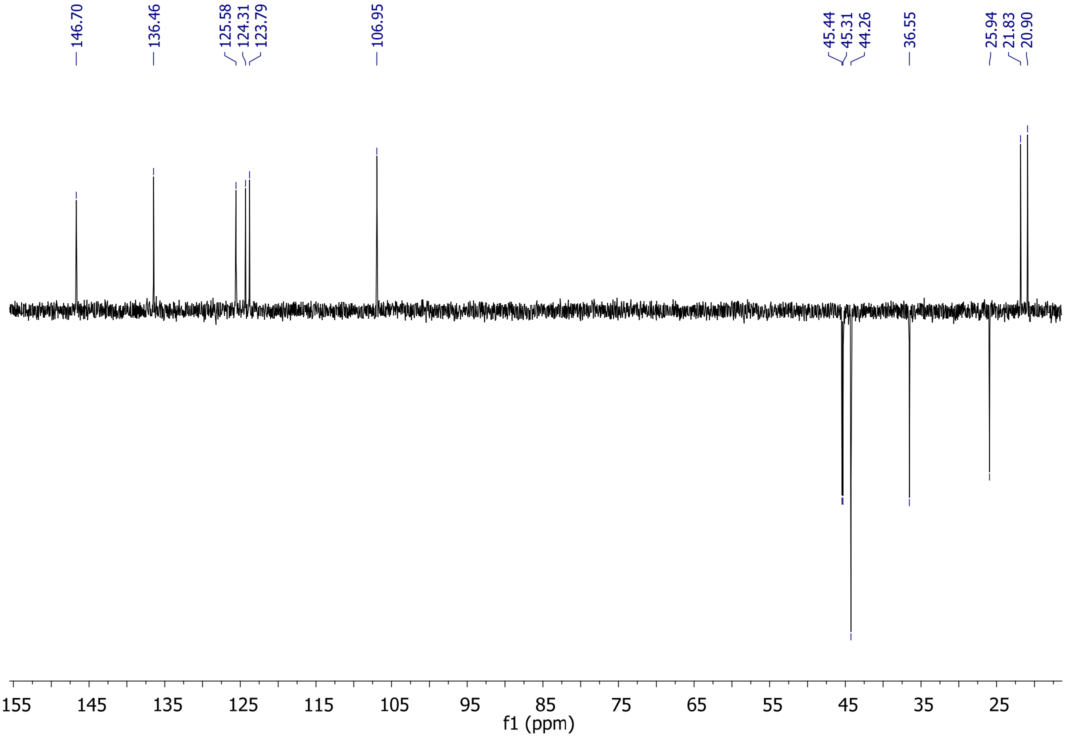

Figure S36: DEPT ($CDCl_3$, 75 MHz) of 5-(6-(4-(dimethylphosphorothioyl)piperazin-1-yl)pyridin-3-yl)-indan-1-one BD118

Solvatochromism

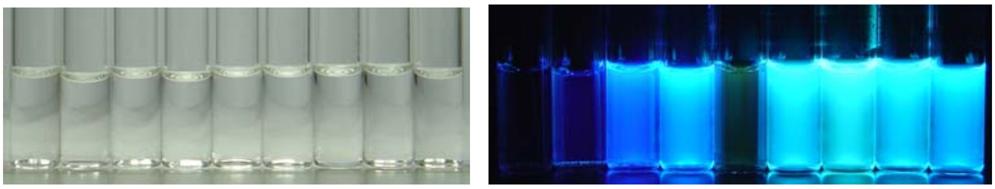

(1) (2) (3) (4) (5) (6) (7) (8) (9) (1) (2) (3) (4) (5) (6) (7) (8) (9)
(a) (b)

Figure S37: BD118 10^{-4} M in (1) Hexane, (2) Et_2O, (3) THF, (4) CH_2Cl_2, (5) MeOH, (6) MeCN, (7) DMSO, (8) DMF or (9) Acetone under (a) direct sunlight and (b) 366 nm light.

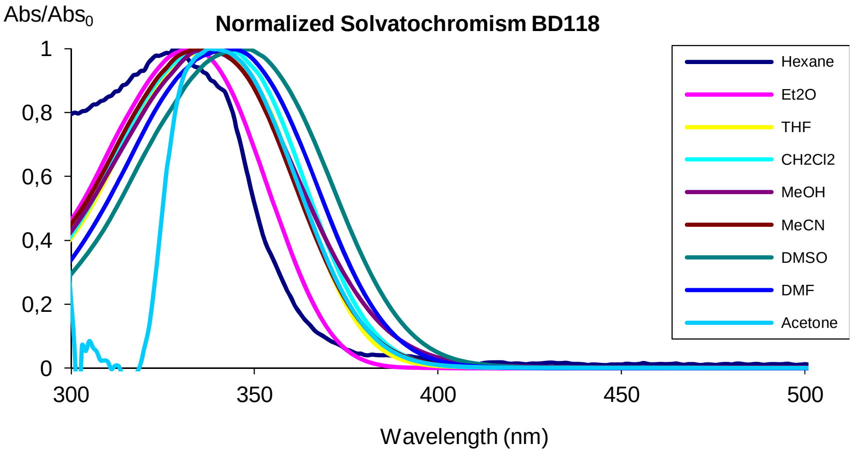

Figure S38: Normalized absorption spectra of **BD118** 10^{-4} M in different solvents.

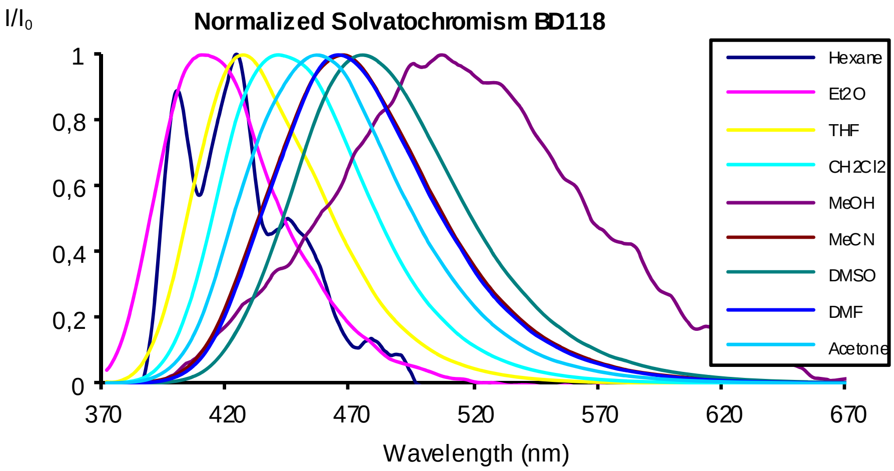

Figure S39: Normalized emission spectra of **BD118** 10^{-4} M in different solvents. λ_{exc} = 366 nm.

SYNTHESIS OF BD117:

Synthesis of tert-butyl 4-(5-(1-(dicyanomethylene)indan-5-yl)pyridin-2-yl)piperazine-1-carboxylate:

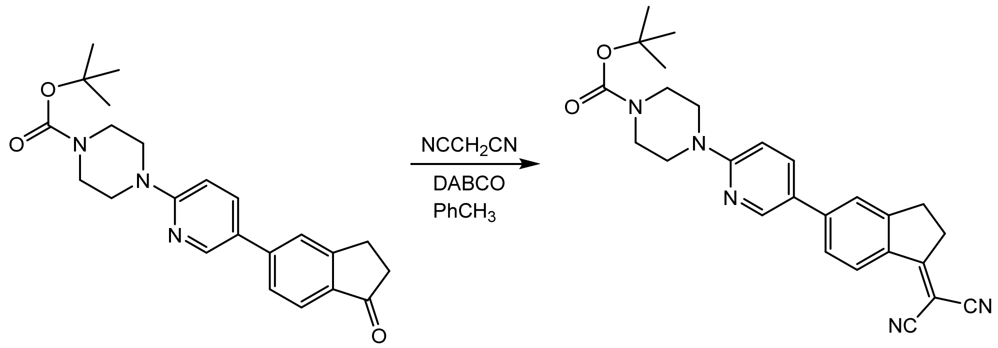

Malononitrile (570 mg, 8.64 mmol) and DABCO (342 mg, 3.05 mmol) were added to a solution of tert-butyl 4-(5-(1-oxoindan-5-yl)pyridin-2-yl)piperazine-1-carboxylate **3c** (1000 mg, 2.54 mmol) in toluene (100 mL). The mixture was heated under reflux for 24 h. Then the mixture was added to water (100 mL acidified with 5 mL of HCl 1 M) and extracted with CH_2Cl_2 (4 x 60 mL). The combined organic extracts were dried (Na_2SO_4) and the solvent was evaporated under reduced pressure. The crude product was purified by flash chromatography (silica, 3 x 30 cm) from CH_2Cl_2 to CH_2Cl_2:AcOEt (10:1), to get tert-butyl 4-(5-(1-(dicyanomethylene)indan-5-yl)pyridin-2-yl)piperazine-1-carboxylate (760 mg, 68%) as a yellow solid, mp: 210-211 ºC (decomp.). IR (KBr, cm^{-1}): 2976, 2909, 2842, 2223 (C≡N), 1690 (C=O), 1599, 1557, 1516, 1409, 1314, 1240, 1168, 1127, 943, 805. ^{1}H NMR ($CDCl_3$, 300 MHz) δ: 8.51 (d, J = 2.5 Hz, 1H, H-C=N), 8.42 (d, J = 8.3 Hz, 1H, ArH), 7.79 (dd, 1H, J_1 = 8.9 Hz, J_2 = 2.5 Hz, ArH), 7.61 (s, 1H, ArH), 7.59 (d, J = 8.3 Hz, 1H, ArH), 6.73 (d, J = 8.9 Hz, 1H, ArH), 3.66-3.55 (m, 8H, 4×CH_2), 3.32-3.20 (m, 4H, 2×CH_2), 1.49 (s, 9H, 3×CH_3). ^{13}C NMR & DEPT ($CDCl_3$, 75 MHz) δ: 178.4, 159.0, 155.3, 154.8, 147.0 (CH_{Ar}), 145.2, 136.2 (CH_{Ar}), 134.1, 126.7 (CH_{Ar}), 125.8 (CH_{Ar}), 124.0, 122.6 (CH_{Ar}), 113.9 (CN), 113.4 (CN), 106.9 (CH_{Ar}), 80.2, 73.1, 44.9 (CH_2), 43.2 (CH_2), 34.8 (CH_2), 29.7 (CH_2), 28.5 (CH_3). MS (EI) m/z (%): 441 (M^+, 21), 311 (16), 299 (29), 285 (100), 272 (28), 257 (15). HRMS (EI): calcd. for $C_{26}H_{27}N_5O_2$: 441.2165 (M^+); found: 441.2171.

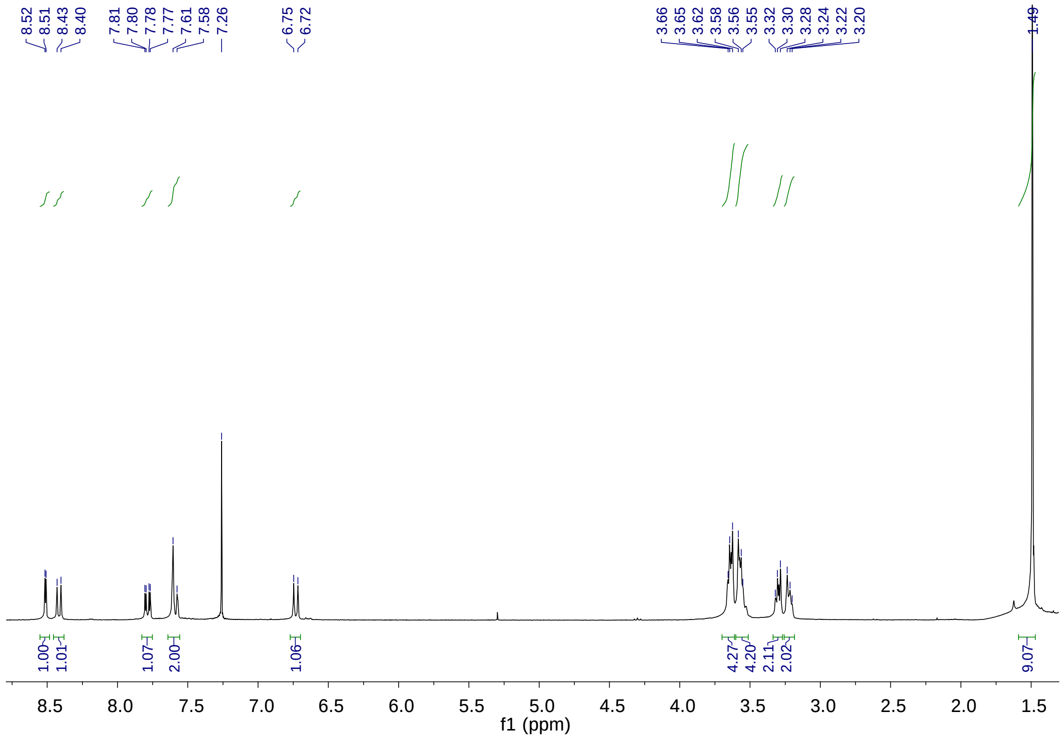

Figure S40: ^{1}H NMR ($CDCl_3$, 300 MHz) of tert-butyl 4-(5-(1-(dicyanomethylene)indan-5-yl)pyridin-2-yl)piperazine-1-carboxylate

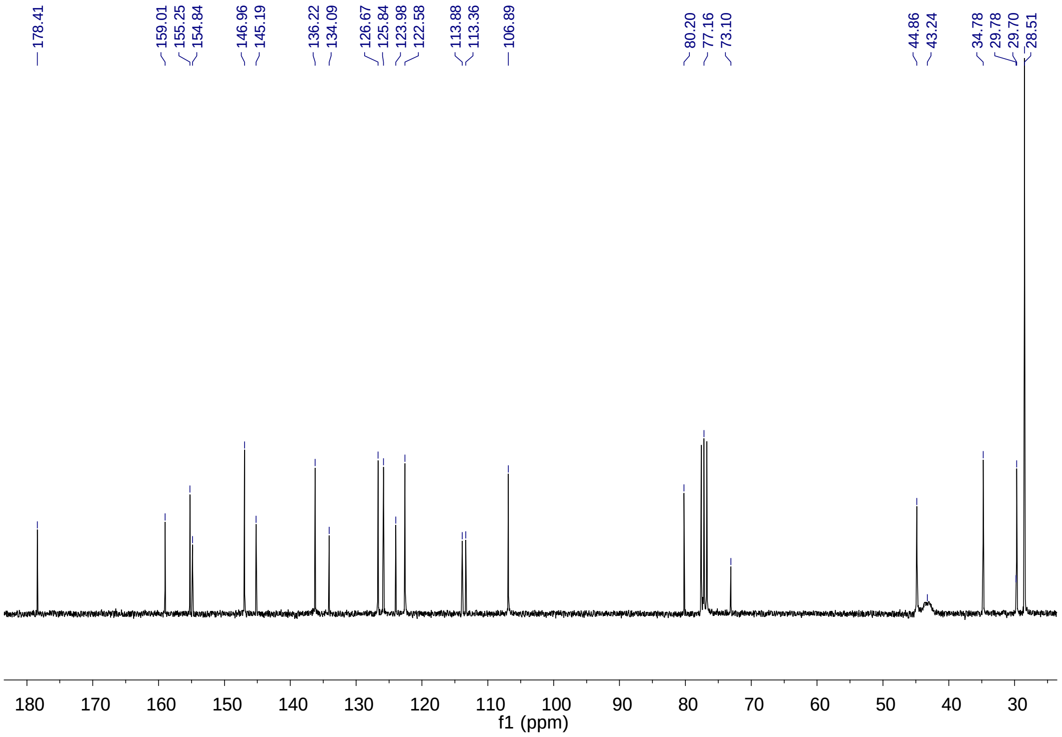

Figure S41: ^{13}C NMR ($CDCl_3$, 75 MHz) of tert-butyl 4-(5-(1-(dicyanomethylene)indan-5-yl)pyridin-2-yl)piperazine-1-carboxylate

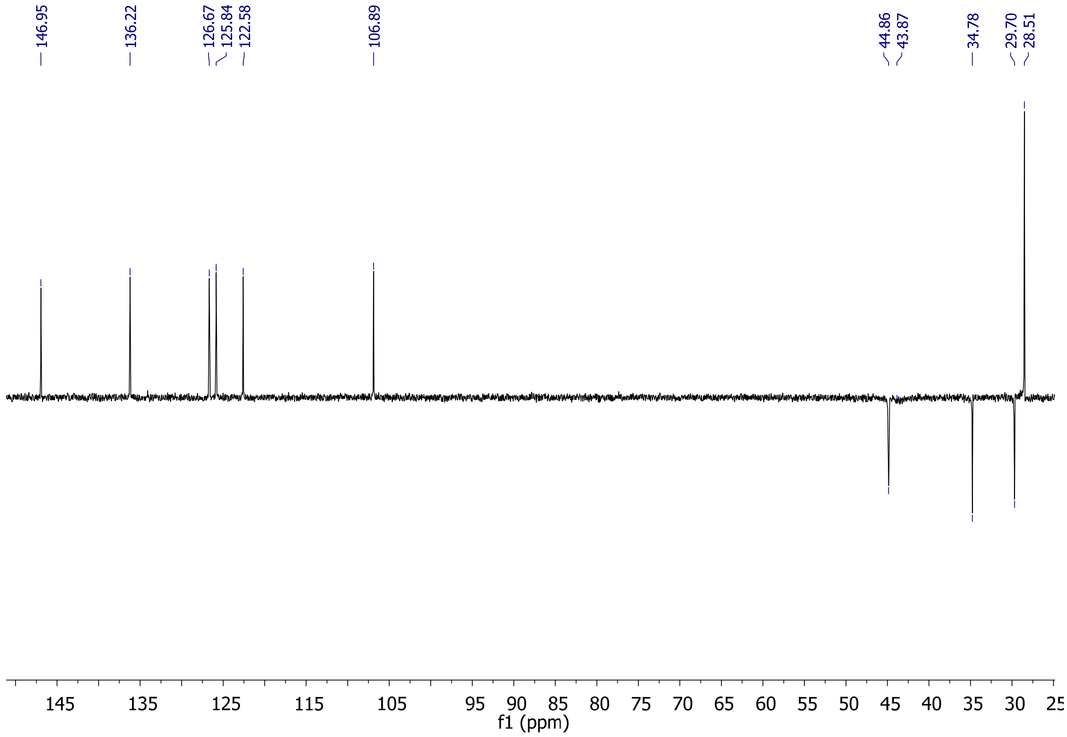

Figure S42: DEPT ($CDCl_3$, 75 MHz) of tert-butyl 4-(5-(1-(dicyanomethylene)indan-5-yl)pyridin-2-yl)piperazine-1-carboxylate

Synthesis of 1-dicyanomethylene-5-[(2-piperazin-1-yl)pyridin-3-yl]indane 1d:

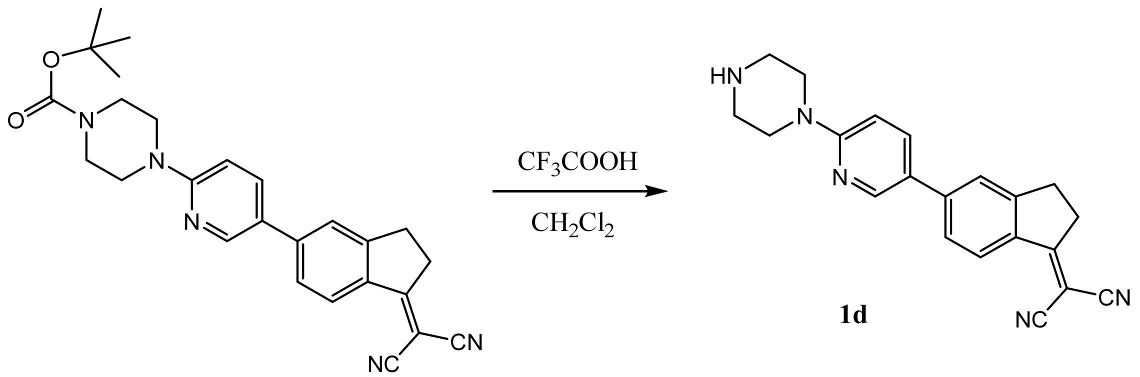

Trifluoroacetic acid (9 mL, ρ= 1.489 g/mL, 117.53 mmol) was added dropwise under stirring to a solution of tert-butyl 4-(5-(1-(dicyanomethylene)indan-5-yl)pyridin-2-yl)piperazine-1-carboxylate (750 mg, 1.70 mmol) in CH_2Cl_2 (20 mL) and the resulting mixture was stirred at room temperature for 20 minutes. Then the mixture was added to water (50 mL), basified to pH = 10 with 5% NaOH and extracted with CH_2Cl_2 (5 x 40 mL). The combined organic extracts were dried (Na_2SO_4), the solvent evaporated under reduced pressure and the residue was recrystallized from CH_2Cl_2-MeOH (5:1) to get 1-dicyanomethylene-5-[(2-piperazin-1-yl)pyridin-3-yl]indane **1d** (576 mg, 98%) as an orange solid, mp: 162-163 ºC (decomp.). IR (KBr, cm^{-1}): 3425 (N-H), 2940, 2919, 2832, 2223 (C≡N), 1598, 1557, 1511, 1307, 1250, 958, 815. ^{1}H NMR ($CDCl_3$, 300 MHz) δ: 8.52 (d, J = 2.5 Hz, 1H, H-C=N), 8.42 (d, J = 8.3 Hz, 1H, ArH), 7.77 (dd, J_1 = 9.0 Hz, J_2 = 2.5 Hz, 1H, ArH), 7.60 (s, 1H, ArH), 7.59 (d, J = 8.3 Hz, 1H, ArH), 6.73 (d, J = 9.0 Hz, 1H, ArH), 3.62 (t, J = 5.0 Hz, 4H, 2×CH_2), 3.32-3.20 (m, 4H, 2×CH_2), 3.00 (t, J = 5.0 Hz, 4H, 2×CH_2), 1.64 (s, 1H, NH). ^{13}C NMR & DEPT ($CDCl_3$, 75 MHz) δ: 178.5, 159.5, 155.3, 147.0 (CH_{Ar}), 145.5, 136.2 (CH_{Ar}), 134.1, 126.8 (CH_{Ar}), 125.9 (CH_{Ar}), 123.7, 122.6 (CH_{Ar}), 114.0 (CN), 113.5 (CN), 106.8 (CH_{Ar}), 73.1, 46.2 (CH_2), 46.1 (CH_2), 34.9 (CH_2), 29.8 (CH_2). MS (EI) m/z (%): 341 (M^+, 32), 311 (22), 299 (100), 285 (85), 273 (91), 257 (17). HRMS (EI): calcd. for $C_{21}H_{19}N_5$: 341.1640 (M^+); found: 341.1637.

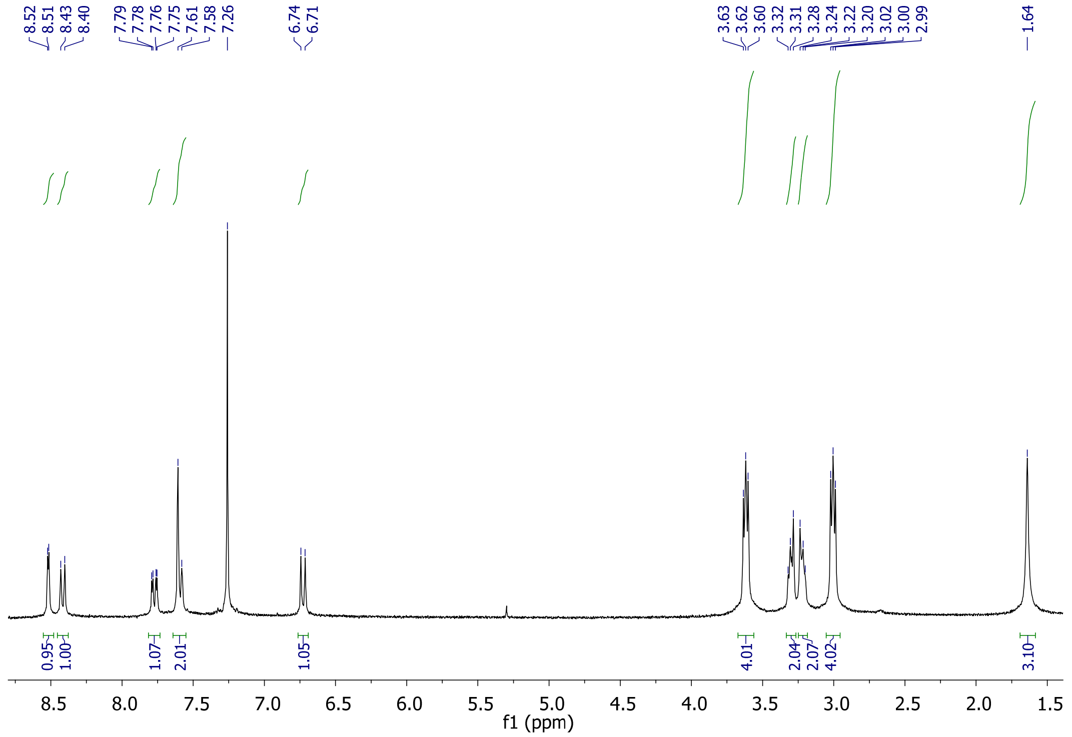

Figure S43: ^{1}H NMR ($CDCl_3$, 300 MHz) of 1-dicyanomethylene-5-[(2-piperazin-1-yl)pyridin-3-yl]indane 1d

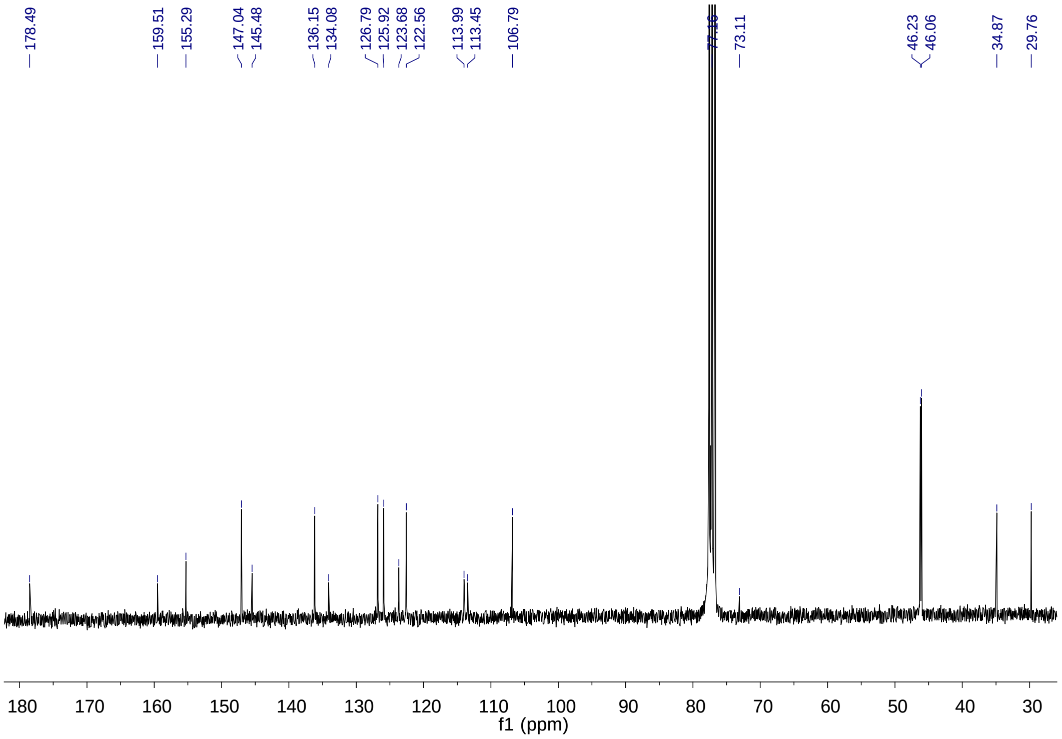

Figure S44: ^{13}C NMR ($CDCl_3$, 75 MHz) of 1-dicyanomethylene-5-[(2-piperazin-1-yl)pyridin-3-yl]indane 1d

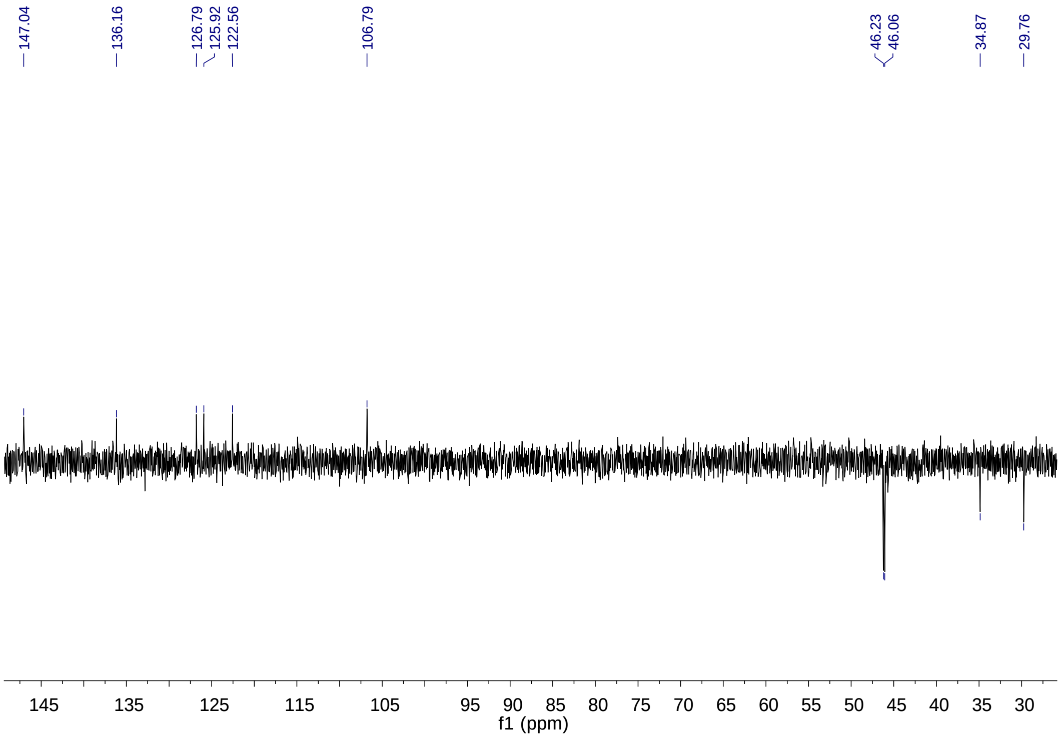

Figure S45: DEPT ($CDCl_3$, 75 MHz) of 1-dicyanomethylene-5-[(2-piperazin-1-yl)pyridin-3-yl]indane 1d

Synthesis of 2-(5-(6-(4-(dimethylphosphorothioyl)piperazin-1-yl)pyridin-3-yl)-indan-1-ylidene)malononitrile BD117:

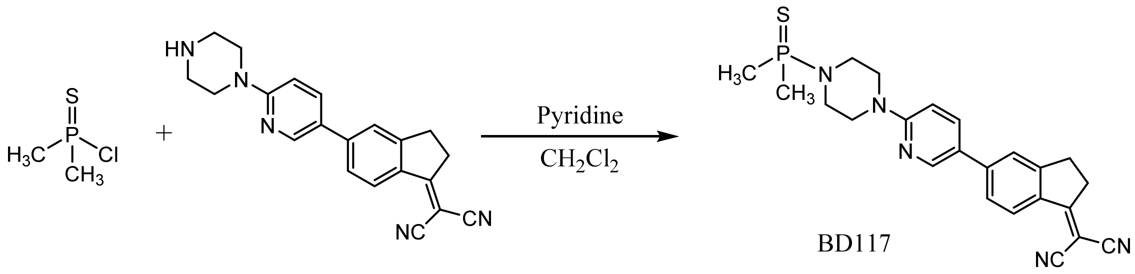

Dimethylthiophosphinic chloride (13.9 µL, ρ = 1.230 g/mL, 0.13 mmol) was added to a solution of 2-(5-(6-(piperazin-1-yl)pyridin-3-yl)-indan-1-ylidene)malononitrile **1d** (50 mg, 0.15 mmol) in CH_2Cl_2 (20 mL) in an ice bath. Then, pyridine (23.6 µL, ρ = 0.982 g/mL, 0.29 mmol) was added to the mixture and the resulting mixture was stirred for 15 minutes at 0 ºC, then for 30 minutes at room temperature and then for 5 hours at 35 ºC. The mixture was then added to acidified water (3 mL of 0.1 M HCl in 30 mL water) and extracted with dichloromethane (5 × 20 mL). The combined organic extracts were dried over anhydrous Na_2SO_4 and the solvent evaporated under reduced pressure. The crude product was purified by flash chromatography (silica, 1 × 20 cm) from CH_2Cl_2 to CH_2Cl_2:MeOH (100:2), to get 2-(5-(6-(4-(dimethylphosphorothioyl)piperazin-1-yl)pyridin-3-yl)-indan-1-ylidene)malononitrile BD117 (55 mg, 87%) as an orange solid. mp: 189-190 ºC (decomp.). IR (KBr, cm^{-1}): 2958, 2924, 2849, 2215 (C≡N), 1594, 1556, 1506, 1248, 1156, 961. ^{1}H NMR ($CDCl_3$, 300 MHz) δ: 8.48 (d, J = 2.0 Hz, 1H, H-C=N), 8.38 (d, J = 8.4 Hz, 1H, ArH), 7.78 (dd, J_1 = 8.9 Hz, J_2 = 2.0 Hz, 1H, ArH), 7.57 (m, 2H, ArH), 6.73 (d, J = 8.9 Hz, 1H, ArH), 3.67 (t, J = 5.0 Hz, 4H, 2×CH_2), 3.28-3.14 (m, 8H, 4×CH_2), 1.87 (s, 3H, P-CH_3), 1.83 (s, 3H, P-CH_3). ^{13}C NMR ($CDCl_3$, 75 MHz) δ: 178.5, 158.9, 155.3, 146.9 (CH_{Ar}), 145.1, 136.3 (CH_{Ar}), 134.1, 126.7 (CH_{Ar}), 125.9 (CH_{Ar}), 124.1, 122.7 (CH_{Ar}), 113.9 (C≡N), 113.4 (C≡N), 107.0 (CH_{Ar}), 73.1, 45.4 (CH_2), 45.2 (CH_2), 44.3 (CH_2), 34.8 (CH_2), 29.7 (CH_2), 21.9 (P-CH_3), 20.9 (P-CH_3). MS (EI) m/z (%): 433 (M^+, 42), 340 (24), 311 (100), 299 (44), 285 (65), 257 (19), 122 (97), 92 (79). HRMS (EI): calcd. for $C_{23}H_{24}N_5PS$: 433.1490 (M^+); found: 433.1493.

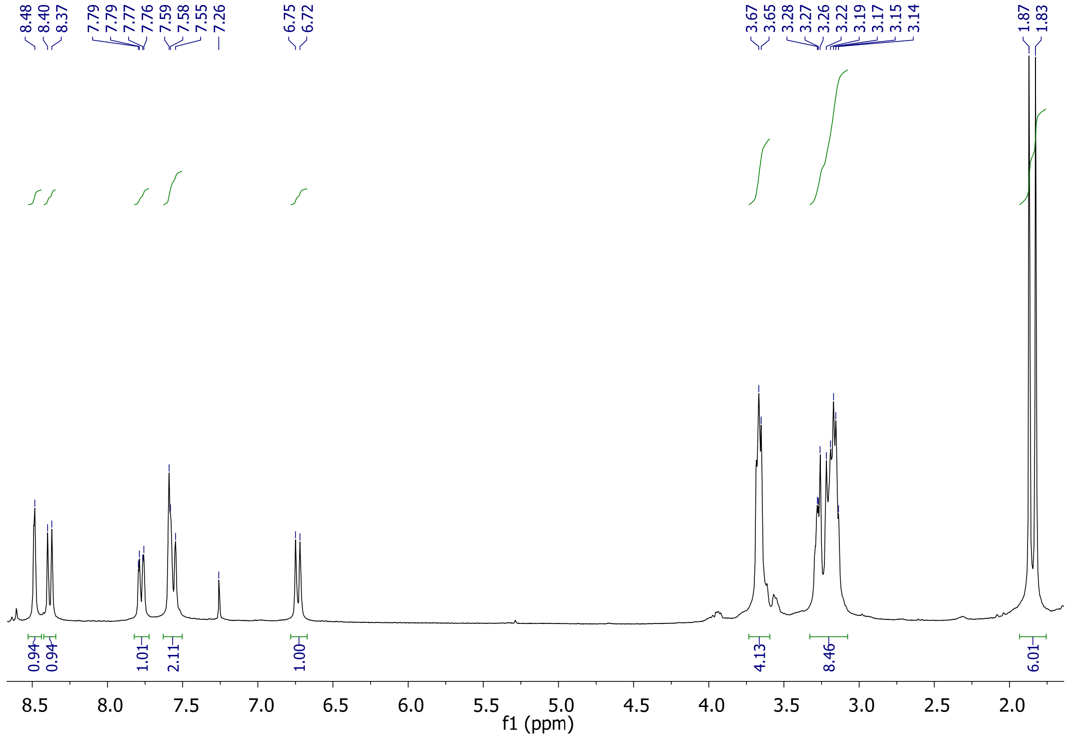

Figure S46: ^{1}H NMR ($CDCl_3$, 300 MHz) of 2-(5-(6-(4-(dimethylphosphorothioyl)piperazin-1-yl)pyridin-3-yl)-indan-1-ylidene)malononitrile BD117

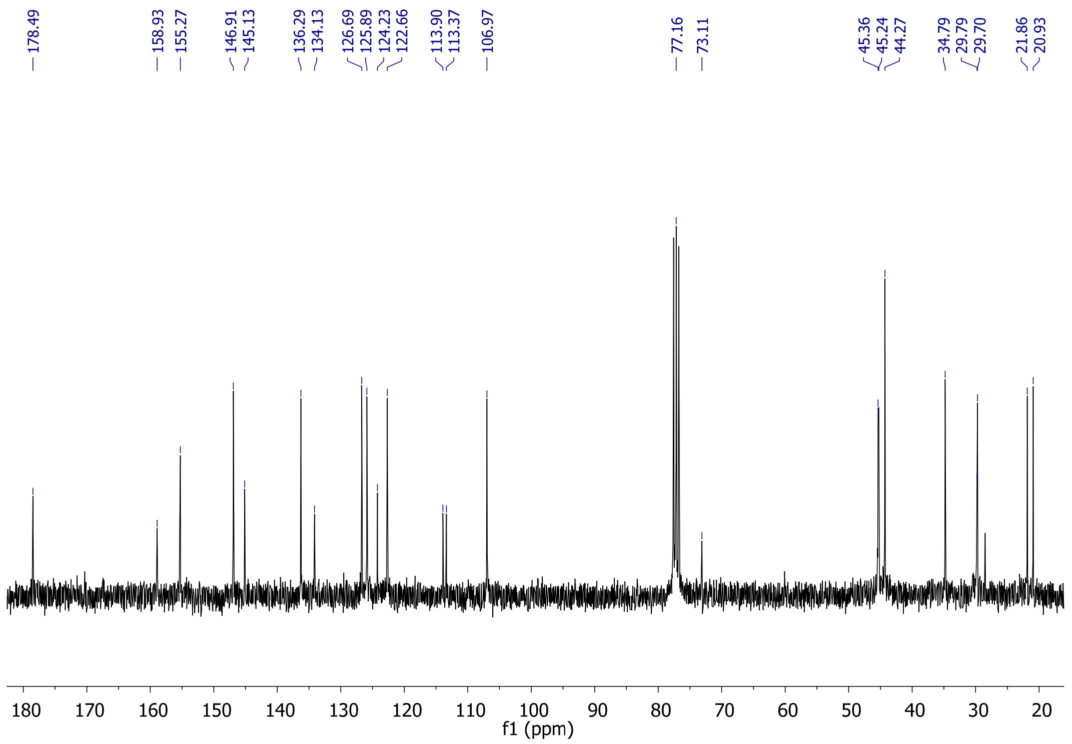

Figure S47: ^{13}C NMR ($CDCl_3$, 75 MHz) of 2-(5-(6-(4-(dimethylphosphorothioyl)piperazin-1-yl)pyridin-3-yl)-indan-1-ylidene)malononitrile BD117

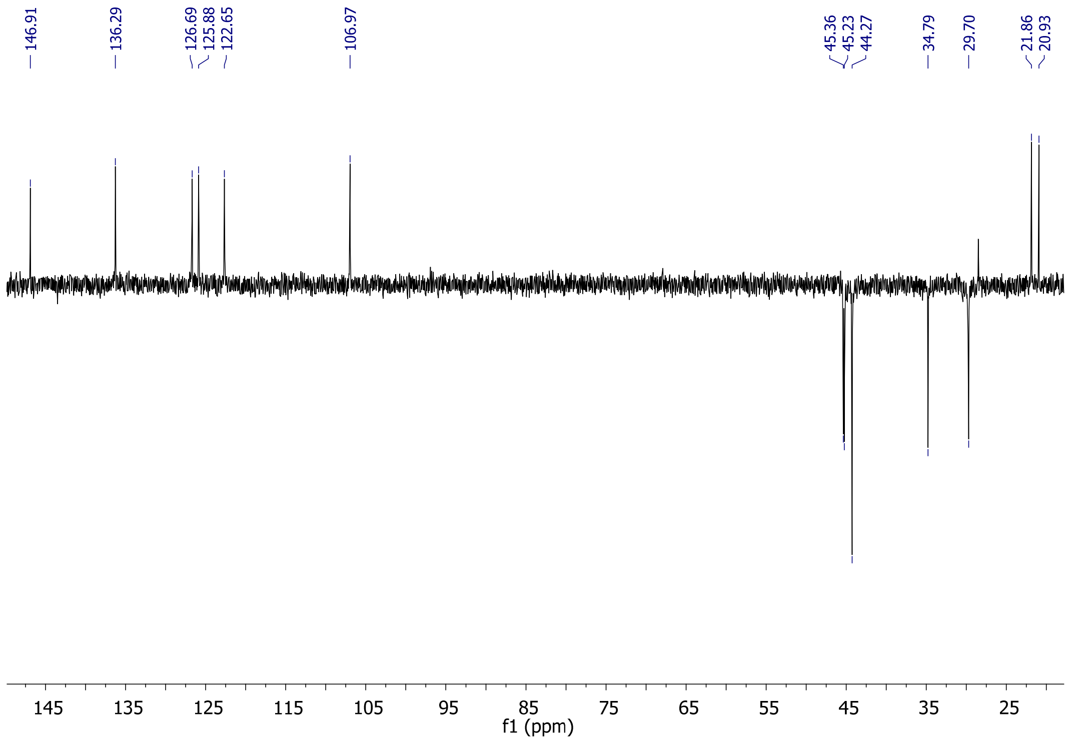

Figure S48: DEPT ($CDCl_3$, 75 MHz) of 2-(5-(6-(4-(dimethylphosphorothioyl)piperazin-1-yl)pyridin-3-yl)-indan-1-ylidene)malononitrile BD117

Solvatochromism

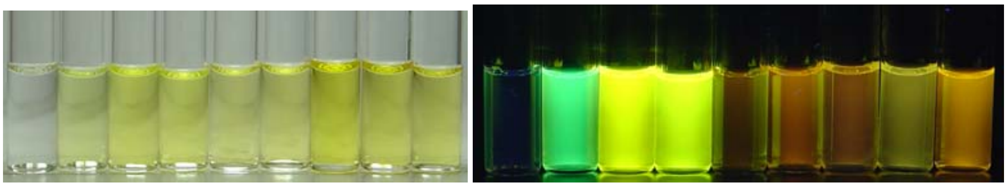

(1) (2) (3) (4) (5) (6) (7) (8) (9) (1) (2) (3) (4) (5) (6) (7) (8) (9)

(a) (b)

Figure S49: BD117 10^{-4} M in (1) Hexane, (2) Et_2O, (3) THF, (4) CH_2Cl_2, (5) MeOH, (6) MeCN, (7) DMSO, (8) DMF or (9) Acetone under (a) direct sunlight and (b) 366 nm light.

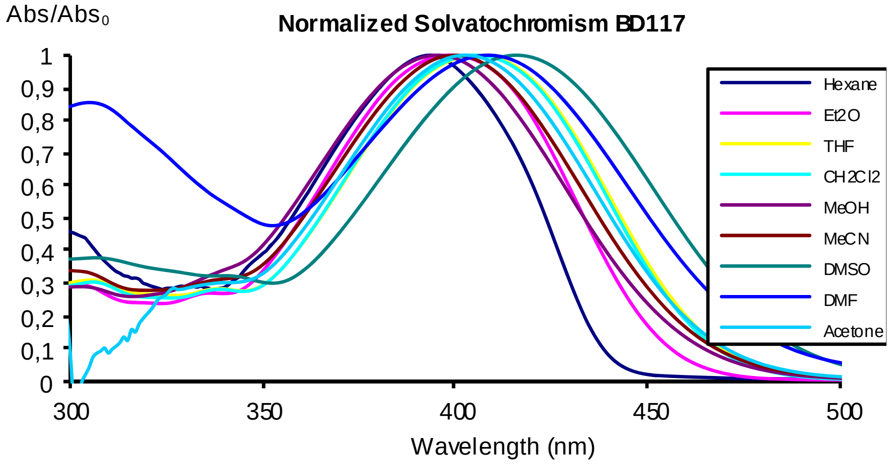

Figure S50: Normalized absorption spectra of **BD117** 10^{-4} M in different solvents.

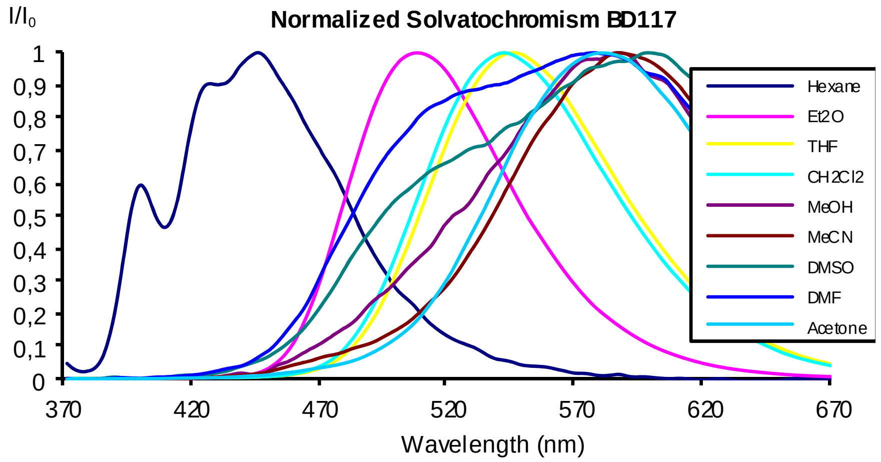

Figure S51: Normalized emission spectra of **BD117** 10^{-4} M in different solvents. $\lambda_{exc} = 366$ nm.

Solvent-dependent study of fluorescence of BD116-BD119 by the Lippert-Mataga equations:

Solvent-dependent Stokes shifts can be described in terms of general and specific solvent effects.[9] General solvent effects are interpreted in terms of the Lippert-Mataga equation (see eq. below), which describes the Stokes shift in terms of the changes in dipole moment which occur upon excitation, and the energy of a dipole in solvents of various dielectric constants (ε) or refractive indexes (n). These general solvent effects occur whenever a fluorophore is dissolved in any solvent, and they are independent of the chemical properties of the fluorophore or the solvent.

$$\bar{\nu}_A - \bar{\nu}_F = \frac{2}{hc}\left(\frac{\varepsilon - 1}{2\varepsilon + 1} - \frac{n^2 - 1}{2n^2 + 1}\right)\frac{(\mu_E - \mu_G)^2}{a^3} + \text{constant}$$

Equation 1. Lippert-Mataga equation. In this equation *h* (= 6.6256 × 10^{-27} ergs) is the Planck's constant, *c* (= 2.9979 × 10^{10} cm/s) is the speed of light, and *a* is the radius of the cavity within the fluorophore resides. v_A and v_F are the wavenumbers (cm^{-1}) of the absorption and emission, respectively.

However, the theory for general solvent effects is usually inadequate for explaining many environments, because fluorophores often interact with their local environment, and therefore with the solvent. Those are the so called *specific solvent effects*, i.e. hydrogen bonding, complex formation or formation of charge transfer states. The presence of specific solvent-fluorophore interactions is often indicated by changes in the shape of emission spectra.

If we only consider *general solvent effects*, the plot of the data obtained from solvatochromism attending to the Lippert-Mataga equation or versus another physicochemical parameter of solvents, such as ET(30), should give a straight line. This sort of plots was performed for each of the four derivatives and comparable results were obtained in all cases. The pictures show a good linearity between the physicochemical parameters represented, except for hexane, due to the low solubility of the probes in this solvent. This clearly indicates a strong influence of general solvent effects and the absence of hydrogen bonding interactions contributing to the Stokes shifts.

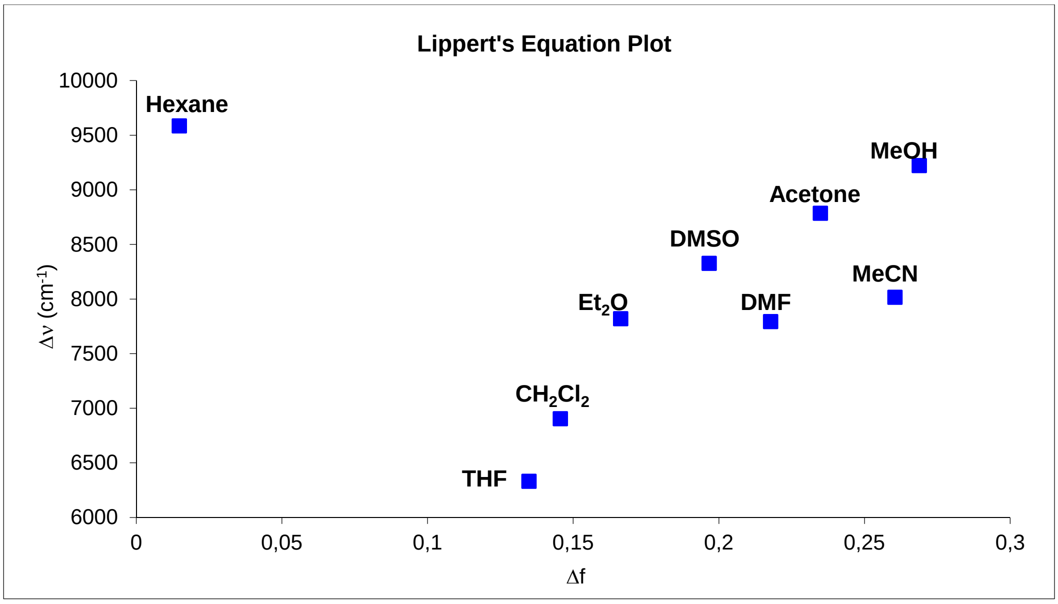

(a)

[9] J. R. Lakowicz, *Principles of Fluorescence Spectroscopy,* Springer, New York, 2006.

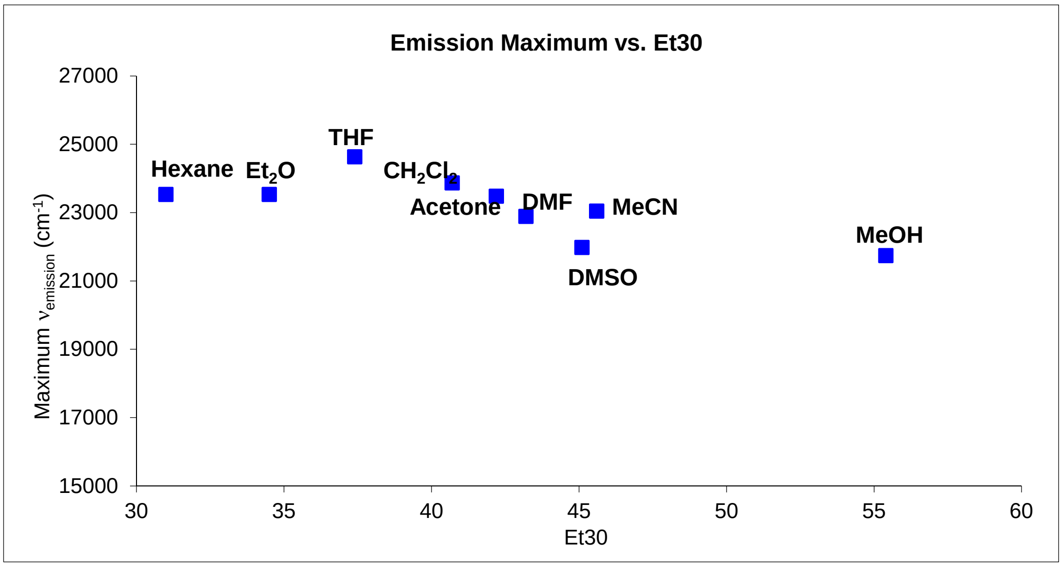

(b)

Figures S52a-b: Plots of (a) Lippert-Mataga equation and (b) ET(30) parameter for the Stokes shifts and maximum emission wavelengths obtained for BD116. ET(30) parameter refers to a solvent polarity scale commonly used.

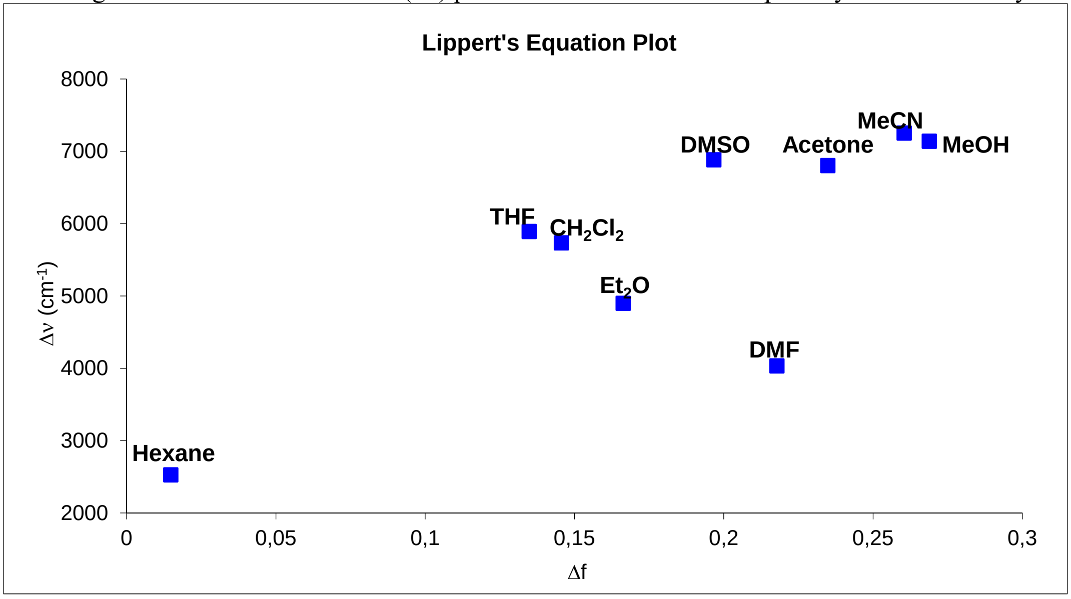

(a)

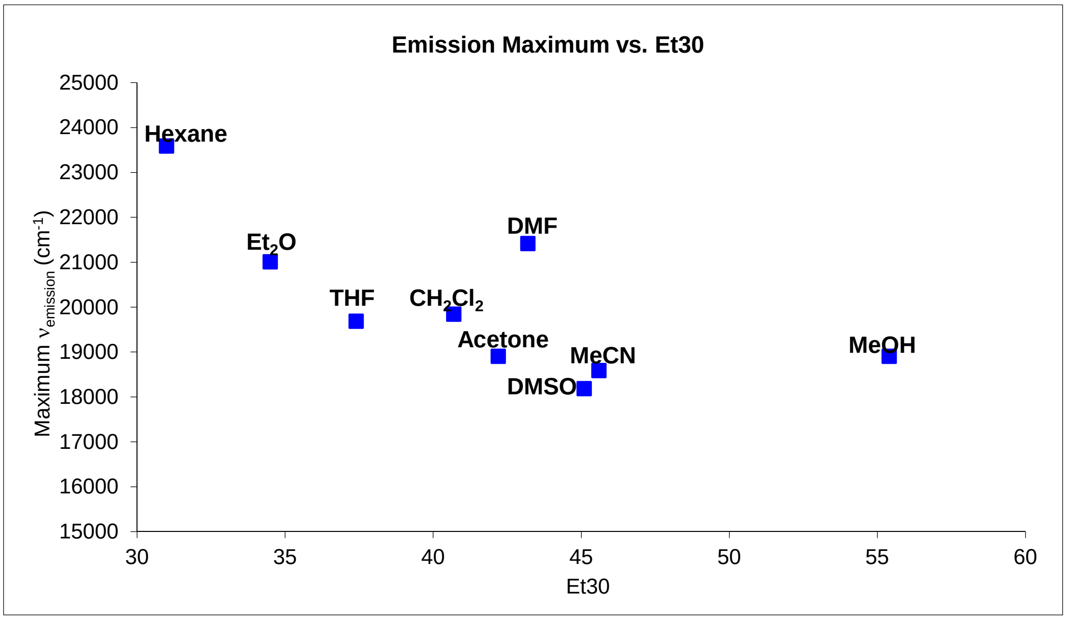

(b)

Figures S53a-b: Plots of (a) Lippert-Mataga equation and (b) ET(30) parameter for the Stokes shifts and maximum emission wavelengths obtained for BD119.

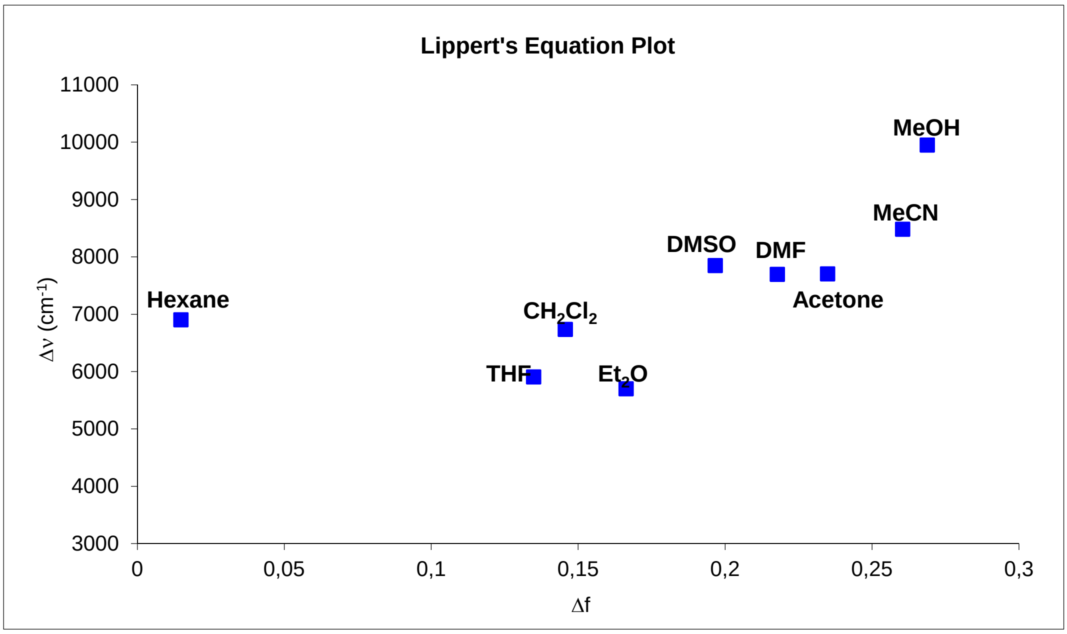

(a)

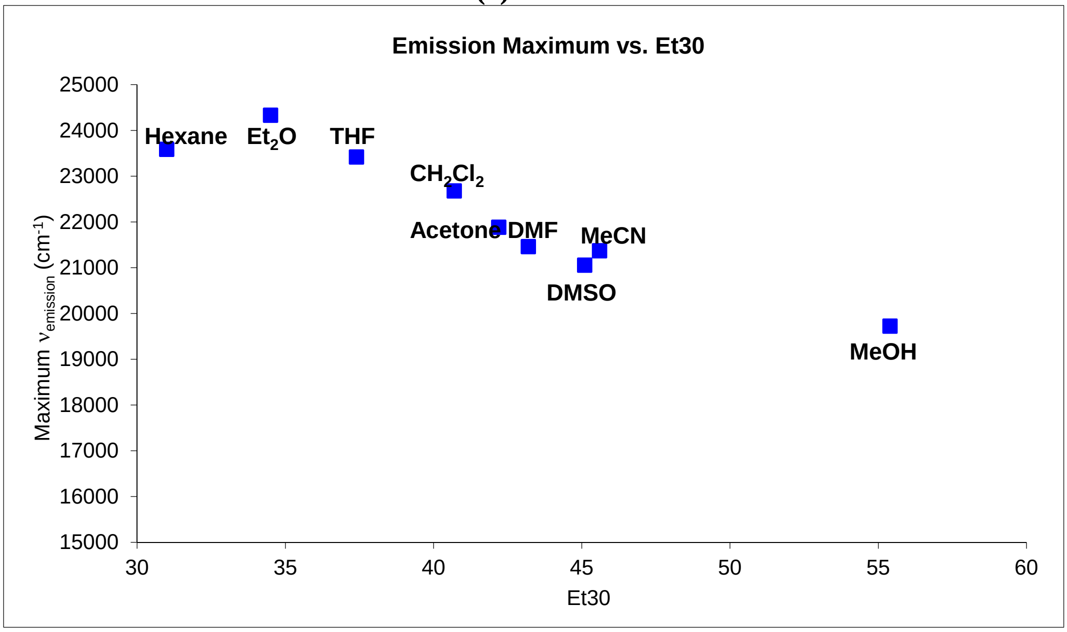

(b)

Figures S54a-b: Plots of (a) Lippert-Mataga equation and (b) ET(30) parameter for the Stokes shifts and maximum emission wavelengths obtained for BD118.

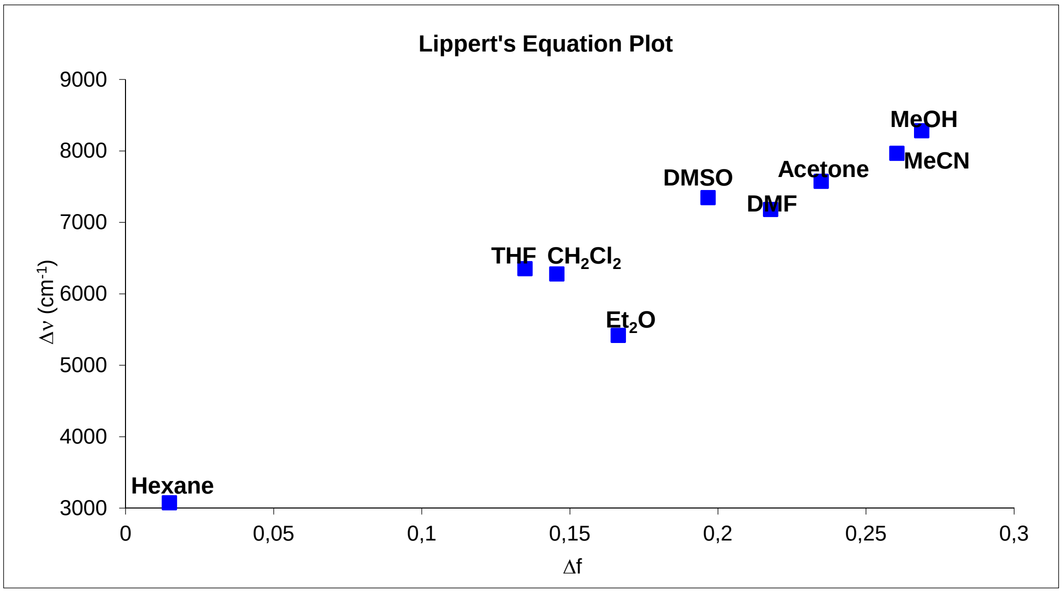

(a)

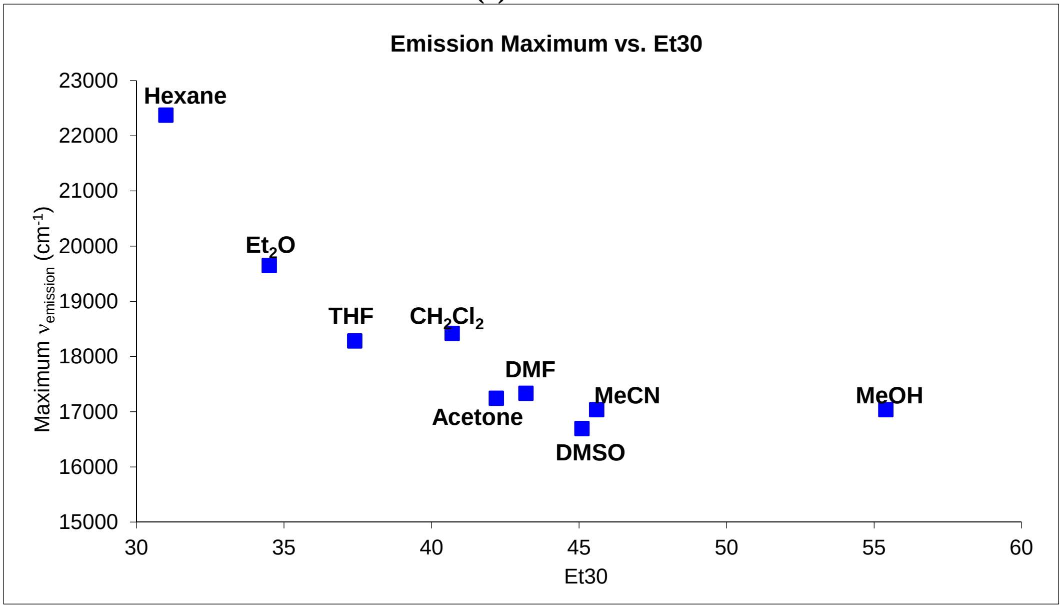

(b)

Figures S55a-b: Plots of (a) Lippert-Mataga equation and (b) ET(30) parameter for the Stokes shifts and maximum emission wavelengths obtained for BD117.

SYNTHESIS OF JG7:

Synthesis of *O,O*-diethyl(4-(5-(1-oxoindan-5-yl)pyrimidin-2-yl)piperazin-1-yl)phosphonothioate JG7:

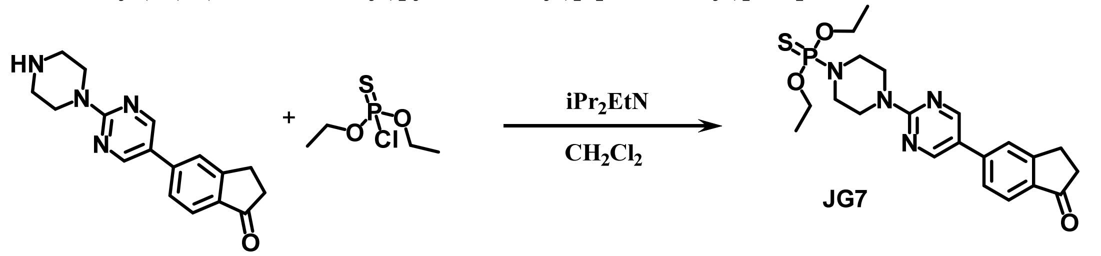

A 50 ml flask, with a magnetic stirrer, was charged with 50 mg of 5-[4-(piperazine-1-yl)pyrimidin]indan-1-one **1a** (0.17 mmol) and 20 ml of CH_2Cl_2, after five minutes under stirring in an ice bath, 24.3 µl of O,O-diethyl chlorothiophosphate (0.15 mmol) and 29.6 µl of $iPrEt_2N$ (0.17 mmol) were added to the solution. The solution was stirred for 20 minutes at 0 ºC, then stirred for 15 minutes at room temperature and then heated under reflux for 48 hours at 35 ^oC. The presence of the initial reagents was followed by thin layer chromatography. TLC chromatography was eluted with dichloromethane:methanol (50:1). Then the solvent was evaporated under reduced pressure and the residue was washed with diethylether to eliminate the remaining thiophosphate. The ether was discarded and the residue was dried under reduced pressure. The residue was treated with aqueous acid (20 ml HCl 0.1 M) and extracted with dichloromethane (3x 15 ml) to eliminate the Hünigs base. The combined organic extracts were washed with water, dried with sodium sulphate and evaporated under reduced pressure, this process allowed to obtain 66 mg of *O*,*O*-diethyl (4-(5-(1-oxoindan-5-yl)pyrimidin-2-yl)piperazin-1-yl)phosphonothioate (90% yield) as a white solid. mp: 159 – 161 ºC (decomp.). IR (KBr, cm^{-1}): 2980, 2902, 2852, 1692 (C=O), 1597, 1514, 1447, 1364, 1307, 1254, 1130, 1036, 970, 803 (P-O), 728 (P=S). ^{1}H NMR ($CDCl_3$, 300 MHz) δ: 8.57 (s, 2H, H-C=N), 7.78 (d, J = 8.0 Hz, 1H, ArH), 7.54 (s, 1H, ArH), 7.46 (d, J = 8.0 Hz, 1H, ArH), 4.02 (q, J = 8.0 Hz, 4H, P-O-CH_2), 3.84 (s, 4H, 2×CH_2) 3.34 (s, 4H, 2×CH_2), 3.17 (t, J = 5.0 Hz, 2H, CH_2), 2.71 (t, J = 5.0 Hz, 2H, CH_2), 1.30 (t, J = 7.0 Hz , 6H, 2×CH_3). ^{13}C NMR ($CDCl_3$, 75 MHz) δ: 206.3 (C=O), 161.2, 156.3 (2×CH_{Ar}), 155.1, 142.0, 136.0, 125.1 (CH_{Ar}), 124.6 (CH_{Ar}), 123.4 (CH_{Ar}), 122.1, 63.2 (CH_2), 63.1 (CH_2), 45.1 (CH_2), 44.4 (CH_2), 43.3 (CH_2), 36.5 (CH_2), 26.0 (CH_2), 16.1 (CH_2), 16.0 (CH_2). MS (EI) m/z (%): 446 (M^+, 100), 447 (M^++1, 24.81), 448 (M^++2, 8), 293 (28), 264 (91), 251 (85), 238(71). HRMS (EI): calcd. for $C_{21}H_{27}N_4O_3PS$: 446.1541 (M^+); found: 446.1542.

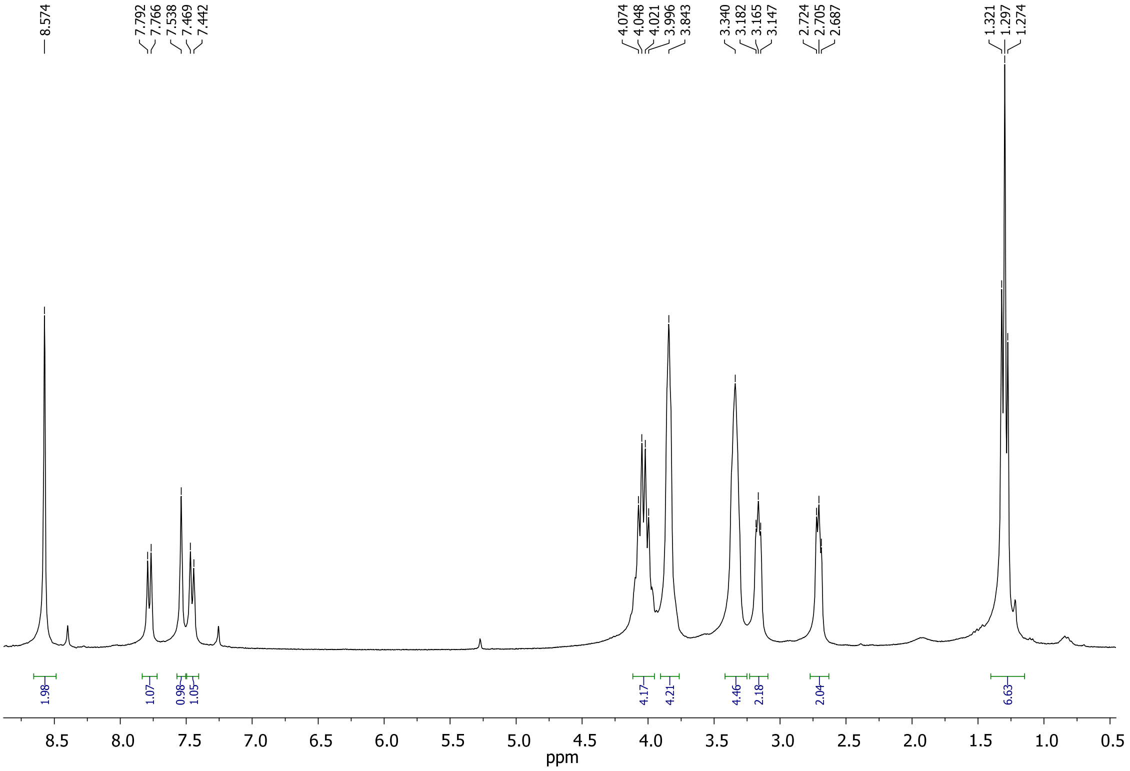

Figure S56: ^{1}H NMR ($CDCl_3$, 300 MHz) of *O,O*-diethyl(4-(5-(1-oxoindan-5-yl)pyrimidin-2-yl)piperazin-1-yl)phosphonothioate JG7

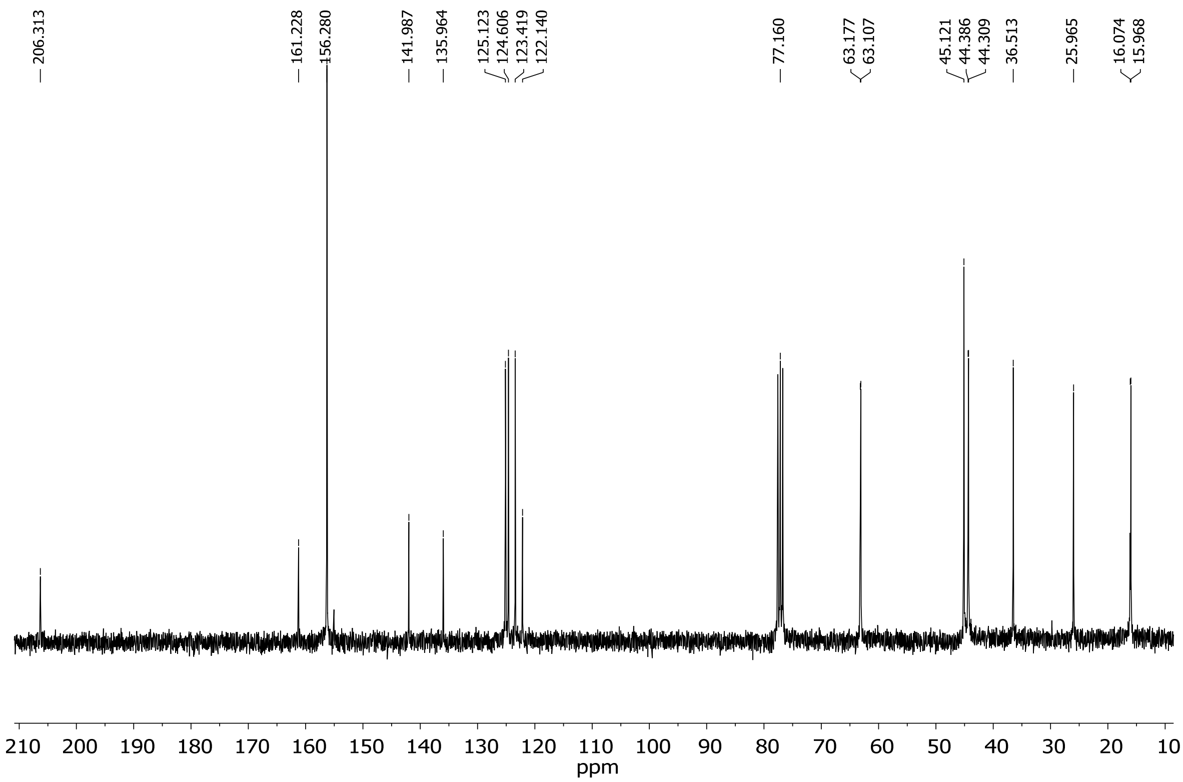

Figure S57: ^{13}C NMR ($CDCl_3$, 75 MHz) of *O,O*-diethyl(4-(5-(1-oxoindan-5-yl)pyrimidin-2-yl)piperazin-1-yl)phosphonothioate JG7

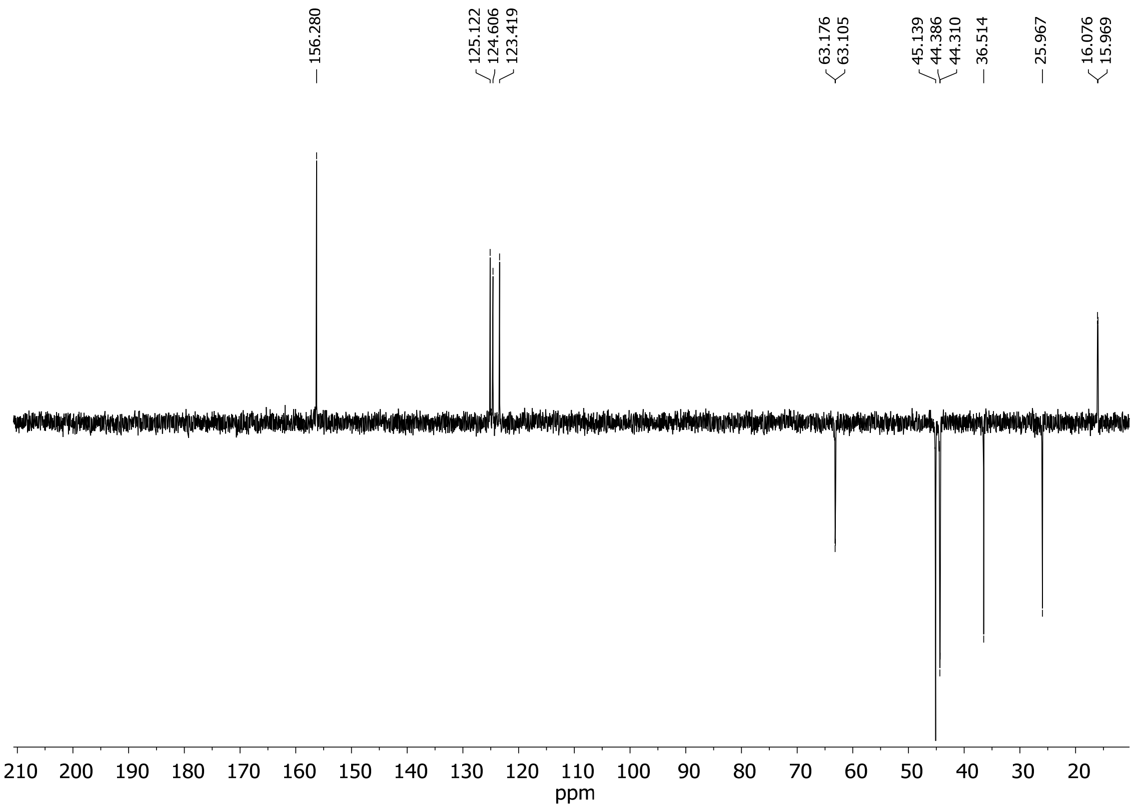

Figure S58: DEPT ($CDCl_3$, 75 MHz) of *O,O*-diethyl(4-(5-(1-oxoindan-5-yl)pyrimidin-2-yl)piperazin-1-yl)phosphonothioate JG7

Solvatochromism

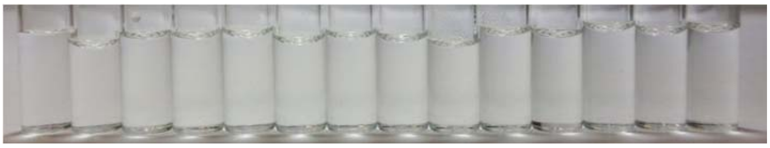

(1) (2) (3) (4) (5) (6) (7) (8) (9) (10) (11) (12) (13) (14)
(a)

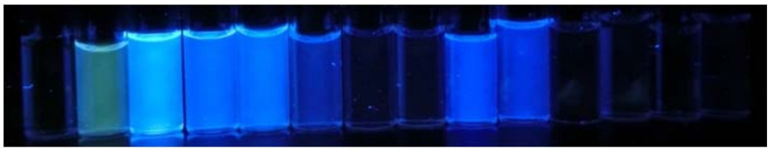

(1) (2) (3) (4) (5) (6) (7) (8) (9) (10) (11) (12) (13) (14)
(b)

Figure S59: JG7 5×10^{-5} M in (1) H_2O, (2) MeOH, (3) DMSO, (4) DMF, (5) MCN, (6) Acetone, (7) AcOEt, (8) THF (9) $CHCl_3$ (10) CH_2Cl_2 (11) Toluene (12) Et_2O (13) Hexane (14) Cyclohexane under (a) direct sunlight and (b) 366 nm light.

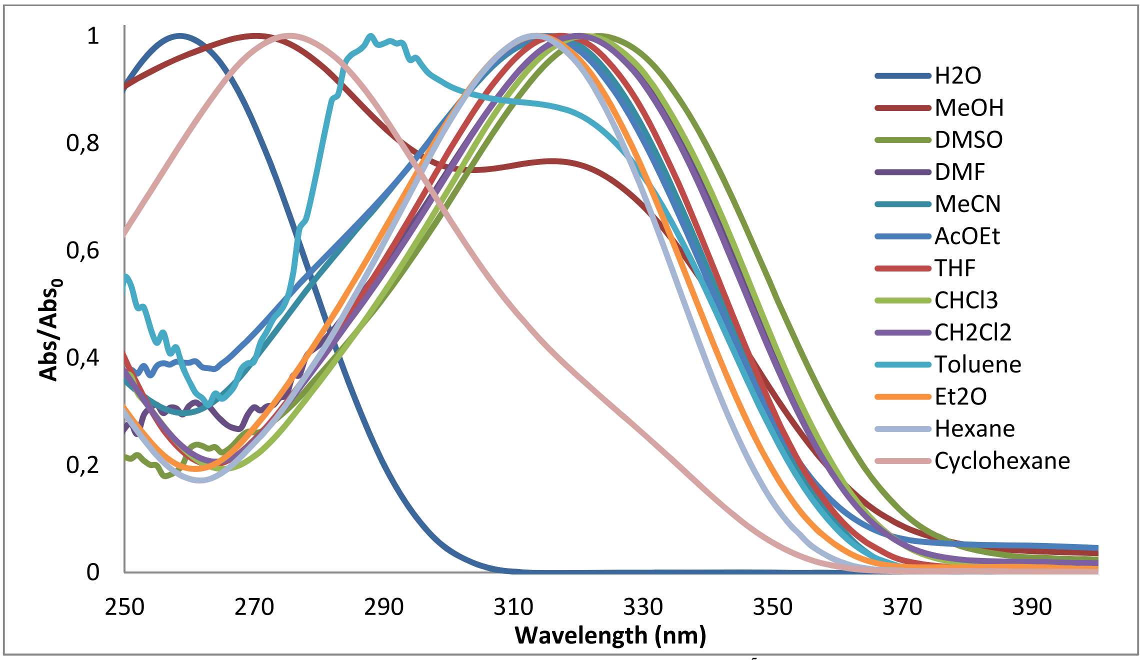

Figure S60: Normalized absorption spectra of **JG7** 5×10^{-5} M in different solvents.

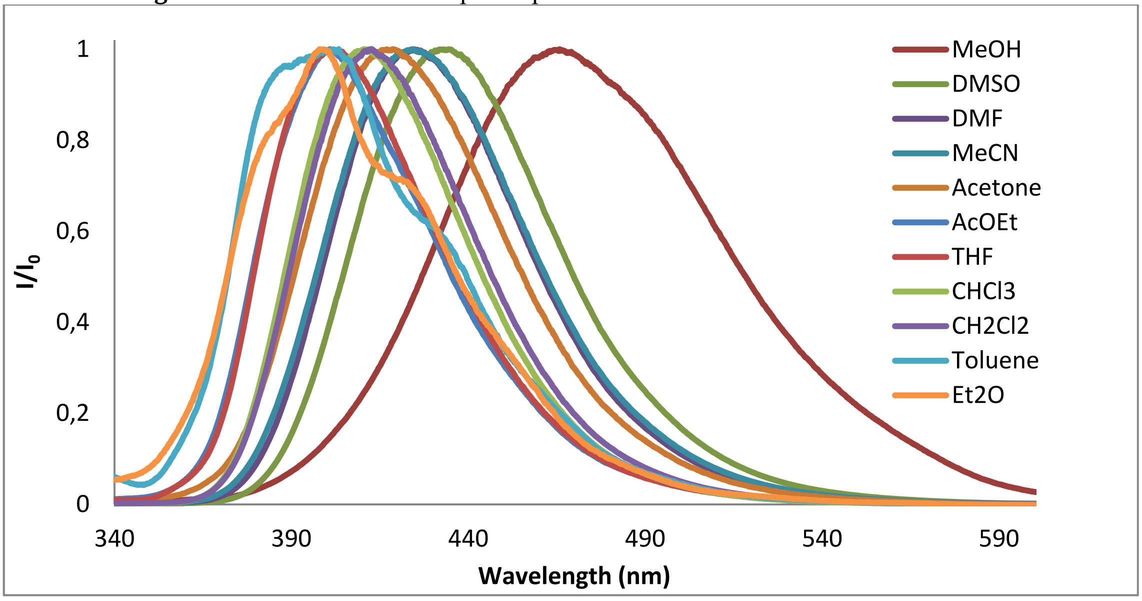

Figure S61: Normalized emission spectra of **JG7** 5×10^{-5} M in different solvents. $\lambda_{exc} = 366$ nm.

SYNTHESIS OF JG30:

***O,O*-diethyl-(4-(5-(1-(dicyanomethylene)indan-5-yl)pyrimidin-2-yl)piperazin-1-yl)phosphonothioate JG30**

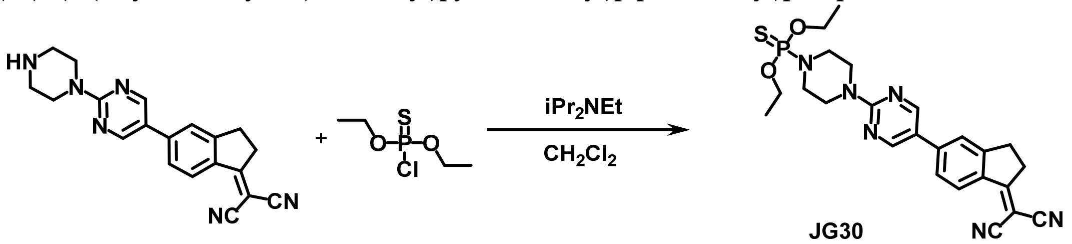

A 100 ml schlenk, under nitrogen atmosphere, was charged with 47 mg of 2-5-[2-(piperazine-1-yl)pyrimidin-5-yl]indan-1-ilydene)malononitrile **1c** (0.14 mmol) and 25 ml of dichloromethane, after five minutes under in an ice bath, 19.9 µl of *O*,*O*-diethyl chlorothiophosphate (0.13 mmol) and 22.6 µl of $^{i}Pr_2NEt$ (0.14 mmol) was added to the solution. The resulting solution was stirred for 10 minutes at 0 ºC, then warmed for 20 minutes at room temperature and then refluxed for 18 hours 35 ºC. The presence of the initial reagents was followed by thin layer chromatography. TLC was eluted with dichloromethane:methanol (50:1). The solvent was evaporated under reduced pressure, the residue was washed with diethylether to eliminate the thiophosphate that had not reacted. The ethereal solution was discarded and the residue was dried under reduced pressure and then was treated with 20 ml of aqueous acid (HCl 0.1M) and extracted with dichloromethane (3 x 15 ml) to eliminate the remainder Hünig's base. The combined organic extracts were washed with water, dried with sodium sulphate, the solvent was evaporated under reduced pressure and the product recrystallized from to dichloromethane-hexane 1:1 to obtain 66 mg of *O*,*O*-diethyl (4-(5-(1-(dicyanomethylene)-indan-5-yl)pyrimidin-2-yl)piperazin-1-yl)phosphonothioate (81% yield) as a slightly yellow solid, mp: 191-193 ºC (decomp.). IR (KBr, cm^{-1}): 2978, 2917, 2900, 2851, 2219 (C≡N), 1594, 1515, 1439, 1363, 1314, 1262, 1134, 1049, 1019, 970, 799 (P-O), 726 (P=S). ^{1}H NMR ($CDCl_3$, 300 MHz) δ: 8.62 (s, 2H, H-C=N), 8.44 (d, *J* = 8.0 Hz, 1H, ArH), 7.58 (s, 1H, ArH), 7.54 (d, *J* = 8.0 Hz, 1H, ArH), 4.16-3.99 (m, 4H, 2×CH_2), 3.89 (t, *J* = 9.0 Hz, 4H, 2×CH_2), 3.40-3.31 (m, 4H, 2×CH_2), 3.28 (m, 4H, 2×CH_2), 1.33 (t, *J* = 7.0 Hz, 6H, 2×CH_3). ^{13}C NMR ($CDCl_3$, 75 MHz) δ: 178.3, 161.4, 156.4 (2×CH_{Ar}), 156.4, 142.6, 134.7, 127.1 (CH_{Ar}), 125.6 (CH_{Ar}), 122.4 (CH_{Ar}), 121.3, 113.8, 77.5, 63.4 (CH_2), 63.3 (CH_2), 45.3 (CH_2), 44.6 (CH_2), 44.5 (CH_2), 34.9 (CH_2), 29.9 (CH_2), 16.2 (CH_3), 16.11 (CH_3). MS (EI) m/z (%): 494 (M^+, 100), 495 (M^++1, 30), 496 (M^++2, 10), 479(20), 461 (22), 341 (32), 312 (81), 299 (92), 286 (60), 182 (72). HRMS (EI): calcd. for $C_{36}H_{44}N_8O_6S$: 494.1654 (M^+); found: 494.1660 (M^+).

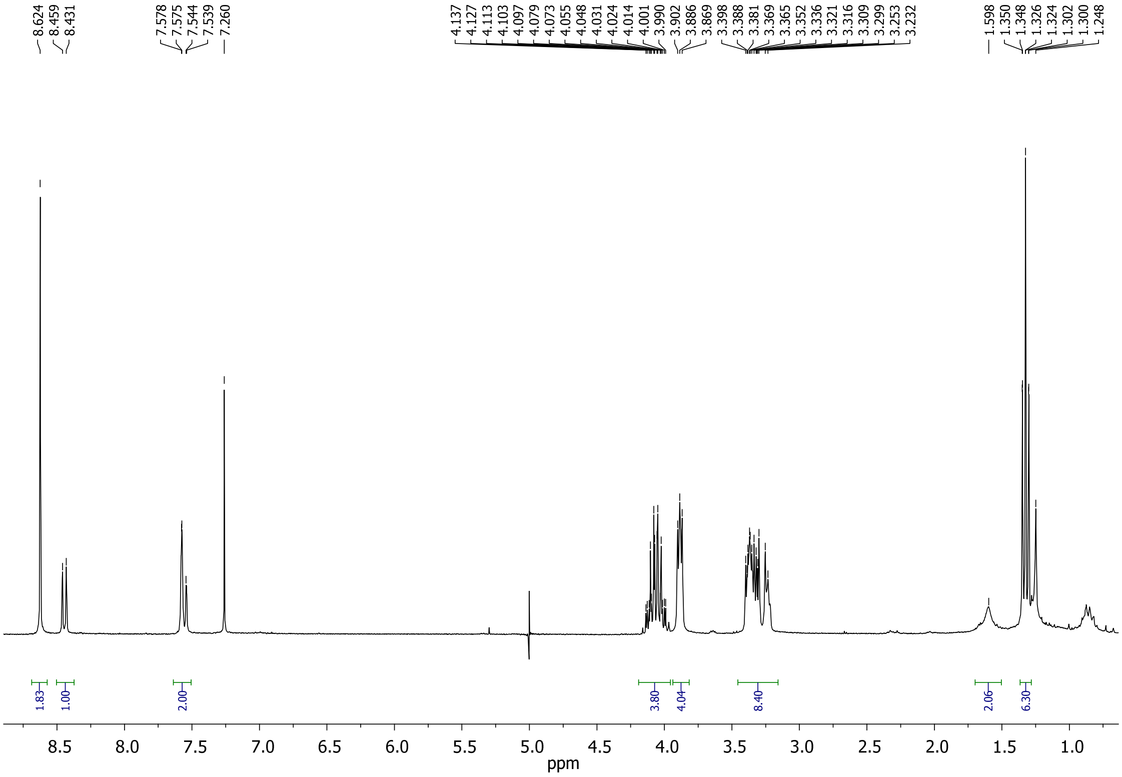

Figure S62: ^{1}H NMR ($CDCl_3$, 300 MHz) of *O,O*-diethyl-(4-(5-(1-(dicyanomethylene)indan-5-yl)pyrimidin-2-yl)piperazin-1-yl)phosphonothioate JG30

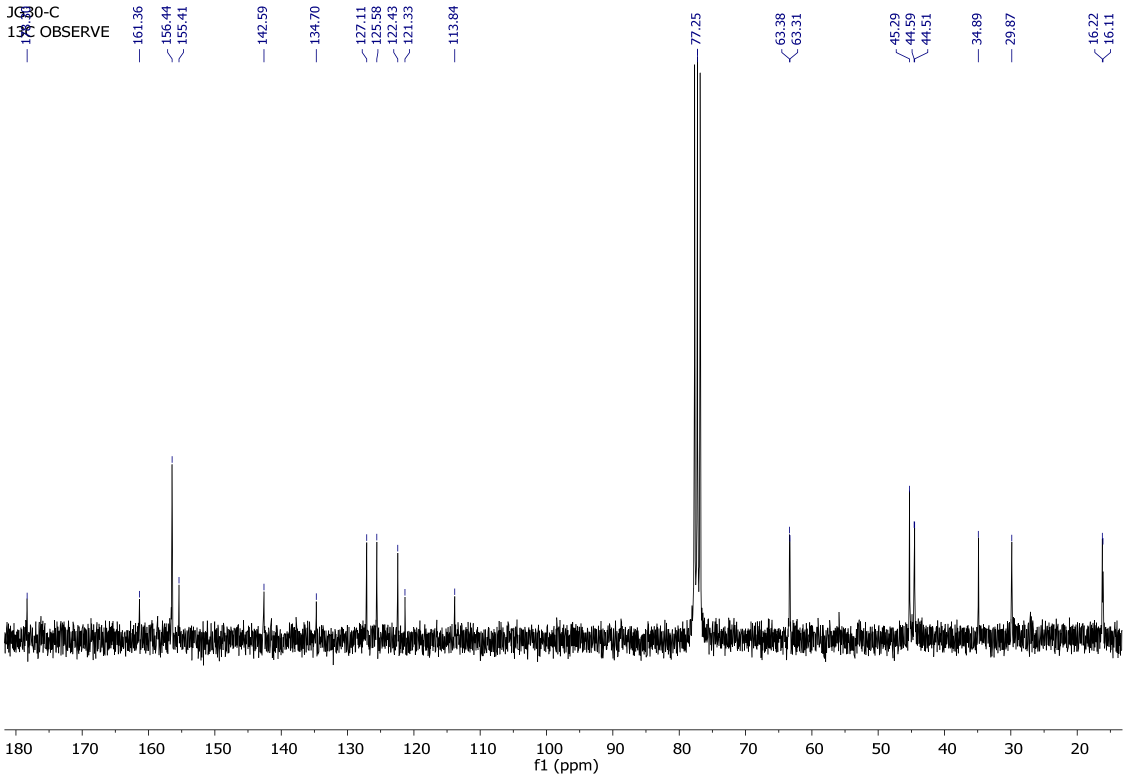

Figure S63: ^{13}C NMR ($CDCl_3$, 75 MHz) of *O,O*-diethyl-(4-(5-(1-(dicyanomethylene)indan-5-yl)pyrimidin-2-yl)piperazin-1-yl)phosphonothioate JG30

Solvatochromism

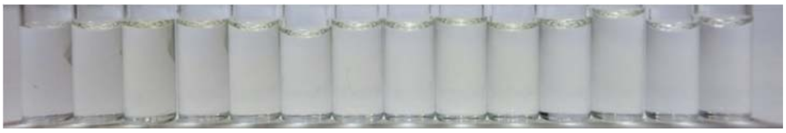

(1) (2) (3) (4) (5) (6) (7) (8) (9) (10) (11) (12) (13) (14)
(a)

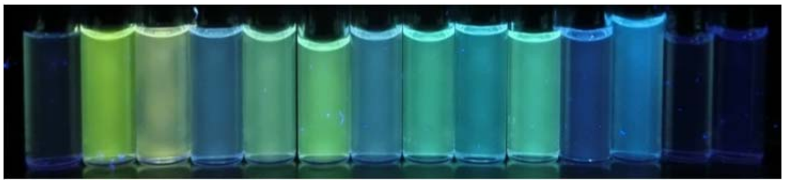

(1) (2) (3) (4) (5) (6) (7) (8) (9) (10) (11) (12) (13) (14)
(b)

Figure S64: JG30 5×10^{-5} M in (1) H_2O, (2) MeOH, (3) DMSO, (4) DMF, (5) MCN, (6) Acetone, (7) AcOEt, (8) THF (9) $CHCl_3$ (10) CH_2Cl_2 (11) Toluene (12) Et_2O (13) Hexane (14) Cyclohexane under (a) direct sunlight and (b) 366 nm light.

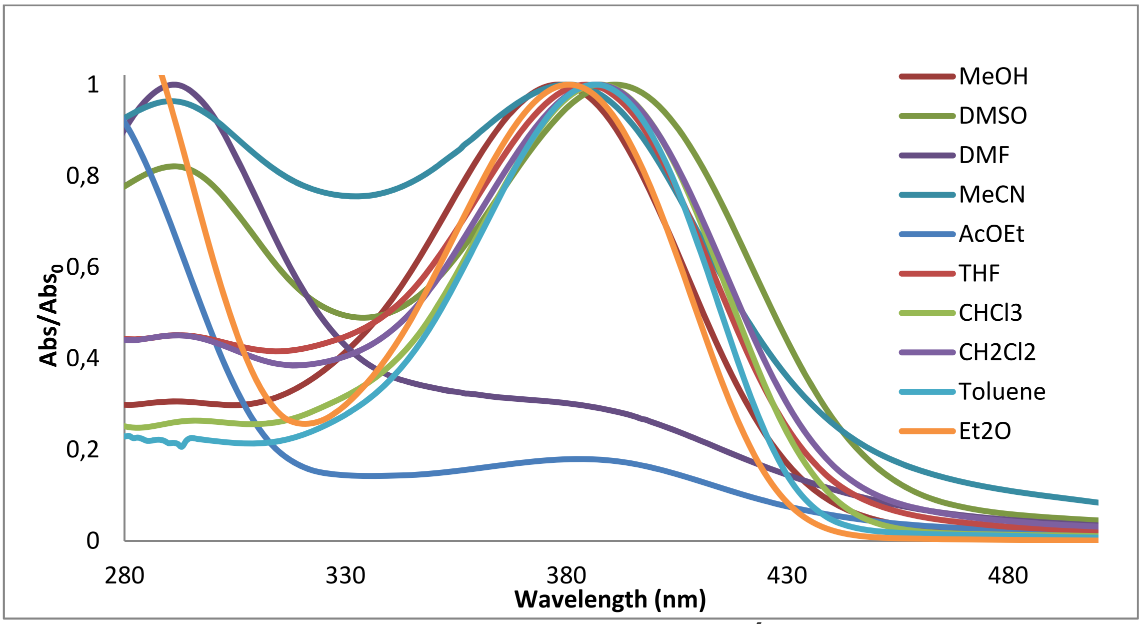

Figure S65: Normalized absorption spectra of **JG30** 5×10^{-5} M in different solvents.

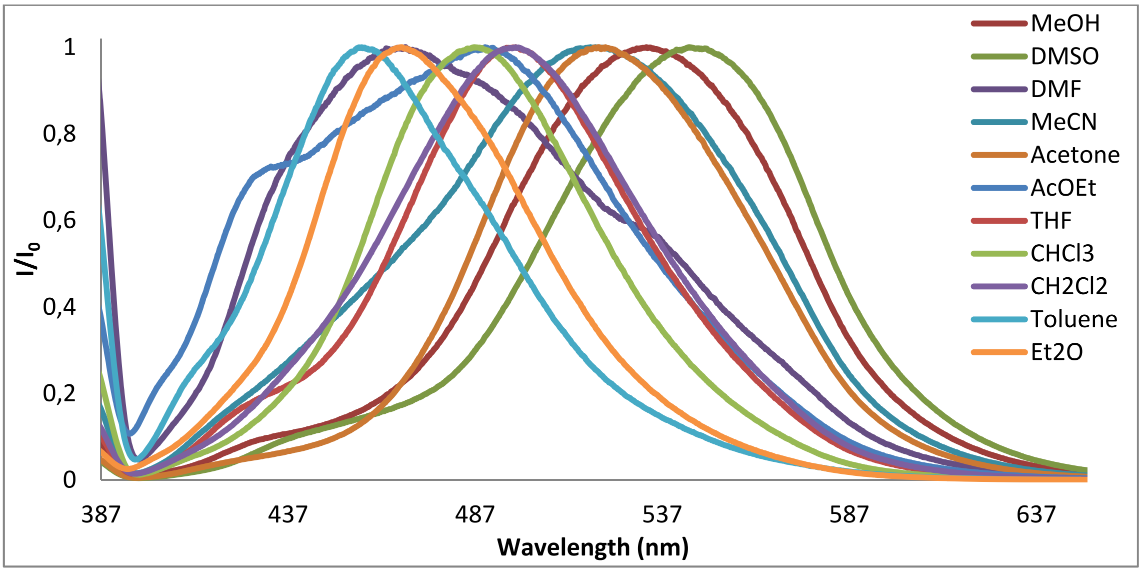

Figure S66: Normalized emission spectra of **JG30** 5×10^{-5} M in different solvents. $\lambda_{exc} = 366$ nm.

SYNTHESIS OF JG15:

Synthesis of *N*-(4-azidophenyl)-4-(5-(1-oxo-indan-5-yl)pyrimidin-2-yl)piperazin-1-carbothioamide JG10:

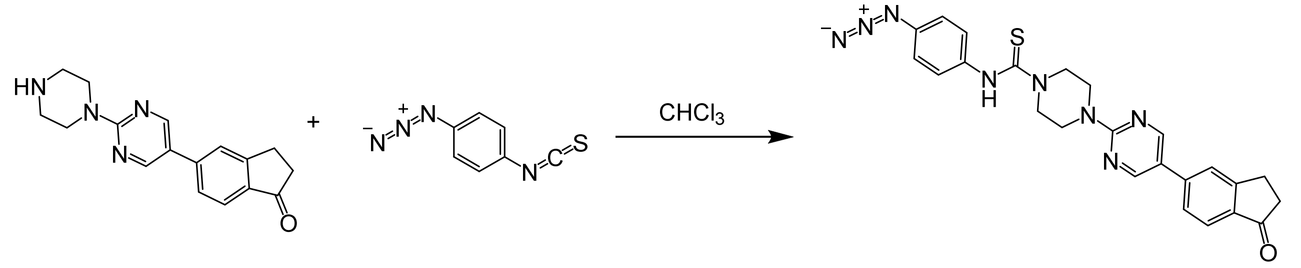

A 50 ml Schlenk, with a magnetic stirrer, was charged with 50 mg of 5-[4-(piperazine-1-yl)pyrimidin]indanone (0.17 mmol) and 20 ml of $CHCl_3$. The resulting solution stirred under nitrogen in an ice bath for five minutes and then 40.66 mg of 1-azide-4-isothiocianatebenzene (0.23 mmol) were added to the solution. The solution was stirred for 20 minutes at 0 ºC, then for 21 hours at room temperature and then refluxed for 30 minutes at 35 ^{o}C. The presence of the initial reagents was followed by thin layer chromatography. TLC was eluted with dichloromethane:methanol (50:1). The solvent was evaporated and the product was purified under flash column chromatography from dichloromethane to dichloromethane:methanol 50:1 v/v to obtain 55.4 mg of *N*-(4-azidophenyl)-4-(5-(1-oxoindan-5-yl)pyrimidin-2-yl)piperazin-1-carbothioamide (56% yield) as a white solid, mp: 188-190 ^{o}C (decomp.). IR (KBr, cm^{-1}): 3359 (N-H), 3022, 2921, 2851, 2110 (N^{-}=N^{+}=N-), 1697 (C=O), 1597, 1503, 1453, 1420, 1355, 1307, 1259, 1224. ^{1}H NMR (DMSO-d_6, 400 MHz) δ: 9.43 (s, 1H, N-H), 8.86 (s, 2H, H-C=N), 7.89 (s, ArH), 7.73 (d, *J* = 8.7 Hz, 1H, ArH), 7.68 (d, *J* = 8.1 Hz, 1H, ArH), 7.36 (d, *J* = 8.7 Hz, 2H, ArH), 7.06 (d, *J* = 8.7 Hz, 2H, ArH), 4.06 (m, 4H, 2×CH_2) 3.93 (m, 4H, 2×CH_2), 3.14 (t, *J* =5.8 Hz, 2H, CH_2), 2.66 (t, *J* = 5.8 Hz, 2H, CH_2). ^{13}C NMR (DMSO-d_6, 100 MHz) δ: 205.9 (C=O), 181.4 (C=S), 160.5, 156.4 (2×CH_{Ar}), 156.2, 141.1, 138.1, 135.3, 135.2, 127.0 (2×CH_{Ar}), 124.7 (CH_{Ar}), 123.6 (CH_{Ar}), 123.3 (CH_{Ar}), 121.2, 118.7 (2×CH_{Ar}), 47.5 (CH_2), 43.0 (CH_2), 36.1 (CH_2), 25.5 (CH_2). MS (MALDI) m/z (%): 471 (M^++1, 21), 443 (100), 411 (45), 393 (19), 337 (95), 295 (43). HRMS (MALDI): calcd. for $C_{24}H_{23}N_8OS$: 471.1710 (M^++1); found: 471.1803 (M^++1).

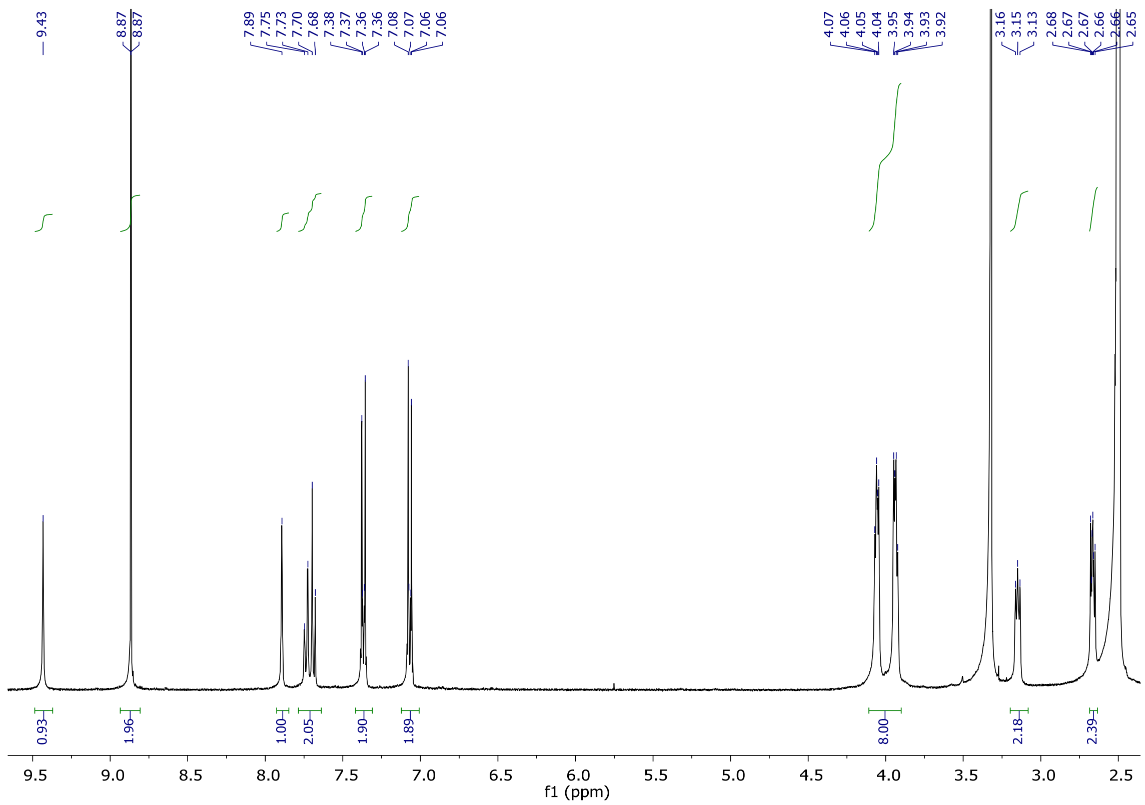

Figure S67: ^{1}H NMR ($CDCl_3$, 300 MHz) of *N*-(4-azidophenyl)-4-(5-(1-oxo-indan-5-yl)pyrimidin-2-yl)piperazin-1-carbothioamide JG10

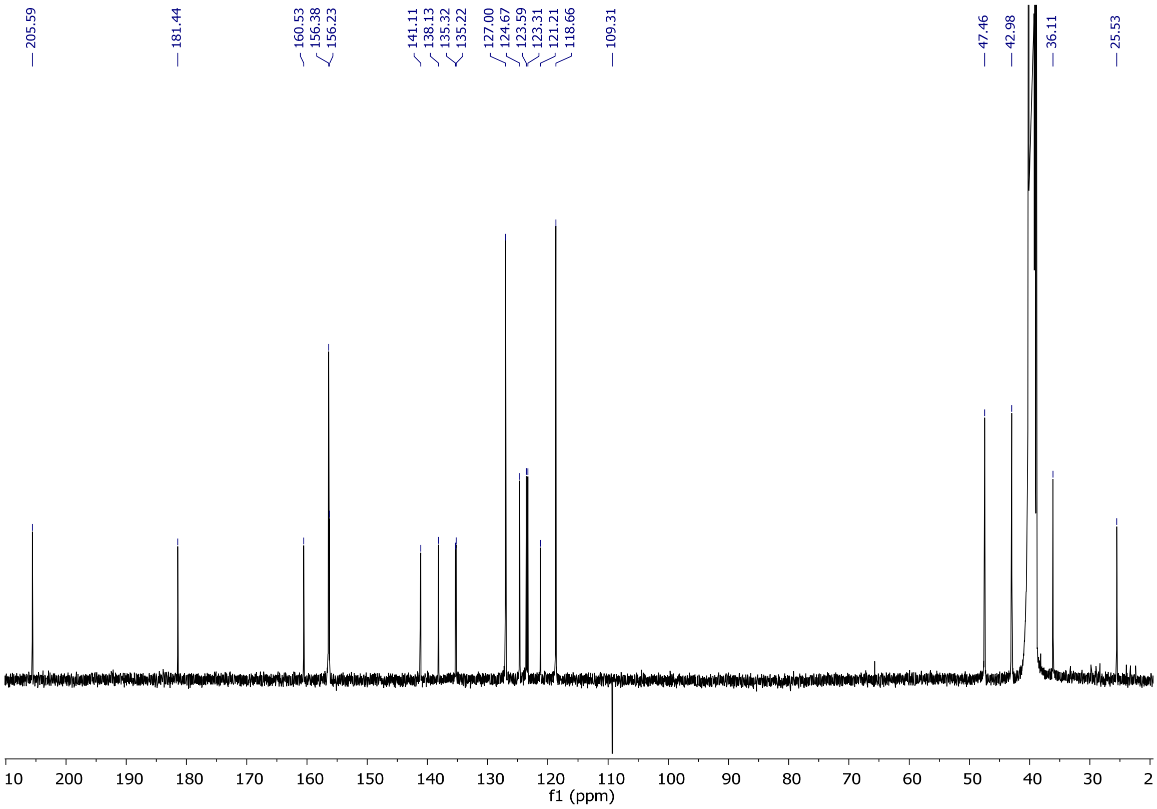

Figure S68: ^{13}C NMR ($CDCl_3$, 75 MHz) of *N*-(4-azidophenyl)-4-(5-(1-oxo-indan-5-yl)pyrimidin-2-yl)piperazin-1-carbothioamide JG10

Synthesis of *N*-(4-(4-(2,5,8,11,14-pentaoxapentadecyl)-1*H*-1,2,3-triazol-1-yl)phenyl)-4-(5-(1-oxoindan-5-yl)pyrimidin-2-yl)piperazin-1-carbothioamide JG15

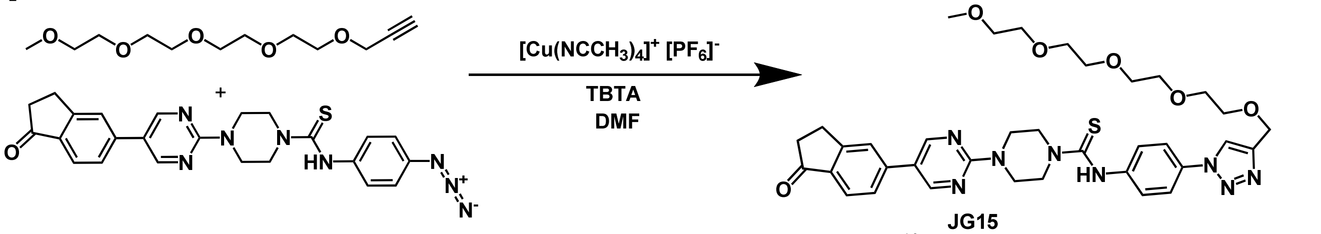

A 100 ml schlenk was charged with 9.4 mg de 2,5,8,11,14-pentaoxaheptadec-16-yne[10] and 20 ml of DMF under nitrogen atmosphere. Then 20 mg of *N*-(4-azidophenyl)-4-(5-(1-oxoindan-5-yl)pyrimidin-2-yl)piperazin-1-carbothioamide, 5 mg of tetrakis(acetonitrile)copper(I) hexafluorophosphate and 5 mg of tris[(1-benzyl-1*H*-1,2,3-triazol-4-yl)methyl]amine were added and the mixture stirred for 1.5 hours at room temperature and for 10 hours at 30°C. The mixture was poured to water (40 ml) and extracted with dichloromethane (3 x 60ml), the combined organic extracts were dried with sodium sulphate, filtered and the solvent was evaporated under reduced pressure. The product was purified by column chromatography from dichloromethane to dichloromethane/methanol 50% v/v. The obtained product was washed up with hexane and pentane, obtaining solid JG15, 20 mg (65% yield), mp: 84-86 °C. IR (KBr, cm^{-1}): 3463 (N-H), 3138, 2918, 2851, 1699 (C=O), 1595, 1514, 1442, 1363, 1307, 1256, 1229, 1103. ^{1}H ($CDCl_3$, 400 MHz) δ: 9.92 (s, 1H, N-H), 8.55 (s, 2H, H-C=N), 7.98 (s, 1H, ArH), 7.79 (d, ^2J = 8 Hz, 1H, ArH), 7.63-7.44 (m, 3H, ArH), 7.45 (d, ^2J = 8 Hz, 1H, ArH), 7.28 (d, J = 8 Hz, 2H, ArH), 4.71 (s, 2H, CH_2), 3.96-3.84 (m, 8H, 4×CH_2), 3.72-3.59 (m, 14H, 7×CH_2), 3.50 (m, 2H, CH_2), 3.33 (s, 3H, CH_3), 3.17 (t, J =5.5 Hz, 2H, CH_2), 2.71 (t, J = 5.5 Hz, 2H, CH_2). ^{13}C NMR ($CDCl_3$, 100MHz) δ: 206.3 (C=O), 180.9 (C=S), 160.9, 156.4 (2×CH_{Ar}), 156.3, 146.0, 141.8, 139.9, 136.2, 133.7, 125.3 (2×CH_{Ar}), 124.7 (CH_{Ar}), 123.6 (CH_{Ar}), 122.9 (CH_{Ar}), 122.9, 121.6 (CH_{Ar}), 71.9 (CH_2), 70.6-70.5 (6×CH_2), 70.0 (CH_2), 64.0 (CH_2), 59.2 (CH_3), 50.4 (CH_2), 43.2 (CH_2), 36.6 (CH_2), 26.0 (CH_2). MS (LSIMS) m/z (%): 739 ([M^++^{23}Na]$^+$, 95), 695 (63), 679 (45), 613 (100), 445. HRMS (LSIMS): calcd. for $C_{36}H_{44}N_8O_6SNa$: 739.3002 (M^++^{23}Na); found: 739.2987 ([M^++^{23}Na]$^+$).

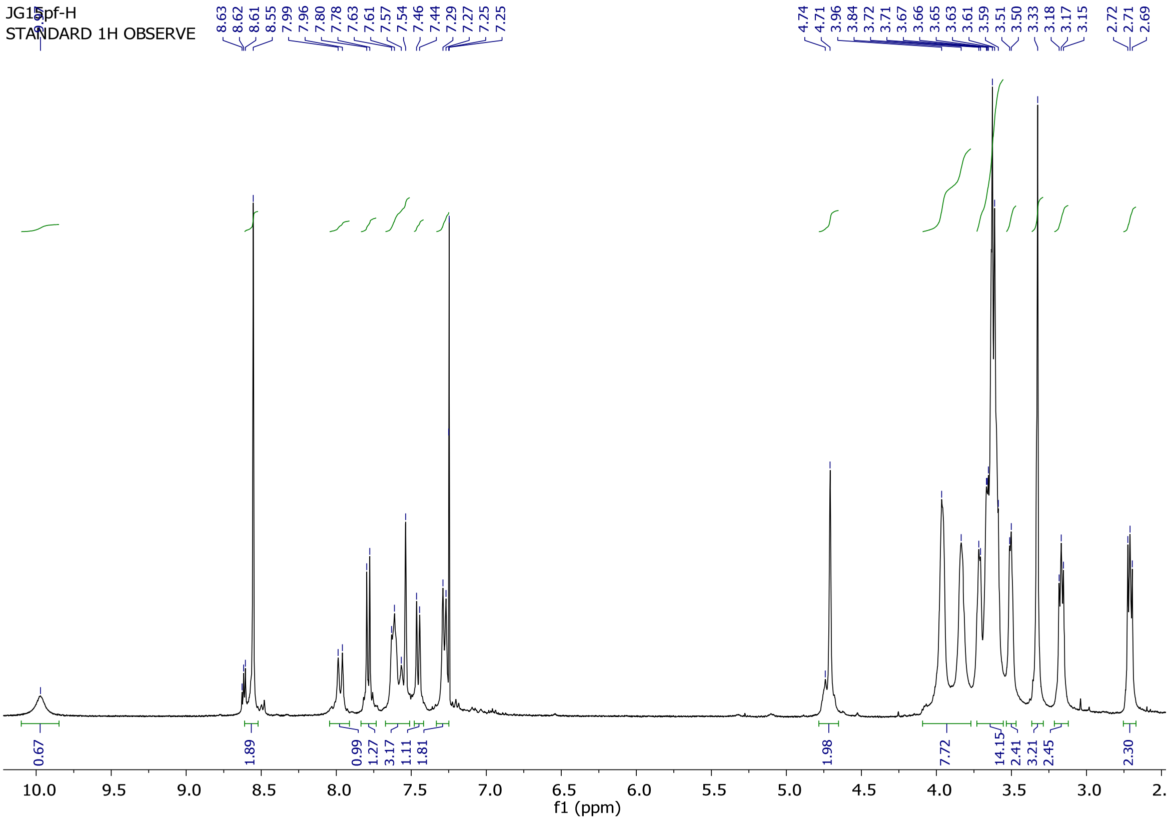

Figure S69: ^{1}H NMR ($CDCl_3$, 300 MHz) of *N*-(4-(4-(2,5,8,11,14-pentaoxapentadecyl)-1*H*-1,2,3-triazol-1-yl)phenyl)-4-(5-(1-oxoindan-5-yl)pyrimidin-2-yl)piperazin-1-carbothioamide JG15

[10] B. A. Scates, B. L. Lashbrook, B. C. Chastain, K. Tominaga, B. T. Elliott, N. J. Theising, T. A. Baker and R. W. Fitch, *Bioorg. Med. Chem.*, 2008, **16**, 10295.

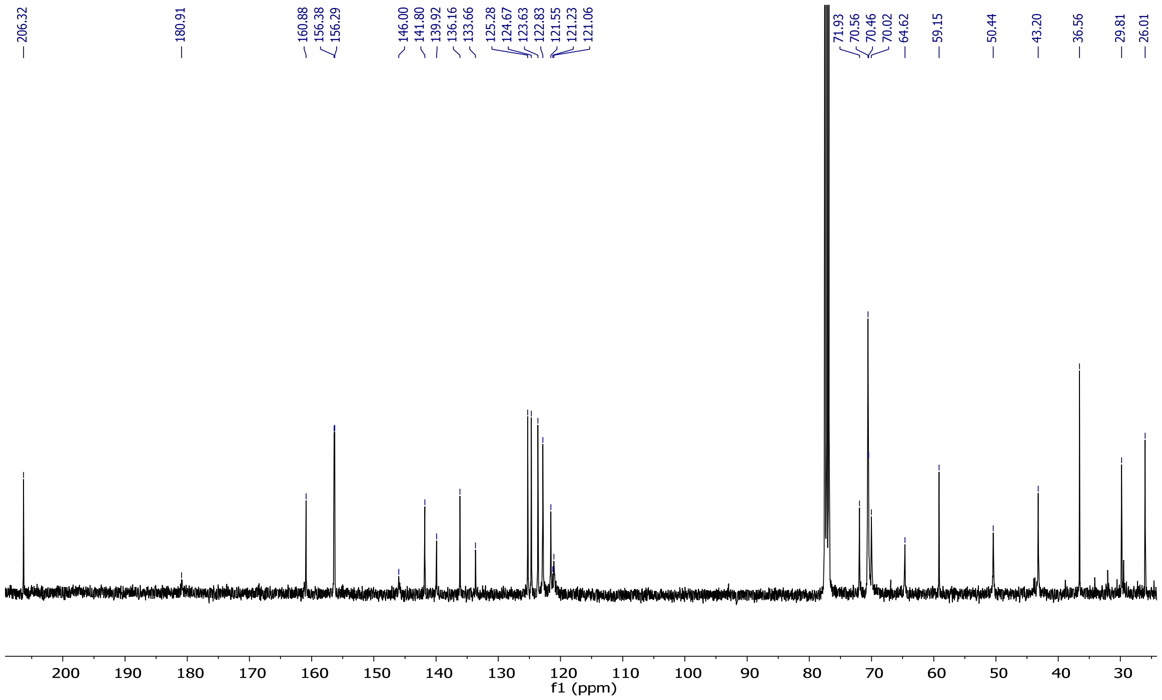

Figure S70: ^{13}C NMR ($CDCl_3$, 75 MHz) of *N*-(4-(4-(2,5,8,11,14-pentaoxapentadecyl)-1*H*-1,2,3-triazol-1-yl)phenyl)-4-(5-(1-oxoindan-5-yl)pyrimidin-2-yl)piperazin-1-carbothioamide JG15

Solvatochromism

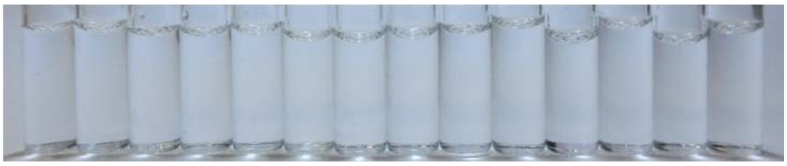

(1) (2) (3) (4) (5) (6) (7) (8) (9) (10) (11) (12) (13) (14)

(a)

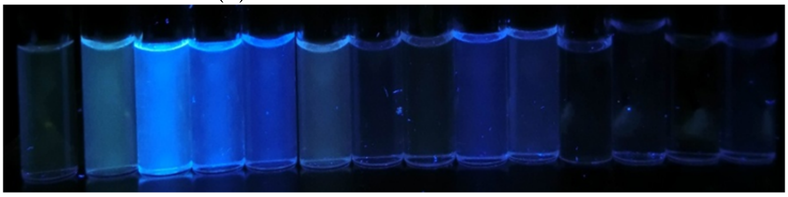

(1) (2) (3) (4) (5) (6) (7) (8) (9) (10) (11) (12) (13) (14)

(b)

Figure S71: JG15 5×10^{-5} M in (1) H_2O, (2) MeOH, (3) DMSO, (4) DMF, (5) MCN, (6) Acetone, (7) AcOEt, (8) THF (9) $CHCl_3$ (10) CH_2Cl_2 (11) Toluene (12) Et_2O (13) Hexane (14) Cyclohexane under (a) direct sunlight and (b) 366 nm light.

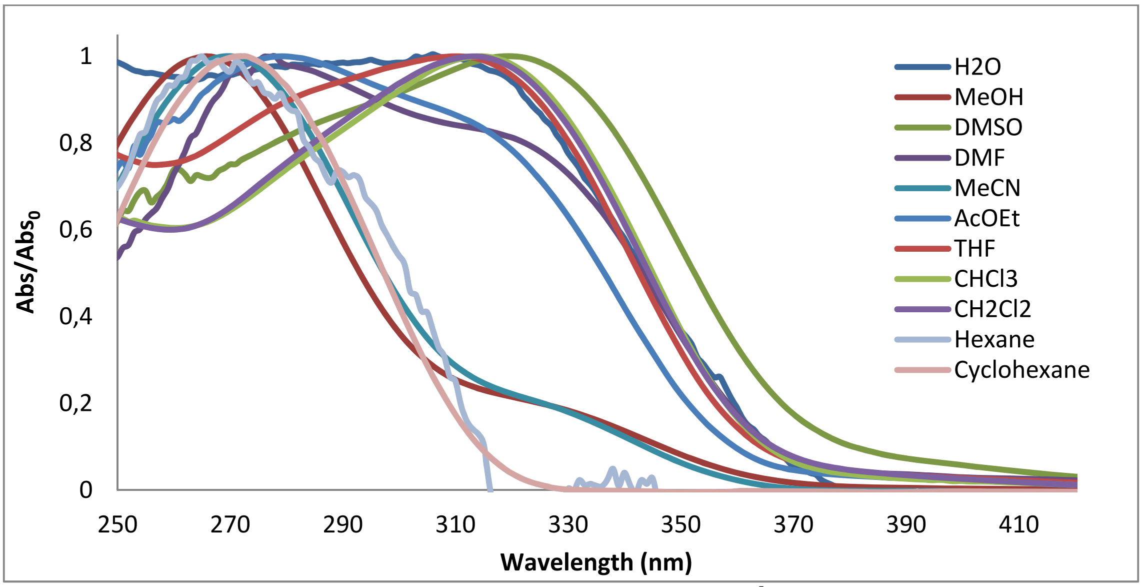

Figure S72: Normalized absorption spectra of **JG15** 5×10^{-5} M in different solvents.

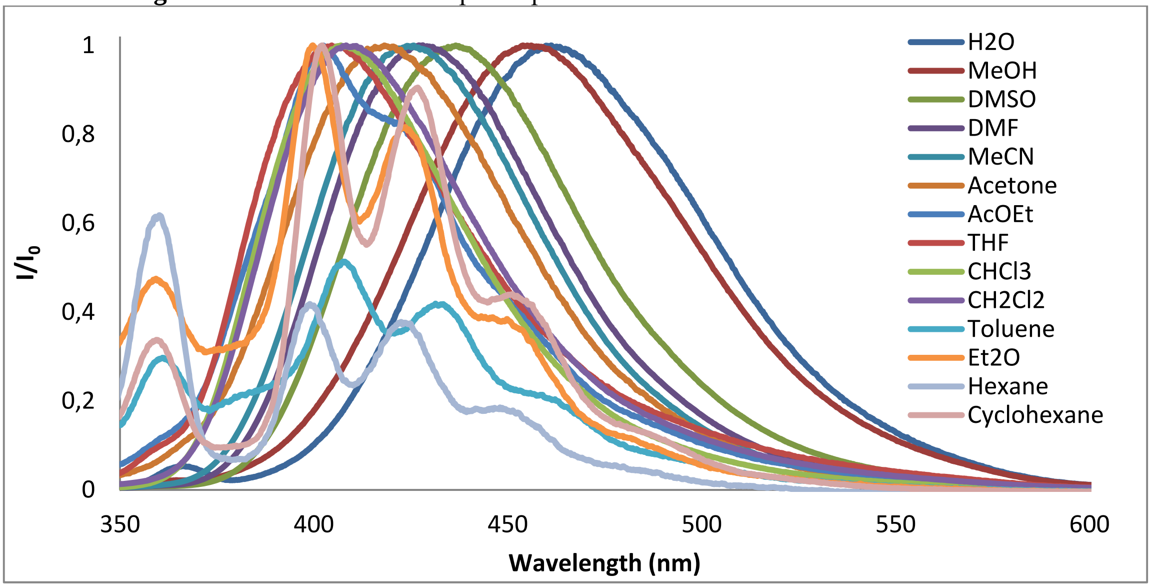

Figure S73: Normalized emission spectra of **JG15** 5×10^{-5} M in different solvents. λ_{exc} = 366 nm.

SYNTHESIS OF JG45:

Synthesis of 3,4,5-tris(methyltetraethyleneoxy)benzyl propargyl ether JG44:

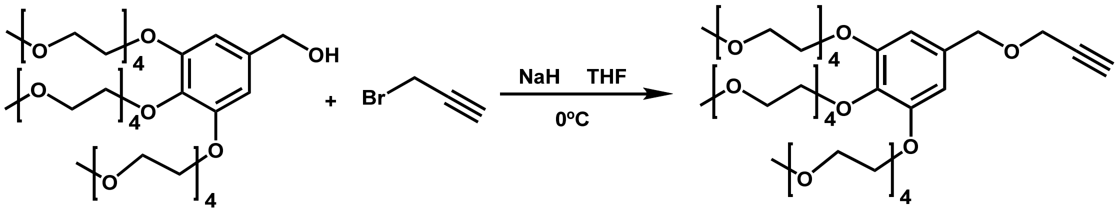

A 100 ml Schlenk with a magnetic stirrer was charged with 70 ml of dry THF under nitrogen atmosphere. Then 2000 mg of 3,4,5-tris(methyltetraethyleneoxy)benzyl alcohol[11] (2.75 mmol) and 80 mg of NaH, 80 % in mineral oil (3.3 mmol), were added at 0 ºC (ice bath) and stirred for five minutes. Then, 337 µl of propargyl bromide (3.03 mmol) were added and stirred for 45 minutes at 0ºC, then the solution was stirred at room temperature for 4 hours, and then refluxed for 17 hours. Then the solution was cooled at room temperature and neutralized to pH = 6 with HCl solution in water (1 M), appearing a white precipitate in the yellow solution. The product was purified by flash column chromatography from CH_2Cl_2 to CH_2Cl_2:MeOH 50:3 v/v, to obtain 1.033 g of 3,4,5-tris(methyltetraethyleneoxy)benzyl propargyl ether as a yellow-orange liquid (49 % yield). IR (KBr, cm^{-1}): 3463 (N-H), 3241 (C≡CH), 2914, 2872, 2851, 2110 (C≡C), 1732, 1650 (C=O), 1592, 1504, 1436, 1358, 1327, 1200. ^1H NMR ($CDCl_3$, 300 MHz) δ: 6.54 (s, 2H, ArH), 4.45 (d, J =6.0 Hz, 2H, CH_2), 4.10 (m, 8H, 4×CH_2), 3.82-3.48 (m, 40H, 20×CH_2), 3.33 (s, 9H, 3×CH_3), 2.45 (t, J = 2Hz, 1H, C≡CH). ^{13}C NMR ($CDCl_3$, 75 MHz) δ: 152.6 (CH_{Ar}), 137.9, 132.8, 107.5 (CH_{Ar}), 79.6 (C≡C), 74.8 (C≡C), 72.3 (CH_2), 71.9 $(CH_2)_n$, 71.5 (CH_2), 70.8-70.5 $(CH_2)_n$, 69.7 (CH_2), 68.8 (CH_2), 59.0 (3×CH_3), 57.0 (CH_2). MS (LSIMS) m/z (%): 764 (M^+, 100), 765 (M^++1, 65), 766 (M^++2, 35), 787 (M^++Na^+). HRMS (LSIMS): calcd. for $C_{37}H_{64}O_{16}$: 764.4194 (M^+); found: 764.4215 (M^+).

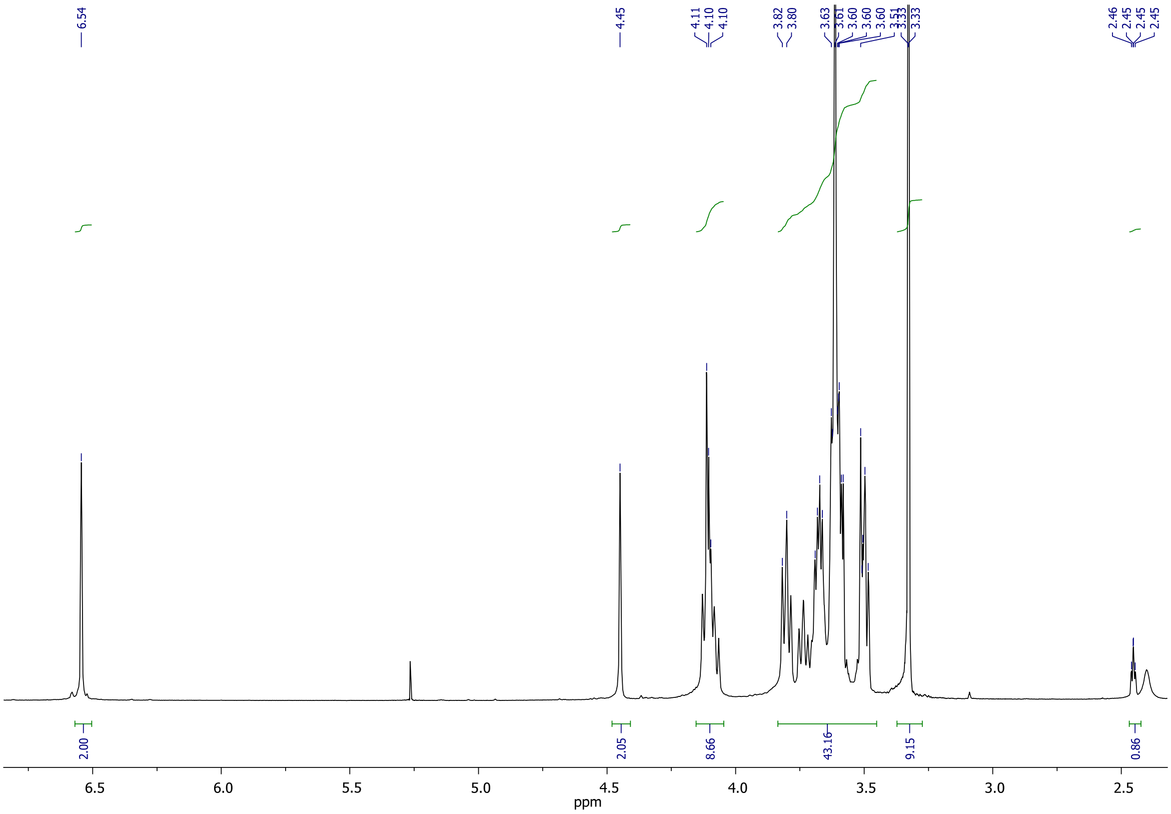

Figure S74: ^1H NMR ($CDCl_3$, 300 MHz) of 3,4,5-tris(methyltetraethyleneoxy)benzyl propargyl ether JG44

[11] O. Middel, W. Verboom and D. N. Reinhoudt, *Eur. J. Org. Chem.,* 2002, 2587.

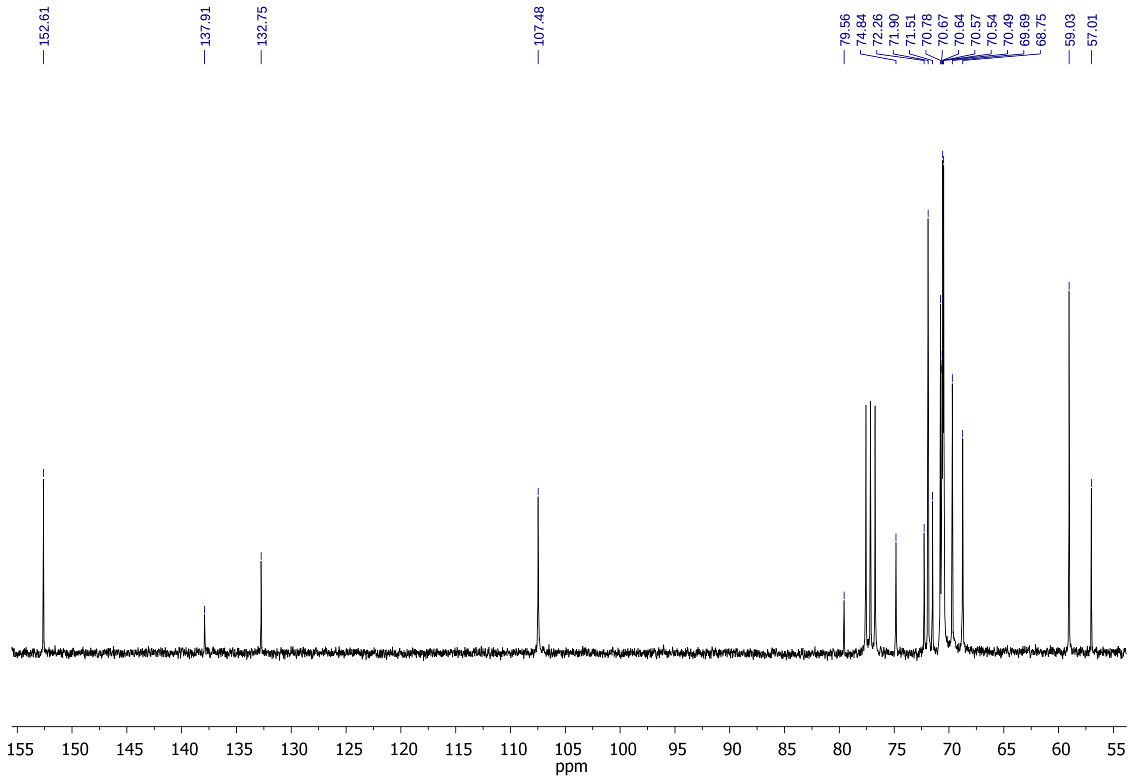

Figure S75: ^{13}C NMR ($CDCl_3$, 75 MHz) of 3,4,5-tris(methyltetraethyleneoxy)benzyl propargyl ether JG44

Synthesis of 4-(5-(1-oxo-2,3-dihydro-1*H*-inden-5-yl)pyrimidin-2-yl)-*N*-(4-(4-(((3,4,5-tris((2,5,8,11-tetraoxatridecan-13-yl)oxy)benzyl)oxy)methyl)-1*H*-1,2,3-triazol-1-yl)phenyl)piperazine-1-carbothioamide JG45:

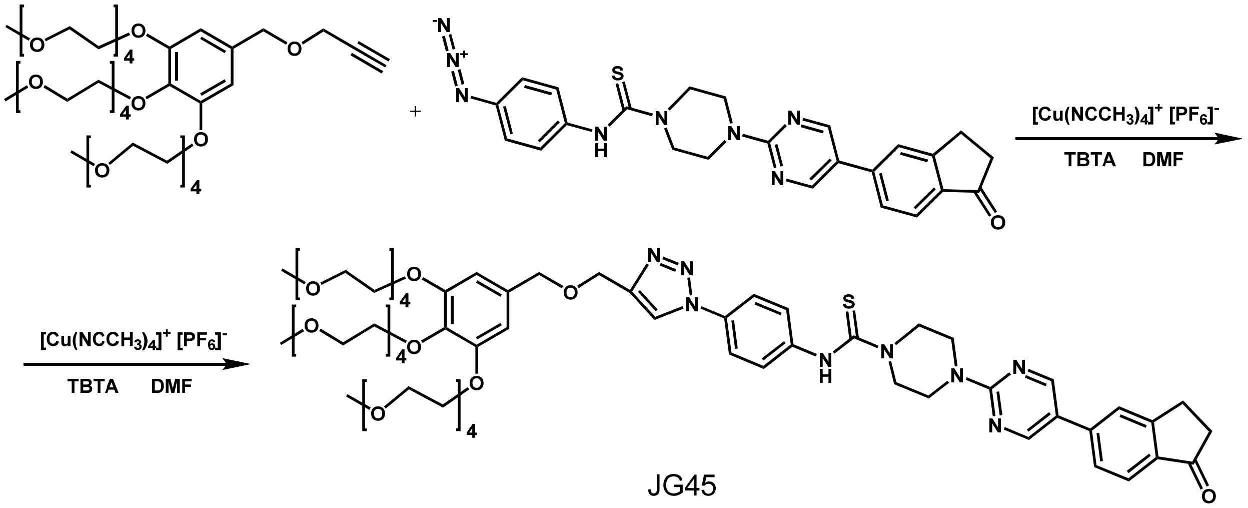

A 100 ml schlenk, under nitrogen atmosphere, was charged with 114.1 mg of 3,4,5-tris(methyltetraethyleneoxy)benzyl propargyl ether JG44 and 60 of DMF. Then, 5 mg of tetrakis(acetonitrile)copper(I) hexafluorophosphate and 5 mg of tris[(1-benzyl-1*H*-1,2,3-triazol-4-yl)methyl]amine and 70 mg of *N*-(4-azidophenyl)-4-(5-(1-oxo-indan-5-yl)pyrimidin-2-yl)piperazin-1-carbothioamide were added successively under stirring. The mixture was stirred at room temperature for an hour and then for 22 hours at 30°C. After work-up, the product was purified by column chromatography from CH_2Cl_2:MeOH 98:2 to CH_2Cl_2:MeOH 90:10. The product was washed up with hexane and pentane, to obtain 90 mg of JG45 (48.8 % yield) as a ligh brown solid, mp: 110-113 °C. IR (KBr, cm^{-1}): 3448 (N-H), 3146-3100, 2914, 2875, 1695 (C=O), 1595, 1507, 1454, 1433, 1357, 1305, 1241, 1223, 1110 (C=S). ^{1}H NMR ($CDCl_3$, 300 MHz) δ: 8.60 (s, 2H, H-C=N), 8.46 (s, 1H, N-H), 7.96 (s, 1H, ArH), 7.79 (d, ^{2}J = 8 Hz, 1H, ArH), 7.65 (d, ^{2}J = 8.6 Hz, 2H, ArH), 7.56 (s, ArH), 7.48 (d, ^{2}J = 8 Hz, 1H, ArH), 7.45 (d, J = 8.6 Hz, 2H, ArH), 6.58 (s, 2H, ArH), 4.71 (s, 2H, CH_2), 4.51 (s, 2H, CH_2), 4.13-3.80 (m, 20H, 10×CH_2), 3.70-3.51 (m, 36H, 18×CH_2), 3.33 (m, 9H, 3×CH_3), 3.18 (t, J = 5.5 Hz, 2H, CH_2), 2.72 (t, J = 5.5 Hz, 2H, CH_2). ^{13}C NMR ($CDCl_3$, 75MHz) δ: 206.5 (C=O), 182.4 (C=S), 160.9, 156.4 (2×CH_{Ar}), 156.3, 152.6, 152.5, 145.8, 141.9, 140.6, 137.5, 136.0, 133.7, 133.6, 125.2

(CH$_{Ar}$), 125.1 (CH$_{Ar}$), 124.6 (CH$_{Ar}$), 123.6 (CH$_{Ar}$), 122.6, 121.1 (CH$_{Ar}$), 107.3 (CH$_{Ar}$), 72.7-68.7 (12×CH$_2$), 63.6 (CH$_2$), 59.1 (3×CH$_3$), 48.8 (CH$_2$), 43.2 (CH$_2$), 36.6 (CH$_2$), 26.0 (CH$_2$). MS (MALDI) m/z (%): 1235 (M^++1, 5), 1214 (8), 1197.6 (11), 1158 (90), 1135 (41), 1113 (100), 1091 (45). HRMS (MALDI): calcd. for $C_{61}H_{87}N_8O_{17}S$: 1235.5910 (M^++1); found: 1235.5945 (M^++1).

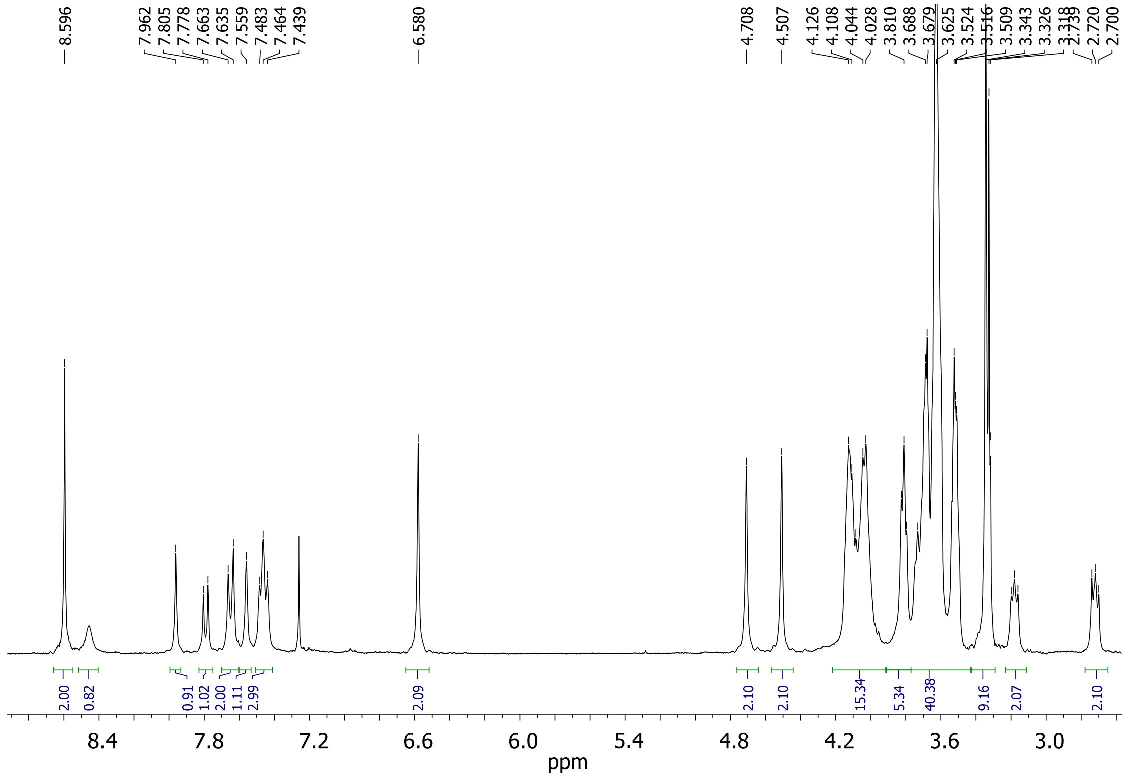

Figure S76: ^{1}H NMR (CDCl$_3$, 300 MHz) of 4-(5-(1-oxo-2,3-dihydro-1*H*-inden-5-yl)pyrimidin-2-yl)-*N*-(4-(4-(((3,4,5-tris((2,5,8,11-tetraoxatridecan-13-yl)oxy)benzyl)oxy)methyl)-1*H*-1,2,3-triazol-1-yl)phenyl)piperazine-1-carbothioamide JG45

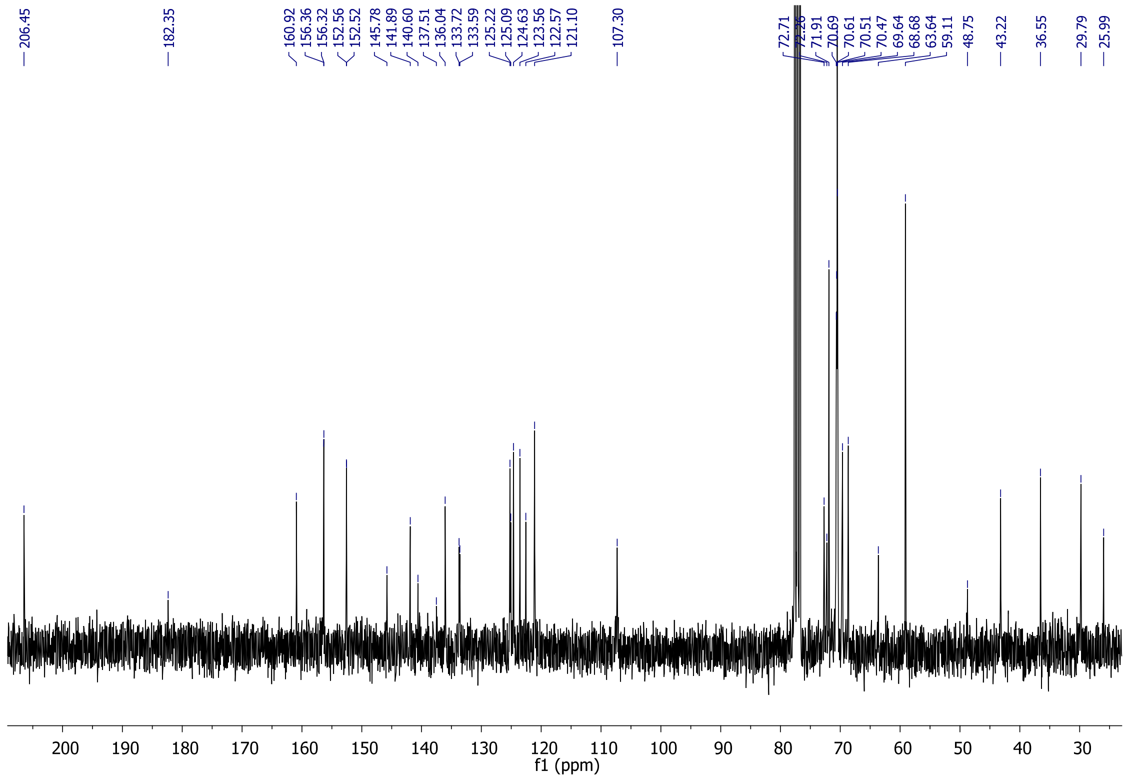

Figure S77: ^{13}C NMR (CDCl$_3$, 75 MHz) of 4-(5-(1-oxo-2,3-dihydro-1*H*-inden-5-yl)pyrimidin-2-yl)-*N*-(4-(4-(((3,4,5-tris((2,5,8,11-tetraoxatridecan-13-yl)oxy)benzyl)oxy)methyl)-1*H*-1,2,3-triazol-1-yl)phenyl)piperazine-1-carbothioamide JG45

Solvatochromism

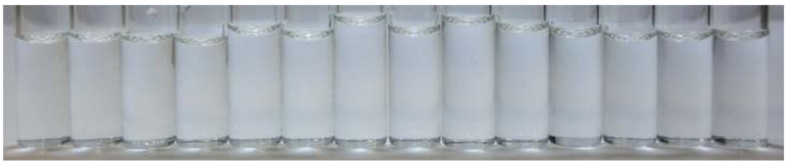

(1) (2) (3) (4) (5) (6) (7) (8) (9) (10) (11) (12) (13) (14)

(a)

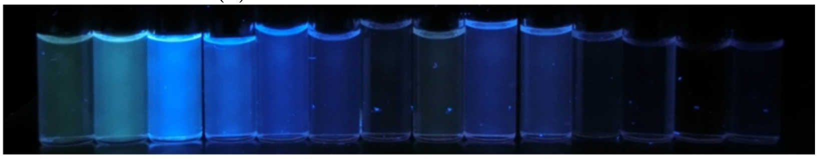

(1) (2) (3) (4) (5) (6) (7) (8) (9) (10) (11) (12) (13) (14)

(b)

Figure S78: JG45 5×10^{-5} M in (1) H_2O, (2) MeOH, (3) DMSO, (4) DMF, (5) MCN, (6) Acetone, (7) AcOEt, (8) THF (9) $CHCl_3$ (10) CH_2Cl_2 (11) Toluene (12) Et_2O (13) Hexane (14) Cyclohexane under (a) direct sunlight and (b) 366 nm light.

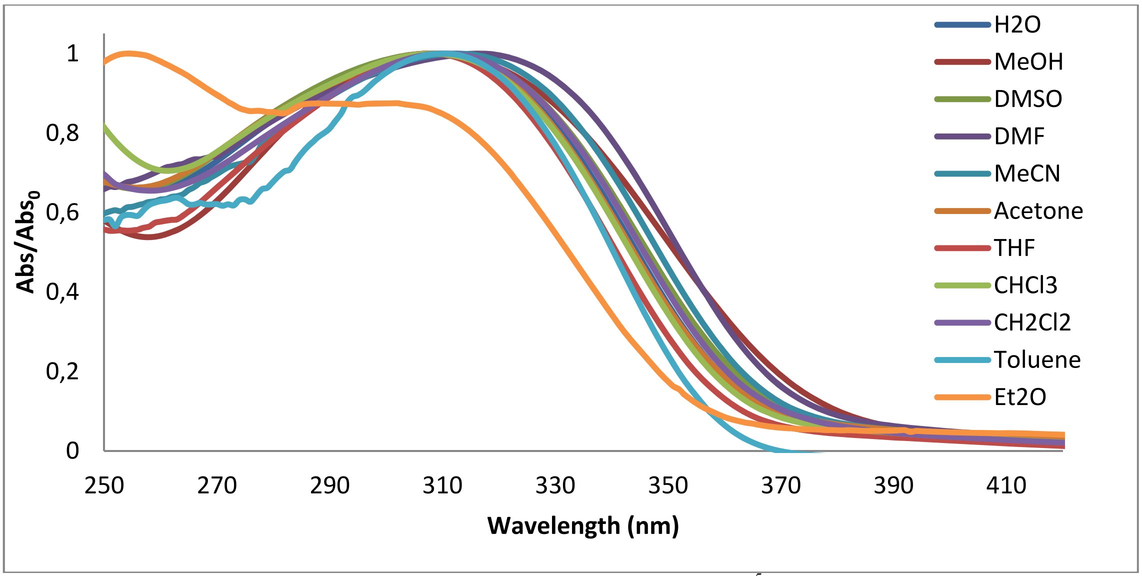

Figure S79: Normalized absorption spectra of **JG45** 5×10^{-5} M in different solvents.

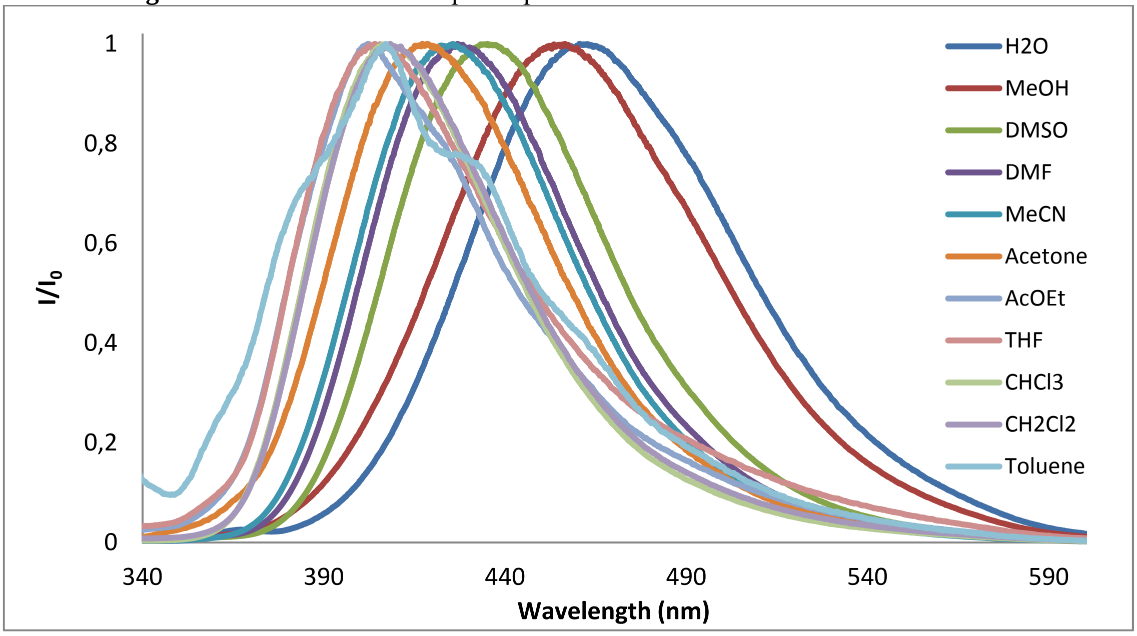

Figure S80: Normalized emission spectra of **JG45** 5×10^{-5} M in different solvents. $\lambda_{exc} = 366$ nm.

Qualitative experiments:

The changes in fluorescence in presence of Hg^{2+} and $MeHg^{+}$ were tested (8 equivalents):

THF Acetone MeCN

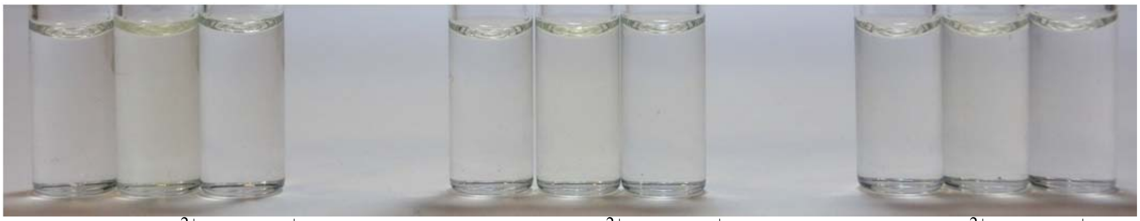

Water – Hg^{2+} - $MeHg^{+}$ Water – Hg^{2+} - $MeHg^{+}$ Water – Hg^{2+} - $MeHg^{+}$

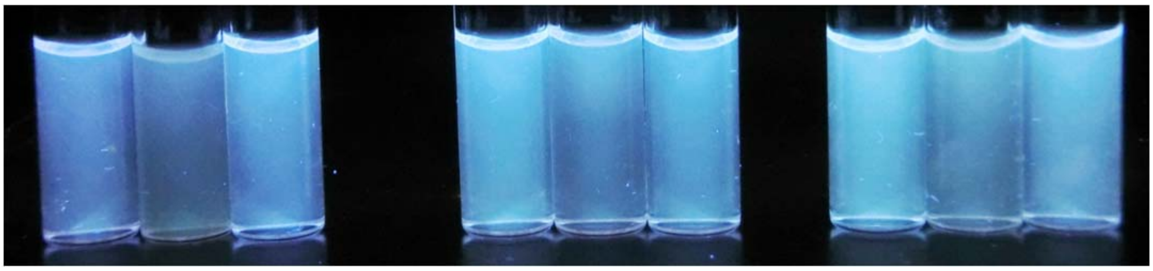

Figure S81: The fluorescence decreases in presence of excess Hg^{2+}. In the case of $MeHg^{+}$ fluorescence increases in the presence of excess $MeHg^{+}$.

SYNTHESIS OF JG47:

Synthesis of 2-(2-methoxypolyethoxy)ethyl 3-(1-(4-(4-(5-(1-oxo-indan-5-yl)pyrimidin-2-yl)piperazine-1-carbothioamido)phenyl)-1H-1,2,3-triazol-4-yl)propanoate JG47:

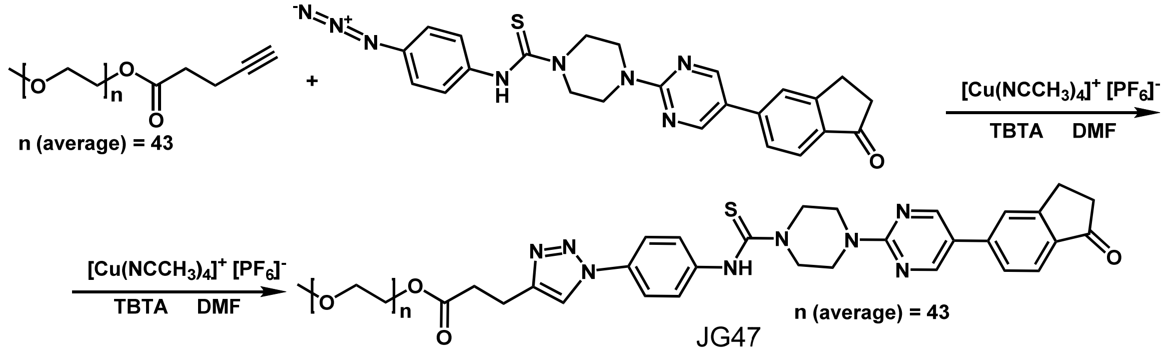

A 100 ml schlenk was charged with 256 mg of commercial polyethylenglycol monomethylether pent-4-ynoate (average MW 2000) and 60 ml of DMF under nitrogen atmosphere. Then, 5 mg of tetrakis(acetonitrile)copper(I) hexafluorophosphate and 5 mg of tris[(1-benzyl-1*H*-1,2,3-triazol-4-yl)methyl]amine and 60 mg of *N*-(4-azidophenyl)-4-(5-(1-oxo-indan-5-yl)pyrimidin-2-yl)piperazin-1-carbothioamide were added successively under stirring. The mixture was stirred at room temperature for 1.5 hours and then for 22 hours at 30°C. After work-up the product was purified by column chromatography from CH_2Cl_2:MeOH 98:2 to CH_2Cl_2:MeOH 88:12 v/v. The obtained product was washed up with hexane and pentane, to obtain JG47, 201 mg (63.5 % yield), as a light yellow solid, mp: 114-116 °C. IR (KBr, cm^{-1}): 3445 (N-H), 3146-3100, 2914, 2875, 1695 (C=O), 1595, 1507, 1454, 1433, 1357, 1305, 1241, 1223, 1110 (C=S). ^{1}H NMR ($CDCl_3$, 300 MHz) δ: 8.63 (s, 2H, H-C=N), 8.28 (s, 1H, N-H), 7.86 (s, 1H, ArH), 7.83 (d, ^2J = 8.0 Hz, 1H, ArH), 7.70 (d, ^2J = 8.6 Hz, 2H, ArH), 7.58 (s, ArH), 7.50 (d, ^2J = 8.0 Hz, 1H, ArH), 7.47 (d, ^2J = 8.6 Hz, 2H, ArH), 4.25 (m, 2H, CH_2), 4.05 (m, 8H, 4×CH_2), 3.85-3.40 (m, 246$H_{average}$, 123$_{average}$×CH_2), 3.70-3.51 (m, 36H, 18CH_2), 3.37 (s, 3H, CH_3), 3.20 (m, 2H, CH_2), 3.13 (m, 2H, CH_2), 2.84 (m, 2H, CH_2), 2.75 (m, 2H, CH_2). ^{13}C NMR ($CDCl_3$, 75 MHz) δ: 206.5 (C=O), 182.2 (C=S), 172.7 (C=O), 161.1, 156.4 (2×CH_{Ar}), 152.6, 147.1, 142.0, 140.9, 136.1, 133.8, 125.4 (CH_{Ar}), 125.3 (CH_{ar}), 124.7 (CH_{Ar}), 123.6 (CH_{Ar}), 122.5, 120.8 (CH_{Ar}), 120.0 (CH_{Ar}), 73.1-68.0 (12×CH_2), 63.9 (CH_2), 59.2 (CH_3), 48.6 (CH_2), 43.4 (CH_2), 36.6 (CH_2), 25.1 (CH_2).

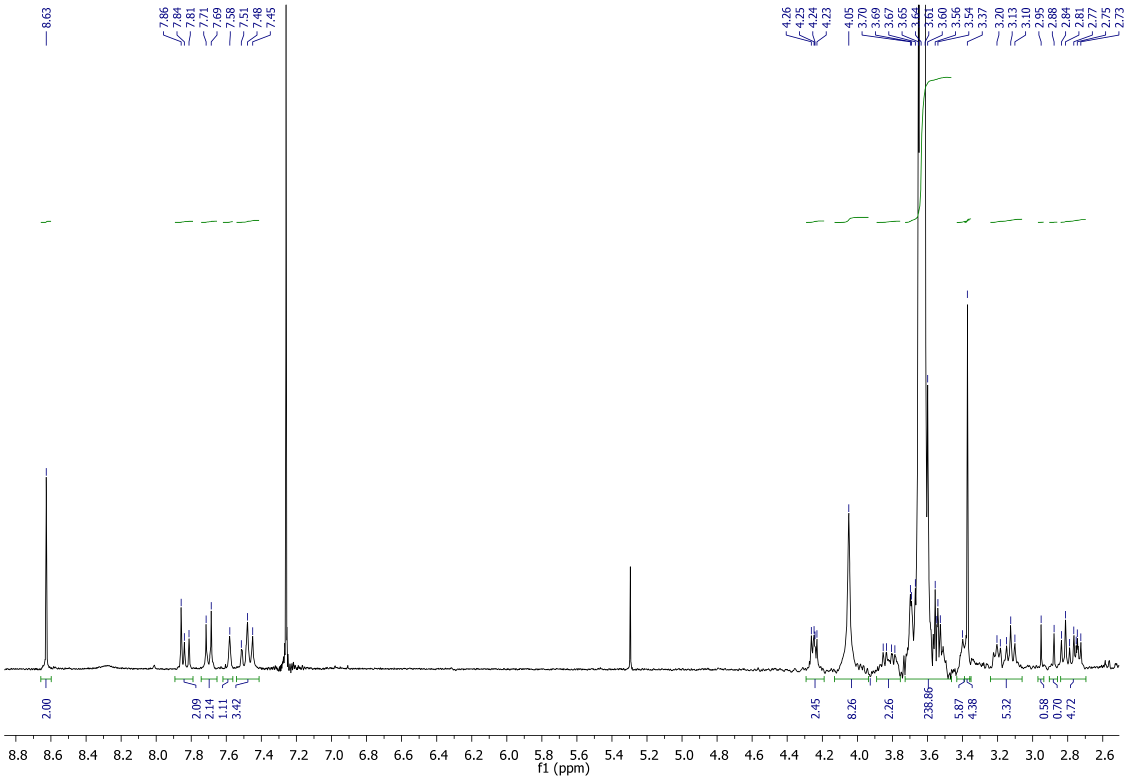

Figure S82: ^{1}H NMR ($CDCl_3$, 300 MHz) of 2-(2-methoxypolyethoxy)ethyl 3-(1-(4-(4-(5-(1-oxo-indan-5-yl)pyrimidin-2-yl)piperazine-1-carbothioamido)phenyl)-1H-1,2,3-triazol-4-yl)propanoate JG47

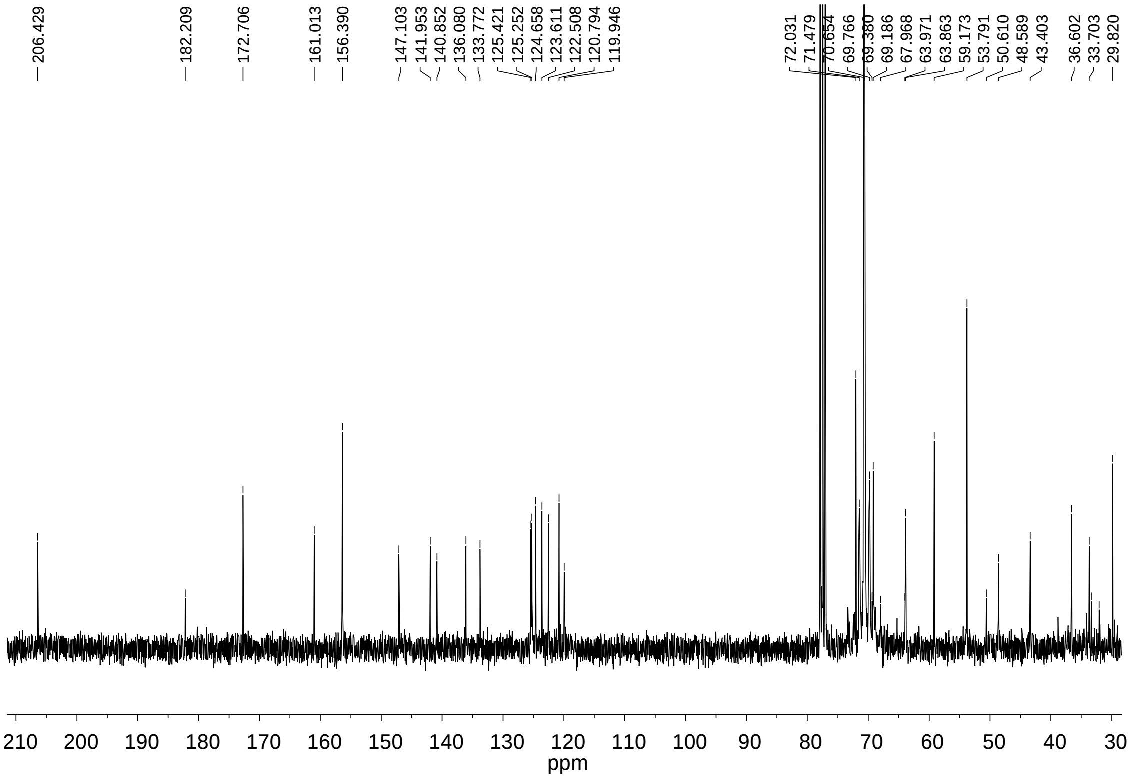

Figure S83: ^{13}C NMR ($CDCl_3$, 75 MHz) of 2-(2-methoxypolyethoxy)ethyl 3-(1-(4-(4-(5-(1-oxo-indan-5-yl)pyrimidin-2-yl)piperazine-1-carbothioamido)phenyl)-1H-1,2,3-triazol-4-yl)propanoate JG47

Solvatochromism

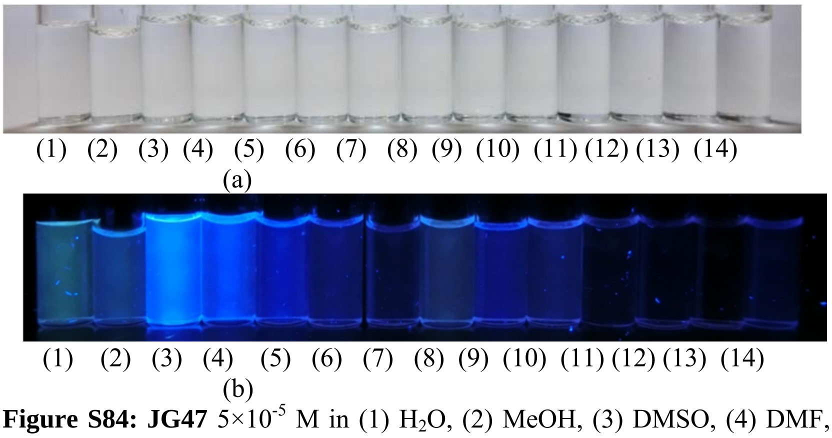

Figure S84: JG47 5×10^{-5} M in (1) H_2O, (2) MeOH, (3) DMSO, (4) DMF, (5) MCN, (6) Acetone, (7) AcOEt, (8) THF (9) $CHCl_3$ (10) CH_2Cl_2 (11) Toluene (12) Et_2O (13) Hexane (14) Cyclohexane under (a) direct sunlight and (b) 366 nm light.

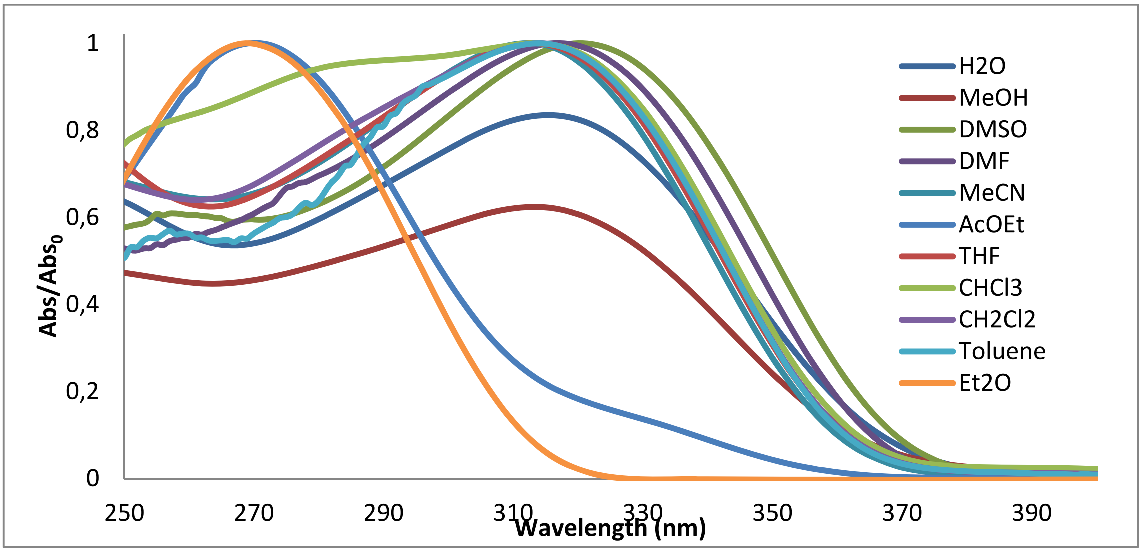

Figure S85: Normalized absorption spectra of **JG47** 5×10^{-5} M in different solvents.

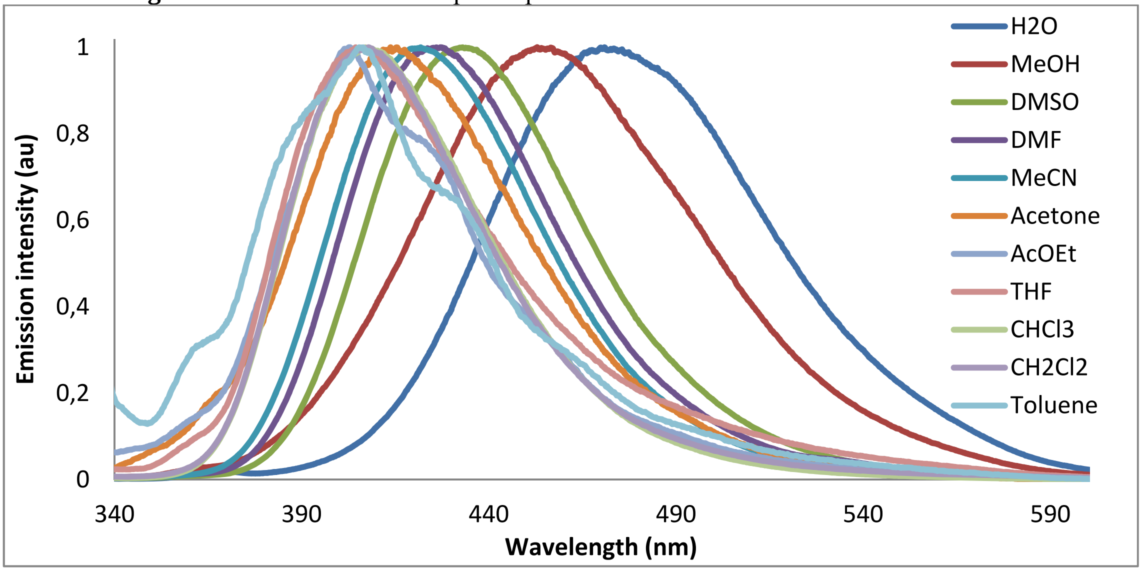

Figure S86: Normalized emission spectra of **JG47** 5×10^{-5} M in different solvents. $\lambda_{exc} = 366$ nm.

Qualitative experiments:

The changes in fluorescence in presence of Hg^{2+} and $MeHg^{+}$ were tested:

H_2O MeOH MECN

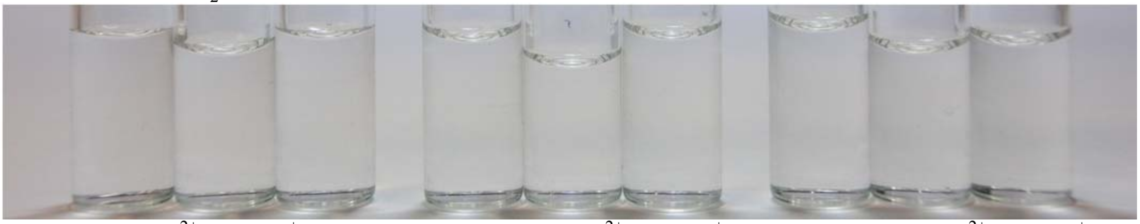

Water – Hg^{2+} - $MeHg^{+}$ Water – Hg^{2+} - $MeHg^{+}$ Water – Hg^{2+} - $MeHg^{+}$

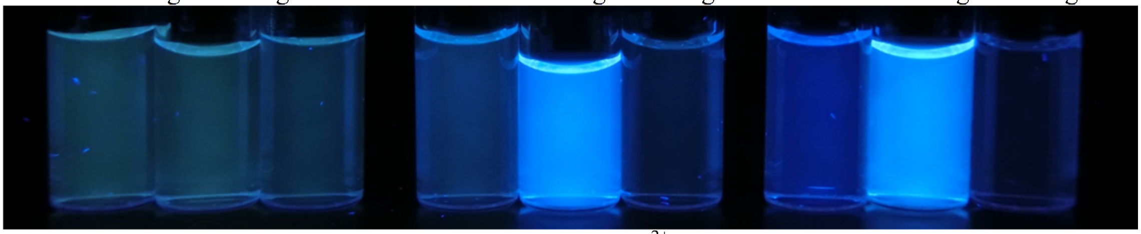

Figure S87: Fluorescence increasing in the presence of Hg^{2+} in MeOH and MeCN. There are no changes when $MeHg^{+}$ was added.

BD116/DNA interaction

The analysis of the BD116/ct6DNA system has been performed in DMSO/water mixtures content until 3% DMSO.

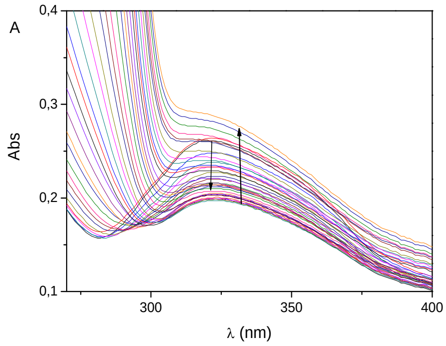

(A)

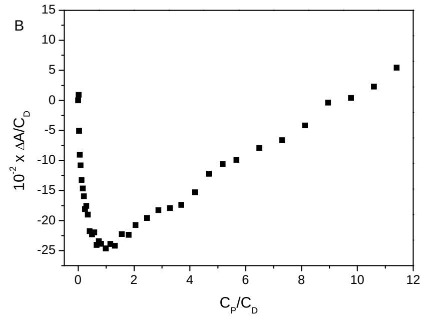

(B)

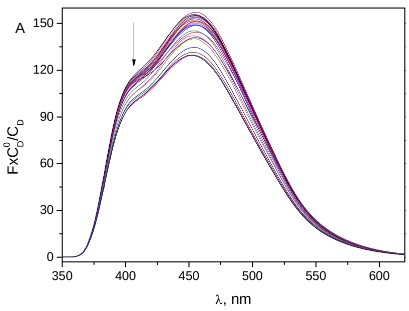

(C)

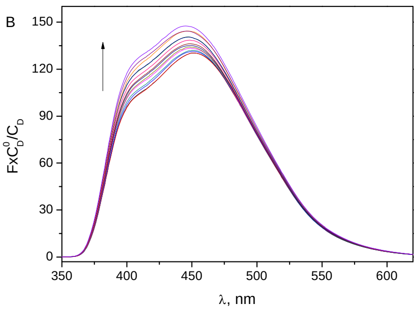

(D)

Figures S88A-D. (A) Spectrophotometric titration of the BD116/ctDNA (P/DNA) system, (B) Binding isotherm, λ = 325 nm. C_P = 2.61 × 10^{-5}M, C_{DNA}/C_P = 0 – 12, I = 0.1 M (NaCl), C) and D) Spectrofluorometric tritrations, C) C_{DNA}/C_P =0-50. D) C_{DNA}/C_P=50- 300. C_P = 6.4 × 10^{-6} M, pH = 7.0 and T = 25°C.

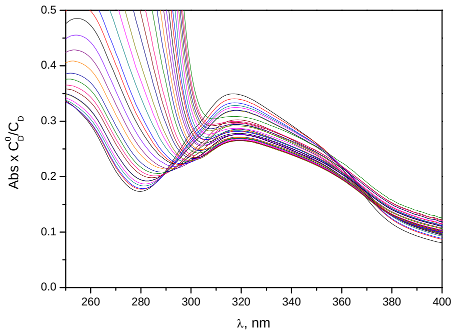

(A)

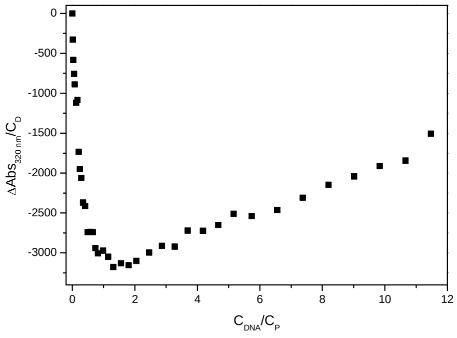

(B)

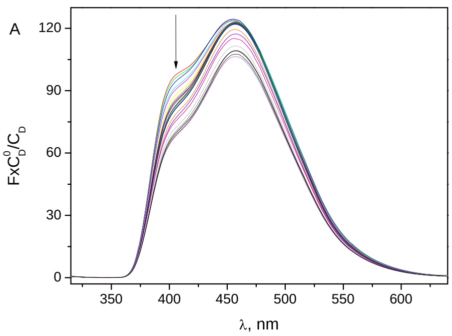

(C)

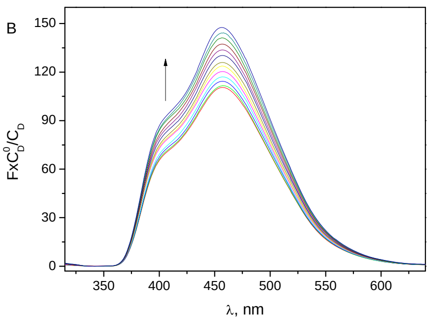

(D)

Figures S89A-D: A) Absorption spectrogram B) Binding isotherm of the system BD116/DNAMeHg, C_P = 2.61 × 10^{-5}M, C_{DNA}/C_P = 0 – 12. Fluorescence spectrogram, λ_{exc} = 320 nm, C_P = 6.4 × 10^{-6}M; C) C_{DNA}/C_P = 0 – 84, D) C_{DNA}/C_P = 94 – 295; I = 0.1 M (NaCl), pH = 7.0, T = 25°C

Relative viscosity is calculated from equation (1).

$$\frac{\eta}{\eta_0} = \frac{t - t_{solv}}{t_{DNA} - t_{solv}} \qquad (1)$$

where t, t_{solv} e t_{DNA} denote respectively the flow time of the sample (DAPI/DNA mixture), the flow time of the solvent and the flow time of 2.9×10^{-4}M DNA. Data have been collected at 20 and 40 °C; the results are shown in Figure 5. The complexity of the analysed systems is confirmed by the viscosity trend that changes it features depending on the reagents ratio, being ascending for $C_D/C_P <$ ca. 0.05 and descending for higher C_D/C_P.

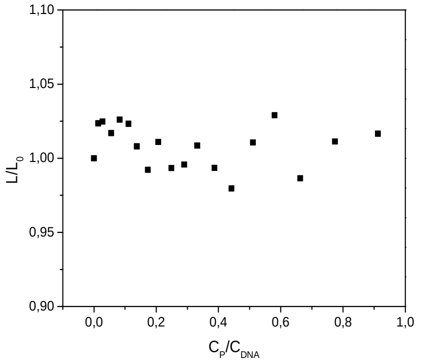

(A)

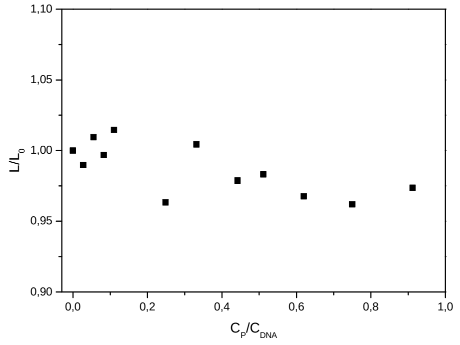

(B)

Figures S90A-B: Relative viscosities of both systems, C_P = 2.0 × 10^{-4}M, C_D/C_P = 0 – 1.0, I = 0.1 M (NaCl), pH = 7.0, T = 25°C

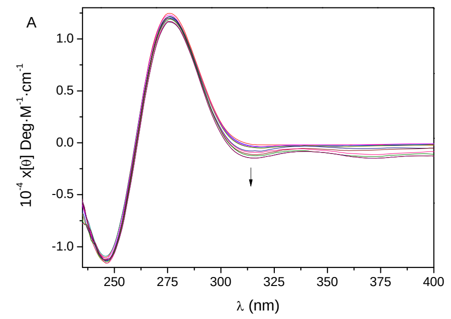

(C)

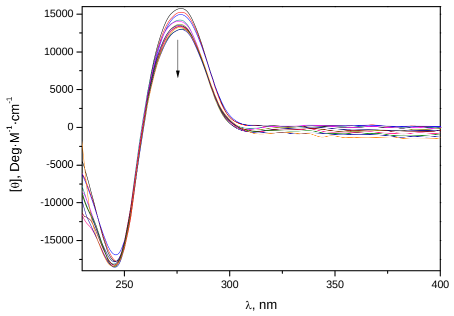

(D)

Figures S90C-D: CD spectra redorded for C) BD116/DNA abs D) BD116/(DNA + MeHg^{+}) systems. C_P between 4.07·10^{-6} and 7.59·10^{-5} M and C_{DNA}: 6.27·10^{-5} M. I = 0.1 M (NaCl), pH = 7.0.

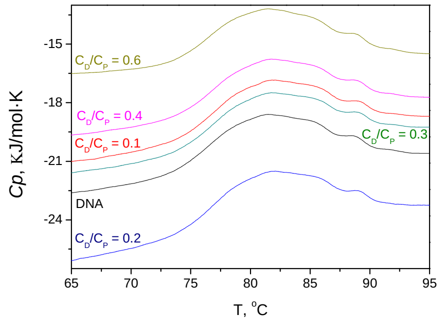

(E)

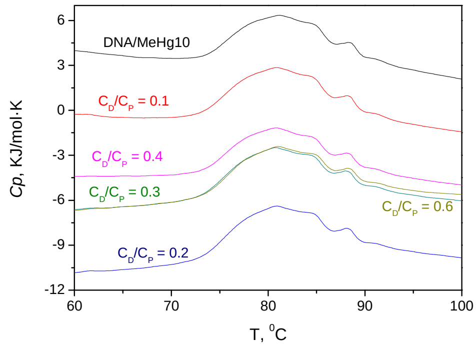

(F)

Figures S90E-F. DSC curves for both systems, C_P = 4.9 × 10^{-4}M, I = 0.1 M (NaCl), pH = 7.0, T = 20 - 95°C, scan rate 1°C/min, P = 3 atm.

Quantum Chemical Calculations:

BD116

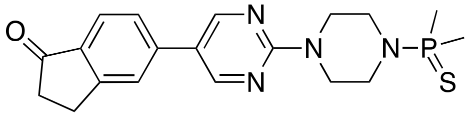

Standard orientation:

		Coordinates (Angstroms)		
Center Number	Atom	X	Y	Z
1	C	1.4905330000	-1.4069970000	0.1065310000
2	C	2.2750900000	-0.3014400000	-0.2596200000
3	C	1.5315040000	0.7901160000	-0.7374250000
4	C	-0.4367720000	-0.3229920000	-0.4501000000
5	H	1.9614140000	-2.3031250000	0.5089040000
6	H	2.0400810000	1.6919100000	-1.0763760000
7	C	3.7467920000	-0.2886610000	-0.1536600000
8	C	4.4277390000	0.8904960000	0.2021790000
9	C	4.4914530000	-1.4650470000	-0.4080580000
10	C	5.8166480000	0.8860580000	0.2942970000
11	H	3.8640160000	1.7911820000	0.4296560000
12	C	5.8755690000	-1.4746840000	-0.3056580000
13	H	3.9683890000	-2.3660280000	-0.7132360000
14	C	6.5295330000	-0.2903670000	0.0450200000
15	H	6.4534350000	-2.3726070000	-0.5028650000
16	C	6.7440640000	2.0300210000	0.6631890000
17	H	6.6301110000	2.8668430000	-0.0353130000
18	H	6.5043150000	2.4229820000	1.6578140000
19	C	8.1640970000	1.4168910000	0.6077490000
20	H	8.8113060000	1.9046670000	-0.1280730000
21	H	8.6890640000	1.4665930000	1.5669810000
22	C	7.9817650000	-0.0595300000	0.2126690000
23	O	8.8740580000	-0.8747480000	0.0691130000
24	N	0.1663240000	-1.4395990000	0.0193460000
25	N	0.2082610000	0.8004840000	-0.8393930000
26	C	-2.5617420000	0.7856070000	-1.0957350000
27	C	-2.5923920000	-1.5129750000	-0.2237260000
28	C	-3.7510150000	1.1322160000	-0.1993240000
29	H	-2.9310690000	0.5040260000	-2.0925350000
30	H	-1.8964970000	1.6412420000	-1.1996890000
31	C	-3.7846570000	-1.1408160000	0.6641300000
32	H	-2.9685980000	-1.9553010000	-1.1582810000

33	H	-1.9523100000	-2.2401930000	0.2739510000
34	H	-4.3719390000	1.8890920000	-0.6817050000
35	H	-3.3781220000	1.5451720000	0.7523310000
36	H	-4.4060000000	-2.0294000000	0.8043810000
37	H	-3.4128060000	-0.8302620000	1.6545940000
38	N	-1.8019610000	-0.3268240000	-0.5267940000
39	N	-4.5666570000	-0.0680480000	0.0361450000
40	P	-6.2478430000	0.0638690000	0.3308160000
41	C	-6.9333850000	-1.5003080000	-0.3393370000
42	H	-6.4681100000	-2.3872940000	0.0999000000
43	H	-8.0057210000	-1.5222640000	-0.1311490000
44	H	-6.7817420000	-1.5050130000	-1.4202110000
45	C	-6.5056090000	-0.0791040000	2.1484440000
46	H	-6.1078470000	0.8160120000	2.6318460000
47	H	-7.5774760000	-0.1366630000	2.3527340000
48	H	-6.0116160000	-0.9649940000	2.5597450000
49	S	-7.0640100000	1.6762730000	-0.4451910000

BD116-$MeHg^{+}$

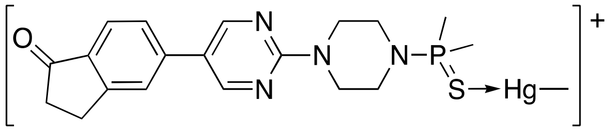

Standard orientation:

		Coordinates (Angstroms)		
Center Number	Atom	X	Y	Z
1	C	4.0746490000	0.1192220000	1.3616500000
2	C	4.8340540000	0.0269580000	0.1849770000
3	C	4.1068290000	0.2741650000	-0.9893470000
4	C	2.1958790000	0.6313830000	0.1888860000
5	H	4.5435220000	-0.0349770000	2.3318220000
6	H	4.5926470000	0.2058430000	-1.9611360000
7	C	6.2742660000	-0.3074480000	0.1846290000
8	C	7.1489040000	0.3145270000	-0.7243570000
9	C	6.7807960000	-1.2569540000	1.1015260000
10	C	8.5026600000	-0.0149770000	-0.7123480000
11	H	6.7711840000	1.0677780000	-1.4109830000
12	C	8.1316120000	-1.5808650000	1.1202160000
13	H	6.0975200000	-1.7574100000	1.7812640000
14	C	8.9809920000	-0.9529680000	0.2064960000

15	H	8.5322540000	-2.3116860000	1.8162840000
16	C	9.6193120000	0.5269340000	-1.5868370000
17	H	9.4164240000	0.3297630000	-2.6456450000
18	H	9.7030150000	1.6148980000	-1.4850810000
19	C	10.8934790000	-0.2027990000	-1.0950940000
20	H	11.3741450000	-0.7979270000	-1.8778280000
21	H	11.6558700000	0.4827010000	-0.7117700000
22	C	10.4449080000	-1.1367990000	0.0415090000
23	O	11.1583180000	-1.8829150000	0.6830890000
24	N	2.7752520000	0.4108810000	1.3815170000
25	N	2.8088310000	0.5738950000	-1.0059380000
26	C	0.0870120000	1.0456230000	-1.0391250000
27	C	0.0563480000	0.8816510000	1.4065480000
28	C	-0.8800000000	2.2319040000	-0.9761670000
29	H	-0.4797460000	0.1147470000	-1.2026550000
30	H	0.7802620000	1.1708930000	-1.8711950000
31	C	-0.9076130000	2.0690340000	1.4785970000
32	H	-0.5141320000	-0.0610590000	1.4302450000
33	H	0.7290690000	0.8922040000	2.2644140000
34	H	-1.5096060000	2.2542850000	-1.8678770000
35	H	-0.3137800000	3.1696790000	-0.9417670000
36	H	-1.5561810000	1.9777180000	2.3519420000
37	H	-0.3395290000	3.0008310000	1.5794420000
38	N	0.8522690000	0.9704180000	0.1939400000
39	N	-1.7280180000	2.2036820000	0.2453360000
40	P	-3.3072060000	1.6887750000	0.1924490000
41	C	-4.1285940000	2.3132640000	1.6960090000
42	H	-3.9201560000	3.3799320000	1.8153460000
43	H	-5.2064610000	2.1663540000	1.5869890000
44	H	-3.7960080000	1.7670230000	2.5798220000
45	C	-4.1025530000	2.5373610000	-1.2127080000
46	H	-3.7483700000	2.1371570000	-2.1637540000
47	H	-5.1821890000	2.3759010000	-1.1512870000
48	H	-3.8952480000	3.6095850000	-1.1594180000
49	S	-3.4962070000	-0.3650630000	0.0345250000
50	Hg	-5.9942060000	-1.1048470000	-0.1761410000
51	C	-8.0748260000	-1.8550710000	-0.3703300000
52	H	-8.7200080000	-1.1493690000	0.1497380000
53	H	-8.2936420000	-1.9016750000	-1.4355780000
54	H	-8.0905440000	-2.8397460000	0.0937450000

BD116-MeHg^{+} (Through N)

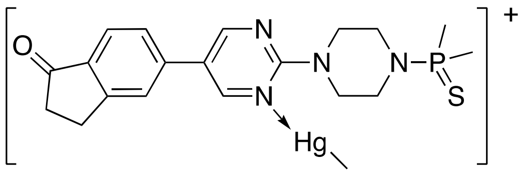

Standard orientation:

		Coordinates (Angstroms)		
Center Number	Atom	X	Y	Z
1	C	1.6914300000	-2.0494580000	-0.8784050000
2	C	2.4862090000	-1.0042820000	-0.3469510000
3	C	1.7620710000	0.1177680000	0.0198880000
4	C	-0.2663340000	-0.9350140000	-0.4360050000
5	H	2.1634540000	-2.9487610000	-1.2697310000
6	H	2.2769990000	1.0148930000	0.3531020000
7	C	3.9596630000	-1.0782460000	-0.2472460000
8	C	4.6197160000	-0.5356750000	0.8695500000
9	C	4.7033350000	-1.6959340000	-1.2770840000
10	C	6.0097130000	-0.6091910000	0.9454280000
11	H	4.0450660000	-0.0960960000	1.6815640000
12	C	6.0889800000	-1.7724150000	-1.1998070000
13	H	4.1907340000	-2.0900990000	-2.1496430000
14	C	6.7250840000	-1.2242980000	-0.0848610000
15	H	6.6779170000	-2.2379430000	-1.9842050000
16	C	6.9314250000	-0.1110700000	2.0443830000
17	H	6.8224130000	0.9702060000	2.1852760000
18	H	6.6799780000	-0.5758490000	3.0042260000
19	C	8.3550450000	-0.4929530000	1.5687480000
20	H	9.0106820000	0.3742540000	1.4437920000
21	H	8.8653090000	-1.1730500000	2.2577980000
22	C	8.1845150000	-1.1928540000	0.2116430000
23	O	9.0676570000	-1.6380080000	-0.4913100000
24	N	0.3736010000	-2.0227610000	-0.9174420000
25	N	0.4060350000	0.1914970000	-0.0445680000
26	C	-2.4108600000	-0.0019220000	0.3895560000
27	C	-2.3906700000	-1.9570230000	-1.1548920000
28	C	-3.5843100000	-0.6644680000	1.1249800000
29	H	-2.8148110000	0.7579700000	-0.3006470000
30	H	-1.7741540000	0.4743430000	1.1401610000

31	C	-3.5631260000	-2.5468680000	-0.3659310000
32	H	-2.7848670000	-1.4261370000	-2.0319980000
33	H	-1.7105300000	-2.7385370000	-1.4875200000
34	H	-4.2187650000	0.1121820000	1.5589200000
35	H	-3.1783030000	-1.2818350000	1.9432130000
36	H	-4.1566730000	-3.1548750000	-1.0525110000
37	H	-3.1814230000	-3.2120230000	0.4253340000
38	N	-1.6197530000	-0.9934310000	-0.3413060000
39	N	-4.3676390000	-1.4626740000	0.1955210000
40	P	-6.0874300000	-1.5386980000	0.3378890000
41	C	-6.6477080000	-1.8579010000	-1.3757940000
42	H	-6.2194540000	-2.7705770000	-1.7999590000
43	H	-7.7356790000	-1.9589670000	-1.3586780000
44	H	-6.3862500000	-1.0001510000	-1.9983140000
45	C	-6.4858920000	-3.0823170000	1.2475910000
46	H	-6.1533360000	-2.9817930000	2.2831960000
47	H	-7.5692700000	-3.2271510000	1.2449760000
48	H	-6.0072120000	-3.9548710000	0.7913810000
49	S	-6.8253960000	0.0914020000	1.1438090000
50	Hg	-0.3830780000	2.4047840000	-0.2358130000
51	C	-0.9482410000	4.5279960000	-0.4380270000
52	H	-1.6643990000	4.5772340000	-1.2559450000
53	H	-1.3874710000	4.8208360000	0.5136290000
54	H	-0.0331550000	5.0744760000	-0.6572220000

BD116-Hg(II)

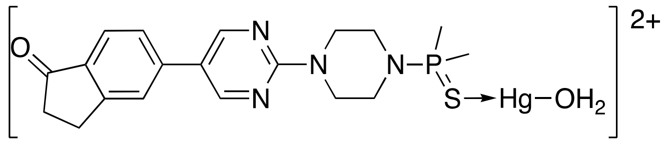

Standard orientation:

		Coordinates (Angstroms)		
Center Number	Atom	X	Y	Z
1	C	4.0553190000	-0.1783290000	1.3440040000
2	C	4.8582420000	-0.1011530000	0.1756610000
3	C	4.1407680000	0.2529790000	-0.9970770000
4	C	2.1903430000	0.3666220000	0.1644900000
5	H	4.5019420000	-0.4037420000	2.3088800000

6	H	4.6475940000	0.3201870000	-1.9563280000
7	C	6.2916410000	-0.3606790000	0.1811140000
8	C	7.1335350000	0.2147700000	-0.8057790000
9	C	6.8588710000	-1.2012930000	1.1842950000
10	C	8.4960890000	-0.0423810000	-0.7788340000
11	H	6.7222280000	0.8887970000	-1.5509310000
12	C	8.2170410000	-1.4651470000	1.2043350000
13	H	6.2159270000	-1.6726810000	1.9196640000
14	C	9.0224830000	-0.8694550000	0.2269500000
15	H	8.6626710000	-2.1150670000	1.9508450000
16	C	9.5874420000	0.4696710000	-1.7001960000
17	H	9.3867550000	0.1783530000	-2.7369460000
18	H	9.6232820000	1.5643360000	-1.6860290000
19	C	10.8957260000	-0.1639880000	-1.1641300000
20	H	11.3888190000	-0.8094170000	-1.8972570000
21	H	11.6361390000	0.5810330000	-0.8566920000
22	C	10.5002030000	-1.0040440000	0.0547490000
23	O	11.2257190000	-1.6768880000	0.7555730000
24	N	2.7536000000	0.0514150000	1.3499400000
25	N	2.8386920000	0.4806120000	-1.0142890000
26	C	0.1080990000	0.9456180000	-1.0459990000
27	C	0.0272360000	0.5264870000	1.3586870000
28	C	-0.6427660000	2.2807750000	-0.8377060000
29	H	-0.6341870000	0.1587530000	-1.2541050000
30	H	0.8047760000	1.0184940000	-1.8796900000
31	C	-0.7229770000	1.8625560000	1.5650330000
32	H	-0.7191710000	-0.2753310000	1.2402260000
33	H	0.6671150000	0.3044860000	2.2113760000
34	H	-1.2406590000	2.5040600000	-1.7222550000
35	H	0.0816500000	3.0923780000	-0.7097330000
36	H	-1.3797830000	1.7803070000	2.4322430000
37	H	-0.0008340000	2.6610990000	1.7665020000
38	N	0.8365400000	0.5900750000	0.1570100000
39	N	-1.4918490000	2.2522530000	0.3679150000
40	P	-3.1370450000	1.8913520000	0.2553530000
41	C	-3.9166920000	2.4770210000	1.7910880000
42	H	-3.6315930000	3.5167270000	1.9762510000
43	H	-5.0013460000	2.4216130000	1.6630080000
44	H	-3.6409710000	1.8541270000	2.6432060000
45	C	-3.8159050000	2.9050750000	-1.0936150000

46	H	-3.4839950000	2.5471230000	-2.0693010000
47	H	-4.9064420000	2.8313460000	-1.0552330000
48	H	-3.5243210000	3.9502430000	-0.9548340000
49	S	-3.4084620000	-0.1270590000	-0.0525850000
50	Hg	-6.0439790000	-1.0666240000	-0.2150320000
51	O	-8.4265070000	-1.8837030000	-0.2526620000
52	H	-8.8913700000	-2.1596430000	-1.0564010000
53	H	-8.8355500000	-2.3713850000	0.4774170000

BD119

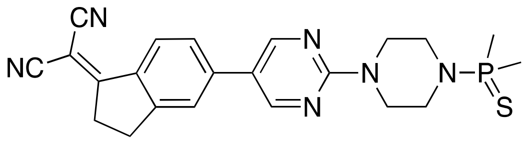

Standard orientation:

		Coordinates (Angstroms)		
Center Number	Atom	X	Y	Z
1	C	-0.4789790000	1.2334410000	0.1332440000
2	C	-1.2200590000	0.1082400000	-0.2676400000
3	C	-0.4333650000	-0.9379660000	-0.7803050000
4	C	1.4889390000	0.2449710000	-0.4602160000
5	H	-0.9830670000	2.0964860000	0.5663090000
6	H	-0.9048960000	-1.8482540000	-1.1488100000
7	C	-2.6859820000	0.0323850000	-0.1614270000
8	C	-3.3280200000	-1.1922910000	0.1016040000
9	C	-3.4817900000	1.1906590000	-0.3220970000
10	C	-4.7115670000	-1.2477260000	0.1943910000
11	H	-2.7376790000	-2.0900580000	0.2596510000
12	C	-4.8634230000	1.1483870000	-0.2211200000
13	H	-3.0021920000	2.1349990000	-0.5577360000
14	C	-5.4945330000	-0.0831960000	0.0387460000
15	H	-5.4395180000	2.0555220000	-0.3554420000
16	C	-5.5702740000	-2.4564610000	0.4750350000
17	H	-5.4254280000	-3.2326430000	-0.2845810000
18	H	-5.3134630000	-2.9117590000	1.4379620000
19	C	-7.0177040000	-1.9076880000	0.4669270000
20	H	-7.6383740000	-2.3845090000	-0.2993910000
21	H	-7.5337200000	-2.0775940000	1.4179240000
22	C	-6.8996860000	-0.4188750000	0.1927770000
23	N	0.8412520000	1.3209500000	0.0468430000

24	N	0.8873710000	-0.8915770000	-0.8842930000
25	C	3.6552140000	-0.7604340000	-1.1348660000
26	C	3.5963730000	1.5091000000	-0.1883910000
27	C	4.8426510000	-1.0956120000	-0.2314500000
28	H	4.0286330000	-0.4310640000	-2.1148650000
29	H	3.0212170000	-1.6338240000	-1.2777960000
30	C	4.7884170000	1.1470820000	0.7046210000
31	H	3.9699560000	1.9906340000	-1.1038750000
32	H	2.9257620000	2.1990610000	0.3217250000
33	H	5.4950850000	-1.8158910000	-0.7281290000
34	H	4.4703720000	-1.5512950000	0.7007940000
35	H	5.3768150000	2.0510150000	0.8828080000
36	H	4.4134710000	0.7923520000	1.6789740000
37	N	2.8494610000	0.3075700000	-0.5421220000
38	N	5.6137640000	0.1225510000	0.0532970000
39	P	7.2970390000	0.0371370000	0.3587220000
40	C	7.9336700000	1.6477560000	-0.2459000000
41	H	7.4418600000	2.5010130000	0.2296310000
42	H	9.0046820000	1.6936400000	-0.0349430000
43	H	7.7827390000	1.6928400000	-1.3259480000
44	C	7.5344260000	0.1201080000	2.1826490000
45	H	7.1609680000	-0.8043250000	2.6288460000
46	H	8.6021810000	0.2031650000	2.3993070000
47	H	7.0097830000	0.9744920000	2.6217900000
48	S	8.1703900000	-1.5175610000	-0.4701500000
49	C	-8.0024190000	0.3964800000	0.1213540000
50	C	-7.9367660000	1.8008030000	-0.1333200000
51	N	-7.8826540000	2.9458320000	-0.3391930000
52	C	-9.3087170000	-0.1566530000	0.3083920000
53	N	-10.3585450000	-0.6360600000	0.4639410000

BD119-MeHg^{+}

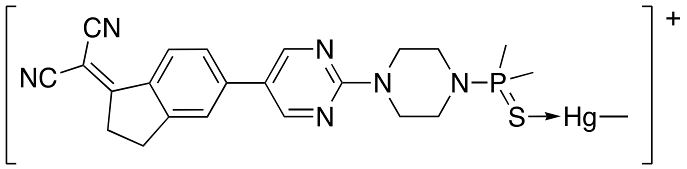

Standard orientation:

		Coordinates (Angstroms)		
Center Number	Atom	X	Y	Z

1	C	3.1578020000	0.1704200000	1.2604760000
2	C	3.9026680000	0.4094840000	0.0944090000
3	C	3.1446820000	0.8932890000	-0.9834700000
4	C	1.2348870000	0.8411590000	0.2491530000
5	H	3.6488950000	-0.1850830000	2.1645060000
6	H	3.6174490000	1.0910120000	-1.9439760000
7	C	5.3562850000	0.1658080000	0.0096880000
8	C	6.1847040000	1.0132830000	-0.7468070000
9	C	5.9369610000	-0.9270070000	0.6893380000
10	C	7.5504390000	0.7661180000	-0.8174540000
11	H	5.7620070000	1.8765000000	-1.2533930000
12	C	7.3005850000	-1.1784290000	0.6312530000
13	H	5.3015590000	-1.6051950000	1.2502760000
14	C	8.1210650000	-0.3262490000	-0.1300720000
15	H	7.7125060000	-2.0288760000	1.1601860000
16	C	8.5967460000	1.5604050000	-1.5607300000
17	H	8.3740870000	1.6107370000	-2.6322280000
18	H	8.6365750000	2.5950350000	-1.2019410000
19	C	9.9205720000	0.8062730000	-1.2816420000
20	H	10.3823530000	0.4284000000	-2.2003740000
21	H	10.6738930000	1.4452300000	-0.8089590000
22	C	9.5590280000	-0.3503820000	-0.3672970000
23	N	1.8458090000	0.3738710000	1.3525460000
24	N	1.8327740000	1.1120490000	-0.9249370000
25	C	-0.9121370000	1.4265070000	-0.8356120000
26	C	-0.8954160000	0.6656950000	1.4961270000
27	C	-1.9261270000	2.5167660000	-0.4765500000
28	H	-1.4430110000	0.5397300000	-1.2174240000
29	H	-0.2387090000	1.7818270000	-1.6157920000
30	C	-1.9119650000	1.7516330000	1.8612520000
31	H	-1.4213230000	-0.2801620000	1.2899950000
32	H	-0.2105350000	0.4989420000	2.3277290000
33	H	-2.5685280000	2.7317820000	-1.3325220000
34	H	-1.3989020000	3.4408160000	-0.2129880000
35	H	-2.5446740000	1.4148970000	2.6846280000
36	H	-1.3864800000	2.6551990000	2.1908300000
37	N	-0.1240030000	1.0842850000	0.3370220000
38	N	-2.7538260000	2.1484240000	0.7020120000
39	P	-4.3112500000	1.5921630000	0.5289290000
40	C	-5.1437120000	1.8112820000	2.1367860000

41	H	-4.9811250000	2.8291730000	2.5013930000
42	H	-6.2153430000	1.6448640000	1.9980800000
43	H	-4.7769190000	1.0902870000	2.8687450000
44	C	-5.1555500000	2.7088690000	-0.6397830000
45	H	-4.7988790000	2.5521720000	-1.6587930000
46	H	-6.2269260000	2.4925750000	-0.6132560000
47	H	-4.9907900000	3.7479130000	-0.3418290000
48	S	-4.4109600000	-0.3746220000	-0.1035500000
49	C	10.4987570000	-1.2272080000	0.1092140000
50	C	10.1910250000	-2.3261940000	0.9700710000
51	N	9.9324350000	-3.2184870000	1.6718040000
52	C	11.8735920000	-1.0641120000	-0.2549190000
53	N	12.9832740000	-0.9061070000	-0.5688800000
54	Hg	-6.8756830000	-1.2054560000	-0.3771420000
55	C	-8.9190310000	-2.0296930000	-0.6506960000
56	H	-8.8515800000	-2.7514560000	-1.4629320000
57	H	-9.1967820000	-2.4985830000	0.2914970000
58	H	-9.5704160000	-1.1942630000	-0.9006200000

BD119-Hg(II)

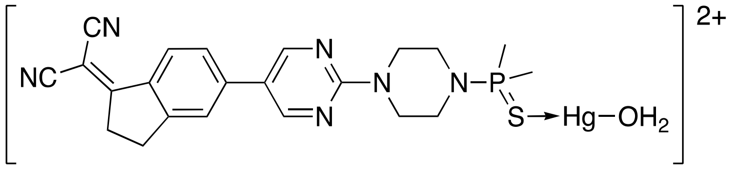

Standard orientation:

		Coordinates (Angstroms)		
Center Number	Atom	X	Y	Z
1	C	3.1444830000	0.0018340000	1.1885230000
2	C	3.9158520000	0.3173770000	0.0404430000
3	C	3.1611230000	0.8610730000	-1.0302530000
4	C	1.2345200000	0.6920170000	0.1656260000
5	H	3.6187870000	-0.3820690000	2.0881740000
6	H	3.6403910000	1.1230670000	-1.9702900000
7	C	5.3549110000	0.1024580000	-0.0307180000
8	C	6.1605300000	0.8404030000	-0.9321190000
9	C	5.9784310000	-0.8662900000	0.8077450000
10	C	7.5241280000	0.6181130000	-0.9833120000
11	H	5.7208400000	1.6092770000	-1.5588600000
12	C	7.3377320000	-1.1011550000	0.7600760000

13	H	5.3743030000	-1.4680270000	1.4770880000
14	C	8.1311560000	-0.3541480000	-0.1404090000
15	H	7.7737600000	-1.8558140000	1.4019630000
16	C	8.5480730000	1.3065300000	-1.8486950000
17	H	8.3100690000	1.1936660000	-2.9117510000
18	H	8.5725170000	2.3831710000	-1.6474150000
19	C	9.8878850000	0.6245940000	-1.4802920000
20	H	10.3427590000	0.1168610000	-2.3383240000
21	H	10.6374410000	1.3389320000	-1.1232460000
22	C	9.5551410000	-0.3811120000	-0.3971720000
23	N	1.8375830000	0.1830900000	1.2616910000
24	N	1.8533720000	1.0464950000	-0.9817440000
25	C	-0.8930360000	1.4031130000	-0.8844910000
26	C	-0.9080090000	0.5109930000	1.3927910000
27	C	-1.7258340000	2.6139600000	-0.4098960000
28	H	-1.5817540000	0.6255100000	-1.2505080000
29	H	-0.2140740000	1.6897750000	-1.6861090000
30	C	-1.7401070000	1.7245830000	1.8605580000
31	H	-1.6014480000	-0.3018270000	1.1236200000
32	H	-0.2403920000	0.1699310000	2.1825440000
33	H	-2.3489390000	2.9726280000	-1.2301080000
34	H	-1.0547480000	3.4299130000	-0.1200990000
35	H	-2.3749630000	1.4281290000	2.6969300000
36	H	-1.0711830000	2.5168530000	2.2139340000
37	N	-0.1238160000	0.8612890000	0.2222360000
38	N	-2.5547910000	2.2890860000	0.7666430000
39	P	-4.1693800000	1.8294360000	0.5871900000
40	C	-4.9729300000	2.0830910000	2.1999450000
41	H	-4.7722630000	3.0971630000	2.5573830000
42	H	-6.0509100000	1.9561670000	2.0664300000
43	H	-4.6323090000	1.3521070000	2.9347060000
44	C	-4.9332420000	3.0110380000	-0.5662810000
45	H	-4.5890690000	2.8489680000	-1.5888100000
46	H	-6.0151020000	2.8530880000	-0.5417170000
47	H	-4.7149730000	4.0353030000	-0.2505210000
48	S	-4.3112330000	-0.1154180000	-0.0649880000
49	C	10.5185540000	-1.1668890000	0.2050500000
50	C	10.2352050000	-2.1197820000	1.2274800000
51	N	9.9883320000	-2.8937740000	2.0620630000
52	C	11.8823220000	-1.0413210000	-0.1950190000

53	N	12.9867500000	-0.9155470000	-0.5434160000
54	Hg	-6.9175500000	-1.1639260000	-0.3501450000
55	H	-9.2467000000	-3.3364790000	-0.1238830000
56	H	-9.3649150000	-2.8068110000	-1.5760510000
57	O	-8.9357590000	-2.6434500000	-0.7239180000

$[Hg(OH_2)_2]^{2+}$

Standard orientation:

		Coordinates (Angstroms)		
Center Number	Atom	X	Y	Z
1	Hg	0.0000000000	0.0000000000	0.0102340000
2	H	-0.5192430000	2.7709200000	0.5445540000
3	H	0.6130030000	2.7396670000	-0.5757690000
4	H	0.5192430000	-2.7709200000	0.5445540000
5	H	-0.6130030000	-2.7396670000	-0.5757690000
6	O	0.0000000000	2.1926430000	-0.0472690000
7	O	0.0000000000	-2.1926430000	-0.0472690000

$[Hg(CH_3)(OH_2)]^{+}$

Standard orientation:

		Coordinates (Angstroms)		
Center Number	Atom	X	Y	Z
1	C	-0.1356930000	2.2547640000	0.0000000000
2	H	0.3726900000	2.5616990000	0.9108530000
3	H	-1.2026210000	2.4644580000	0.0000000000
4	H	0.3726900000	2.5616990000	-0.9108530000
5	Hg	0.0000000000	0.0395360000	0.0000000000
6	H	0.0915900000	-2.8844680000	0.7808910000
7	H	0.0915900000	-2.8844680000	-0.7808910000
8	O	0.1360270000	-2.3137980000	0.0000000000

H_2O

Standard orientation:

		Coordinates (Angstroms)		
Center Number	Atom	X	Y	Z
1	O	0.0000000000	0.0000000000	0.1191880000
2	H	0.0000000000	0.7592860000	-0.4767540000
3	H	0.0000000000	-0.7592860000	-0.4767540000

Topology of the frontier orbitals:

BD116

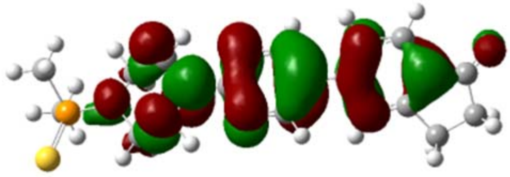

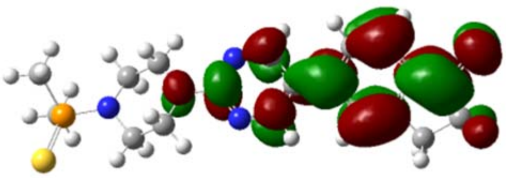

HOMO LUMO

Figure S91: In compound BD116, HOMO displays a π bonding structure centered in the fragment phenylpyrimidine and π antibonding contributions of the piperazine fragment as well as small contribution of a p orbital of the carbonyl oxygen atom. In the same compound, LUMO is a π antibonding centered in the phenypyrimidine fragment and the neighbor carbonyl fragment.

BD119

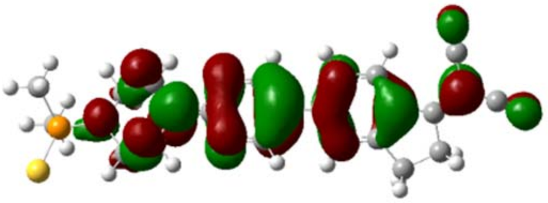

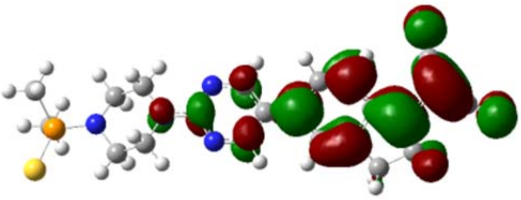

HOMO LUMO

Figure S92: In compound BD119, HOMO displays a π bonding structure centered in the fragment phenylpyrimidine and π antibonding contributions of the piperazine fragment as well as small contribution of p orbitals of the central carbon and nitrogen atoms of the unit $C(CN)_2$. In the same compound, LUMO is a π antibonding centered in the phenyl ring of the phenypyrimidine fragment along with π bonding contribution of the fragment C-C-C and two C-N π antibonding contributions of the neighbor $C(CN)_2$ fragment.

BD116-$MeHg^+$

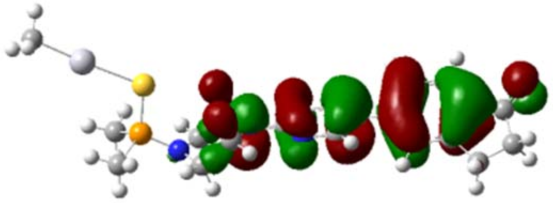

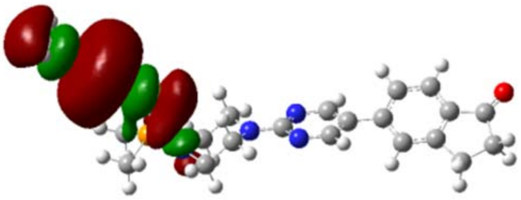

HOMO LUMO

Figure S93: In complex BD116-$MeHg^+$, HOMO displays a π bonding structure centered in the fragment phenylpyrimidine and π antibonding contributions of the p orbital of the nitrogen atom of piperazine fragment directly bonded to the pyrimidine ring, as well as small contribution of a p orbital of the carbonyl oxygen atom. In the same compound, LUMO is an σ antibonding interaction centered in the C-Hg-S fragment.

BD116-Hg(II)

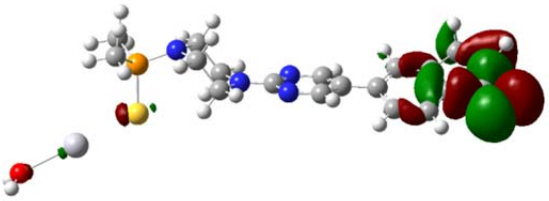

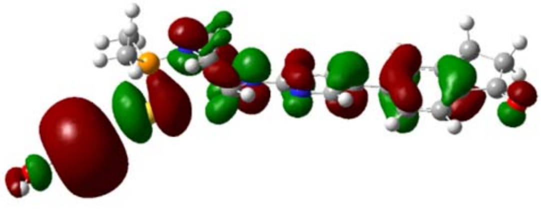

HOMO LUMO

Figure S94: In complex BD116-Hg(II), HOMO is composed mainly by a combination of atomic orbitals of the carbonyl atoms and the two carbon atoms directly bonded to the carbonyl carbon atom. This orbital can be described as two σ bonding C-C interactions and one in plane π antibonding C-O interaction. LUMO for this compound is much delocalized over the molecule and can be described as π antibonding over the fragment phenylpyrimidine with contributions of atomic orbitals p of the piperazine fragment and σ antibonding over the C-Hg-S fragment.

BD119-$MeHg^+$

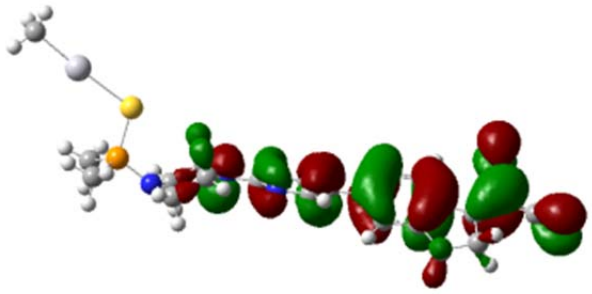

HOMO

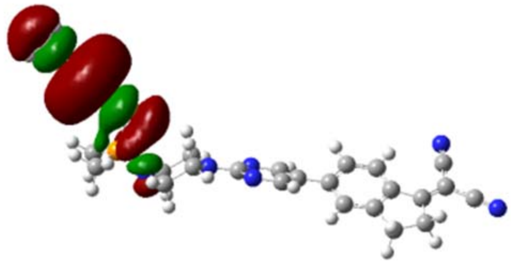

LUMO

Figure S95: In complex BD119-$MeHg^+$, HOMO displays a π bonding structure centered in the fragment phenylpyrimidine and π antibonding contributions of the p orbital of the nitrogen atom of piperazine fragment directly bonded to the pyrimidine ring, as well as C-C and C-N π bonding interactions in the $C=C(CN)_2$ fragment. In the same compound, LUMO is an σ antibonding interaction centered in the C-Hg-S fragment.

BD119-Hg(II)

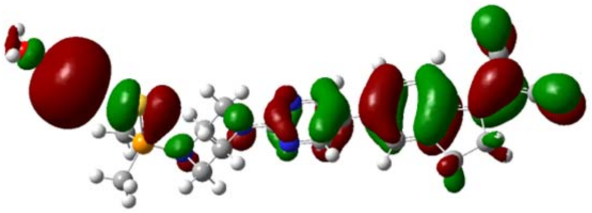

HOMO

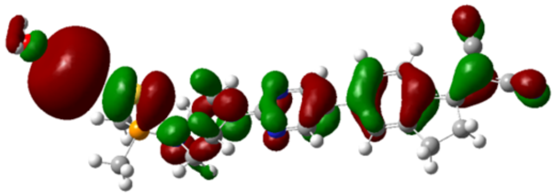

LUMO

Figure S96: In complex BD119-Hg(II), both HOMO and LUMO are delocalized over all the structure of the compound with a π bonding structure centered over the fragment phenylpyrimidine, C-C and C-N π bonding interactions in the $C=C(CN)_2$ fragment and σ antibonding interaction over the C-Hg-S fragment. The main difference between these two orbitals is the participation (not very high) of p orbitals of the carbon atoms of the piperazine ring.

Speciation: In this section, a method for allowing the determination and differentiation of Hg(II) and MeHg(II) mixtures will be explained, by using for this purpose the dyes **BD116** and **BD119**, both at C = 2,5 × 10^{-5} M in MeOH:water 20:80 or MeOH:water 40:60 respectively. The reason is that **BD116** has experimentally demonstrated to be sensitive to both species (Hg(II) and MeHg(II)) whereas the probe **BD119** is only sensitive to Hg(II). Speciation was carried out by using the standard addition method, in which we tried to calculate the concentration of both analytes in a test sample. First of all, Hg(II) and MeHg(II) solutions with a concentration 5 × 10^{-3} M in water were prepared and from these other ones at concentration 10^{-3} M were made by simple dilution. Another test sample was prepared by using the concentrated standard solution, consisting of 80 µL of Hg(II) 5 × 10^{-3} M and 120 µL of MeHg(II) 5 × 10^{-3} M diluted to 1 mL using deionized water. These amounts of analytes were chosen because when they are placed in the cuvette, their amount is larger than the limit of detection calculated for both ($C_{Hg(II),cuvette}$= 4.74 × 10^{-6} M; $C_{MeHg(II),cuvette}$= 7.11 × 10^{-6} M). Once all the solutions were prepared, 2500 µL of the probe solution are pipetted to a fluorescence cuvette and measured exciting at the corresponding excitation wavelength (350 nm for **BD116** and 398 nm for **BD119**) to obtain the blank spectrum. Then 30 µL of the test solution were added to the cuvette, and then it was shaken and measured again. Finally, the calibration curve was performed by successive additions of Hg(II) or MeHg(II) 10^{-3} M, within the linear response concentration range of the probe. In short, 15 calibration curves were recorded following this procedure:

BD119+TestSample+Hg(II) (3 replicates)

BD116+TestSample+Hg(II) (3 replicates)

BD116+TestSample+MeHg(II) (3 replicates)

Once each calibration curve is measured, the fluorescence blank (fluorescence due to the probe) is subtracted for all other measurements. Thus the increase due to the analytes is calculated regardless of the initial value. Finally every regression was validated using least median of squares, a mathematical tool to eliminate outliers, and then by least squares to obtain the linear regression equation. With the calibration curve between **BD119** and Hg(II) it is obtained the amount of Hg(II) contained in the test sample and not the MeHg(II) because this probe is not sensitive to this analyte. After three replicates, a mean value and a confidence interval, with a 95% confidence level and 2 degrees of freedom (number of observations minus one) was obtained:

Replicate	Slope	Intercept	Adj. R^2	$C_{Hg(II)}$ calculated (M)
1	1.9662 × 10^{7}	75.5063	0.9617	3.84 × 10^{-6}
2	2.2931 × 10^{7}	107.8794	0.9841	4.79 × 10^{-6}
3	4.2178 × 10^{6}	13.6108	0.9699	3.23 × 10^{-6}
4	1.3451 × 10^{6}	99.7300	0.9108	7.4143 × 10^{-6}

Table S1. Curves parameters obtained after each calibration of **BD119**-Hg(II).

Mean (M)	Error (M)	Confidence Interval[12] (M)	Theoretical Value (M)
4.82 × 10^{-6}	2.17 × 10^{-6}	[2.65 × 10^{-6}; 6.99 × 10^{-6}]	4.74 × 10^{-6}

Table S2. Statistical parameters of the regression of **BD119**-Hg(II).

It can be observed how the theoretical value and the calculated one are very close, being also included in the confidence interval. This theoretical value will be considered from now on as the real concentration of Hg(II) in the test sample. It is worth to emphasise the importance of this value for the remainder of the method, because it has

[12] The confidence interval is given by the expression: $\left[\bar{x} - t_{(n-1,95\%)}\frac{S}{\sqrt{n}}; \bar{x} + t_{(n-1,95\%)}\frac{S}{\sqrt{n}}\right]$

influence on the calculation of the MeHg(II) concentration and therefore it is very important to determine it with accuracy. Secondly, calibration curves between **BD116** and Hg(II) were measured in a similar way to the explained for **BD119**-Hg(II) but exciting at 350 nm instead. With this calibration it was obtained the total concentration due to Hg(II) and MeHg(II) in the test sample. Subtracting the calculated Hg(II) concentration from the whole value it was obtained the hypothetical amount of MeHg(II) that should be in the test sample if the probe would have the sensitivity for both Hg(II) and MeHg(II). However this is not precise, so this concentration was used to obtain the emission intensity due to MeHg(II). Finally it was replaced in a final calibration curve between **BD116** and MeHg(II) to obtain the MeHg(II) concentration of the test sample.

Replicate	Slope	Intercept	Adj. R^2	$C_{Hg(II)+MeHg(II)}$ calculated (M)	$Emission_{MeHg(II)}$ (a.u.)
1	1.0446×10^8	729.7091	0.9939	6.9856×10^{-6}	963.9614
2	1.0141×10^8	722.3993	0.9971	7.1236×10^{-6}	963.8077
3	1.3831×10^8	800.5733	0.9817	5.7882×10^{-6}	945.1240

Table S3. Curves parameters obtained after each calibration of **BD116**-Hg(II).

Mean (M)	Error (M)	Confidence Interval (M)
957.6310	18.2606	[939.3704; 975.8917]

Table S4. Statistical parameters of the regression of **BD116**-Hg(II).

To conclude, a last calibration between **BD116** and MeHg(II) was performed. In this way the emission intensities obtained in the previous calibration can relate to the analyte concentration. Thus replacing the emission intensity of MeHg(II) in the following curves equations it was obtained the MeHg(II) concentration in the cuvette:

Replicate	Slope	Intercept	Adj. R^2	$C_{MeHg(II)}$ (M)
1	2.7213×10^7	780.0583	0.9995	6.5252×10^{-6}
2	2.3092×10^7	765.7788	0.9959	8.3082×10^{-6}
3	2.0956×10^7	759.9339	0.9948	9.4339×10^{-6}

Table S5. Curves parameters obtained after each calibration of **BD116**-MeHg(II).

Mean (M)	Error (M)	Confidence Interval (M)	Theoretical Value (M)
8.0891×10^{-6}	2.4726×10^{-6}	[5.6165×10^{-6}; 10.562×10^{-6}]	7.11×10^{-6}

Table S6. Statistical parameters of the regression of **BD116**-MeHg(II).

According to the data shown above, it can be concluded that the calculated value and the theoretical one are very close, being included the second one within the confidence interval. Finally, considering the dilution effect committed to prepare the test sample (i.e. 30 μL were placed in the cuvette with the probe to a final volume of 2530 μL and also this 30 μL solution was prepared through a dilution of 120 μL of a more concentrated one to 1000 μL with water), the calculated concentration of the solution is:

$C_{theoretical, MeHg(II)} = 5.00 \times 10^{-3}$ M

$C_{calculated, MeHg(II)} = 5.68 \times 10^{-3}$ M $= 7.11 \times 10^{-6} \times 2530/30 \times 1000/120$

In conclusion, in this section it has been demonstrated how using the standard addition method and two selective probes for MeHg(II) or Hg(II) it is possible the speciation between both analytes in water in test samples containing mixtures of both. The value obtained in this section for the solution of methylmercury, served us as starting point to study the stability of this compound with time, i.e. its decomposition kinetics.

Stability: The stability of MeHg(II) was studied because the existing literature was unclear and contradictory in this respect. That is so because some authors ensure that this is a very stable species but for many others it is quite unstable and should be handle carefully because it may decompose. For instance, Aschner et al. ensured that MeHg(II) is chemically surprisingly stable.[3] This is so because concentrated acids mineralize MeHg(II) very slowly (it has a half-life of 300 days in 1 M H_2SO_4). In fact, degradation of MeHg(II) can take place by three main routes: light, biological and with strong oxidizing reagents. The photo-induced demethylation is maybe the most important route of degradation of organomercurials, especially in shallow places such as clear water lakes and the surface of oceans.[13] MeHg(II) is degraded not only by ultraviolet light (100-400 nm) but also by visible light (400-800 nm), and the decomposition rate is also controlled by the intensity of the wavelength apart from the wavelength strictly speaking. In deeper waters and seas the microbes become the main degradation agents.[14] The methylmercury decomposing process can took place by two routes, depending on environmental conditions. The reductive degradation (leading to CH_4 and Hg(0)) is favoured with high mercury concentrations and dominate in marine sediments. The oxidative degradation (which leads to CO_2 and Hg(II)) predominates at low mercury concentrations and in freshwater sediments. The last known effective pathway of degradation is with the use of strong oxidizing reagents (such as permanganate, halogens or reactive oxygen species).[15] These species can be formed by the irradiation with direct sunlight of water samples containing organic matter and oxygen, which leads to $^{\bullet}OH$, superoxide and $^1O_2$, species reactive enough for an efficient breakdown of the Hg(II)-CH_3 bond. However, many of this papers study the process in natural waters, marine sediments… and there are just a few papers related to the stability of "*ideal samples*" prepared in the laboratory. These articles usually study some of the more influential steps in the determination process[16], such as the drying conditions (better with lyophilization) or the temperature (always below 100 ºC), which can be used without causing any Hg species losses or interconversion. In our particular case, we could observe differences in the measurements between a freshly prepared MeHg(II) solution and another one prepared a couple of months ago and stored in the dark in an amber flask at room temperature. It seemed as if the sensor had lost affinity for this analyte, because the fluorescence produced was lower, whereas it was conserved for Hg(II). Thus we decided to develop a new procedure using our probes to study the degradation with time of MeHg(II) in organic-aqueous samples. To study MeHg(II) degradation in a aqueous-organic mixture (MeOH:H_2O, 20:80), we used a similar procedure to that explained in the previous section referring to the speciation and consisting in preparing a test sample and doing a titration according to the standard addition method. In particular, the concentrated solution of MeHg(II) prepared with a calculated concentration of 5.68×10^{-3} M was titrated once a week for one month, and each time the remaining concentration of MeHg(II) in solution was obtained. This first value of the solution freshly prepared was considered as the 100% amount of MeHg(II). This solution was stored in the dark inside an amber glass (to protect it from light), was only opened before measuring each day for a few seconds period (just enough time to obtain an aliquot, to protect it from air) and kept at room temperature (as indicated by the manufacturer, to decrease its degradation kinetics). A test sample containing 120 µL of this solution and diluted to a final volume of 1 ml with deionized water was also prepared. Finally, the $MeHg^+$ concentration remaining in the solution was determined by a fluorometric titration using the probe **BD116**, 2.5×10^{-5} M in MeOH:H_2O (20:80), a dye sensitive to this analyte and more sensitive than **BD119**. Experimentally 2500 µL of the probe solution were pipetted to a fluorescence cuvette and the spectrum recorded by exciting at 350 nm. Then 30 µL of the test sample were added to the cuvette, shaken and the spectrum was measured again.

[13] (*a*) T. Zhang and H. Hsu-Kim, *Nature Geosci.,* 2010, **3**, 473; (*b*) I. Lehnherr and V. L. St. Louis, *Environ. Sci. Technol.,* 2009, **43**, 5692; (*c*) M. Inoko, *Environ. Pollut. Ser. B,* 1981, **2**, 3.
[14] (*a*) T. Barkay and I. Wagner-Döbler, *Adv. Appl. Microbiol.,* 2005, **57**, 1; (*b*) R. S. Oremland, C. W. Culbertson and M. R. Winfrey, *Appl. Environ. Microbiol.,* 1991, **57**, 130.
[15] (a) J. Chen, S. O. Pehkonen and C. J. Lin, *Water Res.,* 2003, **37**, 2496; (*b*) I. Suda, M. Suda and K. Hirayama, *Arch. Toxicol.,* 1993, **67**, 365.
[16] L. Schmidt, C. A. Bizzi, F. A. Duarte, V. L. Dressler and E. M. M. Flores, *Microchem. J.,* 2013, **108**, 53.

Finally, the calibration curve was performed by successive additions of $MeHg^+$ 10^{-3} M, in a linear response concentration range for the probe. The data at 470 nm were picked up and considered to do the calculations. Each day four replicates were measured. The three curves regressions obtained on the first day were used for the next and the calculated concentration (5.68×10^{-3} M) was established as a 100% value because it is the maximum amount that this solution could contain, being calculated in the following days the percentage that still remains in solution as a simple rule of three. These data were obtained from the plot of the difference among the emission intensities at 470 nm for each measurement and the emission produced by the probe after adding 30 µL of the test sample.

Replicate	Slope	Intercept	Adj. R^2
1	2.7114×10^7	2.6792	0.9993
2	2.3346×10^7	9.8345	0.9951
3	2.1517×10^7	12.7509	0.9968

Table S7. Curves parameters obtained the first day for the complex **BD116**-MeHg(II).

The results recorded the following days were plotted also as the difference of the emission at 470 nm among the one obtained after the addition of the test sample and the others. The data were fitted to linear regressions and to compare them to the initial ones it was calculated the emission intensity caused by the hypothetical addition of a certain amount of MeHg(II) to the cuvette (such as 5.00×10^{-6} M). This concentration value was chosen because the calibration is centred on it and also shows a linear behaviour. The emission values obtained were then substituted in the line equations of the first day, to calculate which one is the real concentration that produces this emission enhancement. Finally, comparing the calculated concentration with the theoretical one (5.00×10^{-6} M), a MeHg(II) percentage is obtained. This percentage can be also related with the initial concentration (5.68×10^{-3} M) to know the remaining concentration in solution of $MeHg^+$. There after, if $MeHg^+$ was as unstable as we thought, this concentration should diminish over time. The results collected are shown below:

Day	1	8	15	23	28	36
Replicate 1 (%)		64.96	38.45	66.40	35.54	36.61
Replicate 2 (%)		69.32	38.52	70.98	35.15	36.39
Replicate 3 (%)		72.50	39.09	74.31	35.42	36.78
Mean (%)	100	68.90	38.70	70.60	35.37	36.60
$C_{MeHg(II),mean}$ (%)	5.68×10^{-3}	3.92×10^{-3}	2.20×10^{-3}	4.01×10^{-3}	2.01×10^{-3}	2.08×10^{-3}

Table S8. Degradation over time of a $MeHg^+$ 5.68×10^{-3} M solution in $MeOH/H_2O$ 20/80 media.

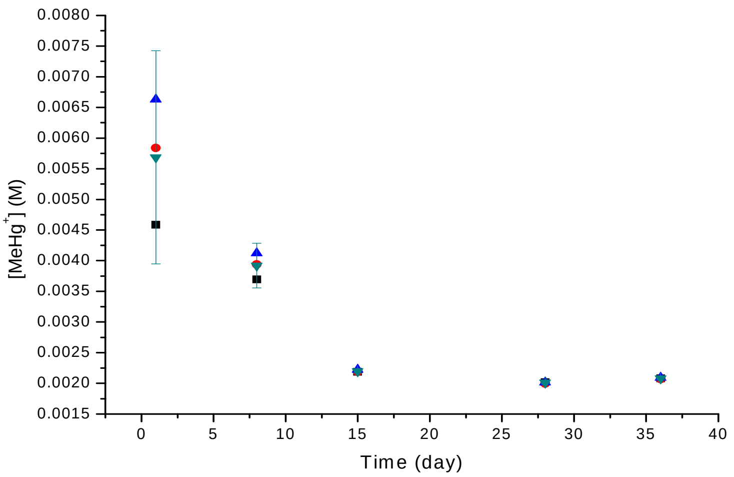

Figure S97: Degradation on time of a MeHg(II) 5.68 × 10^{-3} M solution in MeOH:H_2O (20:80).

These results reveal that within one week around a 30% of MeHg(II) has been degraded, and this amount increased to more than 60% in two weeks. It can be also concluded that the data recorded on the day 23rd are outliers by comparison with both previous and later data. The degradation compound formed had to be insoluble and it precipitated, because if another soluble mercury derivative had been formed, the probe would have detected it. Moreover, during the first two weeks the degradation behaviour is linear, which was used to check the results obtained by ICP-MS analysis:

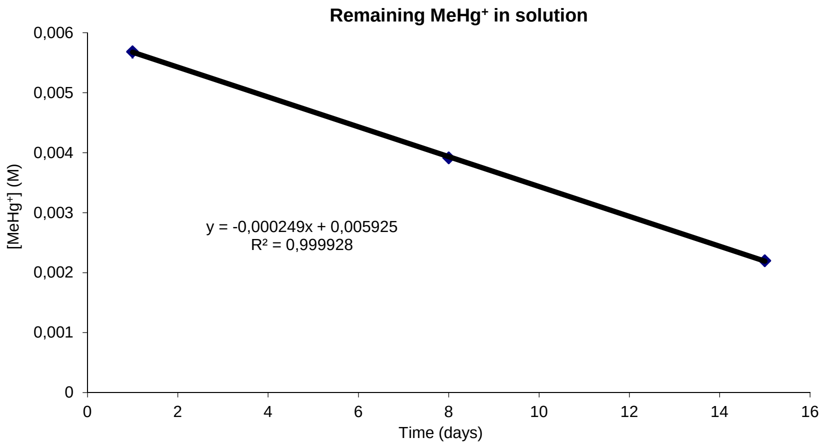

Figure S98: Degradation within 15 days of a MeHg(II) 5.68 × 10^{-3} M solution in MeOH/H_2O 20/80 media.

This result were verified by ICP-MS, a reference technique that determines the total amount of a desired metal in a sample, in this case Hg(II). The sample is subjected to acid digestion with nitric acid to destroy all the organic matter present and thus releasing the mercury retained inside. At the same time, it oxidizes mercury and leads the mercury derivatives to Hg(II). In our particular case a supernatant aliquot of the MeHg(II) 5.68 × 10^{-3} M was measured within 6 days, being found a MeHg(II) concentration of 4.43 × 10^{-3} M (888.03 ppm). By comparison of the result obtained by ICP-MS ($C_{MeHg(II)}$= 4.43 × 10^{-3} M) with the one reached using the straight line equation reported before (x= 6 days→ $C_{MeHg(II)}$= 4.43 × 10^{-3} M), it is possible to say that both methods provide exactly the same result. Thus it can be concluded that these probes and method are perfectly valid for the quantification of Hg(II) and MeHg(II) in mixtures of both and also for studying their stability. In regard to the stability it can be said that MeHg(II) degrades initially very quickly, until reaching an equilibrium concentration after 15 days with about 65% degraded. This result agrees with that described by Devai et al. where a sample of MeHg(II) in methylene

chloride was stored at different temperatures and to determine changes in $MeHg^+$ concentration with time.[17] They concluded that changes in the concentration measured over time were not influenced significantly by the storage temperature, but at the end of the 15-day storage period only about half the initial concentration remained in samples, being a bit higher for the sample stored at room temperature (51.2%) than for the one stored at -25 ºC (40.2%). Thus we think that MeHg(II) is precipitating and forming insoluble salts, because when the solution was shaken we could observe how the concentration increased up to 71% of initial value. It is therefore very important to work with freshly prepared solutions of this compound.

Job's-Plot: To determine the stoichiometry of a binding process, a Job's-Plot study was performed for each of the three complexes. The emission spectra was recorded in the fluorometer and then the data were treated, to obtain a plot of the fluorescence versus the mole fraction of Hg(II) or MeHg(II). From the maximum of each curve, the nature of the complex formed was obtained directly with the following results:

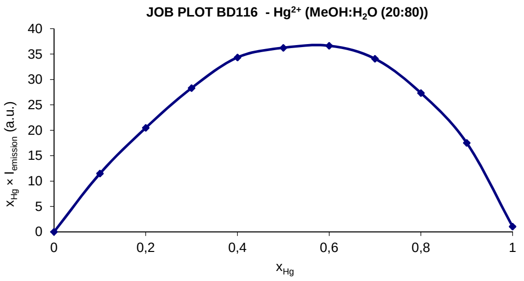

F. S99a

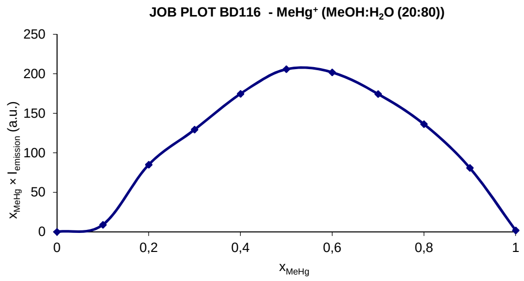

F. S99b

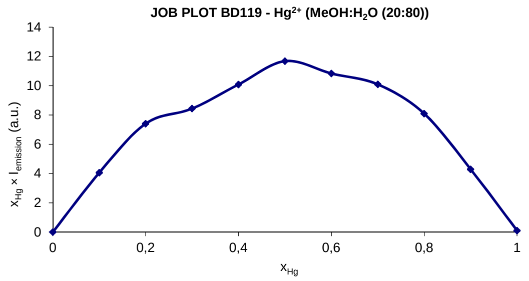

F. S99c

Figures S99a-c: Job's plot of the complexes in methanol:water (20:80). a) **BD116**-Hg(II) (λ_{exc}= 350 nm). b) **BD116**-MeHg(II) (λ_{exc}= 350 nm). c) **BD119**-MeHg(II) (λ_{exc}= 398 nm). C_{ligand}= 2.5 × 10^{-5} M, $C_{analyte}$= 3.92 × 10^{-4} M.

According to the results, it can be concluded that in all cases a complex 1:1 is formed between the probes BD116 and BD119 and the corresponding mercury derivative, because all curves reach a maximum for x_{Hg}= x_{MeHg}= 0.5. This result is in line with the quantum mechanics calculations and the NMR titrations, because there is only one sulphur atom in each fluorophore to bind to every mercury cation.

^{1}H-NMR titration: A titration was carried out in a ^{1}H-NMR Varian 300 MHz spectrometer to determine the binding zone for the formation of the mercury/probes complexes. The process consisted of the preparation of a NMR sample with the probe in a NMR-tube and the successive addition with a microsyringe of increasing amounts of Hg(II), shaking the tube and recording one spectrum after each addition. Due to solubility reasons of the mercury salt (mercury(II) perchlorate), both probe and analyte were dissolved in deuterated acetonitrile. At the end of the titration the following sequence of ^{1}H-NMR was recorded.

[17] I. Devai, R. D. Delaune, W. H. Patrick Jr. and R. P. Gambrell, *Org. Geochem.*, 2001, **32**, 755.

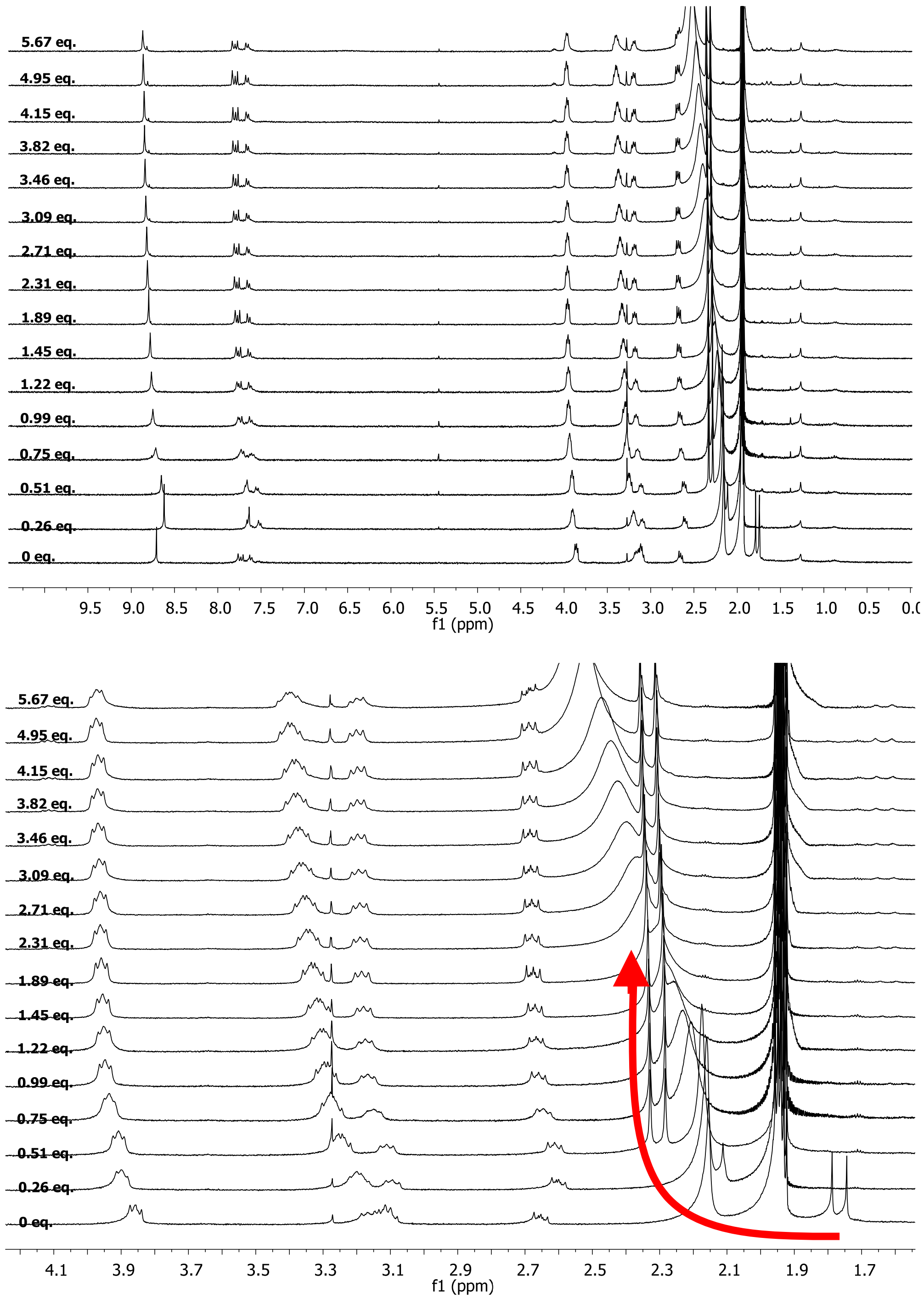

Figure S100: (Upper) ^{1}H-NMR titration spectra of BD116 5.0 × 10^{-3} M in CD_3CN with several Hg(II) additions ($C_{Hg(II)}$= 27.85 × 10^{-3} M). (Lower) Zoom in.

The images above show a downfield chemical shift for both methyl groups of the dimethylthiophosphinoic group, thus indicating that the coordination process takes place by the sulfur atom, something logic because of the known chalcogenophilicity of Hg(II) derivatives. Likewise, the other atoms affected with the complexation process are the two closest methylene groups of the piperazine moiety. In summary, the recognition process of Hg species takes place around the sulfur atom present in the molecule, a fact that the DFT calculations confirmed.

Mass Spectrometry Titration: We performed mass spectra of equimolecular mixtures of BD116 and mercury(II) perchlorate and compared to the mass spectrum of a pure sample of BD116. Mass spectra were taken in a Micromass AutoSpec machine, by electronic impact at 70 eV in both cases. From the comparison of spectra we found that the mass spectra of the mixture gave essentially the peaks corresponding to the intact BD116 and the characteristic cluster of peaks corresponding to Hg^{+}. The cluster of peaks corresponding to BD116-Hg^{2+} should appear at m/z 294, 293 and 292 but in the mass spectrum of the intact BD116 appear also a cluster at the same m/z so it could not be stated any difference. The peak corresponding to a possible oxidation product from BD116 should appear at m/z 370 but this peak did not appear in any of the mass spectrometry experiments we performed with increasing amounts of mercury(II) perchlorate in the mixture, therefore any trace of the sensing mechanism through oxidation of the fluorogenic probe was discarded.

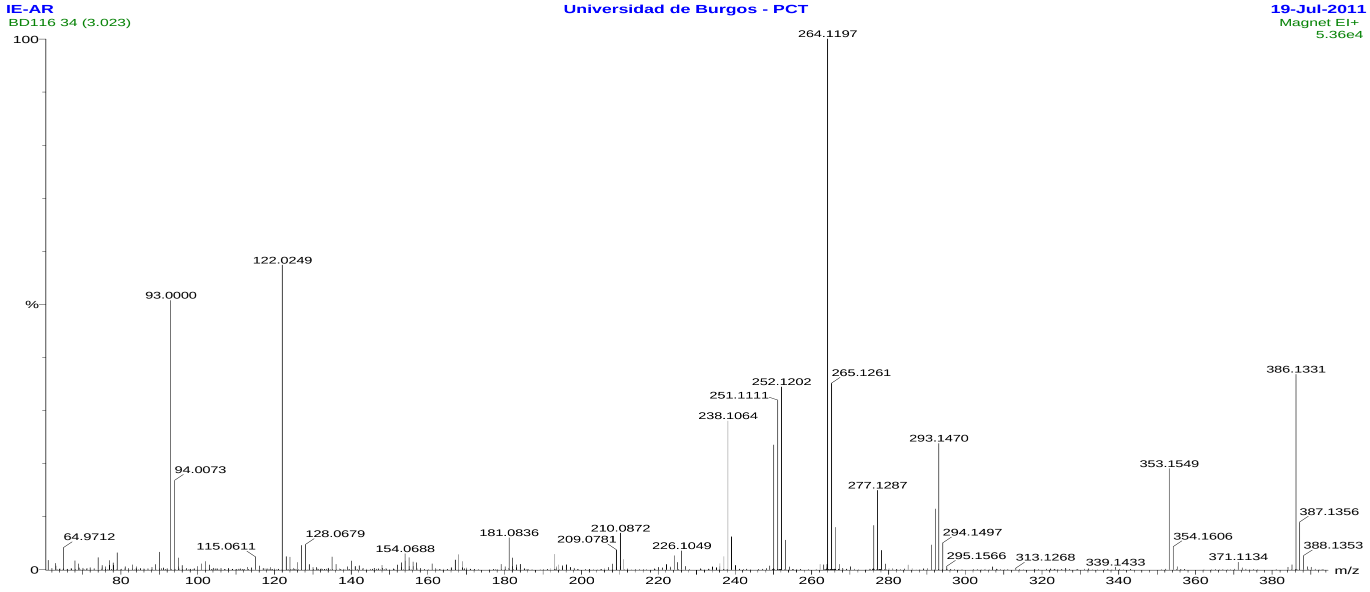

Figure S101: Mass spectrum of BD116

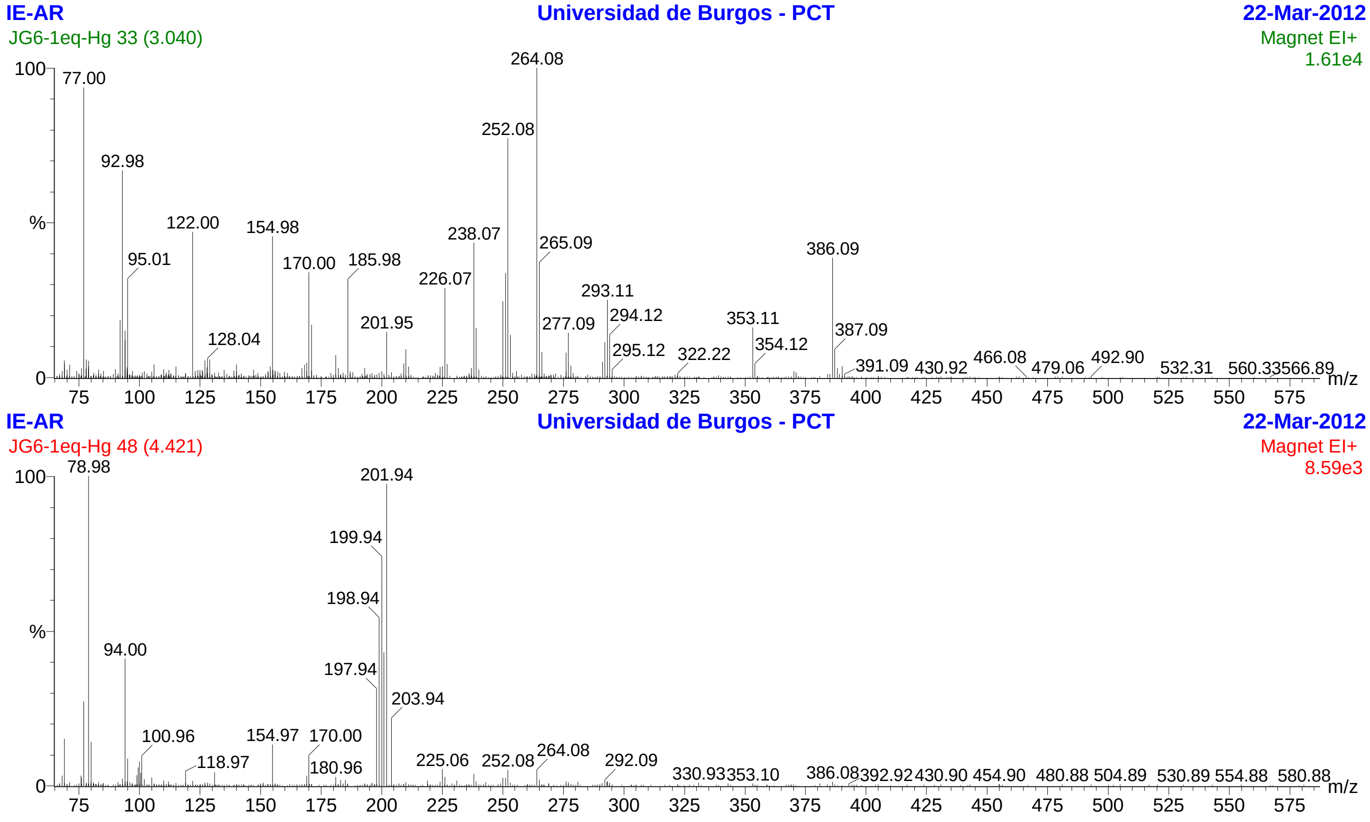

Figure S102: Mass spectra of a equimolecular mixture of BD116-mercury(II) perchorate.
